# Effectiveness of non-pharmacological interventions for fatigue in long term conditions: systematic review and network meta-analysis

**DOI:** 10.1136/bmjmed-2025-001746

**Published:** 2026-03-13

**Authors:** Joanna Leaviss, Jessica E Forsyth, Andrew Booth, David Coyle, George Daly, Sarah Davis, Helen Dawes, Vincent Deary, Kritica Dwivedi, Kate Fryer, Samantha McCormick, Marissa Martyn-St James, Julia Newton, Shijie Ren, Gillian Rooney, Anthea Sutton, Mon Mon-Yee, Christopher Burton

**Affiliations:** 1 Sheffield Centre for Health and Related Research, University of Sheffield, Sheffield, UK; 2 Devices for Dignity HTC, Sheffield, UK; 3 Medical School, University of Exeter, Exeter, UK; 4 Department of Psychology, Northumbria University, Newcastle upon Tyne, UK; 5 Patient and public contributor, Sheffield, UK; 6 Academic Health Science Network North East and North Cumbria, Newcastle upon Tyne, UK

**Keywords:** Health services, Internal medicine, Physical and rehabilitation medicine

## Abstract

**Objective:**

To assess the clinical effectiveness of non-pharmacological interventions for fatigue in adults with long term medical conditions.

**Design:**

Systematic review and network meta-analysis.

**Data sources:**

Medline, Embase, CINAHL, APA PsycInfo, Web of Science Core Collection, and the Cochrane Central Register of Controlled Trials, from database inception to 28 September-3 October 2023, and updated 23-24 September 2024.

**Eligibility criteria for selecting studies:**

Randomised controlled trials of non-pharmacological interventions for fatigue in long term medical conditions where fatigue was a criterion for inclusion, the primary target of the intervention, or the primary or co-primary outcome. Excluded were studies of fatigue in people with cancer, in relation to or after infection, or resulting from injuries or developmental disorders. Studies were limited to European-style healthcare systems.

**Results:**

88 randomised controlled trials were included, comprising 6636 participants for the end of treatment analyses, 1849 participants for the short term (≤3 months after the end of treatment) analyses, and 2322 participants for the long term (>3 months) analyses, allocated to one of 27 interventions. The most common condition studied was multiple sclerosis (51 studies). A range of interventions were identified, and heterogeneity was found within intervention groups and between individual interventions. Interventions varied by duration, delivery methods, and intensity. Compared with usual care, interventions based on cognitive behavioural therapy (CBT) significantly reduced fatigue at the end of treatment (standardised mean difference −0.63, 95% credible interval (CrI) −0.87 to −0.40, 17 studies) and at the long term follow-up (−0.40, −0.63 to −0.21, nine studies). Promotion of physical activity significantly reduced fatigue at all three time points: end of treatment (standardised mean difference −0.32, 95% CrI −0.62 to −0.01, seven studies), short term (−0.51, −0.84 to −0.17, one study), and long term (−0.52, −0.86 to −0.18, two studies). Self-management focusing on energy conservation was not significantly beneficial at the end of treatment (standardised mean difference −0.20, 95% CrI −0.52 to 0.12, 10 studies) or at the short term follow-up (−0.13, −0.51 to 0.25, seven studies) but at longer term follow-up, comparable benefit with other interventions was suggested (−0.42, −0.90 to 0.09, three studies). The standard deviation of the variation between studies in the end of treatment, short term, and long term network meta-analyses indicated moderate heterogeneity of studies in each of the analyses. No significant inconsistency was detected within the networks.

**Conclusions:**

Interventions that support individuals to increase physical activity or that are based on CBT were effective in reducing fatigue in people with long term medical conditions. The strength of the evidence was moderate to low. Although relatively few studies in any condition other than multiple sclerosis exist, the magnitude of effect seemed to be similar across different conditions.

**Systematic review registration:**

PROSPERO CRD42023440141.

**WHAT IS ALREADY KNOWN ON THIS TOPIC**

- Fatigue is common in long term medical conditions
- Clinical trials in specific conditions suggest benefit from non-pharmacologic interventions for fatigue

**WHAT THIS STUDY ADDS**

- Interventions that support individuals to increase physical activity or that are based on cognitive behavioural approaches were effective in reducing fatigue in people with long term medical conditions
- The strength of the evidence for these findings was moderate to low, with considerable heterogeneity between studies and within intervention categories

**HOW THIS STUDY MIGHT AFFECT RESEARCH, PRACTICE, OR POLICY**

- Clinical services should find ways to offer these interventions to people with fatigue associated with long term conditions
- Research should evaluate the feasibility and effectiveness of providing interventions in a transdiagnostic way, rather than for individual medical conditions

## Introduction

 Persistent fatigue is common in long term medical conditions^1^ and is estimated to affect 50% to >70% of people with a wide range of conditions, including rheumatoid arthritis,^2^ multiple sclerosis,^3^ chronic kidney disease,^4^ and type 2 diabetes.^5^ In a large multi-condition cohort study, transdiagnostic factors accounted for considerably more of the variance in fatigue than the medical conditions themselves, suggesting important transdiagnostic causal factors.^6^ Together with feelings of tiredness, fatigue includes a sense of needing to rest, or of difficulty in starting or sustaining voluntary effort.^7 8^ People with medical conditions typically describe their fatigue as “more than ordinary tiredness”^9^ with effects that go beyond the feeling of fatigue.^10 11^ As well as reduced fatigue and a return to meaningful activities,^12^ patients want their experience of fatigue to be validated.^13^ Many patients report feeling that others, including clinicians, do not take fatigue seriously.^14^

Fatigue is common in medical conditions, but correlates poorly with disease severity^15^,^18^ and often persists after the disease has been controlled.^19^ Similarities in fatigue across medical conditions exist, including associated factors,^6^ subjective experience, and impairment.^14^ Current models of fatigue include biological^20^ and psychosocial factors.^1 16^ These models can be viewed in a transdiagnostic framework similar to chronic pain (regardless of cause) and other persistent physical symptoms,^21^ with increasing interest in the role of altered signalling between the brain and body.^22^,^25^ Currently, no licensed drug treatments exist for fatigue in long term conditions.

Non-pharmacological interventions have been developed to overcome fatigue in medical conditions. These interventions include those focusing on physical activity (either managing or increasing activity), those that are more psychologically based, as well as a range of forms of non-invasive stimulation, body-mind practices, and nutritional supplementation. In practice, many fatigue rehabilitation and self-management programmes have multiple components. Because fatigue is increasingly understood in terms of processes in the body and brain, and signalling between the two,^22 24 25^ these different types of non-pharmacological interventions are scientifically plausible. To many patients with fatigue, however, this rationale is often not apparent. Thus proposed interventions may be seen as illogical (physical exercise when patients are already exhausted), stigmatising (psychological interventions implying that fatigue is all in the mind or can be overcome by thinking differently), or inappropriate (body-mind interventions being regarded by some as too alternative). These conceptual barriers to engagement with interventions are an important aspect of acceptability of interventions.^26^

We found two published network meta-analyses of non-pharmacological interventions for fatigue in specific conditions: multiple sclerosis (113 studies)^27^ and after a stroke (10 studies),^28^ as well as one meta-analysis of physical activity interventions across multiple conditions.^29^ We found no reviews of the effectiveness of other interventions for multiple conditions.

We conducted a systematic review and network meta-analysis to investigate the clinical effectiveness of non-pharmacological interventions for fatigue in long term conditions. We chose to conduct a network meta-analysis to compare multiple interventions. The scope of the review reflects the specification of the funders in their call for commissioned research, which specifically excluded fatigue after cancer, fatigue after infection (including myalgic encephalomyelitis or chronic fatigue syndrome), and fatigue in conditions where the diagnosis relies only on symptoms. This evaluation of clinical effectiveness comprises one part of a larger evidence synthesis about interventions for fatigue in long term medical conditions: health economic^30^ and qualitative components (Booth, in press) are reported separately.

## Methods

This systematic review was conducted and reported in accordance with the Cochrane Handbook for Systematic Reviews of interventions^31^ and the Preferred Reporting Items for Systematic Reviews and Meta-Analysis guidelines.^32^ The study eligibility criteria used the PICOS (population, intervention, comparison, outcome, and study design) framework. A change from the published protocol was a limitation on the included studies to countries with comparable healthcare systems to the UK.

### Study eligibility criteria

To be eligible for inclusion, studies had to be randomised controlled trials that met the criteria for population, intervention comparator, outcome, and setting.

#### Population

We included adults with a long term condition, based on the NHS definition as “an illness that cannot be cured but that can usually be controlled with medicines or other treatments.” The commissioning brief specifically excluded fatigue in people with cancer, in relation to or after infection (HIV, hepatitis C, long covid, and myalgic encephalomyelitis or chronic fatigue syndrome), or resulting from injuries or developmental disorders. Also excluded were conditions where symptoms, rather than observable pathology, were the defining features (eg, fibromyalgia or irritable bowel syndrome).

#### Interventions

We included studies of any non-pharmacological intervention in which a stated explicit aim or main outcome was to look at fatigue. The interventions included behavioural, exercise based, and nutritional interventions as well as a range of forms of non-invasive stimulation. We excluded interventions that were specific to a condition (eg, pulmonary rehabilitation in lung disease) or to a problem other than fatigue (eg, vestibular rehabilitation for balance problems in people with multiple sclerosis). Interventions could be delivered face to face or at a distance, and included technology assisted interventions.

#### Comparators

Comparators were usual care, waiting list control, sham, or placebo (for stimulation or nutritional interventions), another non-pharmacological intervention, or attentional control, such as education or information.

##### Outcomes

For the primary outcome, we required that studies reported an established measure for fatigue. We allocated three time points for follow-up: end of treatment, short term (up to three months after the end of treatment), and long term (>3 months after the end of treatment). Where studies reported multiple long term time points, we extracted data for each of these, with the primary analysis based on the longest follow-up data.

### Setting

Studies could be conducted in primary, secondary, or community based settings, but we only included studies which could feasibly be delivered in an outpatient or community based setting. We excluded studies conducted in countries with healthcare systems that were not comparable with the UK system.

### Information sources

A comprehensive search of bibliographic databases to identify randomised controlled trials was conducted from 28 September to 3 October 2023 and updated on 23-24 September 2024. Search strategies combined free text and thesaurus terms related to long term conditions (both specific conditions and general terms, such as chronic disease and long term illness), and terms for fatigue measures (specifically named scales, and general terms for fatigue and assessment). Methodological search filters were used to identify randomised controlled trials. No date or language limits were applied to the search. Table 1 provides details of the databases with search dates. Online supplementary methods 1 has full details of the search strategies.

**Table 1:** Information sources and search dates

Database (date of inception)	Date of first search	Date of updated search
Ovid Medline (1946)	28 September 2023	23 September 2024
Embase from Ovid (1974)	28 September 2023	23 September 2024
CINAHL from EBSCO (1981)	2 October 2023	23 September 2024
Web of Science Core Collection (Science Citation Index and Social Sciences Citation Index) (1900)	2 October 2023	23 September 2024
Cochrane Central Register of Controlled Trials (CENTRAL)	3 October 2023 (issue 10 of 12, October 2023)	24 September 2024 (issue 9 of 12, September 2024)

### Study selection and data collection

We carried out a two stage selection process for inclusion of studies (title and abstract and then full paper review) with Covidence (Veritas Health Innovation) to manage the selection process. Three reviewers (JL, GR, and CB) first reviewed 10% of the titles and abstracts according to prespecified inclusion and exclusion criteria. Concerns about the ambiguity of any criteria were resolved by team discussion. All remaining titles and abstracts were then scrutinised independently by two reviewers (Cohen's κ 0.58). Although this κ value suggests moderate agreement, the double selection process meant that each disagreement was discussed to reach resolution. Generally, conflicts were the result of one reviewer being consistently over inclusive. Common ambiguities were referred to clinical experts (eg, whether a nutritional intervention was classed as a supplement or the dose was too high and should be excluded as a pharmacological intervention). Full texts of potentially eligible studies were then assessed for eligibility. Discrepancies were resolved by discussion between the two reviewers in consultation with a third investigator (CB), if required. The most common discrepancies were about the cut-off criteria for inclusion where boundaries were blurred (eg, whether the intervention focus was managing fatigue or whether fatigue was one of multiple secondary outcomes in a general condition self-management intervention). The updated search was filtered with the same eligibility criteria.

Two reviewers (JL and GR) extracted the data, with the intervention characteristics, with the template for intervention description and replication statement.^33^ Data extraction aimed to reflect sources of complexity, such as: population differences (eg, diagnostic criteria of the included long term conditions); use of multiple components within interventions; expertise and skills of those delivering and receiving the intervention; the intervention context, including method and intensity of delivery; settings; time points of outcome measurement; and attrition. Results (estimates and corresponding standard errors, standard deviations, confidence intervals, or interquartile ranges) were also extracted by one of two reviewers (JL or GR), and checked again by the other reviewer. Given the large number of included studies, contacting the authors of the studies to enquire about missing or incomplete data or data that were only included graphically, was not feasible. Interventions were coded into categories based on the method described below.

#### Risk of bias assessment of included studies

Risk of bias assessment of all studies included in the network meta-analysis of this review was undertaken with an adapted version 2 of the Cochrane risk-of-bias tool (RoB2) for randomised controlled trials.^34^ Given the large number of included studies in our review, we adapted RoB2 to facilitate faster completion, reducing the number of signalling questions from 22 to 15 within five domains. Online supplementary methods 2 has a full description of these methods.

#### GRADE assessment

Review findings were synthesised with an adaptation of the Grading of Recommendations Assessment, Development, and Evaluation (GRADE) framework^35^ to assess the quality of the evidence (certainty in the evidence) for fatigue for each intervention compared with usual care, at each of the three time points analysed. We adapted GRADE to incorporate elements of Confidence in Network Meta-Analysis (CINeMA),^36^ a framework largely based on GRADE, modified to facilitate assessment of network meta-analyses. Although we adopted the assessment framework of CINeMA (eg, methods of assessing heterogeneity and inconsistency), we did not use the current CINeMA analysis platform directly because our analyses were conducted in a bayesian setting. We used a framework based on risk of bias, inconsistency, imprecision, and heterogeneity. We adopted a standardised mean difference threshold of 0.34 to indicate a clinically meaningful difference (detailed in Statistical analysis). Online supplementary methods 3 has a full description of the methods used for the GRADE assessment.

### Classification of conditions

An initial description of the condition (checked against exclusion criteria) was generated for all extracted studies. These descriptions were aggregated into broad disease categories (eg, musculoskeletal disorders). Within neurological disorders, multiple sclerosis and stroke were distinguished from other neurological disorders.

### Classification of interventions

After considering several options, we used a categorical approach to classify interventions, as had previously been used in the review of interventions for fatigue in multiple sclerosis.^27^ This approach recognises that many interventions are complex, with multiple components provided in different ways and in different amounts, such that a component based ontology classification would prove overly complicated for analysis. Instead, we used an iterative inductive process of categorisation in which a hierarchy of categories of interventions was developed from studies and then defined as a set of criteria. All studies were then mapped to these criteria.

The process involved several steps. Firstly, a simple description of the interventions in each arm was recorded by reviewers JL and GR during data extraction. Next, a clinical investigator (CB) reviewed these descriptions to generate an initial classification with draft criteria for each category. The same investigator then reviewed the full text descriptions of interventions and classified the interventions based on the draft criteria: during this process the criteria were edited and refined after discussions with other clinical investigators (HD, JN, and VD). These criteria were then reviewed and tested (for a sample of behavioural interventions) by independent checking of categorisation. Differences were resolved by discussion. The final criteria (online supplementary methods 4) were then reapplied to all included studies. In parallel with this approach, we grouped the individual intervention categories into higher level groups to produce a hierarchical taxonomy. We took this approach because many interventions had multiple (often overlapping) components, although in varying amounts.

### Use of patient focus group and other qualitative data to inform our analysis

From the patient focus groups and a parallel qualitative evidence synthesis (Booth, personal communication), we identified three key observations to guide decisions about inclusion of interventions and conditions for analysis. These observations were: the experience of fatigue is multifaceted and different for each individual (differences (and similarities) are as evident within conditions as between conditions); although few focus group participants had tried specific interventions for fatigue, none of those discussed was unacceptable to most participants; and personal circumstances and experience were important in valuing interventions. These observations informed our study design choices to carry out the primary analysis across conditions, to have no a priori restriction on interventions, and to recognise the importance of personal context in recommendations arising from the analysis. Online supplementary methods 5 has more data on the focus groups.

### Statistical analysis

The primary analysis consisted of three separate network meta-analyses, each corresponding to a different follow-up time point. We used standardised mean difference of the change in fatigue outcomes from baseline as the measure of effect, evaluated with Hedge's correction for small studies. Online supplementary methods 6 has the detailed methodology.

We generated networks of evidence at three time points (end of treatment, short term, and long term) and visualised these with network plots, with edge thicknesses between interventions proportional to the number of studies providing direct comparisons of the interventions, and node sizes proportional to the number of participants. Through assessment of the network plots and edge thicknesses, we appraised the level of direct evidence for each intervention and the areas of sparseness in the network. We conducted a network meta-analysis at each time point with a random effects model in view of the heterogeneity of study design, intervention, and population.^37^ We assumed that the variance (τ^2^) between studies was the same between all comparisons. Parameters of the random effects model were estimated with a bayesian framework. A weakly informative prior, a normal distribution with a mean equal to zero, and a standard deviation of 100 were chosen for the mean treatment effects. A weakly informative uniform prior bounded between 0 and 5 was chosen for the standard deviation between studies, and this value was multiplied by the factor $\frac{\surd 3}{\pi }$ to account for the transformation from the odds ratio scale and the standardised mean difference scale. For some analyses, we found fewer than five studies within the network and thus a more informative log normal prior was chosen for the variance between studies. Online supplementary methods 6 has more details. All analyses were conducted with WinBUGS,^38^ with the R package R2WinBUGS.^39^

Results are presented as the posterior median treatment effects with 95% credible intervals (CrI). The 95% predictive interval (PrI) is also presented in forest plots and shows the range in which the treatment effect of a new study is likely to fall, given the variation seen across existing studies. Rankograms, detailing the probabilities of each intervention's ranking, are also provided. Study heterogeneity was graded and interpreted according to the categories introduced by Ren et al.^40^ The standard deviation (τ) between studies was classified as low (0 to <0.06), moderate (≥0.06 to <0.33), high (≥0.33 to <0.83), or extremely high (≥0.83).^40^ Consistency was checked by comparing the posterior mean residual deviance from the unrelated mean effects model and the network meta-analysis model, and node-splitting analysis. If potential inconsistency was identified, sensitivity analyses were conducted by removing the study with potential inconsistency to explore the impact of the study on the synthesised results.^41^

We conducted four secondary analyses: to examine the sensitivity of the findings to different rules about preferred time point in longer term follow-up studies; to examine potential effects of small studies (<30 participants) within the network; to examine condition (or condition group) specific networks; and to exclude studies identified as pilot or feasibility studies. Finally, to translate findings from the standardised mean difference into clinically meaningful values, we took the estimated clinically important difference on the fatigue severity scale,^42^ mapped with an estimate of the baseline standard deviation of studies within the end of treatment network that used the fatigue severity scale, to calculate the corresponding clinically meaningful standardised mean difference.

### Patient and public involvement

This review included extensive patient and public involvement. Two of the investigators were appointed on the basis of their lived experience of fatigue in long term medical conditions. Also, we convened five focus groups of 25 people with fatigue associated with long term conditions, with the primary purpose of ensuring that any assumptions made about grouping interventions or conditions in the statistical analysis were compatible with patients' experiences.

Participants of focus groups were recruited by advertisement through national peer support organisations and community organisations in South Yorkshire. We invited and recruited purposively to obtain a diverse mixture of long term conditions and ethnic heritage.

Focus groups were co-led by patient and public involvement investigators (DC and SM) and KF. Participants consented to participation and discussions were recorded and transcribed for analysis. Each focus group met on three occasions. In the first meetings, focus groups explored important concerns in relation to the conduct of the review, particularly the similarities and differences in the experience of fatigue between conditions and between interventions. In the second meetings, the focus groups discussed the appropriateness of combining studies across different conditions and questions about acceptability and feasibility of different interventions. The third meetings focused on the emerging findings and sought guidance on the content and framing of future dissemination materials for patients and professionals.

## Results

**Figure 1:**
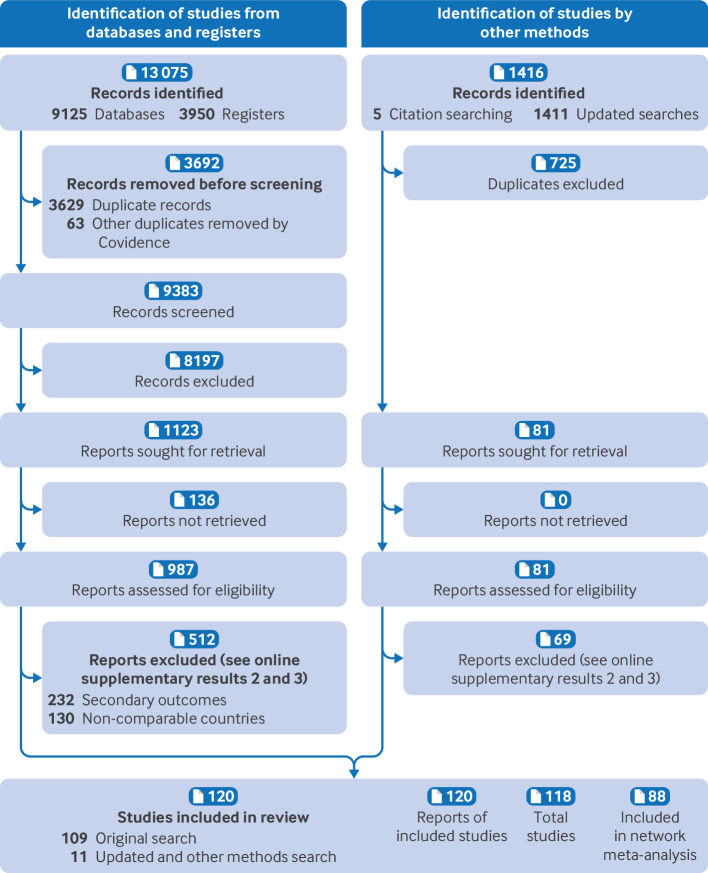
Preferred Reporting Items for Systematic Reviews and Meta-Analyses (PRISMA) flow diagram of inclusion of studies in the review

After de-duplication, we reviewed 10 108 titles and abstracts. From these, 1068 full text articles were assessed for eligibility, of which 118 studies reported in 120 manuscripts were eligible for inclusion. Of these, 88 studies, reported in 90 manuscripts, were included in the network meta-analysis (figure 1 and online supplementary results 1). Online supplementary results 2 lists the 30 studies not included in the network meta-analysis, and online supplementary results 3 provides the reasons for non-inclusion. Online online supplementary results 4 gives the time points that the results in the included studies were reported.

### Medical conditions

The most common condition was multiple sclerosis (51 studies). Six studies reported on stroke and five on other neurological conditions. Twenty studies covered a range of musculoskeletal and connective tissue disorders, ranging from osteoarthritis to systemic sclerosis. The remaining studies included inflammatory bowel disease (n=6), chronic kidney disease (n=3), and diabetes, hypothyroidism, heart disease, and psoriasis (n=1 each).

### Interventions

Table 2 shows the distribution of interventions by conditions, showing the wide range of types of interventions identified that came within our overall definition of non-pharmacological. As expected, we also found heterogeneity within each category of non-pharmacological intervention. Key sources of heterogeneity were: method of delivery (interventions were delivered to individuals and to groups, with in person, telephone, and online formats); length of the intervention (ranging from three weeks to six months, although most behavioural interventions lasted 6-12 weeks); and study populations (differences in method of diagnosis, severity, and duration of condition). The results of the network meta-analysis should take into consideration this heterogeneity, and currently, insufficient numbers of similar studies exist to determine which elements of the study characteristics are the most influential on their effects.

**Table 2:** Distribution of interventions by medical condition

Intervention category	Intervention	Long term conditions						
		Multiple sclerosis	Stroke	Other neurological conditions	Musculoskeletal	Inflammatory bowel disease	Other	Total
Exercise based (n=30)	Exercise supervised	12^56^,^65^	—	1^66^	1^67^	—	2^68 69^	16
	Exercise unsupervised	4^6270^,^72^	—	—	3^73^,^75^	—	—	7
	Promotion of physical activity	3^72 76 77^	—	—	4^67 74 78 79^	—	—	7
Self-management & CBT (n=40)	CBT based intervention	7^80^,^86^	3^43 48 87^	1^88^	3^26 78 89^	2^49 90^	3^44 91 92^-	19
	Active fatigue self-management	1^93^	1^45^	—	1^50^	—	—	3
	Conservative fatigue self-management	8^4694^,^100^	—	1^101^	1^102^	—	1^47^	11
	General self-management	1^99^	1^45^	—	4^51 52 103 104^	—	—	6
	Rehabilitation	1^105^	—	—	—	—	—	1
Mind and body (n=14)	Mind-body	7^53 54 85 97 100 106^	—	1^55^	2^75 107^	—	—	10
	Mindfulness based	2^72 108^	—	—	—	1^109^	—	3
	Other psychological	—	1^43^	—	—	—	—	1
Stimulation (n=14)	External stimulation	4^110^,^113^	—	—	—	—	—	4
	Acupuncture type	—	—	1^114^	—	1^115^	—	2
	Aromatherapy	—	—	—	—	—	1^116^	1
	Transcranial stimulation	4^117^,^120^	—	—	—	—	—	4
	Vagal nerve stimulation	—	—	—	2^121 122^	—	—	2
	Remote ischaemic conditioning	—	1^123^	—	—	—	—	1
Nutritional (n=6)	Nutritional supplement	1^124^	—	—	1^125^	2^126 127^	—	4
	Diet	1^128^	—	—	—	—	—	1
	Plant based	1^129^	—	—	—	—	—	1
	Total	57	7	5	22	6	7	104

CBT, cognitive behavioural therapy.

Our narrative synthesis describes the key areas of heterogeneity and refers to the studies included in the network meta-analysis. Online supplementary results 5-7 have a more detailed description of each study's intervention design, content, and delivery, and population characteristics by study (including those not included in the network meta-analysis).

Self-management interventions were the most represented in the included evidence, evaluated in 19 studies. Of these, 11 evaluated conservative fatigue self-management, three evaluated active self-management, and six evaluated general self-management. Nineteen studies evaluated CBT. In almost all of the studies, the populations were predominantly women, with only five exceptions.^43^,^47^ All of the CBT interventions selected the study populations for having fatigue alongside their long term condition, mostly with cut-off values on a range of fatigue scales, but with two studies allowing self-reported fatigue (yes/no) for entry into the study.^48 49^ Four of the self-management studies, however, did not require participants to have fatigue to enter the trial.^4650^,^52^ We found little variation in the ages of the population across all studies of self-management or CBT, with age usually within the range 40-60 years. Several methods were used to deliver the included interventions, to individuals or to groups. Interventions were delivered online, by telephone, or face to face, with many requiring work at home. Duration of the interventions ranged from three weeks to six months, although most CBT interventions lasted for eight or 12 weeks, and most self-management interventions lasted for 6-12 weeks.

We saw a similar pattern of heterogeneity in the 30 studies that evaluated physical activity interventions. Seven studies investigated promotion of physical activity interventions, 16 evaluated supervised exercise, and seven looked at unsupervised exercise interventions. Age ranges and number of men and women were similar to the self-management and CBT interventions. Although the self-management management interventions were often delivered by psychologists or occupational therapists, or by multidisciplinary teams, exercise interventions, when supervised, tended to be delivered by physiotherapists. On average, these interventions were slightly longer, with a duration of 12-20 weeks more commonly reported.

Fourteen mind and body interventions included 10 mind-body interventions, one mind based, and three mindfulness interventions. Only four of the mind-body interventions tested the intervention as the active treatment arm (two with pilates, one each with yoga and tai chi) while the remainder used relaxation or similar low-intensity intervention as a comparator treatment. Only two of these studies selected participants specifically for fatigue.^53 54^ The interventions frequently had a duration of eight weeks, and were delivered by yoga, pilates, or tai chi instructors, or psychologists specialised in mindfulness or mind-body approaches. Apart from the study of Walter et al,^55^ populations were predominantly women, with ages ranging from 18 to 66 years.

The stimulation intervention studies were generally small, but most studies selected participants who reported fatigue. External stimulation studies tended to focus on participants with multiple sclerosis, whereas the other intervention types varied, studying multiple sclerosis, inflammatory bowel disease, and stroke. The nutritional interventions were generally unique, with only one study in each category. Online supplementary results 5-7 have details of the study characteristics of the individual stimulation and nutritional interventions.

### Risk of bias

**Figure 2:**
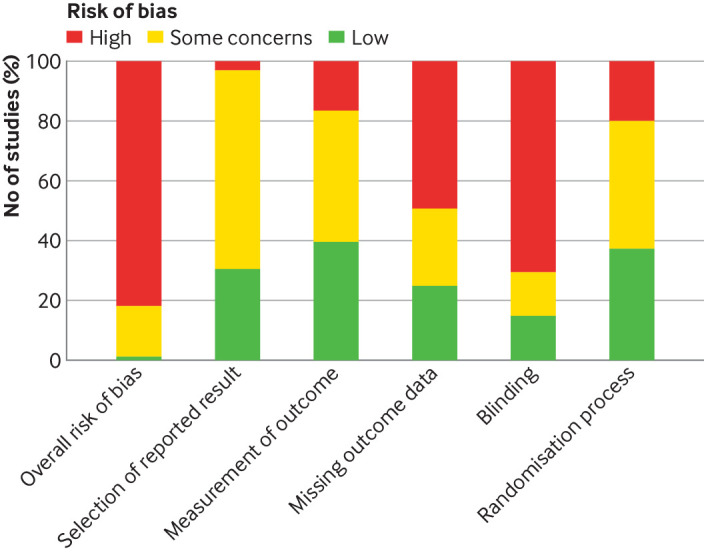
Summary of overall risk of bias and bias arising from selection of reported results, measurement of outcome, missing outcome data, blinding, and randomisation process for all included studies

Figure 2 gives an overall summary of the risk of bias assessments. Online supplementary results 8 report the risk of bias for individual studies. Although the body of evidence has some larger trials, many of the studies were small, under-powered, or pilot or feasibility studies. Also, most trials had at least one behavioural arm (eg, physical activity or self-management) and masking was impossible because of the nature of the intervention, resulting in a high risk of bias judgments, in accordance with RoB2 guidance.^34^

#### Risk of bias from the randomisation process

Although all of the included studies described themselves as randomised controlled trials, almost a quarter did not provide enough detail on the method of randomisation to make a judgment on the potential risk of bias. Studies that were judged to be at high risk of bias for randomisation were mostly because of the use of simple randomisation (alternate or manual). One study used a matched control group as a third arm and another created an additional control arm after randomisation for participants who declined their allocated interventions.

#### Risk of bias due to blinding

Blinding was rarely possible because of the nature of the interventions, most of which were behavioural. Although we acknowledge this practical restriction on study design, a risk of bias could still be introduced. Not masking participants and caregivers was the most common risk of bias across all studies. Also, in studies where masking was possible (eg, studies of interventions with placebo or sham controls), not all of these studies were masked.

#### Risk of bias due to missing outcome data

Sample size was based on a power calculation in only half of the studies. Many of the studies that did not use a power calculation were reported by the authors to be under-powered. About a quarter of the studies reported high attrition (>20%), and for many of these, the withdrawals were not balanced between study arms.

#### Risk of bias from measurement of the outcome

About half of the studies were at low risk of bias for measurement of outcome because of masking of the outcome assessment. In the remaining studies, masking of those conducting the outcome assessment was either specifically reported to have not been conducted or details on the process were not provided.

#### Risk of bias from selective reporting

Most studies were reported to be included in trial registries, mostly National Clinical Trial (ClinicalTrials.gov) or ISRCTN (International Standard Randomised Controlled Trial Number). Locating protocols for many studies within the time and resources of the review was not possible, and we therefore relaxed our criteria by using the study plans on the trial registries to make our judgments when a full protocol was not readily accessible. Where a protocol or study plan was identified, outcomes were mostly analysed according to the protocol.

### Primary analysis

#### Network geometry

**Figure 3:**
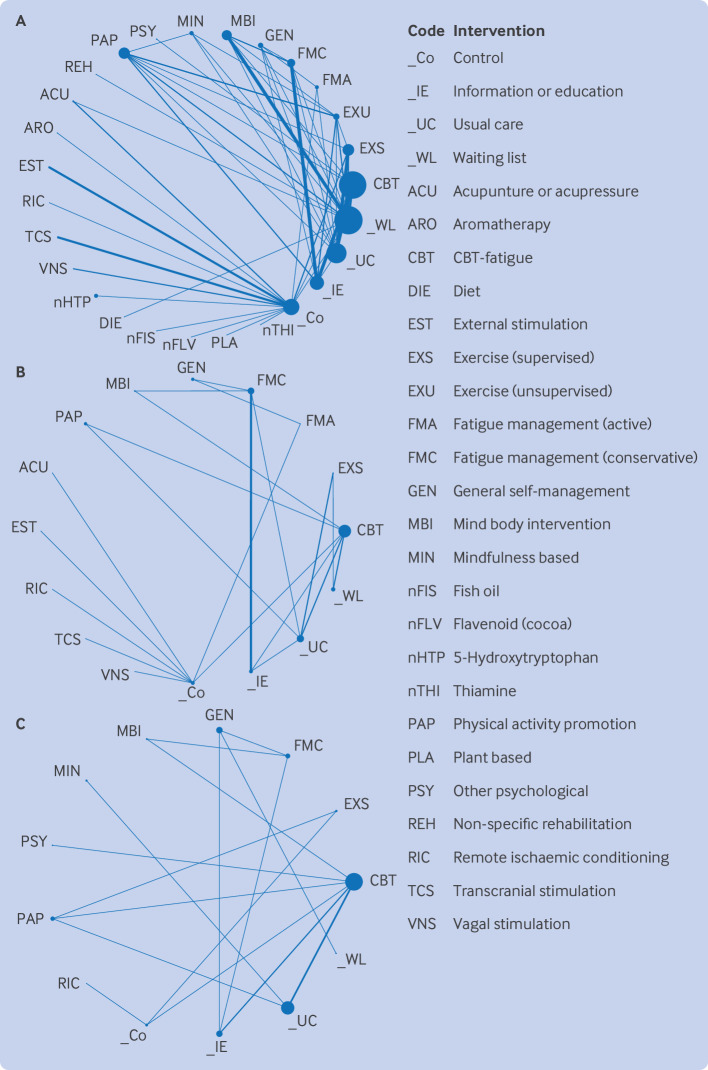
Network geometry for end of treatment (A), short term (B), and long term (C) analyses, indicating number of participants who received each intervention (size of node), number of studies contributing to the direct evidence, and comparisons between interventions (thickness of line)

Figure 3 shows network diagrams for the primary analysis at the different time points. The networks had 27 connected interventions (including control interventions) at the end of treatment, and 16 at the short term and 13 at the long term follow-up time points. The networks were evidenced from 84 studies (6636 participants), 24 studies (1849 participants), and 18 studies (2322 participants), respectively. To ensure connectivity of the networks, the control node includes control, placebo, and sham interventions. Information or education was also included as a comparator rather than an intervention. The end of treatment network showed greater connectivity compared with the short term and long term networks. For multiple intervention comparisons, the number of studies was small and regions within the network had evidence gaps. Therefore, some comparisons of interventions were based only on indirect evidence.

#### Synthesis of results

**Figure 4:**
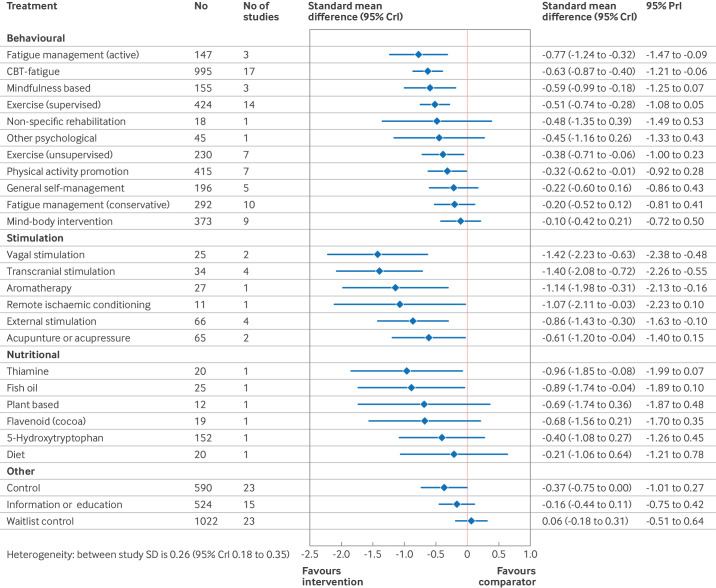
Estimated effects on fatigue outcomes of interventions, relative to usual care, at the end of treatment time point, with 95% credible (CrI) and predictive (PrI) intervals. Number of participants and number of studies are given for context. Broad intervention categorisation is also presented to help interpretation (behavioural, stimulation, nutritional, and other). Control node is displayed because this node functioned to ensure connectivity of the network, but was not an active intervention for consideration or recommendation. SD=standard deviation

Figures4^6^ show the estimated standardised mean differences for each intervention within the networks at each time point. A negative standardised mean difference indicates a reduction in fatigue from baseline relative to usual care. Within each of the forest plots, the final group (other) represents interventions typically included as comparator interventions. Online supplementary statistical data has the rankograms corresponding to the primary analyses. When assessing for inconsistency within the networks, no significant inconsistency was detected within the primary analysis (online supplementary statistical data).

**Figure 5:**
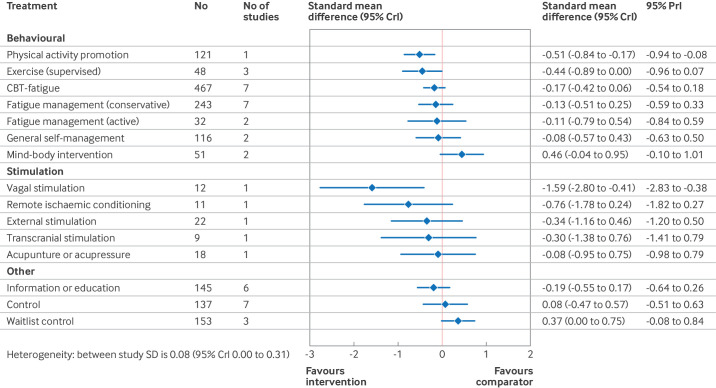
Estimated effects on fatigue outcomes of interventions, relative to usual care, at the short term follow-up, with 95% credible (CrI) and predictive (PrI) intervals. Number of participants and number of studies are given for context. Control node is displayed because this node functioned to ensure connectivity of the network, but was not an active intervention for consideration or recommendation. SD=standard deviation

**Figure 6:**
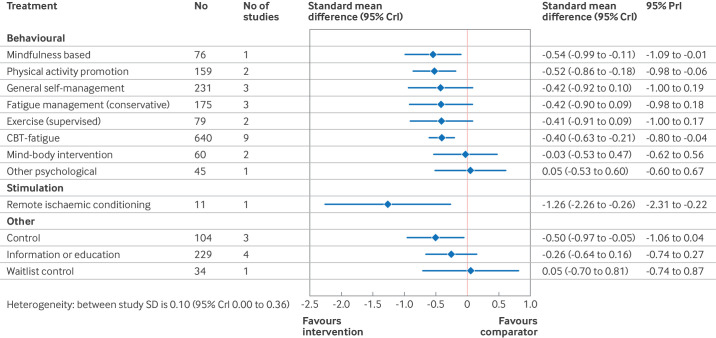
Estimated effects on fatigue outcomes of interventions, relative to usual care, at the long term follow-up, with 95% credible (CrI) and predictive (PrI) intervals. Number of participants and number of studies are given for context. Control node is displayed because this node functioned to ensure connectivity of the network, but was not an active intervention for consideration or recommendation. SD=standard deviation

Relative to usual care, CBT based interventions significantly reduced fatigue at the end of treatment (standardised mean difference −0.63, 95% CrI −0.87 to −0.40, 17 studies) and at the long term follow-up (−0.40, −0.63 to −0.21, nine studies). The reduction at the short term follow-up, with fewer studies, was smaller and not significant (−0.17, −0.42 to 0.06, seven studies). Active fatigue management significantly reduced fatigue at the end of treatment (standardised mean difference −0.77, 95% CrI −1.24 to −0.32, three studies) but this effect was not sustained in the short term (−0.11, −0.79 to 0.54, two studies) and no studies reported long term follow-up. Conservative self-management did not significantly change fatigue at the end of treatment (standardised mean difference −0.20, 95% CrI −0.52 to 0.12, 10 studies), or at the short term (−0.13, −0.51 to 0.25, seven studies) or long term (−0.42, −0.90 to 0.09, three studies) time points. Mindfulness based interventions significantly reduced fatigue at the end of treatment (standardised mean difference −0.59, 95% CrI −0.99 to −0.18, three studies) and at the long term follow-up (−0.54, −0.99 to −0.11, one study). We found no studies evaluating mindfulness at the short term follow-up for inclusion in the short term analysis.

Promotion of physical activity significantly reduced fatigue at all three time points: end of treatment (standardised mean difference −0.32, 95% CrI −0.62 to −0.01, seven studies), short term (−0.51, −0.84 to −0.17, one study), and long term (−0.52, −0.86 to −0.18, two studies) time points. Supervised exercise significantly reduced fatigue at the end of treatment (standardised mean difference −0.51, 95% CrI −0.74 to −0.28, 14 studies), but although standardised mean differences at the short term (−0.44, −0.89 to 0.00, three studies) and long term (−0.41, −0.91 to 0.09, two studies) time points were of comparable magnitude, they were not significant.

Non-invasive stimulation studies were few in number and small in size (14 studies, 228 participants). Although the observed effects at the end of treatment were large, only five studies (72 participants) reported effects at the short term follow-up and one study (11 participants) reported longer term follow-up. Nutritional studies reported end of treatment results only. Estimated effect sizes in the network meta-analysis for non-invasive stimulation and nutritional studies appear larger than reported in the original papers because standardised mean differences in the network meta-analysis were estimated relative to usual care, whereas these interventions were compared with sham or placebo (which in turn had a greater effect than usual care).

In each of the primary analyses, we found moderate heterogeneity, indicating potentially varying treatment effects between studies. This heterogeneity, however, was reduced in the short (0.08, 95% CrI 0.00 to 0.31) and longer term (0.10, 0.01 to 0.36) follow-up analyses compared with the end of treatment analysis (0.26, 0.18 to 0.35), which included a greater number of small and pilot trials.

#### Sensitivity analysis

Online supplementary results 9 presents the results of all of the sensitivity analyses.

#### Studies with multiple follow-up time points after three months

Five studies included in the long term analysis had data available from more than one time point after three months. We reanalysed these longer term data with the shortest follow-up point after three months. This approach had minimal impact on the results of the network meta-analysis.

#### Sensitivity analysis: condition specific analyses

Because of the sparsity of evidence for conditions other than multiple sclerosis, meaningful networks could only be constructed for: multiple sclerosis (at all three time points), musculoskeletal (end of treatment and long term time points), and inflammatory bowel disease, kidney disease, and stroke (end of treatment only). The networks were small other than for multiple sclerosis and thus estimated treatment effects were generally associated with large uncertainty.

#### Sensitivity analysis: exclusion of pilot and feasibility studies

Reanalysis after removal of pilot and feasibility studies had a minimal effect on the results of the network meta-analysis, although some interventions were excluded from the networks.

#### Sensitivity analysis: exclusion of studies with <30 participants

Reanalysis after removal of studies with <30 participants had a minimal effect on the results of the network meta-analysis, although some interventions were excluded from the networks.

#### Clinically important difference

We estimated that a clinically important difference of 3.6 points on the fatigue severity scale (range 9-63)^42^ was equivalent to a standardised mean difference in the network meta-analysis of 0.34. This finding indicates that the effect sizes that reached significance were also likely to be clinically meaningful.

Table 3 summarises the certainty of the evidence for the observed intervention effects based on our adapted GRADE framework at the short term and long term follow-up time points. At the long term (>3 months after the end of treatment) time point, the evidence for promotion of physical activity (interventions that supported individuals to increase physical activity) was rated as moderate. The evidence for CBT based interventions and mindfulness was rated as low. The strength of evidence for all other interventions was rated as very low.

**Table 3:** Summary of risk of Grading of Recommendations Assessment, Development, and Evaluation (GRADE) evidence: fatigue as outcome at long term follow-up (at least 13 weeks after end of treatment)

Intervention	Summary of findings	Quality of evidence	Reason for grading up or down
**Behavioural**			
Promotion of physical activity	Evidence of a medium effect from 2 studies with 159 participants	Moderate	Risk of bias, down 1
CBT based intervention	Evidence of a small effect from 9 studies with 640 participants	Low	Risk of bias, down 1; inconsistency, down 1
Mindfulness based intervention	Evidence of a medium effect from 1 study with 76 participants	Low	Risk of bias, down 1; inconsistency, down 1
General self-management	Non-significant evidence of a small effect from 3 studies with 231 participants	Very low	Risk of bias, down 1; inconsistency, down 1; imprecision, down 1; heterogeneity, down 1
Fatigue management (conservative)	Non-significant evidence of a small effect from 3 studies with 175 participants	Very low	Risk of bias, down 1; inconsistency, down 1; imprecision, down 1; heterogeneity, down 1
Exercise (supervised)	Non-significant evidence of a small effect from 2 studies with 79 participants	Very low	Risk of bias, down 1; inconsistency, down 1; imprecision, down 1; heterogeneity, down 1
Mind-body intervention	Non-significant evidence of unsubstantial effect from 2 studies with 60 participants	Very low	Risk of bias, down 1; inconsistency, down 1; imprecision, down 2; heterogeneity, down 2
Other psychological	Non-significant evidence of unsubstantial effect from 1 study with 45 participants	Very low	Risk of bias, down 1; inconsistency, down 1; imprecision, down 2; heterogeneity, down 2
**Non-invasive stimulation**			
Remote ischaemic conditioning	Evidence of a large effect from 1 study with 11 participants	Very low	Risk of bias, down 1; inconsistency, down 1; heterogeneity, down 1

CBT, cognitive behavioural therapy.

## Discussion

### Summary of main findings

Non-pharmacological interventions for fatigue in long term conditions other than multiple sclerosis have received relatively little attention in terms of large well conducted randomised studies, and have rarely been performed across conditions. Nevertheless, we found evidence of effectiveness of interventions that promote physical activity or are based on CBT. We found weaker evidence of benefit from approaches to self-management of fatigue that focused on energy conservation. These findings seemed to be relatively consistent across conditions, in keeping with other evidence of similarities in fatigue across conditions. The evidence generally had a high risk of bias, mostly because of our strict approach for judgment of the risk of bias involving blinding.

### Strengths and limitations of this study

The strengths of this review include the broad scope of eligible conditions and non-pharmacological interventions. This strength was reinforced by extensive patient and public involvement to ensure that assumptions made by researchers agreed with the lived experience of people with fatigue and long term conditions. We used a network meta-analysis to combine evidence across multiple conditions and interventions to maximise the available information based on our focus groups and qualitative evidence synthesis that identified substantial similarities across conditions. Despite the wide scope of the review, a large number of studies were conducted in people with multiple sclerosis or other neurological disorders.

Our study was limited by concerns common to other reviews of complex interventions (eg, eligibility of studies, choice of time points, categorisation of interventions, and large number of small studies). We identified many studies that included fatigue as one of multiple outcomes. Our inclusion criteria were restricted to studies where fatigue was the main focus (in terms of the population, proposed mechanism of intervention, or main outcome). This approach, however, created a grey area, particularly with self-management type interventions, where subjective judgment and resolution through discussion was often required. Another review team might have used a different operational approach. Although we had relatively strict criteria for inclusion, the small number of studies of general condition self-management which did meet our inclusion criteria had no significant effect, suggesting this approach was justified.

The time points when outcomes were measured varied between studies. We anchored time points to the expected end of treatment rather than enrolment. In practice, this strategy meant that the boundary between short term and long term treatment had a similar relation to time of enrolment only among studies with similar durations of intervention. Because the networks were sparse, the results of the statistical analyses could have been affected if the time point categories had differed. In particular, we considered that studies with the longest follow-up might be penalised relative to studies with shorter follow-up because of the effect of attrition or loss of effect over time, but the sensitivity analysis found no significant evidence of this effect.

Most studies were of behavioural interventions, often with pragmatic designs, and therefore masking of participants was not possible. Risk of bias was consequently rated as high for almost all studies. Although we considered conducting sensitivity analyses to account for studies that were judged to be at high risk of bias, generating a meaningful connected network for the end of treatment, short term, or long term analyses was not possible when restricted to studies at low or with some concerns for risk of bias because of the insufficient number of studies. As a result, the effect of risk of bias could not be examined quantitatively, and findings should therefore be interpreted with appropriate caution. The evidence base included studies with heterogenous interventions, comparators, and time points, as well as differences in the selected populations. The results of our network meta-analysis should be considered in terms of this heterogeneity, and currently insufficient similar studies exist to determine which elements of the study characteristics are the most influential on their effects.

Several interventions had multiple components, many of which were common to interventions across categories. Rather than attempt a component network meta-analysis in which many components were present but in different doses and forms, we developed a classification of interventions and applied a best fit principle of allocation. In some cases, this approach may have obscured the results (eg, the small category of mind-body interventions included several studies where relaxation was used as a low intensity comparator intervention). Furthermore, many different fatigue scales were used to measure outcomes across studies. This strategy necessitated statistical standardisation and may have increased uncertainty because of the inherent differences between the scales used, as a result of the potential variability in focus of the different fatigue scales. Future trials in this area would benefit from more standardised methodology, to reduce the observed uncertainty and enable more confident interpretation of the results across studies.

The reduction in observed heterogeneity between studies with a longer follow-up emphasises the importance of adequate study duration to generate robust conclusions. Many studies were small, including pilot or feasibility studies. These studies may be more likely to have a high risk of bias. Excluding pilot and feasibility studies, however, had little effect on the key findings, similar to excluding small studies, although with this approach, we saw loss of some whole intervention categories, highlighting the lack of large scale trials in these areas. A few studies evaluated emerging treatments, in particular non-invasive transcranial and vagus nerve stimulation. These studies showed potentially large short term effects, and although still at an experimental stage, these treatments warrant further research.

### Comparison with existing research

Our study was a transdiagnostic review of multiple non-pharmacological interventions for long term conditions. We were aware of two previously published reviews in multiple sclerosis^27^ and stroke,^28^ but we applied stricter inclusion criteria than these reviews to focus on fatigue outcomes. We identified one transdiagnostic review of promotion of physical activity for fatigue that found sustained benefits with an estimated standardised mean difference slightly larger than those from our network meta-analysis.^29^ We also restricted studies to those conducted in western healthcare systems and cultures to maximise transferability to those systems.

### Implications for practice, policy, and research

Our review findings provided evidence for the effectiveness of interventions that promoted an increase in physical activity or were based on CBT. Although we saw these effects across different long term conditions, all interventions were delivered within one condition. We found some evidence for mindfulness based intervention and some forms of non-invasive stimulation that may warrant further research. We found no clear best intervention. This finding is likely a result in part of individual patient needs and preferences, and the complex nature of the interventions. For pragmatic reasons, we categorised the interventions into groups, but the heterogeneity that we have highlighted, with the lack of firm understanding of the active mechanisms underlying successful interventions (particularly the complex behavioural interventions), means considerable overlap between intervention components exists. Drawing conclusions about best interventions is therefore done with caution. From our parallel focus group work and qualitative evidence synthesis, we recognise that offering patients a choice of interventions is better than aiming for one best treatment for all. We recommend that further research focuses on delivery of interventions in a transdiagnostic format.

## Conclusions

Our review found that interventions that support individuals to increase physical activity or that are based on CBT are effective in reducing fatigue in people with long term medical conditions. The strength of the evidence for these findings was moderate to low, and we found considerable heterogeneity between studies, even within intervention categories. Future larger scale trials are needed to minimise concerns that were apparent in our review (ie, small number of condition specific studies and the identified heterogeneity within populations, interventions, and controls). Although relatively few studies in conditions other than multiple sclerosis exist, the magnitude of effect seems to be similar across different conditions. Future research should examine the feasibility of delivering interventions across multiple conditions at the same time rather than for individual medical conditions.

## Supplementary material

10.1136/bmjmed-2025-001746 online supplemental file 1

10.1136/bmjmed-2025-001746 online supplemental file 2

## Data Availability

Data are available in a public, open access repository.
