# Supplementary material for: Effectiveness of non-pharmacological interventions for fatigue in long term conditions: systematic review and network meta-analysis
Source: BMJ Med. 2026 Mar 13;5(1):e001746. doi: 10.1136/bmjmed-2025-001746 (PMC12993367; doi:10.1136/bmjmed-2025-001746)
Supplement: online supplemental file 1 [file bmjmed-5-1-s001.pdf]

Effectiveness of non-pharmacological interventions for fatigue in long term conditions: systematic review and network meta-analysis

Supplementary Results

Table of Contents

SUPPLEMENTARY RESULTS 1 STUDIES INCLUDED IN NETWORK META-ANALYSIS 3

SUPPLEMENTARY RESULTS 2 CHARACTERISTICS OF POTENTIALLY ELIGIBLE STUDIES, NOT INCLUDED IN NETWORK META-ANALYSIS 8

SUPPLEMENTARY RESULTS 3 REASONS FOR NON-INCLUSION OF STUDIES IN NMA 13

SUPPLEMENTARY RESULTS 4 TIMING OF OUTCOME MEASURES 14

4.1 SELF-MANAGEMENT INTERVENTIONS 14
4.2 STIMULATION INTERVENTIONS 18
4.3 NUTRITIONAL INTERVENTIONS 19

SUPPLEMENTARY RESULTS 5 INTERVENTION CONTENT: STUDIES INCLUDED IN NMA 20

5.1 BEHAVIOURAL INTERVENTIONS 20

SUPPLEMENTARY RESULTS 6 INTERVENTION DELIVERY CHARACTERISTICS FOR STUDIES IN THE NMA 50

6.1 BEHAVIOURAL INTERVENTIONS 50
6.2 STIMULATION INTERVENTIONS 60
6.3 NUTRITIONAL INTERVENTIONS 62

SUPPLEMENTARY RESULTS 7 INTERVENTION CHARACTERISTICS, STUDIES NOT INCLUDED IN NMA 65

7.1 INTERVENTION CONTENT 65
7.2 INTERVENTION DELIVERY 78

SUPPLEMENTARY RESULTS 8 RISK OF BIAS SUMMARY PLOTS BY INTERVENTION GROUP 85

8.1 CBT-BASED INTERVENTIONS 85
8.2 FATIGUE SELF-MANAGEMENT INTERVENTIONS 86
8.3 MIND-BODY INTERVENTIONS 87
8.4 PHYSICAL ACTIVITY PROMOTION INTERVENTIONS 88
8.5 EXTERNAL STIMULATION INTERVENTIONS 89
8.6 NUTRITIONAL AND OTHER SUPPLEMENT INTERVENTIONS 90

SUPPLEMENTARY RESULTS 9 ADDITIONAL STATISTICAL REPORTING 91

9.1 PRIMARY ANALYSIS: INCONSISTENCY CHECKS 92
9.1.1 END OF TREATMENT (EOT) 92
9.1.2 SHORT TERM (ST) 92
9.1.3 LONGER TERM (LT) 93
9.2 PRIMARY ANALYSIS: RANKOGRAMS 94
9.2.1 EOT 94
9.2.2 ST 98
9.2.3 LT 101
9.3 NMA SCENARIO ANALYSIS: USE OF ALTERNATIVE DATA TO INFORM THE LT ANALYSIS 103
9.4 NMA SCENARIO ANALYSIS: EXCLUSION OF STUDIES WITH <30 PARTICIPANTS 104
9.4.1 EOT 104
9.4.2 ST 106
9.4.3 LT 107
9.5 NMA SCENARIO ANALYSIS: RELAXATION OF THE TRANSDIAGNOSTIC ASSUMPTION 108
9.5.1 EOT 108
9.5.2 ST 113
9.5.3 LT 115
9.6 NMA SCENARIO ANALYSIS: EXCLUSION OF PILOT AND FEASIBILITY STUDIES 117
9.6.1 EOT 117
9.6.2 ST 119
9.6.3 LT 120
9.7 REFERENCES 121

Supplemental Results 1
Studies included in Network Meta-analysis

Study	Condition	Diagnostic Criteria	Selected for fatigue	Fatigue selection criteria
Dalgas 2010	Multiple Sclerosis	Physician diagnosis + Expanded Disability Status Scale (EDSS) score between 3.0 and 5.5	Y	Significant fatigue with a Fatigue Severity Scale (FSS) score above 4
Englund 2022	Multiple Sclerosis	Physician diagnosis	Y	Fatigue Scale for Motor and Cognitive Functions (FSMC), with a score of ≥53
Escudero-Uribe 2017	Multiple Sclerosis – relapsing remitting	Physician diagnosis	Y	Fatigue Severity Scale (FSS) ≥4 and fatigue as one of the most disabling symptoms
Feys 2019	Multiple Sclerosis	N/R	Y	Fatigue Scale for Motor and Cognitive Functions (FSMC)N/R
Gervasoni 2014	Multiple Sclerosis	Expanded Disability Status Scale (EDSS) < 8	Y	N/R
Heine 2017	Multiple Sclerosis	Physician diagnosis and Expanded Disability Status Scale (EDSS) ≤ 6.0	Y	Checklist Individual Strength (CIS20r) fatigue subscale - severe fatigue⩾35
Kratz 2020	Multiple Sclerosis	Physician diagnosis	Y	N/R
Langeskov-Christensen 2022	Multiple Sclerosis	N/R	Y	N/R
Louie 2022	Multiple Sclerosis	Physician diagnosis and Expanded Disability Status Scale (EDSS) ≤ 6.5	Y	N/R
McCullagh 2008	Multiple Sclerosis	Relapsing -remitting or secondary progressive type multiple sclerosis only	Y	Modified Fatigue Impact Scale (MFIS), cut-off value of 38 to discriminate fatigued from non-fatigued participants.
Kucharski 2019	Rheumatoid Arthritis	Physician Diagnosis (ACR criteria)	Y	N/R
Ortiz-Rubio 2018	Parkinsons Disease	UK Brain Bank Criteria in the II-III Hoehn & Yahr stages	Y	N/R
Diaz 2023	Psoriasis	Physician diagnosis, without PsA and dermatology life quality index scores < 7	N	N/A
Pozehl 2008	Heart Failure	New York Heart Association class II, III or IV	Y	N/R
Geddes 2009	Multiple Sclerosis	Diagnosis of MS greater than 1 year	Y	N/R
Maurer 2018	Multiple Sclerosis	Physician diagnosis of RRMS	Y	N/R
Torkhani 2021	Multiple Sclerosis	Physician diagnosis at least 12 months prior and relapse free in previous 90 days	N	N/A
Durcan 2014	Rheumatoid Arthritis	Physician Diagnosis (ACR critiera)i	Y	N/R
Katz 2018	Rheumatoid Arthritis	Physician-diagnosed RA	Y	N/R

Tench 2003	Systemic Lupus Erythematosus	Physician Diagnosis (ACR)	Y	N/R
Lutz 2017	Multiple Sclerosis	Physician diagnosis	Y	N/R
Turner 2016	Multiple Sclerosis	Physician diagnosis	Y	Reporting fatigue (Modified Fatigue Impact Scale [MFIS] score greater than or equal to 20).
Bachmair 2022	Inflammatory arthritis	Inflammatory rheumatic disease diagnosed by specialist	Y	Reported fatigue to be a problem that was persistent (>3 months) with score(>6/10 on NRS for average fatigue over past 7 days)
Callahan 2014	Arthritis	Self-report any type of doctor diagnosed arthritis or join pain /stiffness with associated limitation.	N	N/A
Ehde 2015	Multiple Sclerosis	self-reported physician diagnosis	Y/N	Either pain, depression or significant fatigue symptoms.(score ³10 on the 5-item MFIS Short Form)
Gay 2023	Multiple Sclerosis – relapsing remitting	Physician diagnosis	Y	Fatigue at screening visit MFIS score >45
Moss-Morris 2012	Multiple Sclerosis	Physician diagnosis	Y	Significant fatigue indicated by a score of >4 on the Fatigue Scale
Pottgen 2018	Multiple Sclerosis	Self-reported diagnosis verified by a clinician letter in 50% random sample	Y	Fatigue indicated by >43 on the FSMC
Thomas 2013	Multiple Sclerosis	Physician diagnosis	Y	Fatigue impacting on daily life (FSS total score >4)
van den Akker 2017	Multiple Sclerosis	Physician diagnosis	Y	Experience of severe fatigue (CIS20r fatigue ≥35)
van Kessel 2008	Multiple Sclerosis	Physician diagnosis	Y	A fatigue score of 4 or greater on the Fatigue Scale
Mead 2022	Stroke	Any stroke between three months and two years previously	Y	Answered 'Yes' to both the Greater Manchester Stroke Assessment Tool fatigue questions
Nguyen 2019	Stroke	History of stroke	Y	Clinically significant self-reported fatigue (FSS ≥4) and/or poor sleep
Zedlitz 2012	Stroke	Any stroke >4 months before treatment	Y	Severe fatigue (CIS-fatigue ≥40)
Okkersen 2018	Myotonic Dystrophy (type 1)	Physician diagnosis	Y	Severely fatigued CIS20r subscale fatigue ≥35
Hewlett 2011	Rheumatoid Arthritis	Physician diagnosis	Y	Scoring ≥6 for fatigue in the past week (VAS)
Hewlett 2019a	Rheumatoid Arthritis	Physician diagnosis	Y	Fatigue severity ≥6/10 on an NRS
Jhamb 2023	Kidney Disease	Receiving in-centre treatment	Y/N	Cutoffs for a clinically significant level for fatigue, pain or depression
Picariello 2021	End-stage Kidney Failure	Physician diagnosis	Y	Experiencing Physician levels of fatigue, ≥18 on the Chalder Fatigue Scale
Artom 2019	Inflammatory Bowel Disease	Physician diagnosis	Y	Self-reported fatigue
Bredero 2023	Inflammatory Bowel Disease	Physician diagnosis	Y	Scoring ≥27 on the subjective fatigue scale of the CIS-20
Menting 2017	Type 1 Diabetes	Diagnosed for at least 1 year	Y	Score ³35 on the fatigue severity subscale of the CIS, with duration of more than 6 months
Rietberg 2014	Multiple Sclerosis	Physician diagnosis	Y	Chronic fatigue according to the MSCCPG definition
Clarke 2012	Stroke	Hospital stroke clinic or known to the local Stroke Foundation	Y	Experiencing fatigue FSS >3.9
Murphy 2024	Systemic Sclerosis	Physician diagnosis	Y	Moderate to severe fatigue (average score >4 AC on the FSS

Abonie 2020	Multiple Sclerosis	Physician diagnosis	N	N/A
Askari 2022	Multiple Sclerosis	Physician diagnosis	Y	Having a score >5.4 on the FSS
Blikman 2017	Multiple Sclerosis	Physician diagnosis	Y	Severely fatigued CIS20r subscale fatigue >35
Garcia Jalon 2013	Multiple Sclerosis	Physician diagnosis	Y	Scoring 4 or more on the FSS
Hersche 2019	Multiple Sclerosis	Physician diagnosis	Y	Fatigue Severity Scale score > 4
Hugos 2010	Multiple Sclerosis	Physician diagnosis	N	N/A
Hugos 2019	Multiple Sclerosis	Physician diagnosis	Y	Moderate to severe fatigue (scores>25 on the MFIS)
Kos 2016	Multiple Sclerosis	Physician diagnosis	Y	A high impact of fatigue (VAS score of at least 60)
Ghahari 2010		Self-reported diagnosis	Y	Minimum FSS score of 4
Murphy 2010	Osteoarthritis	Knee or hip OA>= 3 months with radiographic evidence	N	N/A
Farragher 2022	Kidney	Undergoing haemodialysis for ≥3 months	Y	Scored an average of ≥4 on items 5,7,8 and 9 on FSS
Austin 1996	Systemic Lupus Erythematosus	Physician diagnosis	Y	Moderate to severe fatigue due to SLE >2.5 FSS score
Feldthusen 2016	Rheumatoid Arthritis	Physician diagnosis	Y	Fatigue >50 on VAS
Hammond 2008	Inflammatory Arthritis	Rheumatoid or other inflammatory arthritis	N	N/A
Khan 2020	Systemic Lupus Erythematosus	Physician diagnosis	N	N/A
DeGiglio 2015	Multiple Sclerosis	Physician diagnosis	N	N/A
Callahan 2016	Arthritis	Self-reported doctor diagnosed arthritis of any type	N	N/A
Fleming 2019	Multiple Sclerosis	Self-reported physician diagnosis of MS	Y	MFISN/R
Fleming 2021	Multiple Sclerosis	Self-reported, physician diagnosed	Y	21-item Modified Fatigue impact Scale (MFIS)N/R
Sgoifo 2017	Multiple Sclerosis	Cliical diagnosis with at least one month free of relapses	N	N/A
Walter 2019	Parkinsons Disease	Physician diagnosis; score of 1.5 on the Modified Hoehn and Yahr Scale	N	N/A
Goren 2022	Crohns Disease	Physician diagnosis, Harvey-Bradshaw Index (HBI) between 5 and 16	N	N/A
Grossman 2010	Multiple Sclerosis	Relapsing -remitting or secondary progressive type multiple sclerosis only	N	N/A
Granja-Dominguez 2022	Multiple Sclerosis	McDonald Criteria	Y	FSS ≥ 4
Mostert 2005	Multiple Sclerosis	MS as defined by Poser	Y	FSS ≥ 3.5
Piatkowski 2009	Multiple Sclerosis	Clinically definite, relapsing-remitting MS	N	N/A
Voggenberger 2022	Multiple Sclerosis	McDonald Criteria	Y	FSS ≥ 36
Kluger 2016	Parkinsons Disease	UK Brain Bank criteria for PD	Y	Moderate/severe fatigue using International Parkinson & Movement Disorder Society UPDRS fatigue item
Horta 2020	Inflammatory bowe disease	IBD diagnosis (Harvey-Bradshaw score <5 and modified Mayo score ≤2)	Y	FACIT-FS score <40
Hawkins 2019		Clinically diagnosed with hypothyroidism.	N	N/A

Cancelli 2018	Multiple Sclerosis	Physician diagnosis	Y	mFIS >35
Charvet 2018	Multiple Sclerosis	Physician diagnosis	N	N/A
Salemi 2019	Multiple Sclerosis	Physician diagnosis	Y	MFIS >20
Tecchio 2015	Multiple Sclerosis	N/R	Y	MFIS >15
Aranow 2021	Systemic Lupus Erythematosus	Physican diagnosis (Revised ACR or SLICC)	N	N/A
Tarn 2023	Sjogrens Syndrome	Physician Diagnosis	N	N/A
Moyle 2023	Stroke	Any stroke .	Y	FSS-7 ≥ 4) for at least 4 weeks
Coe 2019	Multiple Sclerosis	RRMS (<10 year since diagnosis),	Y	FSS> 4/7
Arriens 2015	Systemic Lupus Erythematosus	SLE according to the 1997 revised ACR criteria	N	N/A
Bager 2021	Inflammatory Bowel Disease	Physician Diagnosis (Crohn's Disease or Ulcerative Colitis)		
Truyens 2022	Inflammatory Bowel Disease	N/R	Y	VAS ≥ 5
Chase 2023		N/R	N	N/A
Johnson 2006	Multiple Sclerosis	Physician Diagnosis		

Systemic Lupus Erythematosus	Systemic Lupus Erythematosus	ACR criteria for diagnosis	Y	Fatigue Severity Scale ≥ 3.7
Callahan 2008	arthritis	Self-reported arthritis	N	N/R
Chalah 2020	MS	McDonald criteria	Y	FSS/MFIS
Coe 2022	Parkinsons Disease	Clinical diagnosis; between 1-2 on Hoehn and Yahr scale	N	N/A
Coghe 2018	MS	Diagnosis based on the 2010 McDonald criteria	N	N/R
Daltroy 1995	Rheumatoid Arthritis	American College of Rheumatology criteria	N	N/R
DeCarvalho 2012	MS	McDonald Criteria	Y	FSS > 27
DeDoncker 2021	Stroke	Stroke > 3 months ago	Y	A score of FSS ≥4
Drory 2001	ALS	Revised El Escorial criteria	N	N/R
Finlayson 2011	MS	Self-reported diagnosis	Y	Fatigue score of 4 or greater (moderate to severe fatigue)
Gaede 2018	MS	Diagnosis based on 2005 revised McDonald criteria	Y/N	Either a score of ≥4 on the Fatigue Severity Scale or ≥12 Beck Depression Inventory
Hidding 2017	Parkinsons Disease	Advanced idiopathic PD Hoehn and Yahr stage: 2.2 ±	N	N/A
Irish 2017	Relapsing-remitting MS	Neurologist diagnosis (McDonald criteria)	N	N/A
Kim 2011	MS	Mcdonald criteria	Y	Fatigue for > two months (Fatigue Severity Scale (FSS) score >=4)
Kos 2007	MS	Physician diagnosis of MS	Y	High impact of fatigue score
Lee 2021	MS	N/R	Y	Severe fatigue (MFIS score 38)
Mateen 2020	MS	McDonald Criteria	Y	FSS ≥36

Mathiowetz 2005	MS	Phsycician diagnosis of MS	Y	FSS score of 4 or greater
McNelly 2016	IBD	Crohn’s disease or ulcerative colitis in remission, clinically and biochemically	Y	Self-reported fatigue
O’Connor 2019	IBD	Quiescent IBD (clinical & biochemical)	Y	Scoring 1 or more on Section I of the Crohn’s and Colitis UK IBD fatigue self-assessment scale.
Palsdottir 2020	Stroke	Acute stroke =- CHECK****	N	N/A
Plow 2022	MS	Physician confirmed diagnosis of MS	Y	Moderate to severe fatigue.
Robb-Nicholson 1989	Systemic Lupus Erythematosus	N/R	Y	N/R
Saoite 2014	MS	Diagnosed by physician	Y	FSS ≥4
Theander 2002	Sjogren's syndrome	Copenhagen criteria	N	N/A
van Kessel 2016	MS	neurologist diagnosis of MS	Y	Chalder fatigue score of 4 or greater
Voet 2014	Neuromuscular disorder	Known to study team or registered on neuromuscular database	Y	Severe fatigue (CIS-fatigue ≥35)
Vogelaar 2011	Crohns Disease	Phsycician diagnosis	Y	A high fatigue score (≥ 35 on the CIS dimension 1)

Supplemental Results 2

Characteristics of potentially eligible studies, not included in network meta-analysis

Avaux 2016	Systemic Lupus Eythematosus	ACR criteria for diagnosis	Y	Fatigue Severity Scale ≥ 3.7
Callahan 2008	arthritis	Self-reported arthritis	N	N/R
Chalah 2020	MS	McDonald criteria	Y	FSS/MFIS
Coe 2022	Parkinsons Disease	Clinical diagnosis; between 1-2 on Hoehn and Yahr scale	N	N/A
Coghe 2018	MS	Diagnosis based on the 2010 McDonald criteria	N	N/A
Daltroy 1995	Rheumatoid Arthritis	American College of Rheumatology criteria	N	N/A
DeCarvalho 2012	MS	McDonald Criteria	Y	FSS > 27
DeDoncker 2021	Stroke	Stroke > 3 months ago	Y	A score of FSS ≥4
Drory 2001	ALS	Revised El Escorial criteria	N	N/R
Finlayson 2011	MS	Self-reported diagnosis	Y	Fatigue score of 4 or greater (moderate to severe fatigue)
Gaede 2018	MS	Diagnosis based on 2005 revised McDonald criteria	Y/N	Either a score of ≥4 on the Fatigue Severity Scale or ≥12 Beck Depression Inventory
Hidding 2017	Parkinsons Disease	Advanced idiopathic PD Hoehn and Yahr stage: 2.2 ±	N	N/A
Irish 2017	Relapsing-remitting MS	Neurologist diagnosis (McDonald criteria)	N	N/A
Kim 2011	MS	Mcdonald criteria	Y	Fatigue for > two months (Fatigue Severity Scale (FSS) score >=4)
Kos 2007	MS	Physician diagnosis of MS	Y	High impact of fatigue score
Lee 2021	MS	N/R	Y	Severe fatigue (MFIS score 38)
Mateen 2020	MS	McDonald Criteria	Y	FSS ≥36
Mathiowetz 2005	MS	Phsycician diagnosis of MS	Y	FSS score of 4 or greater
McNelly 2016	IBD	Crohn’s disease or ulcerative colitis in remission, clinically and biochemically	Y	Self-reported fatigue
O’Connor 2019	IBD	Quiescent IBD (clinical & biochemical)	Y	Scoring 1 or more on Section I of the Crohn’s and Colitis UK IBD fatigue self-assessment scale.
Palsdottir 2020	Stroke	Admitted to hospital with acute stroke or in the chronic phase (1 year post stroke)	N	N/A
Plow 2022	MS	Physician confirmed diagnosis of MS	Y	Moderate to severe fatigue.
Robb-Nicholson 1989	Systemic Lupus Erythematosus	N/R	Y	N/R
Sabapathy 2011	MS	N/R	N	N/A
Saoite 2014	MS	Diagnosed by physician	Y	FSS ≥4
Theander 2002	Sjogren's syndrome	Copenhagen criteria	N	N/A
van Kessel 2016	MS	Neurologist diagnosis of MS	Y	Chalder fatigue score of 4 or greater

Voet 2014	Neuromuscular disorder	Known to study team or registered on neuromuscular database	Y	Severe fatigue (CIS-fatigue ≥35)
Vogelaar 2011	Crohns Disease	Phsycician diagnosis	Y	A high fatigue score (≥ 35 on the CIS dimension 1)
Vogelaar 2014	IBD	Diagnosis of IBD of at least 6 months	Y	CIS-fatigue score of ≥35

References:

Avaux, M., Hoellinger, P., Nieuwland-Husson, S., Fraselle, V., Depresseux, G., & Houssiau, F. A. (2016). Effects of two different exercise programs on chronic fatigue in lupus patients. *Acta Clinica Belgica*, *71*(6), 403-406.

Callahan, L. F., Mielenz, T., Freburger, J., Shreffler, J., Hootman, J., Brady, T., ... & Schwartz, T. (2008). A randomized controlled trial of the people with arthritis can exercise program: symptoms, function, physical activity, and psychosocial outcomes. *Arthritis Care & Research: Official Journal of the American College of Rheumatology*, *59*(1), 92-101.

Chalah, M. A., Grigorescu, C., Padberg, F., Kümpfel, T., Palm, U., & Ayache, S. S. (2020). Bifrontal transcranial direct current stimulation modulates fatigue in multiple sclerosis: a randomized sham-controlled study. *Journal of Neural Transmission*, *127*, 953-961.

Coe, S., Andreoli, D., George, M., Collett, J., Reed, A., Cossington, J., ... & Dawes, H. (2022). A feasibility study to determine whether the daily consumption of flavonoid-rich pure cocoa has the potential to reduce fatigue and fatigability in people with Parkinson's (pwP). *Clinical Nutrition ESPEN*, *48*, 68-73.

Coghe, G., Corona, F., Marongiu, E., Fenu, G., Frau, J., Lorefice, L., ... & Cocco, E. (2018). Fatigue, as measured using the Modified Fatigue Impact Scale, is a predictor of processing speed improvement induced by exercise in patients with multiple sclerosis: data from a randomized controlled trial. *Journal of Neurology*, *265*, 1328-1333.

Daltroy, L. H., Robb-Nicholson, C., Iversen, M. D., Wright, E. A., & Liang, M. H. (1995). Effectiveness of minimally supervised home aerobic training in patients with systemic rheumatic disease. *Rheumatology*, *34*(11), 1064-1069.

de Carvalho, M. L. L., Motta, R., Konrad, G., Battaglia, M. A., & Brichetto, G. (2012). A randomized placebo-controlled cross-over study using a low frequency magnetic field in the treatment of fatigue in multiple sclerosis. *Multiple Sclerosis Journal*, *18*(1), 82-89.

De Doncker, W., Ondobaka, S., & Kuppuswamy, A. (2021). Effect of transcranial direct current stimulation on post-stroke fatigue. *Journal of neurology*, *268*, 2831-2842.

Drory, V. E., Goltsman, E., Reznik, J. G., Mosek, A., & Korczyn, A. D. (2001). The value of muscle exercise in patients with amyotrophic lateral sclerosis. *Journal of the neurological sciences*, *191*(1-2), 133-137.

Finlayson, Marcia, et al. "Randomized trial of a teleconference-delivered fatigue management program for people with multiple sclerosis." *Multiple Sclerosis Journal* 17.9 (2011): 1130-1140.

Gaede, G., Tiede, M., Lorenz, I., Brandt, A. U., Pfueller, C., Dörr, J., ... & Paul, F. (2017). Safety and preliminary efficacy of deep transcranial magnetic stimulation in MS-related fatigue. *Neurology: Neuroimmunology & Neuroinflammation*, *5*(1), e423.

Hidding, U., Gulberti, A., Horn, A., Buhmann, C., Hamel, W., Koeppen, J. A., ... & Pötter-Nerger, M. (2017). Impact of combined subthalamic nucleus and substantia nigra stimulation on neuropsychiatric symptoms in Parkinson’s disease patients. *Parkinson’s Disease*, *2017*(1), 7306192.

Irish, A. K., Erickson, C. M., Wahls, T. L., Snetselaar, L. G., & Darling, W. G. (2017). Randomized control trial evaluation of a modified Paleolithic dietary intervention in the treatment of relapsing-remitting multiple sclerosis: a pilot study. *Degenerative neurological and neuromuscular disease*, 1-18.

Kim, E., Cameron, M., Lovera, J., Schaben, L., Bourdette, D., & Whitham, R. (2011). American ginseng does not improve fatigue in multiple sclerosis: a single center randomized double-blind placebo-controlled crossover pilot study. *Multiple Sclerosis Journal*, *17*(12), 1523-1526.

Kos, D., Duportail, M., D'hooghe, M. B., Nagels, G., & Kerckhofs, E. (2007). Multidisciplinary fatigue management programme in multiple sclerosis: a randomized clinical trial. *Multiple Sclerosis Journal*, *13*(8), 996-1003.

Lee, J. E., Titcomb, T. J., Bisht, B., Rubenstein, L. M., Louison, R., & Wahls, T. L. (2021). A modified MCT-based ketogenic diet increases plasma β-hydroxybutyrate but has less effect on fatigue and quality of life in people with multiple sclerosis compared to a modified paleolithic diet: a waitlist-controlled, randomized pilot study. *Journal of the American College of Nutrition*, *40*(1), 13-25.

Mateen, F. J., Vogel, A. C., Kaplan, T. B., Hotan, G. C., Grundy, S. J., Holroyd, K. B., ... & Videnovic, A. (2020). Light therapy for multiple sclerosis-associated fatigue: a randomized, controlled phase II trial. *Journal of Neurology*, *267*, 2319-2327.

Mathiowetz, V. G., Finlayson, M. L., Matuska, K. M., Chen, H. Y., & Luo, P. (2005). Randomized controlled trial of an energy conservation course for persons with multiple sclerosis. *Multiple Sclerosis Journal*, *11*(5), 592-601.

McNelly, A., Nathan, I., Monti, M., Grimble, G., Norton, C., Bredin, F., ... & Forbes, A. (2016). Inflammatory bowel disease and fatigue: the effect of physical activity and/or omega-3 supplementation.

O'Connor, A., Ratnakumaran, R., Warren, L., Pullen, D., Errington, A., Gracie, D. J., ... & Ford, A. C. (2019). Randomized controlled trial: a pilot study of a psychoeducational intervention for fatigue in patients with quiescent inflammatory bowel disease. *Therapeutic advances in chronic disease*, *10*, 2040622319838439.

Pálsdóttir, A. M., Andersson, G., Grahn, P., Norrving, B., Kyrö Wissler, S., Petersson, I. F., & Pessah-Rasmussen, H. (2015). A randomized controlled trial of nature-based post-stroke fatigue rehabilitation. *International Journal of Stroke*, *10*(S1).

Plow, M., Motl, R. W., Finlayson, M., & Bethoux, F. (2022). Response heterogeneity in a randomized controlled trial of telerehabilitation interventions among adults with multiple sclerosis. *Journal of telemedicine and telecare*, *28*(9), 642-652.

Robb-Nicholson, L. C., DALTROY, L., EATON, H., GALL, V., WRIGHT, E., Hartley, L. H., ... & Liang, M. H. (1989). Effects of aerobic conditioning in lupus fatigue: a pilot study. *Rheumatology*, *28*(6), 500-505.

Sabapathy, N. M., Minahan, C. L., Turner, G. T., & Broadley, S. A. (2011). Comparing endurance-and resistance-exercise training in people with multiple sclerosis: a randomized pilot study. *Clinical rehabilitation*, *25*(1), 14-24.

Saiote, C., Goldschmidt, T., Timäus, C., Steenwijk, M. D., Opitz, A., Antal, A., ... & Nitsche, M. A. (2014). Impact of transcranial direct current stimulation on fatigue in multiple sclerosis. *Restorative neurology and neuroscience*, *32*(3), 423-436.

Theander, E., Horrobin, D. F., Jacobsson, L. T., & Manthorpe, R. (2002). Gammalinolenic acid treatment of fatigue associated with primary Sjögren's syndrome. *Scandinavian journal of rheumatology*, *31*(2), 72-79.

Van Kessel, K., Wouldes, T., & Moss-Morris, R. (2016). A New Zealand pilot randomized controlled trial of a web-based interactive self-management programme (MSInvigor8) with and without email support for the treatment of multiple sclerosis fatigue. *Clinical Rehabilitation*, *30*(5), 454-462.

Voet, N., Bleijenberg, G., Hendriks, J., de Groot, I., Padberg, G., van Engelen, B., & Geurts, A. (2014). Both aerobic exercise and cognitive-behavioral therapy reduce chronic fatigue in FSHD: an RCT. *Neurology*, *83*(21), 1914-1922.

Vogelaar, L., Van't Spijker, A., Vogelaar, T., van Busschbach, J. J., Visser, M. S., Kuipers, E. J., & der Woude, C. J. V. (2011). Solution focused therapy: A promising new tool in the

management of fatigue in Crohn's disease patients: Psychological interventions for the management of fatigue in Crohn's disease. *Journal of Crohn's and Colitis*, *5*(6), 585-591.

Vogelaar, L., van't Spijker, A., Timman, R., van Tilburg, A. J., Bac, D., Vogelaar, T., ... & van der Woude, C. J. (2014). Fatigue management in patients with IBD: a randomised controlled trial. *Gut*, *63*(6), 911-918.

Supplemental Results 3

Reasons for non-inclusion of studies in NMA

Studies not included in NMA	
**Study**	**Reason for exclusion from NMA**
Avaux 2016	Graphical only
Callahan 2008	No SDs for EOT, means only
Chalah 2020	No Ns for each intervention group
Coe 2022	Graphical data only
Coghe 2018	Wilcoxon test statistics only, no means
Daltroy 1995	Means only or group difference no SDs
De Carvelho 2012	Graphical only
De Doncker 2021	Graphical only
Drory 2001	Graphical only
Finlayson 2011	Between group t-test only, no means only Cohen's D
Gaede 2018	No means and SDs, graphs and reduction numbers
Hidding 2017	Individual patient data only
Irish 2017	Graphs and percentage increases only
Kim 2011	Graphical only, means only for baseline
Kos 2007	Change scores compares groups
Lee 2021	Graphical only
Mateen 2020	No SDs
Mathiowetz 2005	Data is difference between groups
McNelly 2016	4 x 4 factorial with merged groups
O'Connor 2019	No SDs
Palsdottir 2020	No SDs
Plow 2022	Beta coefficients or graphical only
Robb-Nicholson 1987	Correlations plus baseline data only
Sabapathy	Intervention arms the same category
Saiote 2014	Fatigue scores graphical or means by responders/non responders
Theander 2002	Correlations only
van Kessel 2016	2 arms same intervention category
Voet 2014	Median and ranges only
Vogelaar 2011	No Means, % of participants with decreased fatigue
Vogelaar 2014	No Means, % of participants with decreased fatigue

Supplemental Results 4
Timing of outcome measures

4.1 Self-management interventions

Behavioural Interventions						
**Study**	**Population**	**Intervention duration (EOT)**	**Short term**	**Long term**	**Short Long term (if any)**	
**Self-Management**						
**Fatigue self-management - conservative**						
Abonie 2020	MS	4 weeks				
Askari 2022	MS	12 weeks				
Blikman 2017	MS	4 months	10 weeks	36 weeks		
Farragher 2022	Kidney	8 weeks	12 weeks			
Finlayson 2011	MS	6 weeks	3 months	3 months		
GarciaJalon 2013	MS	5 weeks	3 months			
Ghahari 2010	Chronic	7 weeks	3 months			
Hersche 2019	MS	3 weeks	3 months			
Hugos 2010	MS	6 weeks				
Hugos 2019a	MS	6 weeks	3 months	6 months		
Kos 2016	MS	3 weeks	3 months			
Mathiowetz 2005						
Murphy 2010	OA	2 weeks	10 weeks			

**Fatigue self-management - active**						
Clarke 2012	Stroke	6 weeks	3 months			
Murphy 2024	Sys. Sclerosis	12 weeks				
O’Connor 2019	IBD	6 months				
Rietberg 2014	MS	12 weeks	12 weeks			
Vogelaar 2014	IBD	3 months		3 months		
**General self-management**						
Austin 1996	SLE	6 months				
Feldthusen 2016	RA	12 weeks		6 months		
Hammond 2008	RA			12 months	6 months	
Khan 2020	SLE	16 weeks				
**CBT – fatigue**						
Artom 2019	IBD	8 weeks		10 months	4 months	
Bredero 2023	IBD	8 weeks				
Ehde 2015	MS			10 months	4 months	
Gay 2023	MS	6 weeks		12 months	6 months	
Hewlett 2011	RA	6 weeks				
Hewlett 2019a	RA	6 weeks		46 weeks	20 weeks	
Jhamb 2023	Kidney	12 weeks				
Mead 2022	Stroke	4 months	2 months			
Menting 2017	T1 Diabetes	5 months				
Moss-Morris 2012	MS	10 weeks				
Nguyen 2019	Stroke	2 months	2 months			

Okkersen 2018	MD	10 months				
Picariello 2021	Kidney	12 weeks				
Pottgen 2018	MS	12 weeks	12 weeks			
Thomas 2013	MS	6 weeks		1 month	4 months	
van Kessel 2008	MS	8 weeks	3 months	6 months		
Van Kessel 2016	MS	10 weeks				
van den Akker 2017	MS	4 months	10 weeks	36 weeks		
Zedlitz 2012	Stroke	12 weeks		6 months		
**Physical Activity**						
**Physical activity promotion**						
Bachmair 2022	Inflammatory	22 weeks	6 weeks	34 weeks		
Callahan 2014	Arthritis	20 weeks				
Lutz 2017	MS	6 weeks				
Turner 2016	MS	6 months				
**Exercise – supervised**						
Dalgas 2010	MS	12 weeks	12 weeks			
Diaz 2023	PsO	16 weeks				
Englund 2022	MS	12 weeks				
Escudero-Uribe 2017	MS	12 weeks				
Heine 2017	MS	16 weeks		36 weeks		
Feys 2019	MS	12 weeks				
Gervasoni 2014	MS	2 weeks				
Kratz 2020	MS	8 weeks				

Kucharski 2019	RA	20 weeks		12 months		
Langeskov-Christensen 2022	MS	24 weeks				
Louie 2022	MS	12 weeks	12 weeks			
McCullagh 2008	MS	12 weeks	3 months			
Ortiz-Rubio 2018	PD	8 weeks				
Pozehl 2008	HF	24 weeks				
**Exercise – unsupervised**						
Durcan 2014	Arthritis	12 weeks				
Geddes 2009	MS	12 weeks				
Katz 2018	RA	21 weeks				
Maurer 2018	MS	6 months				
Tench 2003	SLE	12 weeks				
**Active recreational**						
**Rehabilitation**						
DeGiglio 2015	MS	8 weeks				
**Mindbody**						
Callahan 2016	Arthritis	8 weeks				
Fleming 2019	MS	8 weeks				
Fleming 2021	MS	8 weeks				
Walter 2019	PD	8 weeks				
Sgoifo 2017	MS	8 weeks				
**Mindfulness**						

Goren 2022	Crohn’s	3 months				
Grossman 2010	MS	8 weeks		6 months		
Torkhani 2021	MS	8 weeks				

4.2 Stimulation interventions

Study	Population	Intervention duration (EOT)	Short term	Long term	Short Long term (if any)	
**Vagal stimulation**						
Aranow 2021	SLE	5 days	1 week			
Tarn 2023	Sjogren’s	54 days				
**Trans Cranial stimulation**						
Cancelli 2018	MS	5 days				
Chalah 2020	MS	5 days				
Charvet 2018	MS	4 weeks				
Salemi 2019	MS	2 weeks	1 month			
Tecchio 2015	MS	5 days				
**External stimulation**						
Granja-Dominguez 2022	MS	4 weeks	3 months			
Mostert 2005	MS	4 weeks				
Piatkowski 2009	MS	12 weeks				
Voggenberger 2022	MS	2 weeks				
**Aromatherapy**						
Hawkins 2019	Hypothyroidism	14 days				

**Acupuncture/acupressure**						
Horta 2020	IBD	8 weeks	8 weeks			
Kluger 2016	PD	6 weeks				
**RIC**						
Moyle 2023	Stroke	6 weeks	6 weeks	18 weeks		

4.3 Nutritional Interventions

**Study**	**Population**	**Intervention duration (EOT)**	**Short term**	**Long term**	**Short Long term (if any)**	
**Fish oil**						
Arriens 2015	SLE	6 months				
**Thiamine HD**						
Bager 2021	IBD	4 weeks				
**5-HTP**						
Truyens 2022	IBD	8 weeks				
**Flavenoid - cocoa**						
Coe 2019	MS	6 weeks				
**Diet**						
Chase 2023	MS	16 weeks				
**Plant**						
Johnson 2006	MS	4 weeks				

Supplemental Results 5
Intervention content: studies included in NMA
5.1 Behavioural Interventions

Tables of intervention characteristics for studies in the NMA

Behavioural Interventions						
**Study/ Population**	**Pop.**	**Study N**	**Intervention (as named in study)**	**Intervention description**	**Intervention aim**	
**Self-Management**						
**Fatigue self-management - conservative**						
1.Abonie 2020	MS	21	Tailored activity pacing	Tailored pacing based on data from an accelerometer and logbook. Personalised report based on symptom-activity relationship - physical activity, fatigue, physical activity patterns. Develop strategies to develop graded consistent physical activity or increase rest as necessary.	Individual tailoring of intervention should improve the success of activity pacing interventions	
			Control	No intervention	Control.	
2.Askari 2022	MS	26	MSInform	Information about fatigue, fatigue rating and monitoring fatigue. Goal setting for fatigue management. Occupational performance coaching to reflect on meaningful activities affected by MS fatigue. Problem solving.	Improve performance in personally valued activities whilst building skills to address future challenges.	
			Control	Access to the control section of the MSInform website.	Control.	
3.Blikman 2017	MS	86	Energy Conservation Management (TREFAMs)	Aerobic training; Cognitive Behavioural Therapy; Energy Conservation Management	Teaching people to identify and modify their activities to reduce the impact of fatigue on daily life	

			Information only	Nurse consultations providing standardised information about MS fatigue	To control for attention and information about fatigue	
4.Farragher 2022	Kidney	30	Personal Energy Management Programme (PEP)	Energy management strategies e.g. simplifying tasks, pacing, using assistive devices, organising home environments. Structured energy management problem-solving strategies. Assisted application of the principals.	To improve life participation by helping identify energy management strategies to facilitate individual life participation goals.	
			General Disease Self-Management Programme	General information about kidney disease management.	Control.	
5.GarciaJalon 2013	MS	23	Energy Conservation Programme	A group based Energy Conservation Programme, educating people with multiple sclerosis on how to analyse and modify their own activity patterns in order to cope with their fatigue.	To modify unhelpful behaviours to manage fatigue	
			Peer support group	Peer support consisting of education and discussion of common topics for people with multiple sclerosis as recommended by the MS Society, the MS Trust and Action MS	Active control	
6.Ghahari 2010	Neuro	95	Online fatigue self-management programme	Importance of rest, communication, body mechanics, rearranging activity stations, setting priorities and standards, balancing a schedule.	N/R	
			Information only fatigue self-management programme	Information as intervention group, but no activities	N/R	
			Control	Routine care	N/R	

7.Hersche 2019	MS	47	Inpatient Energy Management Education + RAU	Learning how to manage available energy in order to achieve a satisfying and meaningful daily routine. Participants acquire knowledge and understanding about factors that influence energy and the<br>consequences of fatigue on their habits and lifestyle. Identifying and implementing tailored behavior modification + rehabilitation as usual	To ensure that participants learn how to manage available energy in order to achieve a<br>satisfying and meaningful daily routine.	
			Progressive Muscle Relaxation +RAU	A standardized series of relaxation exercises (involving 11 large muscle groups) combined with deep breathing + rehabilitation as usual	To achieve enhanced mental relaxation by reducing muscle tension	
8.Hugos 2010	MS	41	Fatigue Take Control formal group fatigue program	DVD viewing, topic focused discussion, individual goal setting, homework assignments. Identification of treatable or secondary causes of fatigue such as depression, sleep disturbance, deconditioning. Setting goals and priorities, environmental modification, managing mobility, energy effectiveness strategies, importance of exercise.	Fatigue can be reduced by guiding individuals to make the environmental, behavioural and lifestyle changes necessary to manage MS fatigue	
			Wait list	Usual activities	A control	
9.Hugos 2019a	MS	204	Fatigue Take Control group education program	DVD viewing, topic discussion, individual goal setting. Aspects of MS fatigue e.g. depression, sleep disturbance, heat sensitivity, deconditioning. Setting goals and priorities, managing mobility problems, energy conservation strategies.	Based on belief that fatigue can be reduced by guiding individuals to make environmental, behavioural and lifestyle changes necessary to manage fatigue	
			MS Take Control group program	Educational pamphlets and group discussion around: MS and your emotions; solving cognitive	No DVDs or goal setting activities	

				problems; taming stress, food for thought, MS and Nutrition, urinary dysfunction, Vitamins, minerals and herbs in MS.		
10.Kos 2016	MS	31	SMOoTH self management occupational, therapy programme	Strategies to support clients to take control over the performance of activities within the limits of their available energy, raising self-efficacy.	Based on principles of the Energy Conservation/Enveloped Theory	
			Stress management and relaxation	Education about the role of stress in MS, practicing relaxation techniques.	To alleviate stress which may play an important factor in persistence of fatigue	
11.Murphy 2010	OA	32	Tailored activity pacing	Accelerometer data to measure physical activity, symptom log, diary of daily activities. Study specific education module on activity pacing, tailored activity recommendations based on personalised report.	To use tailored activity pacing to address symptoms that interfere with activity engagement	
			General activity pacing	Accelerometer data to measure physical activity, symptom log, diary of daily activities. Study specific education module on activity pacing. No tailored recommendations.	To control for tailoring of activity pacing.	
**Fatigue self-management - active**						
12.Clarke 2012	Stroke	19	Fatigue Management Group	Psychoeducation aimed at alleviating fatigue symptoms. Fatigue diary (tracking fatigue and activities) and homework. Group brainstorming to find solutions to problems identified. Sharing individual experiences and individual assistance.	To evaluate the benefits of educational fatigue management	
			General Stroke Education	Psychoeducation not particularly aimed at alleviating fatigue. Information presented in a didactic format with illustrations from daily life. Sharing individual experiences and individual assistance.	A control	

13.Murphy 2024	SS	173	Resilience- Building Energy Management Program (RENEW)	Focuses on wellness through bolstering self-efficacy, positive experiences, and emotions as opposed to focusing on reducing symptom burden or suffering. Positive activity interventions encourage behavioral activation by inviting patients to engage in pleasant activities. Physical activity, pacing activities, relaxation techniques, practicing adaptive (positive) thoughts, taking care of one's body, healthy diet, and sleep.	Theoretical grounding in self-efficacy theory and positive psychology, which teaches people to more optimally respond to stressors to build resiliency	
			Wait list	Usual routine	Control	
14.Rietberg 2014	MS	48	Multidisciplinary rehabilitation	An individually tailored programme focused on optimising self-management behaviour in daily life activities on the domains of physical fitness, behaviours or cognitions that perpetuate fatigue, and energy conservation. Physical therapy; occupational therapy; social work	To investigate the effects of an individually tailored multidisciplinary outpatient rehabilitation programme on MS fatigue	
			MS Nurse consultation	Nurse consultation to set goals and evaluated in a follow-up session	A mono-disciplinary programme as control	
**General self-management**						
15.Austin 1996	SLE	58	Telephone Counselling	Counselling targeting six behaviours: self-care activities in managing fatigue; patient's communication skills; removing barriers to medical care; medication self-management; symptom monitoring; stress control methods.	To assist patients in decreasing fatigue, physical function and improving psychological function.	
			Symptom monitoring	A review of fatigue; physical function; self-care activities; social activity; support from family; flare ups; joint pain; mood and tension.	To assist patients in decreasing fatigue, physical function and improving psychological function.	

16.Feldthusen 2016	RA	70	Person-centred Physical Therapy	Self-care plan to manage fatigue focused on tailoring health-enhancing physical activity and balancing life activities.	To devise a mutually agreed care plan	
			Control	Usual activities	Control.	
17.Hammond 2008	RA or PsA	218	Modular Behavioural Arthritis Education Programme	Looking after your joints; keeping mobile and managing pain and mood; advice, goal setting and action planning towards recommended frequency targets. Behavioural joint protection programme, health beliefs, personal impact of arthritis, understanding factors affecting symptoms, attitudes, self management methods, motivation to change.	A modular structure is proposed to promote sustained behavioural change	
			Standard information focused education programme	What is arthritis, how it affects the joints and body; drug treatments; managing arthritis. Exercise: 30 minute stretching program, rest, posture pain management. Joint protection, managing fatigue, healthy diet.	A control	
18. Khan 2020	SLE	50	Digital Therapeutic Intervention	Tracking of lifestyle activities (e.g. diet, sleep habits, physical activity, bowel movements); analysis and organisation of data; presentation of data to health coach. Telehealth coaching sessions based on individual data.	To identify and intervene on dietary and other lifestyle factors	
			Usual Care	Usual care as recommended by treating physician	Control.	
**CBT – fatigue**						
19. Artom 2019	IBD	31	Cognitive Behavioural Therapy	IBD-fatigue explained; CBT for IBD-fatigue; activity scheduling; improving your sleep; understanding IBD symptoms; changing your thinking; managing stress; determining a sense of control and coping	Disease-related factors trigger fatigue. The ways in which people respond cognitively, emotionally and behaviourally to their fatigue may then	

				with emotions; social support; preparing for the future. CBT manual.	contribute to the perpetuation or worsening of symptoms. The targeting of cognitions, emotions and behaviour related to fatigue through (CBT) may improve clinical and psychosocial outcomes	
			Information	CCUK 'Fatigue in IBD' Information Sheet to use without therapist help	Control	
20. Bredero 2023	IBD	113	Mindfulness-Based Cognitive Therapy	A structured group intervention. Group meditation, cognitive-behavioural exercises, psycho-education (fatigue symptoms and management, stress management), daily homework. Helping patients to develop more non-judgemental awareness of fatigue experiences, to learn to de-centre from negative feelings and perceptions of fatigue, to become more aware of unhelpful automatic reactions, and to make conscious choices about doing physical activity.	To focus away from unhelpful reactions to fatigue and physical activity	
			Wait List	Usual activities	A control	
21. Ehde 2015	MS	163	Self-management telehealth	Evidenced-based cognitive-behavioural and positive psychology strategies for helping participants self-manage pain,	To help adults with MS effectively manage fatigue, chronic pain, and/or depression.	
			MS education telehealth	Telephone-delivered MS education intervention, educational material on additional topics such as fatigue and nutrition	A rigorous active control	

22. Gay 2023	MS	105	CBT FACETS+	Management of MS-related fatigue, incorporating elements of cognitive-behavioural, energy effectiveness, self-efficacy therories. To help people normalise their experience of fatigue, learn to change the way they think about fatigue to a more adaptive perspective and make more effective use of their energy.	To challenge and modifying dysfunctional beliefs and thoughts related to fatigue that can contribute to its onset, maintenance and amplification.	
			Standard care	Local standard care comprising general advice and information about MS-related fatigue, including its characteristics, contributory factors and ways to reduce its impact. Information booklet and tips for fatigue management.	A control	
23. Hewlett 2011	RA	168	Cognitive Behavioural Therapy	Topic likely to improve fatigue: thoughts, feelings and behaviours related to fatigue were addressed using Socratic questioning and guided discovery to enable patients to work out links themselves. Problem-solving; goal setting; self-monitoring or activity/rest and energy management.	To help patients turn cognitive and behavioural changes into improved well-being	
			Information only	Arthritis Research UK leaflets ‘Fatigue and RA’ and fatigue excerpts from ‘Looking after your joints’. A session covering fatigue symptoms, consequences, causes	Control	
24.Hewlett 2019a	RA	333	Cognitive behavioural approach RAFT	RAFT course uses CBT approaches to address behaviours likely to be related to fatigue and their underpinning thoughts and feelings. Exploratory questioning, goal setting, peer support to enhance self-efficacy, prompting changes in self-management	Enhancing self-efficacy prompts changes in fatigue self-management	
			Usual Care	Arthritis Research UK fatigue self management booklet based on the original RAFT intervention.	A control	

				Group session covering fatigue symptoms, consequences, causes and self-management suggestions.		
25.Jhamb 2023	Kidney	160	Collaborative care	Targeted at 1 or more symptoms (fatigue,pain, and/or depression) based on patients' reported levels of each symptom and preference. Using an individualized and shared decision-making approach, pharmacotherapy, and/or CBT were offered. A stepped approach to treatment intensification allowed for monitoring patient adherence, treatment response, preferences, and outcomes, and modifying the treatment to achieve the best possible outcome for each patient. The CBT strategies were contextualized to address the unique challenges and needs of each patient receiving hemodialysis.	Treatment of symptom clusters may be more effective given that many of the physical and mental symptoms frequently coexist, are highly correlated, can exacerbate each another, and may share similar biologic and psychologic pathogenesis.	
			Health education	ESKD-relevant education on relevant topics - kidney transplantation, heart health, immunizations, diet, travel per patient preference via telemedicine delivered in the dialysis units or at home.	Attention control	
26.Mead 2022	Stroke	76	Cognitive Behavioural Intervention	Focused on the potentially reversible nature of fatigue, teaches (a) overcome fears about physical activity, (b) increase physical activity using diary monitoring and activity scheduling, (c) achieve a balance between activities, rest and sleep and (d) address unhelpful thoughts related to fatigue and low mood if present.	Symptoms, feelings and behaviours are interconnected and that identifying unhelpful thoughts, and challenging them, e.g. through the use of behavioural experiments, can lead to changes.	

			Information only	Patient information leaflet<br>provided by the Stroke Association	Control	
27.Menting 2017	T1D	120	CBT	Dia-Fit CBT. Goal setting; regulation of sleep-wake pattern; formulation of helpful fatigue-related beliefs; activity regulation and graded activity; coping with pain; optimisation of social support and interactions; reducation of diabetes-related distress; step-by-step realisation of goals.	Assumes that disease-specific elements trigger fatigue, which is maintained by cognitive behavioural factors. CBT aims to address these perpetuating factors.	
			Wait list	Care as usual	A control.	
28.Moss-Morris 2012	MS	45	MS Invigor8	Website based on a CBT programme containing modules on MS fatigue; a fatigue diary; rest and activity patterns; improving sleep; understanding MS symptoms; recording thoughts; managing stress; emptions, support and the future.	To test a behavioural approach to MS fatigue with a clear conceptualisation of fatigue.	
			Standard care	Usual activities	A control	
29.Nguyen 2019	Stroke	15	CBT	CBT addressing fatigue and sleep encompassing principles of psychoeducation, behavioural activation, behavioural experiments, cognitive restructuring, problem-solving, relapse prevention, plus suitable exercise guidelines to encourage physical exercise to improve energy, sleep and mood	To investigate the efficacy of individual CBT targeting fatigue and insomnia with exercise to improve energy, sleep and mood	
			Wait list	Treatment as usual	Control.	
30.Okkersen 2018	MD	255	CBT with optional graded exercise	Cognitive behavioural therapy customised to individual participants by selecting from modules including regulating sleep/wake pattern; compensating for reduced patient initiative;	Patient reported HRQoL can be improved by addressing reduced patient initiative,	

				formulating helpful beliefs about fatigue and myotonic dystrophy type 1; optimising social interactions; coping with pain. Optional graded exercise where available.	optimising physical activity, and alleviating fatigue	
			Standard care	Standard care applicable to the patient's home country	Control.	
31.Picariello 2021	Kidney	24	CBT (BReF)	CBT based self-management intervention aimed specifically at fatigue. Targets fatigue thoughts, emotions and behaviours by creating consistent activity and rest routine, graded increase of daily activity, and identifying and managing unhelpful thoughts in relation to fatigue.	To target the perpetuators of fatigue which is likely to lead to improvements	
			Wait list	Usual renal care	Control.	
32.Pottgen 2018	MS	275	Self-guided online fatigue intervention	ELEVIDA programme: based on CBT strategies conveyed through simulated dialogue.	To test a web-based version of CBT for MS fatigue to improve accessibility.	
			Wait list	Usual activities	A control	
33.Thomas 2013	MS	164	FACETS group based fatigue management programme	A conceptual framework integrating elements from cognitive behavioural, social-cognitive, energy effectiveness, self-management and self-efficacy theories.	To normalise fatigue experiences, learn helpful ways of thinking about fatigue and use available energy more efficiently.	
			Usual care	Current local practice alone	A control	
34.van Kessel 2008	MS	72	CBT	Manual based of a cognitive behaviour model of fatigue. Socratic questioning. Individually tailored to focus on aspects that were important to participants. Goal setting according to specific issues; development of behavioural and cognitive strategies.	To challenge and behavioural, cognitive, emotional and external factors that may be contributing to MS fatigue.	

			Relaxation training	Participants taught a range of relaxation techniques including diaphramatic breathing, progressive muscle relaxation, visualisation, cue-controlled relaxation, rapid relaxation.	To control for therapist contact and support	
35.van den Akker 2017	MS		CBT TREFAMS-CBT	Cognitive behavioural therapy protocol with modules on formulating goals; regulating sleep/wake pattern; changing beliefs regarding MS; changing beliefs regarding fatigue; reducing the focus on fatigue; regulation of physical, social and mental activity, addressing the role of the environment; handling pain.	Disease-related factors trigger fatigue in MS, and cognitive, emotional and behavioural factors determine the extent to which fatigue interferes with daily life. CBT aims to address these factors if dysfunctional.	
			Control treatment	Written and oral information about MS fatigue; discussion of personal experiences in coping with fatigue and other fatigue-related issues	Attention control	
36.Zedlitz 2012	Stroke	83	Cognitive Therapy and Graded Activity Training COGRAT	Cognitive treatment emphasising pacing and relaxation to mange fatigue and psychological distress, plus graded activity including walking on a treadmill, strength training, and homework assignments	To test whether adding graded activity to cognitive therapy is effective at alleviating fatigue and fatigue like symptoms in stroke patients	
			Cognitive Therapy only CO	Cognitive treatment emphasising pacing and relaxation to mange fatigue and psychological distress	To test the effectiveness of CO alone	
**Physical Activity**						
**Physical activity promotion**						
37.Bachmair 2022	IRD	367	Cognitive behavioural approach	LIFT CBA - psychological intervention targeting unhelpful beliefs and behaviours and aiming to replace them with more adaptive ones	Aimed to replace unhelpful behaviours with more adaptive ones	

			Personalised exercise programme	LIFT PEP - exercise programme individually tailored and combined with graded exposure behavioural therapy aimed to normalise misperceptions of effort and enhance exercise tolerance	Aimed to normalise misperceptions of effort and enhance exercise tolerance	
			Usual Care	VERSUS arthritis education booklet for fatigue	Control.	
38.Callahan 2014	RA	354	Behavioural Lifestyle Intervention ALED	Instructor-led group discussion<br>session covering topics such as setting goals, enlisting support, and managing time. Group discussions reinforce material in the ALED Workbook.	Behavioral theory–based lifestyle program teaches appropriate cognitive and behavioral skills to identify and overcome barriers to physical activity participation.	
			Wait List	Usual daily activities	Control	
39.Lutz 2017	MS	14	EG-I	Participants were taught neurophysiological essentials in MS disease, (neuro) physiological effects of sports, and physical exercises in general and specific for MS, MS-specific recommendations of exercise training, training principles, and the importance of resting periods. In order to guarantee a comprehensive treatment, various types of exercise training (cardiorespiratory, strength, coordination/reflex-based, and flexibility) were offered based on individual performance abilities.	Evaluate the effects of the revised six-week ePEP on self-regulated and long-term exercise behaviour	
			EG-W	Instructed not to change their daily routines	Control	
40.Turner 2016	MS	64	Physical Activity Counseling	Telephone-Administered Physical Activity Counseling. Telephone counseling and home-based telehealth monitoring.<br>Education as control arm plus mailed graphic feedback, 6 telephone counseling sessions using	MI encourages behavior change by contrasting current behavior, such as physical inactivity, with	

				principles of motivational interviewing, and telehealth home monitoring to track progress on physical activity goals.	desired goals and values, such as physical fitness, good self-care, and quality of life, in a manner that is empathetic, evocative, collaborative and intended to promote self-efficacy.	
			Physical activity education	Self-directed physical activity education. Advice to increase physical activity and a DVD with examples of in-home<br>exercises for multiple physical ability levels.	Control	
**Exercise – supervised**						
41.Dalgas 2010	MS	38	Progressive resistance training [PRT]	Intervention to improve muscular strength, functional capacity, and reduce fatigue	N/R	
			Usual care	Continued previous daily activity level	Control	
42.Diaz 2023	PsO	118	Aerobic training program	Aerobic training program on a conventional motorized treadmill, consisting of a<br>warm-up, treadmill exercise at a<br>work intensity of 50–65% of peak heart rate (increasing by 5% every four weeks) measured during a previous maximal treadmill test, cool-down.	Sedentary lifestyle may influence the natural course of psoriasis natural and the existence of comorbidities	
			Control	N/R	Control	

43.Englund 2022	MS	140	High-Intensity Resistance Training (HIRT) - Group A	Resistance training	To compare the effects of high-intensity resistance training (HIRT) on self-reported fatigue	
			High-Intensity Resistance Training (HIRT) - Group B	Resistance training	As above with fewer sessions	
			Control	No intervention	Control.	
44.Escudero-Uribe 2017	MS	55	Whole Body Vibration	Exercises (amplitude 1⁄4 3 mm, average frequency 1⁄4 4 Hze1 Hz/sec) using a Zeptor Med System. Vibrations transmitted to the body stimulate the participants' muscle spindles, generating subconscious muscle contractions.		
			Balance Trainer System	Dynamic balance with the BT system, a mechanical device that provides a fall-safe balancing environment. The BT software (Balance-Soft version 01.04.02) includes different types of exercises and games that force a person's centre of gravity to be shifted in different directions, thereby activating their leg, pelvis, and trunk muscles.		
			Wait List	Usual activities	Control	
45.Heine 2017	MS	89	Aerobic training	Aerobic interval training	To test the effectiveness of aerobic training on MS-related fatigue	
			Usual care	Consultations with an MS nurse including reliable information on MS-related fatigue and guidance from the experienced MS nurse	Education control	

46.Feys 2019	MS	42	Group exercise	Remotely supervised community-located “start-to-run” program	To test the effectiveness of physical activity on Fatigue	
			Waiting List Control Group (WLC)	No intervention	Control.	
47.Gervasoni 2014	MS	22	Arm cycling and task-oriented exercises	Aerobic training and task-oriented rehabilitation programme	Aerobic activity will improve fatigue and fatiguability in people with MS	
			Wait list	Crossed over to intervention group after 8 weeks	Control	
48.Kratz 2020	MS	20	Exercise therapy	Weekly educational modules and resources, and equipment for a range of exercises (yoga mat, 1 set of 5 resistance bands attached to a carabiner, 1 leg strap with carabiner, a door anchor for securing resistance bands); weekly exercise logs, and a wrist-worn pedometer/HR monitor.	To test the benefits of exercise in improving fatigue	
			Telephone exercise intervention	A weekly phone call	Control	
49.Kucharski 2019	RA	74	Aerobic and resistance exercise	Moderate-to-high intensity, aerobic and resistance exercise in the gym with person-centred guidance	Moderate to high intensity exercise will improve fatigue	
			Home exercise	Performed light home-based exercise for mobility, lower body strength and balance, but no gym-based exercise	Control	
50.Langeskov-Christensen 2022	MS	86	High intensity aerobic exercise	High-intensity progressive aerobic exercise (PAE).	High-intensity aerobic exercise leads to cardioprotective benefits and may be superior in ameliorating secondary	

					MS fatigue through a higher increase in fitness and motor efficiency	
			Wait List	Habitual lifestyle (including ongoing physiotherapy treatment).	Control	
51.Louie 2022	MS	33	Exercise and education programme	Program incorporating behaviour change education, exercise and community integration		
			Usual care	Usual daily activities	Control.	
52.McCullagh 2008	MS	30	Exercise	3 months' exercise programme	To determine if exercise benefits patients with multiple sclerosis	
			Usual care	Usual daily activities	Control	
53.Ortiz-Rubio 2018	PD	46	Resistance training program	Training structure included 5 to 10min warm up, core activities and 5-min cool-down, lower-extremity exercises focused on strengthening all major muscle groups of lower limbs with the aid of elastic bands in a seated position. Exercises at lower loads (elastic bands resistance of 1.5 kg), then exercises performed in 1–3 sets with 10–15 repetitions in each and using a band with a resistance of 2.7 kg. The rate of progression was modified and adapted according to specific physical limitations.	Examine the effects of a twice-a-week resistance training program using elastic bands during 8 weeks on dynamic balance and fatigue in patients with PD.	
			Low intensity exercise	Weak low-intensity exercise program in order to introduce similar social interaction, enjoyment and physical activity levels. This program included breathing, stretching and relaxation activities, with the activities performed in a seated position.	Control	

54.Pozehl 2008	HF	21	Exercise programme	Four different aerobic modalities (treadmills, stationary bikes, rowers, and arm ergometers) were utilized according to individual tolerance during the aerobic phase. Intensity of this phase was set at 60–85% maximum VO2 obtained from the baseline cardiopulmonary exercise test and a rating of 12–14 of perceived exertion (RPE) on the Borg scale. The strength/resistance training consisted of subjects performing light upper-body exercises (military press, biceps curl, and lateral deltoid raises) and lower-body exercises (knee extension, side hip raise, and hip extension) with 1–10 lb hand and ankle weights. Wall push-ups, abdominal curl-ups, and/or pelvic tilts were also included in the 20-minute strength/ resistance training.	Aerobic exercise will improve fatigue in people with heart failure	
			Usual care	Usual daily activities	Control	
**Exercise – unsupervised**						
			Usual	Instructed not to perform any physical activity besides their usual daily life requirements. Every 14 days they also received a phone call.	Control	
55.Durcan 2014	RA	80	Home-based exercise	Specific exercises were prescribed to target the individual deficiencies identified. Cardiovascular Exercise: 5 days of moderate intensity cardiovascular exercise, based on a walking program. Resistance Training: Each major muscle group to be trained 2–3 days per week 40–50% of 1 RM. In addition, functional exercises were prescribed according to deficiency identified in HAQ. Flexibility and Neuromotor Conditioning: A daily stretching regimen was devised for each	Evaluate the effect of an exercise program on self-reported sleep quality and fatigue in RA.	

				patient. Timed 1 leg stands were prescribed for neuromotor health. These were advised 2–3 days per week.		
56.Geddes 2009	MS	12	Exercise programme	An individualised home walking program. Participants adjusted their walking speed to stay within their prescribed HR range using a home Heart Rate Monitor. The exercise group subjects were instructed to walk 3 times per week for 12 weeks. For the first 2 weeks, the subjects walked 5 minutes below the lower limits of their THR range, followed by 15 minutes of walking within their THR range, and then a 5-minute cool down below their THR range. During weeks 3 through 12, training time increased in the THR range to 20 to 30 minutes. Weekly exercise log including RPE values and received biweekly telephone calls to monitor their exercise compliance.	To investigate the effects of a convenient 12-week home exercise walking program on cardiovascular parameters, energy expenditure, and fatigue perception in individuals with mild to moderate MS.	
			No regular exercise	The control group was asked to refrain from any regular exercise during the period.	Control	
57.Katz 2018	RA	96	Pedometer + step log	Educational booklet and discussion, plus a pedometer and a diary to record daily step counts from the pedometer. The step diary with prewritten dates and space to record each day's steps and notes about other activities, problems with the activity monitor, injuries, or other relevant issues.	To test the comparative effectiveness of exercise with the additional of step targets.	
			Pedometer + step log + step targets	Educational booklet and discussion, pedometer and step diary, and individualized daily step targets. Step targets were based on the week of activity monitoring between the baseline and randomization visits, and were calculated to	As above.	

				increase participants' average daily step counts by 10% for every 2 weeks of the intervention period.		
			Education only	Received an educational brochure (Be Active Your Way: A Guide for Adults). Guided discussion of simple ways to increase physical activity in daily life based on the booklet. The brochure was available in English and Spanish.	Control	
58.Maurer 2018	178	MS	Exercise	The individual exercise schedules comprised strengthening exercises twice a week and endurance training once a week. Balance or core stability exercise could be added. The personal exercise schedule and the comprised exercises were explained in a two-day on-site introductory group session at the beginning of the intervention period. Participants documented each exercise session via a web-based application (duration, type of exercises, number of repetitions, and sets, perceived exertion) and used an electronic exercise diary that could be supervised by the exercise therapist.	Evaluated the effect of an exercise intervention on fatigue in relapsing–remitting MS patients receiving fingolimod.	
			Wait List	No intervention	Control	
59.Tench 2003	SLE	93	Aerobic exercise therapy	Asked to exercise at home at least three times a week for between 30 and 50 min for a period of 12 weeks at a heart rate corresponding to 60% of peak oxygen consumption. The main exercise was walking but patients were encouraged to take other forms of exercise, such as cycling and swimming, and were seen every 2 weeks for a supervised exercise session.	To compare aerobic exercise therapy with relaxation therapy	

			Relaxation therapy	Asked to listen to a 30-min relaxation audiotape a minimum of three times a week in a darkened, warm and quiet room and were seen every 2 weeks for a supervised relaxation session.	To compare aerobic exercise therapy with relaxation therapy	
			No intervention	Asked to continue with their normal daily activity pattern and specifically asked to avoid doing any extra physical activities. They were reviewed at follow-up but not seen at other times.	Control	
**Active recreational**						
**Rehabilitation**						
60.DeGiglio 2015	MS	35	Cognitive rehabilitation with commercial video game	Training in games of memory, attention and visuospatial processing, and calculations	N/R	
			Wait list	Wait list control	Control.	
**Mindbody**						
61.Callahan 2016	RA	343	Tai Chi	12 tai chi movements	Reduce arthritis symptoms	
			Control	Usual activities	Control.	
62.Fleming 2019	MS	17	Home-based pilates	Pilates following a DVD	Effect of pilates on anxiety, depression and fatigue in people with MS	
			Supervised pilates	Certified pilates instructor supervises pilates exercises	Effect of pilates on anxiety, depression and fatigue in people with MS	
			Wait list control	Maintain pre-trial activity level	Control.	

63.Fleming 2021	MS	80	Pilates	Home-based pilates guided by DVD	To improve anxiety, depression and fatigue through pilates	
			Wait list control	Pre-intervention physical activity levels and contacted by email or telephone to ensure completion of biweekly outcome assessments	Control.	
64.Walter 2019	PD	27	Yoga	Progressive yoga for PD, focused on balance, strength and mobility. Meditation, physical postures, breathwork.	Non-motor symptoms e.g. pervasive fatigue can lead to decreased HRQoL. Physical activity can alleviate non-motor symptoms.	
			Wait List	Usual care	Control	
65.Sgoifo 2017	MS	48	Integrated Imaginative Distention Therapy	A selection of Jacobson relaxation exercises with breath awareness, motor imaging, body imaginative scan, imaginative experience. After the practice, the participants were invited to a group discussion, managed by the psychotherapist. Participants were invited to repeat the IID steps at home.	Joins interventions previously proven effective on MS fatigue: relaxation, self-awareness, and psychotherapy	
			Wait List	Usual activities	Control	
**Mindfulness**						
66.Goren 2022	CD	116	COBMINDEX	COBMINDEX (Cognitive Behavioural and Mindfulness-based stress reduction with Daily Exercise) is a psychological intervention including techniques such as breathing awareness, body scanning, muscle relaxation, and mindfulness	To improve the quality of life by reducing psychological distress and fatigue in patients with Crohn's Disease	
			Wait list control	No form of psychological instruction during the study period	Control.	

67.Grossman 2010	MS	150	Mindfulness-Based Intervention (MBI)	Specific exercises and topics within the context of mindfulness training, i.e., practices during lying, sitting, and dynamic yoga postures, as well as during everyday life, e.g., stressful situations and social interactions. Mindfulness exercises included observation of sensory, affective, and cognitive domains of perceptible experience.	Proposes that non-judgmental awareness of moment-to-moment experience (i.e., mindfulness) may positively affect accuracy of perception, acceptance of intractable health-related changes, realistic sense of control, and appreciation of available life experiences.	
			Usual care (UC)	Received regular, currently optimal medical care during the duration of the study	Control.	
68.Torkhani 2021	MS	35	Mindfulness-Based Intervention (MBI)	Daily mindfulness training associated with a Physical Activity program, delivered via internet	To compare with Implementation Intention in reducing Multiple Sclerosis symptoms	
			Implementation Intention	If-then plan associated with a Physical Activity program, delivered via internet	To with mindfulness in reducing Multiple Sclerosis symptoms	
			Control group	Not guided to develop if-then plans and they did not receive any mindfulness training, however, they received the same PA program	Control	

**Stimulation Interventions**						
**Study**	**Population**	**Study N**	**Intervention (as named in study)**	**Intervention description**	**Intervention aim**	
**Vagal stimulation**						
69.Aranow 2021	SLE	18	VNS	Vagus Nerve Stimulation	The inflammatory	

					reflex is a physiological mechanism that attenuates the innate inflammatory response. Stimulation of the vagus nerve results in the reduction of inflammatory mediators	
			Sham Stimulation	Sham VNS	Control	
70.Tarn 2023	Sjögren’s Syndrome	40	VNS	VAGUS NERVE STIMULATION	Reduce fatigue & pain	
			Sham	Sham VNS	Control	
**Trans Cranial stimulation**						
71.Cancelli 2018	MS	10	tDCS	Cross over transcranial direct current stimulation (tDCS)	Reduce fatigue symptoms	
			Sham			
72.Charvet 2018	MS	42	(Study 2) tDCS	To evaluate whether tDCS can reduce fatigue in individuals with MS.	Reduce fatigue	
			Sham		Control	
73.Salemi 2019	MS	17	tRNS	Transcranial direct current stimulation	Stimulate motor cortex to improve fatigue	
			Sham tRNS	Sham	Control	
74.Tecchio 2015	MS	21	Transcranial direct current stimulation whole body	Cross-over bilateral whole body S1 anodal tDCS/ hand treatment/ sham	Reduce fatigue and assess whether it also induces changes in the excitability of	

					sensorimotor cortical areas	
			tDCS hand & sham			
**External stimulation**						
75.Granja-Dominguez 2022	MS	44	Pulsed electromagnetic field therapy	PEMF	Effects of PEMF therapy on the self-reported level of fatigue in people with RRMS.	
			Placebo	Sham	Control	
76.Mostert 2005	MS	24	Pulsed Magnetic field therapy	Pulsed magnetic therapy	Reduce fatigue	
			Sham	Sham pulsed magnetic therapy	Control	
77.Piatkowski 2009	MS	37	Bio-Electro-Magnetic-<br>Energy-Regulation (BEMER)	8 minutes twice every day at home. In the treatment group (verum), the BEMER mattress was activated BEMER pulsed electromagnetic fields	To evaluate the long-term effects of BEMER therapy in MS patients with significant fatigue	
			Sham	As above but no magnetic field was generated although there was the typical<br>BEMER sound.		
78.Voggenberger 2022	MS	26	Bright light therapy (BLT)	Light box positioned at a height aligned with eyes at a distance of 30 cm, at which 10 000 lux were achieved. Participants were instructed to keep their eyes open during the whole 30 min of light therapy.	Improve fatigue	
			Dim red light therapy (DRL)	As above The light boxes were identical in both groups, with the only difference	Placebo	

				that we installed a filter that dimmed the light to 200 lux and tinted it red		
**Aromatherapy**						
79.Hawkins 2019	Hypothyroidism	54	Peppermint aromatherapy	Essential oil blend was primarily composed of peppermint (Mentha x piperita) essential oil. In addition to the peppermint essential oil, small amounts of black pepper (Piper nigrum) essential oil, clove bud (Eugenia caryophyllus) essential oil, white grapefruit (Citrus x paradisii) essential oil, and bergamot (Citrus Aran- tium bergamia)	Peppermint essential oil is traditionally used to reduce fatigue by aromatherapists	
			Avocado vegetable oil	A bottle of avocado vegetable oil with disposable paper inhaler sticks. This oil was selected due to its light green hue which resembles the color of the essential oil blend used for the intervention group, and for its lack of an aroma.	Placebo	
**Acupuncture/ acupressure**						
80.Horta 2020	IBD	46	EAc	Electroacupuncture	Evaluate effect on fatigue	
			ShEAc	Sham electroacupuncture	Placebo effect	
			Wait List	Waiting list	Control	
81.Kluger 2016	PD	94	Acupuncture	Acupuncture needles inserted at 10 points	Improve fatigue	
			Sham	Toothpicks used on sham points	Control	

**RIC**						
82.Moyle 2023	Stroke	24	Remote Ischaemic Conditioning RICFAST	Inflating a blood pressure cuff around the participant's upper arm to 200 mmHg for 5 min and then deflating for 5 min.	RIC can preserve mitochondrial function, improve tissue perfusion and may mitigate PSF.	
			Sham RIC	As above, inflation pressure 20 mmHg	Control	

**Nutritional Interventions**						
**Study**	**Population**	**Study N**	**Intervention (as named in study)**	**Intervention description**	**Intervention aim**	
**Fish oil**						
83.Arriens 2015	SLE	50	Fish oil	Fish oil (6 capsules/day equaling 2.25 g EPA and 2.25 g DHA)	Reduced omega-3 fatty acids, which are powerful anti-oxidants observed in SLE. This deficiency may be causally related to oxidative stress, inflammation, disease activity, and fatigue in SLE.	
			Placebo	Visually identical capsules	Control	
**Thiamine HD**						
84.Bager 2021	IBD	40	Thiamine	High-dose oral thiamine for 4 weeks (containing 300 mg thiamine hydrochloride), 4 weeks of	Most interventions are of behavioural or	

				washout, 4 weeks of oral placebo<br>Daily dose depended on gender and body weight (BW) according to the following scheme:<br>Females: BW < 60 kg: 600 mg (2 tablets), BW 60-70 kg: 900 mg (3 tablets), BW 71-80 kg: 1200 mg (4 tablets), and BW > 80 kg: 1500 mg (5 tablets)<br>Males: BW < 60 kg: 900 mg (3 tablets), BW 60-70 kg: 1200 mg (4 tablets), BW 71-80 kg: 1500 mg (5 tablets), and BW > 80 kg: 1800 mg (6 tablets)	psychological character not on pharmacological treatments. Therefore, high-dose oral thiamine versus placebo for chronic fatigue in patients with quiescent inflammatory bowel disease	
			Placebo	Oral placebo for 4 weeks, 4 weeks of washout, 4 weeks of high-dose oral thiamine (containing 300 mg thiamine hydrochloride).<br>Daily dose depended on gender and body weight (BW) according to the following scheme:<br>Females: BW < 60 kg: 600 mg (2 tablets), BW 60-70 kg: 900 mg (3 tablets), BW 71-80 kg: 1200 mg (4 tablets), and BW > 80 kg: 1500 mg (5 tablets),<br>Males: BW < 60 kg: 900 mg (3 tablets), BW 60-70 kg: 1200 mg (4 tablets), BW 71-80 kg: 1500 mg (5 tablets), and BW > 80 kg: 1800 mg (6 tablets).	To test high-dose oral thiamine as an alternative to behavioural or pharmacological interventions	
**5-HTP**						
85.Truyens 2022	IBD	166	5-HTP	Oral 5-HTP (100 mg) twice daily for 8 weeks (then crossover - no washing out period given placebo twice daily for 8 weeks)	Effect of 5-Hydroxytryptophan on Fatigue in Quiescent Inflammatory Bowel Disease	

			Placebo	Placebo twice daily for 8 weeks (then crossover - no washing out period then given oral 5-HTP (100 mg) twice daily for 8 weeks)	Effect of 5-Hydroxytryptophan on Fatigue in Quiescent Inflammatory Bowel Disease	
**Flavenoid - cocoa**						
86.Coe 2019	MS	40	High-flavanol cocoa drink	Consume one sachet with heated rice milk (after an overnight fast) at the same time each morning. Wait 30 minutes before consuming any other food or beverage and/or take their medication. Usual diet followed for the rest of the day. High flavanoid content.	Investigate whether flavanoid rich cocoa will improve fatigue in people with RRMS	
			Low-flavanol cocoa drink	As above but with low flavanoid content.	Control	
**Diet**						
87.Chase 2023	MS	39	Low fat diet	Nutrition counselling + low fat diet. A low-fat diet (fat total daily calories⩽20%) with saturated fat<7% of daily caloric intake and the rest of caloric breakdown consisting of 20% protein and 60% carbohydrate (primarily complex).	There is a possible association between weight loss and fatigue.	
			Wait List	Usual diet	Control	
			Usual diet	Consume their pre-study vitamins, supplements, and/or medications.	Control	
**Plant-based**						
88.Johnson 2006	MS	21	Ginko	Four 60mg tablets of EGb-761 (ginko extract) per day	Will ginko extract improve functional performance	

			Placebo	Placebo	Control	

Supplemental Results 6
Intervention delivery characteristics for studies in the NMA
6.1 Behavioural Interventions

Study/ Population	Individual/ group	Setting	Number of sessions	Duration of sessions	Total duration of intervention	Intervention provider
**Self-Management**						
**Fatigue self-management - conservative**						
Abonie 2020	I	Home monitoring	1	30 minutes intervention session after 7 days home monitoring	4 weeks	N/R
	N/A	N/A	N/A	N/A	4 weeks	N/A
Askari 2022	I	Web-based plus telephone sessions	6 telephone calls	30-60 minutes	12 weeks	Web + registered occupational therapist
	I	Web-based	N/A	N/A	12 weeks	Web only
Blikman 2017	I	Outpatient rehabilitation department	12	45 minutes	4 months	Occupational therapist
	I	Outpatient rehabilitation department	3	45 minutes	4 months	Nurse
Farragher 2022	I	Web-based + one-to-one training	3 web modules + 4-6 face-to-face sessions	Web modules: 20-30 minutes; one-to-one sessions: 30 minutes	7-9 weeks	Occupational therapist

	I	Web-based one-to-one sessions	6 to 8 sessions	N/R	7-9 weeks	Trained study coordinator
GarciaJalon 2013	G	Face to face	5	2 hours	5 weeks	Therapist
	G	Face to face	5	2 hours	5 weeks	Therapist
Ghahari 2010	G	Online	6	2-3 hours	7 weeks	Occupational therapist
	I	Online	6	N/R	7 weeks	Group facilitator for technical queries only
	I	N/A	N/A	N/A	7 weeks	N/A
Hersche 2019	G	Rehab centre	6 x 1 hour + 1 x 0.5 hours	1 hour or 0.5 hours	3 weeks	Occupational therapist
	G	Rehab centre	6	1 hour	3 weeks	Physical therapist
Hugos 2010	G	N/R	6	2 hours	6 weeks	MS healthcare professionals via DVD
	N/A	N/A	N/A	N/A	6 weeks	N/A
Hugos 2019a/2017	G	N/R	6	2 hours	6 weeks	MS professionals
	G	N/R	6	2 hours	6 weeks	MS professionals
Kos 2016	I	Face to face	3	60-90 minutes	3 weeks	Occupational therapist
	I	Face to face	3	60-90 minutes	3 weeks	Occupational therapist
Murphy 2010	I	Home and face to face	2	1.5 hours	4 weeks	Occupational therapist
	I	Home and face to face	2	1.5 hours	4 weeks	Occupational therapist
**Fatigue self-management - active**						
Clarke 2012	G	Face to face	6	60 minutes	6 weeks	Researcher
	G	Face to face	6	60 minutes	6 weeks	Researcher

Murphy 2024	G	Online	9	15-30 minutes	12 weeks	Health coaches
	N/A	N/A	N/A	N/A	12 weeks	N/A
Rietberg 2014	I	Outpatient	PT 24; minimum 2 session for other treatments	PT 45 minutes; 1 hour other treatments	12 weeks	Multi-diciplinary
	I	Outpatient	2	1 hour	12 weeks	Nurse
**General self-management**						
Austin 1996	I	Telephone	N/A	N/A	6 months	Certified reality therapy counselor
	I	Telephone	N/A	N/A	6 months	Trained staff member
Feldthusen 2016	I	Face to face	According to preferences	According to preferences	12 weeks	Physical therapist
	I	N/A	N/A	N/A	12 weeks	N/A
Hammond 2008	G	District or community hospitals	8	2.5 hours	3-4 weeks	Experienced therapists
	G	District or community hospitals	5	2 hours	3-4 weeks	Experienced therapists
Khan 2020	I	Telehealth	16	20-30 minutes	16 weeks	Health coach
	I	N/A	N/A	N/A	16 weeks	Usual physician
**CBT – fatigue**						
Artom 2019	I	Telephone	1 x 1 hour and 7 x 30 minutes	1 x 1 hour and 7 x 30 minutes	8 weeks	Therapist
	I	Home	N/A	N/A	8 weeks	N/A

Bredero 2023	G	Outpatient	8	2.5 hours	8 weeks	3 licensed and experienced mindfulness trainers.
	N/A	N/A	N/A	N/A	8 weeks	N/A
Ehde 2015	I	Telehealth	8 + 2 follow-up calls	45-60 minutes	8 weeks + follow up call at 4 and 8 weeks post treatment	Study therapist
	I	Telehealth	8 + 2 follow-up calls	45-60 minutes	8 weeks + follow up call at 4 and 8 weeks post treatment	Study therapist
Gay 2023	G	Face to face	6 + 4 booster sessions	90 minutes	6 weeks + booster sessions	Occupational therapists, physiotherapists, MS nurses
	I	Face to face	N/A	N/A	6 weeks	Usual clinician
Hewlett 2011	G	Face to face	6	2 hours	6 weeks	Clinical psychologist + occupational therapist
	G	Face to face	1	1 hour	6 weeks	Rheumatology nurse
Hewlett 2019a	G	Face to face	7	2 hours for first 6 weeks, 1 hour consolidation session	6 weeks	Nurses, occupational therapists
	N/A	N/A	N/A	N/A	6 weeks	N/A
Jhamb 2023	I	Telemedicine at home	12	45-60 minutes	12 weeks	Therapist with counselling qualification
	G	Face to face	6	20-30 minutes	12 weeks	Research coordinator

Mead 2022	I	Telephone	6	1 hour	12 weeks	Stroke nurses + physiotherapist + psychologist
	I	Home	N/A	N/A	12 weeks	N/A
Menting 2017	I	Web-based with face to face	5 to 8	50 minutes	5 months	Therapist
	N/A	N/A	N/A	N/A	5 months	N/A
Moss-Morris 2012	I	Web-based	8	25 to 50 minutes	8 weeks	Assistant psychologist
	N/A	Standard care	N/A	N/A	8 weeks	N/A
Nguyen 2019	I	Face to face with homework	8	N/R	2 months	Licensed psychologists and exercise physiologist
	N/A	N/A	N/A	N/A	2 months	N/A
Okkersen 2018	I	Face to face	10 to 14	N/R	10 months	Therapists experienced in CBT
	N/A	N/A	N/A	N/A	10 months	N/A
Picariello 2021	I	Face to face and telephone	3 to 5	2 session of 1 hour + 1 to 3 sessions of 30 minutes	3 months	Researcher with background in health psychology
	N/A	N/A	N/A	N/A	3 months	N/A
Pottgen 2018	I	Web-based	Average access 14.5 times	N/R	12 weeks	Web-site
	N/A	N/A	N/A	N/A	12 weeks	N/A
Thomas 2013	G	Face to face	6	90 minutes	6 weeks	Health professionals

	N/A	N/A	N/A	N/A	6 weeks	N/A
van Kessel 2008	I	Face to face or telephone	8	50 minutes	8 weeks	Therapist
	I	Face to face or telephone	8	50 minutes	8 weeks	Therapist
van den Akker 2017	I	Face to face	12	N/R	16 weeks	MS nurse
	I	Face to face	3	45 minutes	16 weeks	MS nurse
	N/A	N/A	N/A	N/A	16 weeks	N/A
Zedlitz 2012	G	Face to face	24	2 hours	12 weeks	Neuropsychologists, physiotherapists
	G	Face to face	12	2 hours	12 weeks	Neuropsychologists
**Physical Activity**						
**Physical activity promotion**						
Bachmair 2022	I	Telephone delivery	7	45 minutes	14 weeks + booster at 22 weeks	Therapist
	I	Telephone delivery	7	45 minutes	14 weeks + booster at 22 weeks	Therapist
	N/A	N/A	N/A	N/A	14 weeks	N/A
Callahan 2014	G	Face to face	20	1 hour	20 weeks	ALED instructors
	I	N/A	N/A	N/A	20 weeks	N/A
Lutz 2017	I	Home-based	N/R	N/R	12 weeks	N/R
	I	Home-based	N/R	N/R	12 weeks	N/R
Turner 2016	I	Telephone delivery	6	30 to 60 minutes	6 months (3 months counseling + 3 months telehealth monitoring)	Study therapist

	I	Home based	N/A	N/A	6 months	N/A
**Exercise – supervised**						
Dalgas 2010	G	Training facility (gym)	24 sessions	60-75 minutes	2 sessions per week for 12 weeks	Principal investigator
	N/A	N/A	N/A	N/A	12 weeks	N/R
Diaz 2023	I	N/R	48	1 hour	16 weeks	N/R
	N/R	N/R	N/R	N/R	16 weeks	N/R
Englund 2022	G	Karolinska University Hospital, Stockholm, Sweden	24 sessions	60 minutes	2 sessions per week for 12 weeks	Physiotherapist
	G	Karolinska University Hospital, Stockholm, Sweden	12 sessions	60 minutes	1 sessions per week for 12 weeks	Physiotherapist
	N/A	N/A	N/A	N/A	N/A	N/A
Escudero-Uribe 2017	G	Face to face	24	60 to 100 minutes	12 weeks	Neurologic physical therapist.
	G	Face to face	24	60 to 100 minutes	12 weeks	Neurologic physical therapist.
	N/A	N/A	N/A	N/A	12 weeks	N/A
Heine 2017	I	Outpatient clinic for supervised sessions, home-based for the rest	48 sessions (12 supervised, 36 home-based)	30 minutes	16 weeks	Physiotherapists
	I	Outpatient clinic	3 sessions	45 minutes	16 weeks	MS nurse
Feys 2019	G	Running track at KULeuven	36 sessions	N/R	3 sessions per week for 12 weeks	Research assistant

	N/A	N/A	N/A	N/A	N/A	N/A
Gervasoni 2014	I	Hospital-based rehabilitation setting	20 sessions	60 minutes (30 minutes of arm cycling, 30 minutes of task-oriented exercises)	16 weeks (8-week active period and an 8-week resting period)	Physical therapists
	N/A	N/A	N/A	N/A	16 weeks	N/A
Kratz 2020	I	Home-based + physical therapist	8 sessions	30 mins (endurance), 30 mins (strength)	8 weeks	N/R
	I	Home-based + physical therapist	8 sessions	N/R	8 weeks	N/R
Kucharski 2019	G	Gym-based exercise and home-based exercise	60 sessions	27 minutes	3 sessions per week for 20 weeks	Physiotherapists
	I	Home-based	N/R	N/R	20 weeks	N/A
Langeskov-Christensen 2022	G	N/R	48 sessions	30 to 60 minutes	2 sessions per week for 24 weeks	N/R
	N/A	N/A	N/A	N/A	24 weeks	N/A
Louie 2022	G	Outpatient rehabilitation facility	20 sessions (14 exercise and 6 education)	60 minutes	Twice weekly exercise and once weekly education sessions for 12 weeks	Physiotherapist and an exercise physiologist
	N/A	N/A	N/A	N/A	12 weeks	N/A
McCullagh 2008	G	At home and also attended exercise classes	36 sessions	50 minutes	Twice-weekly supervised exercise	Physiotherapists

		held in a hospital physiotherapy gym			sessions for 12 weeks, and one home exercise session per week.	
	N/A	N/A	N/A	N/A	12 weeks	N/A
Ortiz-Rubio 2018	I	N/R	16 sessions	60 mins	8 weeks	N/R
	I	N/R	16 sessions	60 mins	8 weeks	N/R
Pozehl 2008	I	Standard cardiac rehabilitation setting	72 sessions	60 mins	24 weeks	N/R
	I	Standard cardiac rehabilitation setting	N/R	N/R	24 weeks	N/R
**Exercise – unsupervised**						
Durcan 2014	I	Home-based	N/R	N/R	12 weeks	N/R
	I	N/A	N/A	N/A	12 weeks	N/A
Geddes 2009	I	Home-based	36 sessions	30 mins	12 weeks	N/R
	I	N/A	N/A	N/A	12 weeks	N/A
Katz 2018	I	Home-based	N/R	Daily	21 weeks	N/R
	I	Home-based	N/R	Daily	21 weeks	N/R
	I	N/A	N/A	N/A	21 weeks	N/A
Maurer 2018	I	Home-based	N/R	N/R	12 months	N/R
	I	N/A	N/A	N/A	12 months	N/A
Tench 2003	I	Home-based	3 sessions/week	30-50 mins	12 weeks	N/R
	I	Home-based	3 sessions/week	30 mins	12 weeks	N/R
	I	N/A	N/A	N/A	12 weeks	N/A

**Active recreational**						
**Rehabilitation**						
DeGiglio 2015	I	Home	40	30 minutes	8 weeks	Psychologist
	N/A	N/A	N/A	N/A	8 weeks	N/A
**Mindbody**						
Callahan 2016	G	20 community locations in North Carolina and New Jersey	16 sessions	60 minutes	2 sessions per week for 8 weeks	Instructors trained by AF master tai chi trainers
	N/A	N/A	N/A	N/A	N/A	N/A
Fleming 2019	I	Home-based	16 sessions	60 minutes	2 sessions per week for 8 weeks	DVD instructions and weekly telephone call
	G	University of Limerick	16 sessions	60 minutes	2 sessions per week for 8 weeks	Pilates instructor
	N/A	N/A	N/A	N/A	N/A	N/A
Fleming 2021	I	Home-based	16 sessions	60 minutes	2 sessions per week for 8 weeks	Pilates instructor
	N/A	N/A	N/A	N/A	N/A	N/A
Walter 2019	G	Face to face	16 sessions	60 minutes	8 weeks	Yoga therapist
	N/A	N/A	N/A	N/A	8 weeks	N/A
Sgoifo 2017	G	Healthcare facility	8 sessions	60 minutes	Once a week for 2 months	Skilled psychotherapist
	N/A	N/A	N/A	N/A	N/A	N/A
**Mindfulness**						

Goren 2022	I	Online video conferences	7 sessions	60 minutes	3 months	Clinical social workers who underwent special training in cognitive-behavioral and mindfulness-based stress reduction
	N/A	N/A	N/A	N/A	N/A	N/A
Grossman 2010	G	In-person sessions at a clinic	9 sessions	2.5 hours per session with one 7 hour session	8 weekly sessions, plus 1 full-day session	Certified mindfulness teachers with at least 9 years of experience
	N/A	N/A	N/A	N/A	N/A	N/A
Torkhani 2021	I	Remote (TailorBuilder tool)	48 sessions	10 minutes	8 weeks	Pre-recorded sessions
	I	Remote (TailorBuilder tool)	8 sessions	Variable	8 weeks	Plans approved by trainer; weekly telephone call follow up
	N/A	N/A	N/A	N/A	N/A	N/A

6.2 Stimulation Interventions

**Study/ Population**	**Individual/ group**	**Setting**	**Number of sessions**	**Duration of sessions**	**Total duration of intervention**	**Intervention provider**
**Vagal stimulation**						
Aranow 2021	I	Feinstein Institutes for Medical Research	4	5 minutes	4 days	N/R

	I	Feinstein Institutes for Medical Research	4	5 minutes	4 days	N/R
Tarn 2023	I	Hospital	108	120 seconds	54 days	N/R
	I	Hospital	108	120 seconds	54 days	N/R
**Trans Cranial stimulation**						
Cancelli 2018	I	Hospital	5	15 mins	5 days	N/R
	I	Hospital				
Charvet 2018	I	Home	10	20 mins	2 weeks	N/R
	I	Home	10	20 mins	2 weeks	N/R
	I		20	20 mins	4 weeks	N/R
Salemi 2019	I	N/R	10	15 mins	2 weeks	N/R
	I					
Tecchio 2015	I	Hospital	5	15 mins	5 days	N/R
	I					
Granja-Dominguez 2022	I	Hospital	20	45 mins	4 weeks	N/R
	I					
Mostert 2005	I	Hospital	10 per week	16 mins	3-4 weeks	N/R
	I					
Piatkowski 2009	I	Home	24	8 mins	12 weeks	N/R
	I	Home	24	8 mins	12 weeks	N/R
Voggenberger 2022	I	Home	Daily	30 minutes	30 days	N/R

	I	Home	Daily	30 minutes	30 days	
**Aromatherapy**						
Hawkins 2019	I	Home	Daily	15 minutes	14 days	N/R
	I	Home	Daily	15 minutes	14 days	N/R
**Acupuncture/ acupressure**						
Horta 2020	I	N/R	9	20 mins	7 weeks	3 senior acupuncturists
	I	N/R	9	20 mins	7 weeks	3 senior acupuncturists
Kluger 2016	I	Clinic	12	30 mins	6 weeks	Licensed acupuncturist
	I	Clinic	12	30 mins	6 weeks	N/A
**RIC**						
Moyle 2023	I	Hospital or home	18	40 minutes	6 weeks	Researcher, self, or carer
	I	Hospital or home	18	40 minutes	6 weeks	Researcher, self, or carer

6.3 Nutritional Interventions

Study/ Population	Individual/ group	Setting	Number of sessions	Duration of sessions	Total duration of intervention	Intervention provider
**Fish oil**						

Arriens 2015	I	Home	6 capsules per days	N/A	6 months	N/A
	I	Home	6 capsules per days	N/A	6 months	N/A
**Thiamine HD**						
Bager 2021	I	Home-based	N/A	N/A	12 weeks	Herlev Hospital Pharmacy
	I	Home-based	N/A	N/A	12 weeks	Herlev Hospital Pharmacy
**5-HTP**						
Truyens 2022	I	Home-based	N/A	N/A	16 weeks	University Hospital Ghent Clinical Trial Unit
	I	Home-based	N/A	N/A	16 weeks	University Hospital Ghent Clinical Trial Unit
**Flavenoid - cocoa**						
Coe 2019	I	Home-based with an optional home visit in week 3	N/A	N/A	6 weeks	N/R
	I	Home-based with an optional home visit in week 4	N/A	N/A	6 weeks	N/R
**Diet**						
Chase 2023	I	Home	Daily diet. Plus 2 to 3 diet counselling sessions.	N/R	12 weeks	Dieticians
	N/A	N/A	N/A	N/A	12 weeks	N/A

**Plant**						
Johnson 2006	I	Home-based	N/A	N/A	4 weeks	Dr. Wilmar P. Schwabe Company, Gmb, Germany
	I	Home-based	N/A	N/A	4 weeks	Dr. Wilmar P. Schwabe Company, Gmb, Germany

Supplemental Results 7
Intervention characteristics, studies not included in NMA
7.1 Intervention content

Behavioural Interventions						
**Study/ Population**	**Pop.**	**Study N**	**Intervention (as named in study)**	**Intervention description**	**Intervention aim**	
**Self-Management**						
**Fatigue self-management - conservative**						
Finlayson 2011	MS	190	Teleconference Fatigue Management Program	Discussions about fatigue; how to communicate about fatigue; body mechanics; activity analysis - evaluating priorities; living a balanced life - taking control of your day; goal setting.	To teach behavioural changes that will lead to improvement in fatigue severity and HRQoL	
			Wait List	Usual daily activities	Control	
			Progressive Muscle Relaxation +RAU	A standardized series of relaxation exercises (involving 11 large muscle groups) combined with deep breathing + rehabilitation as usual	To achieve enhanced mental relaxation by reducing muscle tension	
Kos 2007	MS	51	Multidisciplinary Fatigue Management Programme	Information concerning possible strategies to manage fatigue and reduced energy levels, ie, pharmacological treatment, diet, informing and involving the social environment, regular sleep, exercise, relaxation, cooling, assistive devices, adaptation of home or work environment and energy saving methods	To reduce the impact of MS fatigue on daily life	

			Placebo Intervention Programme	Information on topics that did not concern themes directly related to fatigue (ie, car adaptations and driving abilities, communication skills, lift techniques for back protection and general information about MS)	Active control	
Mathiowetz 2005	MS	169	Energy Conservation Course	Based on theory of psychoeducational group development. Long and short term goal setting; practice activities and homework. Importance of rest throughout the day, positive and effective communication; proper body mechanisms; ergonomic principles; modification of the environment; changing standards; setting priorities; activity analysis and modification; living a balanced lifestyle.	To determine whether energy conservation education can reduce the impact of fatigue in persons with MS	
			Wait list	Usual activities	A control	
**Fatigue self-management - active**						
O'Connor 2019	IBD	23	Psychoeducation	Structured around psychological and physical interventions, which were geared towards understanding fatigue, energy conservation, management strategies and improving relaxation techniques tailored to the specific needs of patients with IBD.	To test whether or not fatigue, energy and quality of life indices could be improved	
			Usual care	Standard medical care	Control	
Vogelaar 2014	IBD	98	Solution Focused Therapy	Solution-focused course, focussing on coping styles for fatigue. Psychoeducation about IBD and fatigue and SFT. Focus is on the existing adequate coping abilities of patients, rather than on their problems.	To develop coping skills to enhance fatigue management	
			Usual care	Received care as usual	Control	

**CBT – fatigue**						
van Kessel 2016	MS	39	MSInvigor8 + support	Interactive CBT self-management programme with email support from a clinical psychologist. Explanation of MS fatigue and the CBT approach; topics such as activity scheduling, improving sleep, altering unhelpful thinking and patterns and behaviour, managing stress, coping with emotions, social support, preparing for the future.	To test the addition of email support to the MSINVIGOR8 programme	
			MSInvigor8	Interactive CBT self-management programme as above with no email support	Control.	
Voet 2014	FSHD	57	CBT	Modules based on known fatigue perpetuating factors. Directed at insufficient coping with their disease; dysfunctional cognitions regarding fatigue, activity, pain or other symptoms; fatigue catastrophising; dysregulation of sleep or activity; poor social support; negative social interactions.	To alleviate individually relevant fatigue-perpetuating factors.	
			Aerobic exercise	Cycling exercises on an ergometer, with cardiovascular monitoring. Aim to achieve 50%-60% increase in heart rate reserve.	To increase exercise which plays a central role in perpetuating fatigue	
**Physical Activity**						
**Physical activity promotion**						
McNelly 2016	IBD	52	Omega-3 and exercise	Individual consultation with a personal trainer provided at week 1. Advice consisted of personalised goal-setting using the treatment paradigm of treat-to-target to initiate an increase in physical activity levels of at least 30%.	To compare the effectiveness of individual advice to increase physical activity (PA) and/or ementation with omega-3 fatty acids on fatigue in patients with inactive IBD	

			Placebo and exercise	A 15-minute conversation with the researcher about the participant’s dietary habits and general health was undertaken at week 1, including questions such as: ‘Can you tell me about your current dietN/R’, ‘Did you have to change your diet following the diagnosis of IBDN/R’ and ‘In what way has IBD affected your general healthN/R’ No advice was given by the researcher regarding exercise.	As above	
			Omega-3 and no exercise	A total daily oral dose comprised 2970mg of pharmaceutical-grade omega-3 fatty acids — 2250mg of EPA and 150mg of DHA (takeOmega3, Edinburgh, UK)—in three capsules. Guidelines suggest that doses of up to 3g per day of marine-derived omega-3 fatty acids are safe, and a high EPA:DHA ratio is thought to be preferable.	As above	
			Placebo and no exercise	Capsules with a similar appearance to the omega-3 supplement capsules, but which contained a placebo: capric and caprylic acid.	Control	
Callahan 2008	RA	346	PACE exercise programme	A land-based exercise programme to promote self-management of arthritis through exercise	Exercise programs of moderate intensity are proposed to improve HRQoL in individuals with rheumatoid arthritis	
			Wait list	Usual activities	Control	
**Exercise – supervised**						
Avaux 2016	SLE	45	Supervised exercise	Endurance exercises (walking or bicycle) with the aim of achieving between	SLE patients have<br>a lower cardiovascular capacity and a lower muscle strength compared	

				60 and 80% of the theoretical maximal heart rate; and (ii): strengthening exercises (with elastoband or weights for both upper and lower limbs). Plus education about benefits of exercise.	to controls, suggesting that fatigue could be improved by exercise	
			Home exercise	As above but unsupervised	To test benefits of supervision	
			Control	No training (participants who declined to train or refused their allocation)	Control	
Coghe 2018	MS	22	Physical activity	Supervised training program	To improve processing speed, fatigue, and motor performance in patients with multiple sclerosis	
			Usual care	Usual daily activities	Control.	
			Control	N/R	Control	
Englund 2022	MS	140	High-Intensity Resistance Training (HIRT) - Group A	Resistance training	To compare the effects of high-intensity resistance training (HIRT) on self-reported fatigue	
			High-Intensity Resistance Training (HIRT) - Group B	Resistance training	As above with fewer sessions	
			Control	No intervention	Control.	
			Usual care	Usual daily activities	Control.	
**Exercise – unsupervised**						
Daltroy 1995	RA and SLE	71	Home cardiopulmonary	Stationary bicycles were provided for the exercisers. Each subject was asked to	Stimulating longer-term compliance by providing	

			conditioning programme	exercise to achieve a heart rate of 60-80% of the maximum heart rate achieved on the ETT. Pulse meters were provided to help patients monitor their heart rates and as a compliance-enhancing strategy. The physical therapist instructed the patient at home when setting up the bike, and made a second visit 2-3 weeks later at an exercise session to check the patient's ability to follow the regimen correctly.	patients with initial gains in endurance and self-confidence, but without the costs associated with long-term, supervised training.	
			Control	Encouraged to maintain current level of activity during the programme and as an attention control the physical therapist would ring in weekly.	Control	
Drory 2001	ALS	25	Exercise	Received list of exercises involving most muscle groups of the four limbs and trunk. The exercise program was developed for each patient, individually taking into account his general health, neurological status and actual fitness level. The main purpose of the exercise program was to improve muscle endurance, having the muscles work against only modest loads but undergo significant changes in length. The exercise program was demonstrated to each patient individually and reviewed at each clinic visit.	To determine the effect of moderate regular exercise under professional guidance on various parameters of HRQoL	
			Usual	Instructed not to perform any physical activity besides their usual daily life requirements. Every 14 days they also received a phone call.	Control	
Plow 2022	MS	170	Physical activity plus fatigue self-management	Group teleconference sessions + individually tailored phone calls. Taught how to engage in a pedometer-based walking programme, set goals, overcome obstacles, and self-monitor progress. Additional content adapted from the	N/R	

				Managing Fatigue programme.		
			Physical activity only	Group teleconference sessions + individually tailored phone calls. Taught how to engage in a pedometer-based walking programme, set goals, overcome obstacles, and self-monitor progress.	N/R	
			Contact control	Generic health information<br>(e.g. healthy eating and preventive screening).	Control	
Robb-Nicholson 1989	SLE	23	Aerobic conditioning	Exercise at home for 30 min three<br>times per week for 8 weeks to attain 60-80% of their maximum heart rate achieved during the exercise tolerance test (the target range). Walking, cycling or jogging were permitted.	To determine the effects of aerobic conditioning in SLE	
			Non-aerobic exercise	Non-aerobic stretching exercises	Control	
**Active recreational**						
Palsdottir 2020	Stroke	101	Nature-based rehabilitation	Daily themed sessions: morning gathering with a cup of herbal tea, allowing participants to feel at ease after travelling from their homes; physical activities, such as a garden walk, tricycling, or “on the spot” exercises, which were held indoors in the greenhouses when the weather was not favourable; garden and horticultural occupation, in a group or on their own, or “just being” (i.e. mental recovery on their own enjoying the garden); and gathering for “closure for the day”, with some light refreshments harvested from the garden, fresh or preserved.	Offering an enriched environment and multiple sensory stimuli through meaningful nature-based occupations has been shown to improve general health and wellbeing.	

			Usual care	Usual daily activities	Control	
**Other psychological**						
Vogelaar 2011	CD	29	Solution-Focused Therapy	The solution-focused model offers a wide range of interventions that channel the attention of patients towards constructing possible solutions. SFT was modified to focus on fatigue management.	Fatigue contributes to impairment of HRQoL. No problem exists - the solution to a problem is finding the exception when no problem exists. Patients learn to be in the moment and the problem disappears.	
			Problem-solving Therapy	Based on a general model of problem solving, adjusted for the purpose of patients with Crohn's Disease.	To increase the capabilities of the patients to deal with the daily stressful problems caused by CD	
			Usual care	Standard medical care and no additional psychological interventions.	Control	

Stimulation Interventions						
**Study**	**Population**	**Study N**	**Intervention (as named in study)**	**Intervention description**	**Intervention aim**	
**Trans Cranial stimulation**						
Chalah 2020	MS	11	Tdcs & shAM	Active transcranial direct current stimulation (tDCS) and Sham	Brain stimulation to relieve fatigue	
DeDoncker 2021	Stroke	33	tDCS	Increase cortical excitability using anodal transcranial direct current stimulation (tDCS).	Increase cortical excitability to ease fatigue	
			Sham	Sham	Control	
Gaede 2018	MS	33	rTMS - left PFC	H6 coil rTMS over the left prefrontal cortex	PFC stimulation is effective for depression - potential use for fatigue is supported by the high overlap between fatigue and depressive symptoms. Stimulates circuits implicated in fatigue	
			rTMS - MC	H10 coil rTMS over the primary motor cortex bilaterally	PFC and MC stimulation directly targets circuits for which alterations in fatigue were reported	
			Sham stimulation	Sham rTMS over the left prefrontal cortex	Control	

Hidding 2017	PD	12	Conventional subthalamic nucleus stimulation	High frequency stimulation of the Subthalamic Nucleus Stimulation	LC might therefore represent an important structure in the pathogenesis of certain neuropsychiatric symptoms such as apathy, fatigue, or depression.	
			Combined subthalamic nucleus and substantia nigra stimulation	High frequency Stimulation of the subthalamic nucleus and substantia nigra stimulation	LC might therefore represent an important structure in the pathogenesis of certain neuropsychiatric symptoms such as apathy, fatigue, or depression.	
Saoite 2014	MS	14	Transcranial direct current stimulation (tDCS)	Cross over one block real tDCS, one block sham	To assess whether fatigue symptoms can be reduced by excitability-enhancing anodal transcranial direct current stimulation (tDCS).	
			Sham			
**External stimulation**						
DeCarvalho 2012	MS	50	Magnetic field therapy	Pulsed low frequency magnetic field	Beneficial effects of magnetic fields may improve fatigue	

			Sham	Sham	Control	
Mateen 2020	MS	35	Bright White Light Therapy (BWLT)	Light box with instructions. Participants were instructed to sit in front of the light box with eyes approximately 36″ from the light source to achieve desired LT exposure, aligned with their eyes and at a distance of 30 cm, at which 10 000 lux were achieved. Participants were instructed to keep their eyes open during the whole 30 min of light therapy.		
			Dim Red light therapy (DRLT)	As above with the only difference that a filter was installed that dimmed the light to 200 lux and tinted it red.		

Nutritional Interventions						
**Study**	**Population**	**Study N**	**Intervention (as named in study)**	**Intervention description**	**Intervention aim**	
**American Ginseng**						
Kim 2011	MS	56	Ginseng	100mg capsules/day week 1; 2 capsules/day week 2; 4 capsules/day week 3-6	Drug treatments available for MS fatigue are limited in their efficacy. Herbal treatments may help	
			Placebo	Placebo	Control	
**GLA**						

Theander 2002	Sjorgen's syndrome	87	GLA	800mg of GLA (Gammalinolenic acid) given daily	To evaluate GLA's efficacy on treating Sjorgen's syndrome with fatigue	
			GLA	1600mg of GLA (Gammalinolenic acid) given daily	To evaluate GLA's efficacy at a higher dose on treating Sjorgen's syndrome with fatigue	
			Placebo	Containing mainly corn oil and no GLA given daily	Control	
**Flavenoid - cocoa**						
Coe 2022	PD	30	High-flavanol cocoa drink	Intervention taken following an overnight fast, at the same time each morning. 1 sachet containing 18g of cocoa powder (high flavanoid cocoa (10.79mg/g), contained in silver air tight sachets (identical in appearance to the control) consumed with 200ml of rice milk each morning on an empty stomach, at least 15-30 minutes before any food or drink consumption. Followed usual medication and diet.	To test whether daily consumption of flavanoid reduce fatigue in those with Parkinson's	
			Low-flavanol cocoa drink	As above but with 1 sachet containing 18g of cocoa powder (low flavanoid cocoa (1.02mg/g)), contained in silver air tight sachets (identical in appearance to the intervention).	Control	
**Diet**						
Irish 2017	MS	34	Modified Paleolithic dietary intervention	Diet consists mainly of fish, grass fed and pasture-raised meats, vegetables, fruits,	Evaluation of a modified Paleolithic	

				fungi, roots, and nuts; excludes grains, legumes, and dairy products; and limits refined sugars, starches, processed foods, and oils. The Paleo diet is relatively high in vitamins B, D, E, and K, polyunsaturated fatty acids, coenzyme Q10, α-lipoic acid, polyphenols, carotenoids, zinc, and selenium.	dietary intervention in the treatment of relapsing-remitting multiple sclerosis. One symtom includes fatigue, so this was one of the major topics they based the study on	
			Usual care (control)	Typical physician recommendations for MS	Control	
Lee 2021	MS	15	Modified Paleolithic diet	The modified Paleolithic diet (Wahls PaleoTM Diet) includes: 1) nine daily recommended servings of vegetables comprised of leafy green vegetables, sulfur rich vegetables, and deeply coloured fruits and vegetables; 2) encourages plant and animal protein, seaweed, nutritional yeast, non-dairy milks; and 3) excludes gluten-containing grains, eggs, casein. Participants were given the Whole Life Nutrition Cookbook	Used as a comparator to the other 2 groups tested	
			Medium-chain triglyceride (MCT)-based ketogenic diet	A ketogenic version of the modified Paleolithic diet with these additional requirements: 1) no starchy vegetables or fruit; 2)reduce vegetable consumption to 6 servings daily; and 3)increase fat intake with additional MCTs to achieve a daily goal of 70% of total calories from fat	Investigate the feasibility of a modified MCT-based ketogenic diet and its impact on plasma b-hydroxybutyrate and MS	
			Usual diet	Consume their pre-study vitamins, supplements, and/or medications.	Control	

7.2 Intervention delivery

Behavioural Interventions						
**Study/ Population**	**Individu al/ group**	**Setting**	**Number of sessions**	**Duration of sessions**	**Total duration of intervention**	**Intervention provider**
**Self-Management**						
**Fatigue self-management - conservative**						
Finlayson 2011	G	Teleconference	6	70 minutes	6 weeks	Licensed occupational therapist
	I	N/A	N/A	N/A	6 weeks	N/A
Kos 2007	G	Face to face	4	2 hours	4 weeks	Occupational therapist
	G	Face to face	4	2 hours	4 weeks	Occupational therapist
Mathiowetz 2005	G	Community	6	2 hours	6 weeks	Occupational therapists
	N/A	N/A	N/A	N/A	6 weeks	N/A
**Fatigue self-management - active**						
O'Connor 2019	G	Face to face	3	1 hour	6 months	Occupational therapist
	N/A	N/A	N/A	N/A	6 months	N/A
Vogelaar 2014	G	Face to face	6 + booster at month 6	1.5 hours	3 months	N/R
	N/A	N/A	N/A	N/A	3 months	N/A

**CBT – fatigue**						
van Kessel 2016	I	Web-based	8	25 to 50 minutes	8 weeks	Web-site with email support from a skilled clinical psychologist
	I	Web-based	8	25 to 50 minutes	8 weeks	Web-site with no therapeutic contact
Voet 2014	I	Face to face	Minimum 3 sessions	50 minutes	16 weeks	Cognitive behaviour therapist
	I	Home and supervised	3 supervised sessions and minimum 40 home sessions	30 minutes	16 weeks	Physical therapist
	N/A	N/A	N/A	N/A	16 weeks	N/A
**Physical Activity**						
**Physical activity promotion**						
McNelly 2016	I	Home-based	N/R	N/R	12 weeks	N/R
	I	Home-based	N/R	N/R	12 weeks	N/R
	I	Home-based	N/R	N/R	12 weeks	N/R
	I	Home-based	N/R	N/R	12 weeks	N/R
Callahan 2008	G	Community	At least one class	N/R	8 weeks	Exercise and health professionals
	N/A	N/A	N/A	N/A	8 weeks	N/A
**Exercise – supervised**						
Avaux 2016	I	Hospital validation centre	Individualised	3 hours per week	12 weeks	Multidisciplinary team

	I	Home	Individualised	3 hours per week	12 weeks	Unsupervised
	N/A	N/A	N/A	N/A	12 weeks	N/A
Coghe 2018	G	N/R	72 sessions	60 minutes	3 sessions per week for 24 weeks	Two coaches specializing in physical activity
	N/A	N/A	N/A	N/A	24 weeks	N/A
Englund 2022	G	Karolinska University Hospital, Stockholm, Sweden	24 sessions	60 minutes	2 sessions per week for 12 weeks	Physiotherapist
	G	Karolinska University Hospital, Stockholm, Sweden	12 sessions	60 minutes	1 sessions per week for 12 weeks	Physiotherapist
	N/A	N/A	N/A	N/A	N/A	N/A
**Exercise – unsupervised**						
Daltroy 1995	I	Home-based + physical therapist	36 sessions	30 mins	12 weeks	N/R
	I	Home-based + physical therapist	N/R	N/R	12 weeks	N/R
Drory 2001	I	Home-based	2 sessions/day	15 mins	12 months	N/R
	I	N/A	N/A	N/A	12 months	N/A
Plow 2022	G	Telephone	10	N/R	12 weeks	Occupational therapist + research assistant
	G	Telephone	10	N/R	12 weeks	Occupational therapist + research assistant

	G	Telephone	10	N/R	12 weeks	Occupational therapist + research assistant
Robb-Nicholson 1989	I	Home-based	24	30 minutes	8 weeks	Unsupervised
	I	Home-based	24	30 minutes	8 weeks	N/A
**Active recreational**						
Palsdottir 2020	G	Alnarp Rehabilitation garden	20	3.5 hours	10 weeks	Occupational therapist; horticulturalist; psychologist; physiotherapist
		N/A	N/A	N/A	10 weeks	Individualised multidisciplinary care
**Other psychological**						
Vogelaar 2011	N/R	Outpatients	5 sessions	N/R	3 months	Experienced psychotherapist
	N/R	Outpatients	10 sessions	N/R	3 months	Experienced psychotherapist
	N/A	N/A	N/A	N/A	3 months	N/A

**Stimulation Interventions**						
**Study/ Population**	**Individual/ group**	**Setting**	**Number of sessions**	**Duration of sessions**	**Total duration of intervention**	**Intervention provider**
**Transcranial Stimulation**						

Chalah 2020	I	Hospital	5 real, 5 sham	20 mins	5 days of each with 3 week washout period in-between	
DeDoncker 2021	I		2	20 mins	1 day	
	I		2	20 mins	1 day	
Gaede 2018	I	Clinic	3 sessions per week	16 minutes	6 weeks	N/R
	I	Clinic	3 sessions per week	16 minutes	6 weeks	N/R
	I	Clinic	3 sessions per week	16 minutes	6 weeks	N/R
Hidding 2017	I	Clinic	N/R	N/R	3 weeks	N/R
	I	Clinic	N/R	N/R	3 weeks	N/R
Saoite 2014	I	Clinic	5	20 minutes	5 days/ 2 weeks wash-out/ 5 days	
	I					
**External stimulation**						
DeCarvalho 2012	I	Outpatient dept.	24	24 mins	8 weeks	
	I					
Mateen 2020	I	Clinic/Home	15	30 mins	15 days	
	I					

**Nutritional Interventions**						
**Study/ Population**	**Individual/ group**	**Setting**	**Number of sessions**	**Duration of sessions**	**Total duration of intervention**	**Intervention provider**
**American Ginseng**						

Kim 2011	I	Home-based	N/A	N/A	6 weeks	Afexa Life Sciences, Edmonton, Canada
	I	Home-based	N/A	N/A	6 weeks	Afexa Life Sciences, Edmonton, Canada
**GLA**						
Theander 2002	I	Home-based	N/A	N/A	6 months	Scotia Pharmaceutical Ltd., Guilford, Surrey, UK
	I	Home-based	N/A	N/A	6 months	Scotia Pharmaceutical Ltd., Guilford, Surrey, UK
	I	Home-based	N/A	N/A	6 months	Scotia Pharmaceutical Ltd., Guilford, Surrey, UK
**Flavanoid - cocoa**						
Coe 2022	I	A hotel	N/A	N/A	6 days	OBU in the Oxford Brookes Centre for Nutrition and Health (OxBCNH) kitchen

	I	A hotel	N/A	N/A	6 days	OBU in the Oxford Brookes Centre for Nutrition and Health (OxBCNH) kitchen
**Diet**						
Irish 2017	I	Home-based plus visit every 2 weeks	Visit every 2 weeks visit	N/R	3 months	N/R
	I	Home-based plus visit every 2 weeks	Visit every 2 weeks visit	N/R	3 months	N/R
Lee 2021	I	Home based	Nutritional ketosis monitoring every 4 weeks	N/R	12 weeks	Wahls Paleo Diet + dietician
	I	Home based	Nutritional ketosis monitoring every 4 weeks	N/R	12 weeks	Wahls Paleo Plus + dietician
	I	Home based	Nutritional ketosis monitoring every 4 weeks	N/R	12 weeks	Dietician

Supplemental Results 8

Risk of Bias summary plots by intervention group

8.1 CBT-based interventions

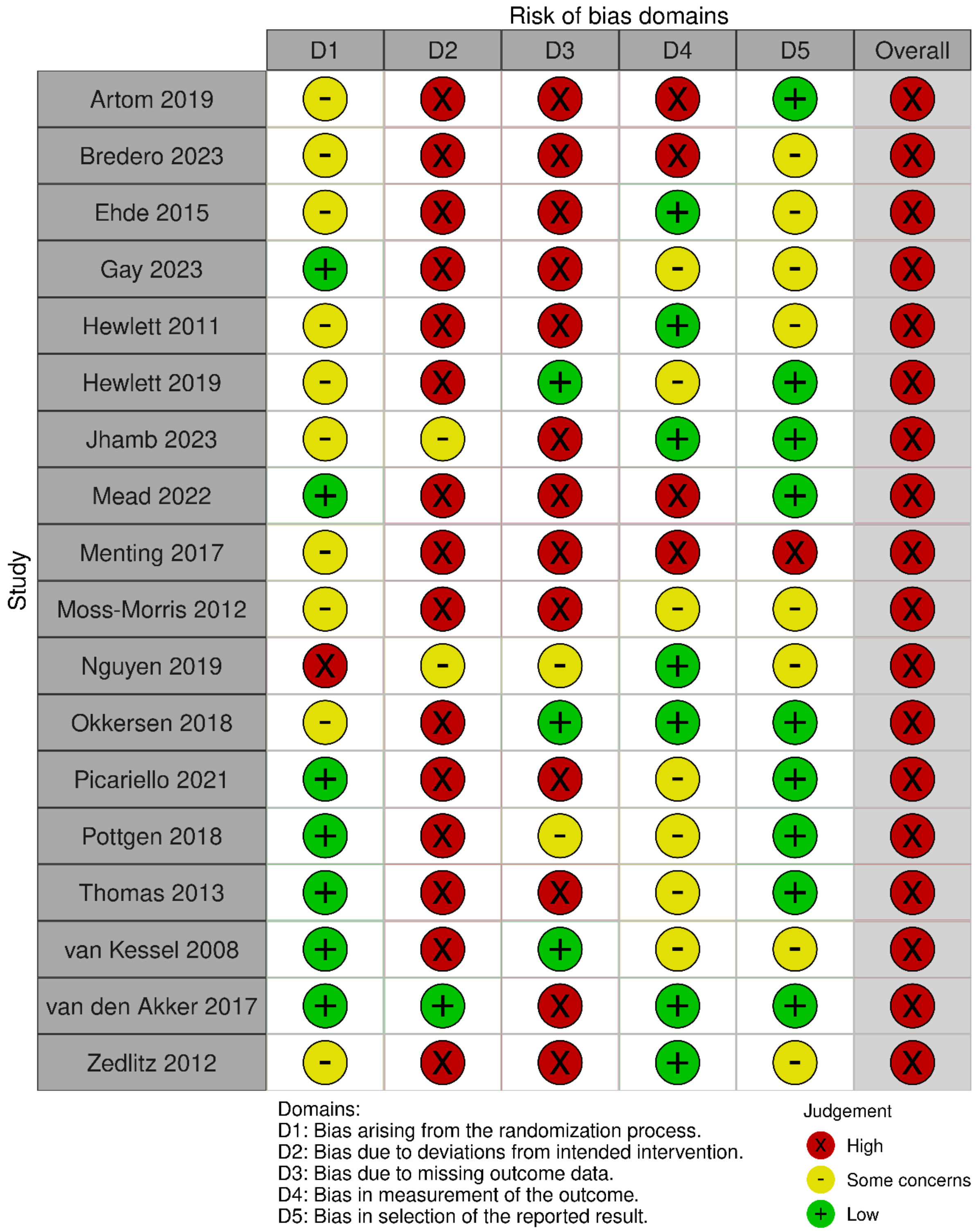

8.2 Fatigue self-management interventions

Risk of bias domains

Study	D1	D2	D3	D4	D5	Overall
Abonie 2020	-	X	X	X	-	X
Askari 2022	-	+	-	+	-	-
Blikman 2017	-	X	X	+	+	X
Farragher 2022	+	X	X	X	+	X
GarciaJalon 2013	+	X	X	+	-	X
Ghahari 2010	-	X	X	X	-	X
Hersche 2019	+	X	X	+	-	X
Hugos 2010	+	X	X	-	-	X
Hugos 2019b	+	X	+	-	-	X
Kos 2016	X	X	-	+	-	X
Murphy 2010	-	-	X	+	-	-
Clarke 2012	X	X	X	-	-	X
Murphy 2024	-	X	+	-	+	X
Rietberg 2014	X	X	+	X	+	X
Austin 1996	X	X	X	+	-	X
Feldthusen 2016	+	X	+	+	-	X
Hammond 2008	+	X	-	-	-	X
Khan 2020	+	X	-	-	+	X

Domains:
D1: Bias arising from the randomization process.
D2: Bias due to deviations from intended intervention.
D3: Bias due to missing outcome data.
D4: Bias in measurement of the outcome.
D5: Bias in selection of the reported result.

Judgement
X High
- Some concerns
+ Low

8.3 Mind-body interventions

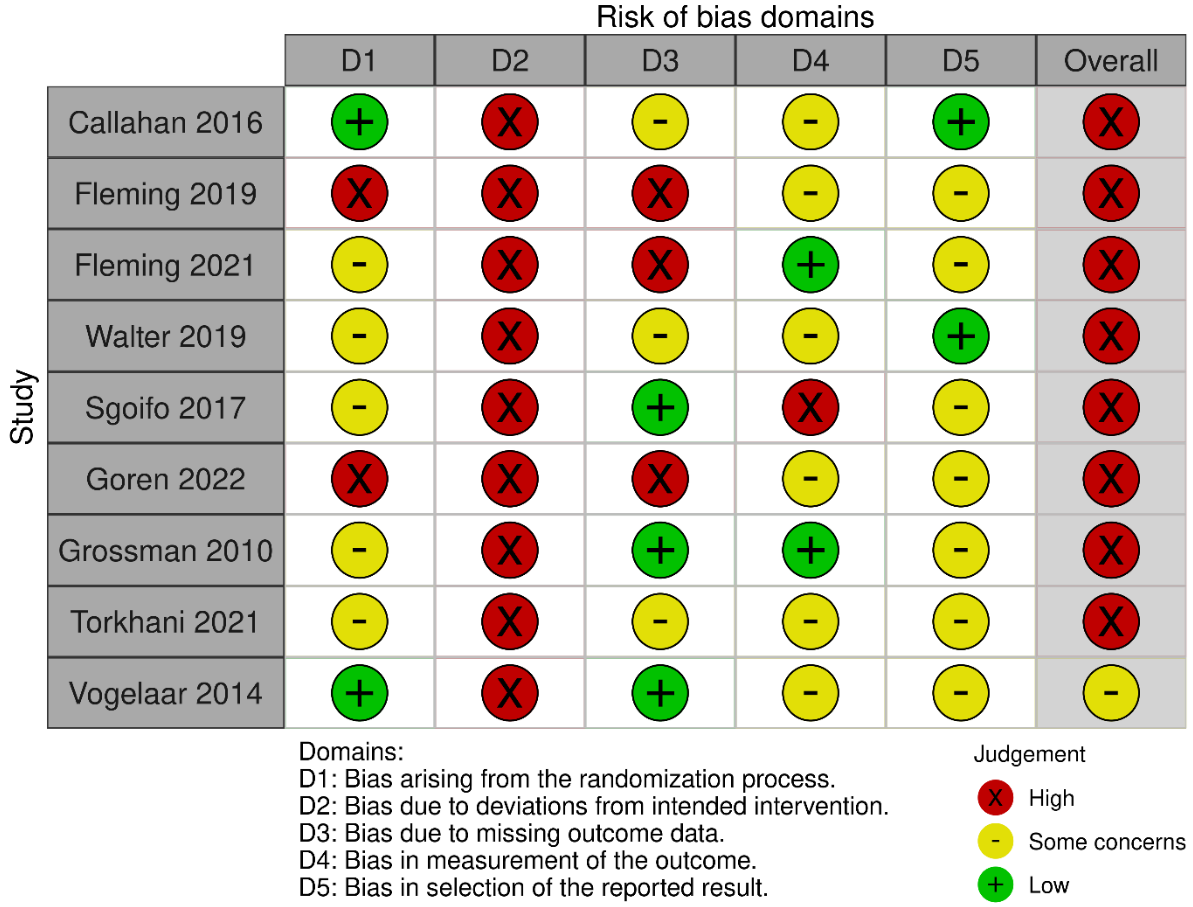

8.4 Physical Activity Promotion Interventions

Risk of bias domains

Study	D1	D2	D3	D4	D5	Overall
Bachmair 2022	+	X	+	+	+	X
Callahan 2014	X	X	-	+	-	X
Lutz 2017	X	X	X	+	-	X
Turner 2016	+	-	+	+	-	-
Dalgas 2010	-	X	X	-	-	X
Diaz 2023	+	X	+	-	-	X
Englund 2022	X	-	+	+	+	X
Escudero-Uribe 2017	-	X	X	+	-	X
Heine 2017	-	X	X	X	+	X
Feys 2019	X	X	X	-	-	X
Gervasoni 2014	-	X	X	+	-	X
Kratz 2020	-	X	-	-	-	X
Kucharski 2019	+	X	-	+	-	X
Langeskov-Christensen 2022	X	X	X	+	-	X
Louie 2022	-	X	X	+	-	X
McCullagh 2008	X	X	X	X	-	X
Ortiz-Rubio 2018	+	X	-	X	-	X
Pozehl 2008	X	-	-	-	X	X
Durcan 2014	X	X	-	-	-	X
Geddes 2009	-	X	-	-	X	X
Katz 2018	X	X	X	-	-	X
Maurer 2018	+	X	X	-	-	X
Tench 2003	-	X	X	-	-	X
Palsdottir 2020	-	X	-	+	+	X
DeGiglio 2015	-	X	-	+	-	X

Domains:
D1: Bias arising from the randomization process.
D2: Bias due to deviations from intended intervention.
D3: Bias due to missing outcome data.
D4: Bias in measurement of the outcome.
D5: Bias in selection of the reported result.

Judgement
X High
- Some concerns
+ Low

8.5 External stimulation interventions

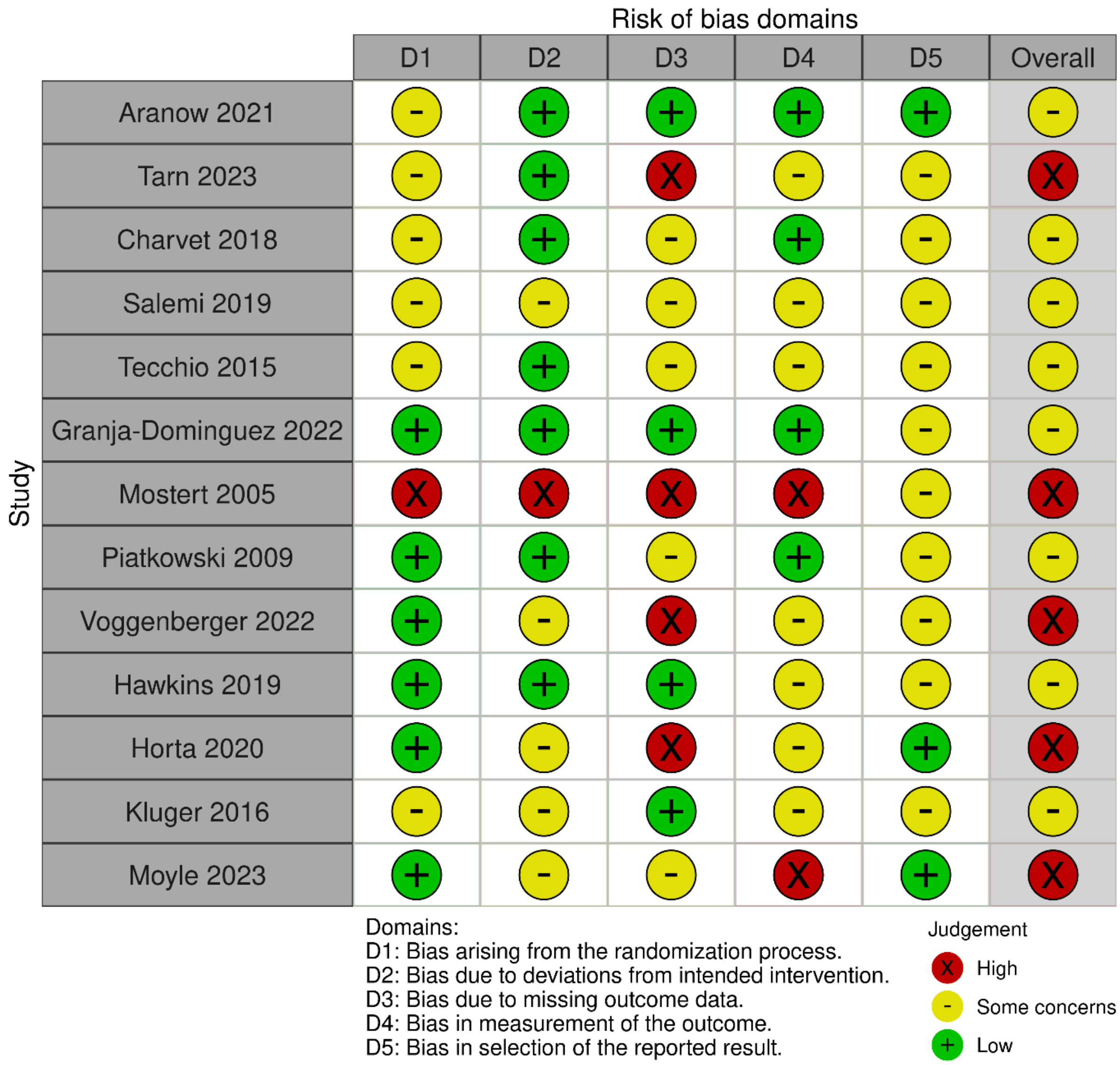

8.6 Nutritional and other supplement interventions

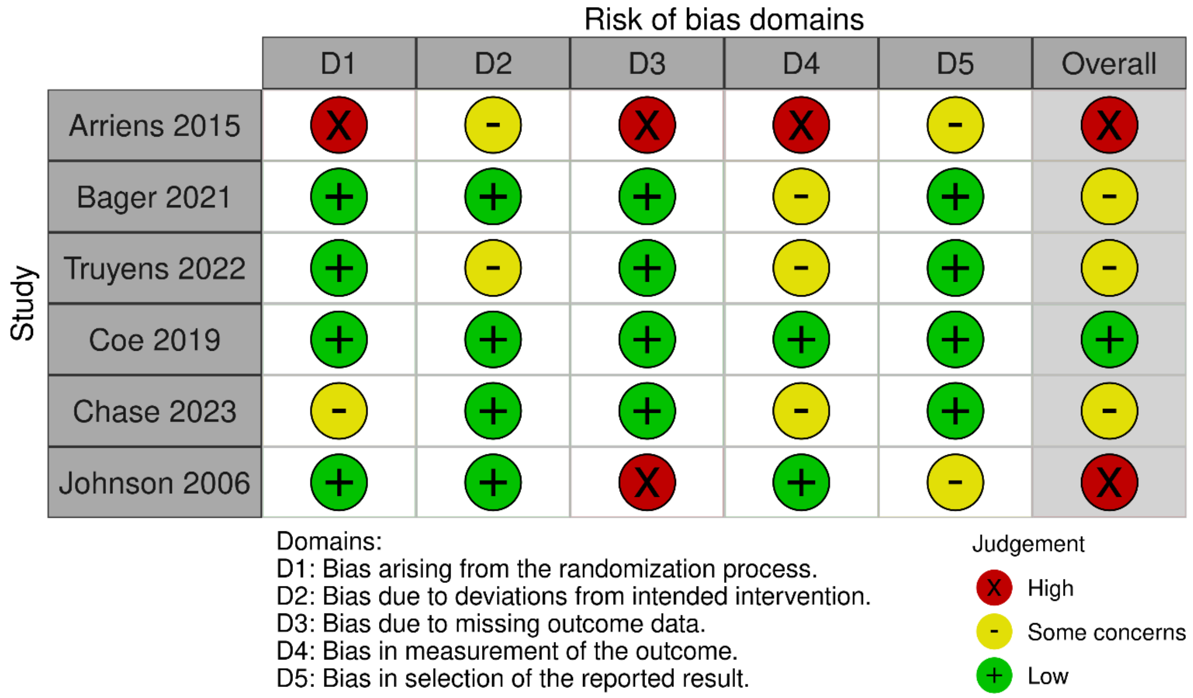

Supplemental Results 9 Additional statistical reporting

All network diagrams are labelled with three letter intervention identifiers, as summarized in Table 1.

Table 1 Three letter intervention identifier codes included within network diagrams.

Code	Intervention	Code	Intervention
_Co	Control	MBI	Mind-body intervention
_IE	Info/ Education	MIN	Mindfulness based
_UC	Usual care	nFIS	Fish Oil
_WL	Wait list	nFLV	Flavenoid (Cocoa)
ACU	Acupuncture/ pressure	nHTP	5-HTP
ARO	Aromatherapy	nTHI	Thiamine
CBT	CBT-fatigue	PAP	Physical activity promotion
DIE	Diet	PLA	Plant-based
EST	External stimulation	PSY	Other psychological
EXS	Exercise (supervised)	REH	Non-specific rehabilitation
EXU	Exercise (unsupervised)	RIC	Remote Ischaemic Conditioning
FMA	Fatigue management (active)	TCS	Transcranial Stimulation
FMC	Fatigue management (conservative)	VNS	Vagal Stimulation
GEN	General self management	MBI	Mind-body intervention

9.1 Primary analysis: inconsistency checks

9.1.1 End of treatment (EOT)

The mean posterior residual deviances were compared between the unrelated mean effects model and NMA models, Figure 1. The following studies were identified as being below the $y = x - 0.5$ line indicating potential inconsistency within the network; Louie 2022[1], Fleming 2021[2] Menting 2017[3], Langeskov-Christensen 2022[4], Turner 2016[5] and Horta 2020[6]. The studies were checked for any errors in data extraction or noticeable population differences, but none were identified. Node-splitting was subsequently used to assess whether there was any statistically significant inconsistency within the network, none was identified and thus no further action was taken.

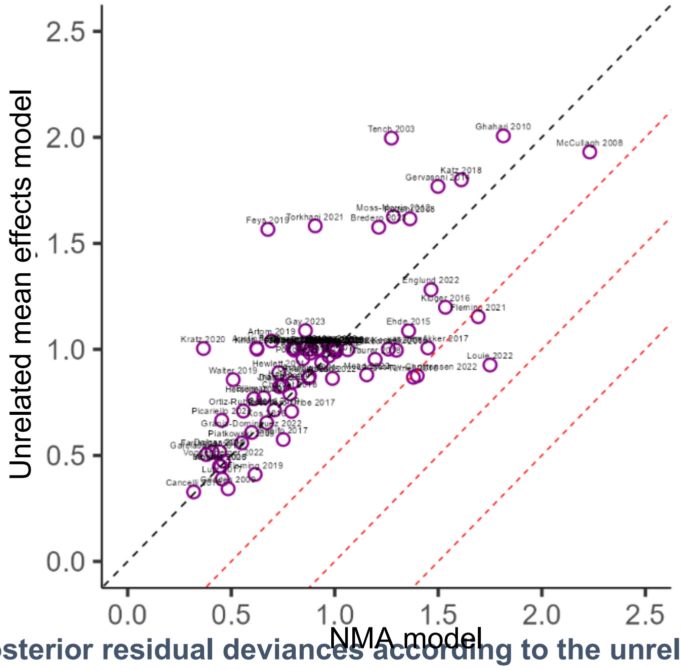

Figure 1 Mean posterior residual deviances according to the unrelated mean effects model and the NMA model, at end of treatment. Black dashed line is given by $y = x$, red dashed lines represent contours separated by differences of 0.5 between the two models. Any studies below the first red dashed line indicative of potential inconsistency.

9.1.2 Short term (ST)

The mean posterior residual deviances were compared between the unrelated mean effects model and NMA models, Figure 2. The following study was identified as being below the $y = x - 0.5$ line; Clarke 2012[7].The study was inspected for any errors that may have occurred during data extraction or noticeable population differences, but none were found. Node-splitting was subsequently used to assess whether there was any statistically significant inconsistency within the network, none was identified and thus no further action was taken.

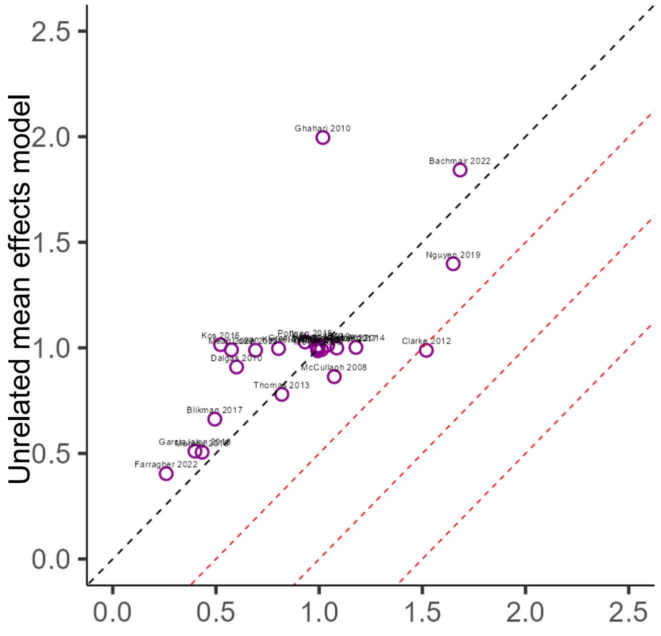

Figure 2 Mean posterior residual deviances according to the unrelated mean effects model versus the NMA model, at short term. Black dashed line is given by $y = x$, red dashed lines represent contours separated by differences of 0.5 between the two models. Any studies below the first red dashed line indicative of potential inconsistency.

9.1.3 Longer term (LT)

The mean posterior residual deviances were compared between the inconsistency and consistency models, Figure 3. No studies were identified as being below the $y = x - 0.5$ line, suggesting that there is no evidence of inconsistency within the network.

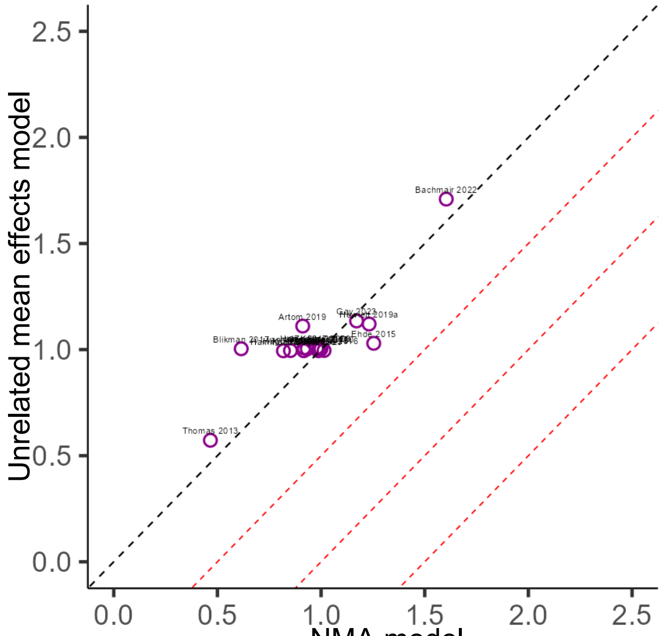

Figure 3 Mean posterior residual deviances according to the unrelated mean effects model and NMA model, at long term. Black dashed line is given by $y = x$ red dashed lines represent contours separated by differences of 0.5 between the two models. Any studies below the first red dashed line indicative of potential inconsistency.

9.2 Primary analysis: rankograms

A rankogram is a graphical output commonly used in network meta-analysis to display the probability that each treatment occupies each possible rank, from best to worst. For every treatment, the rankogram shows a distribution of probabilities across all ranks, reflecting the uncertainty in how treatments compare. Treatments with most of their probability mass concentrated toward the top ranks are more likely to be among the best options, whereas flatter or more spread-out curves indicate greater uncertainty in their relative performance. Rankograms should be interpreted alongside effect estimates and uncertainty intervals, as a high probability of being "best" does not necessarily mean a treatment has a clinically meaningful advantage.

9.2.1 EOT

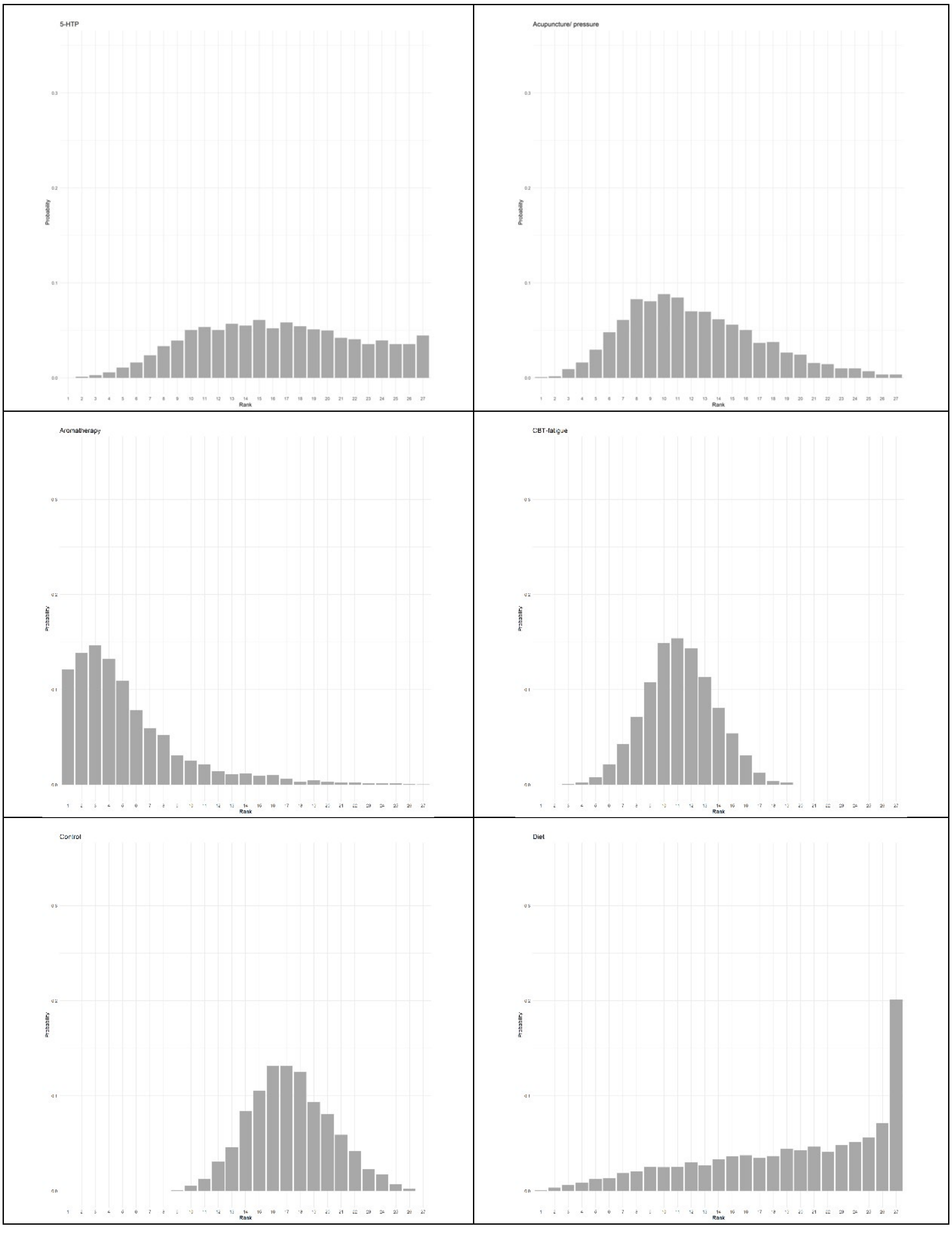

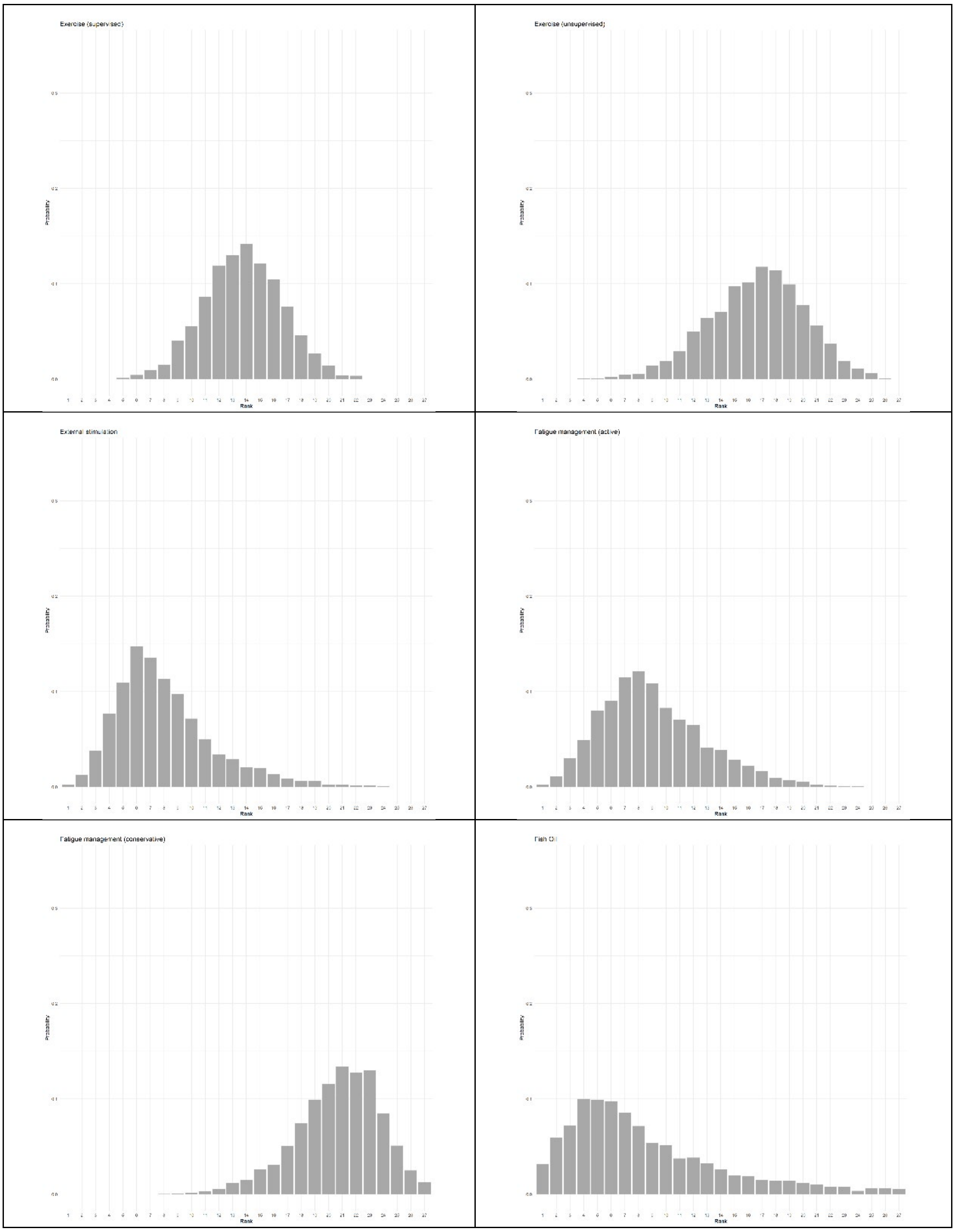
Exercise (supervised)
Exercise (unsupervised)
External stimulation
Fatigue management (active)
Fatigue management (conservative)
Fish Oil
Probability
Rank

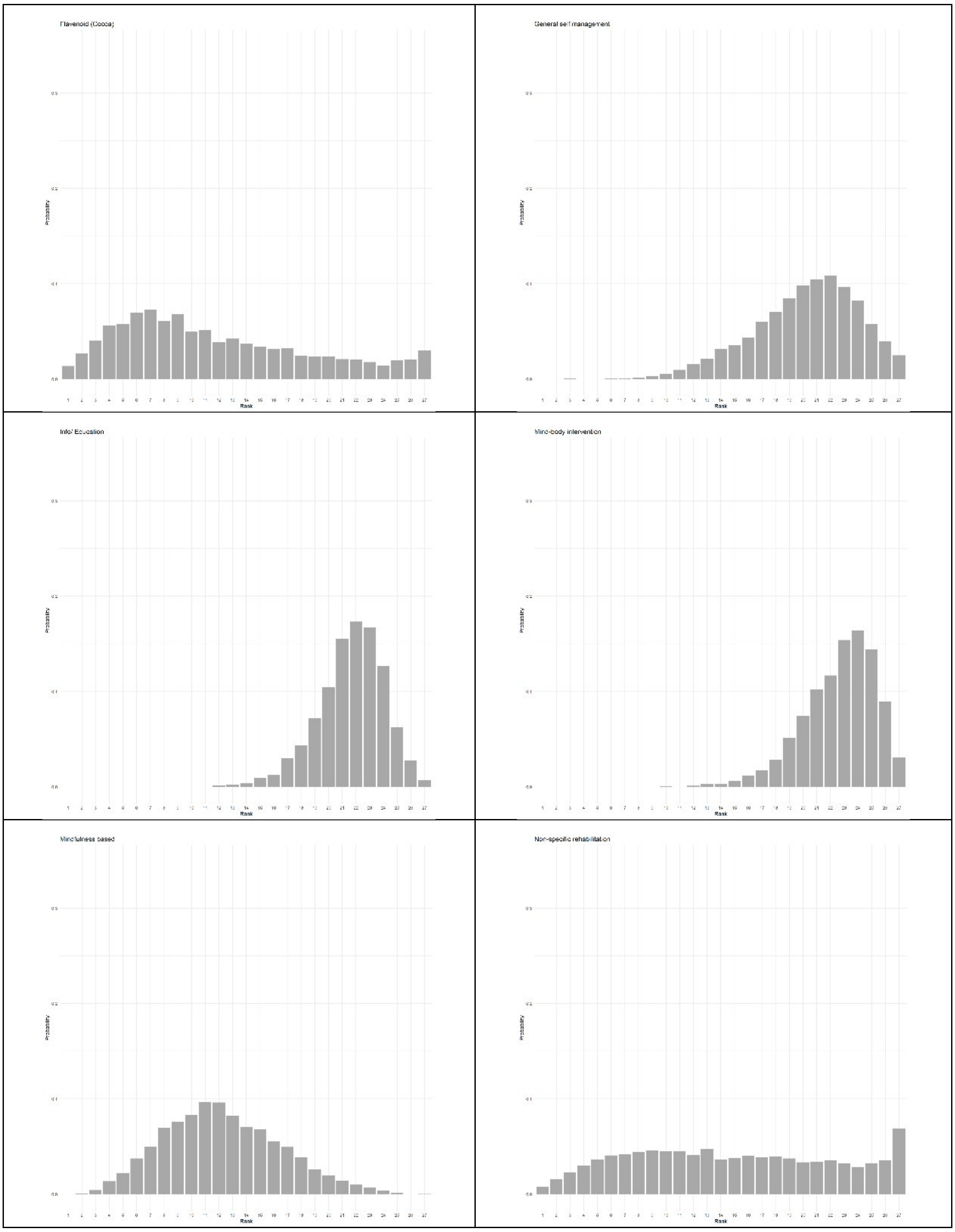
Flavenoid (Cocoa)
General self management
Info/ Education
Mind-body intervention
Mindfulness based
Non-specific rehabilitation
Probability
Rank

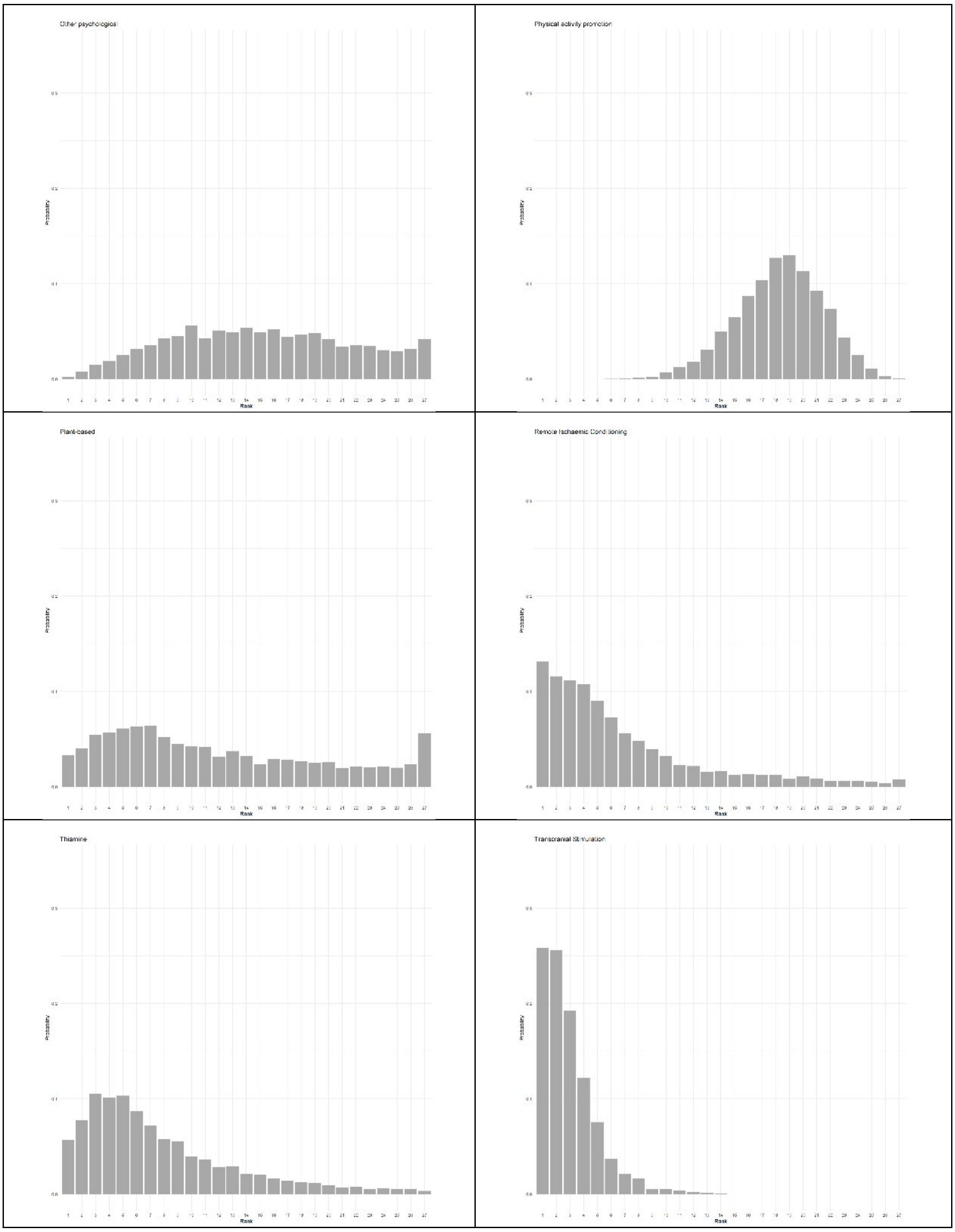
Other psychological
Physical activity promotion
Plant-based
Remote Ischaemic Conditioning
Thiamine
Transcranial Stimulation
Probability
Rank

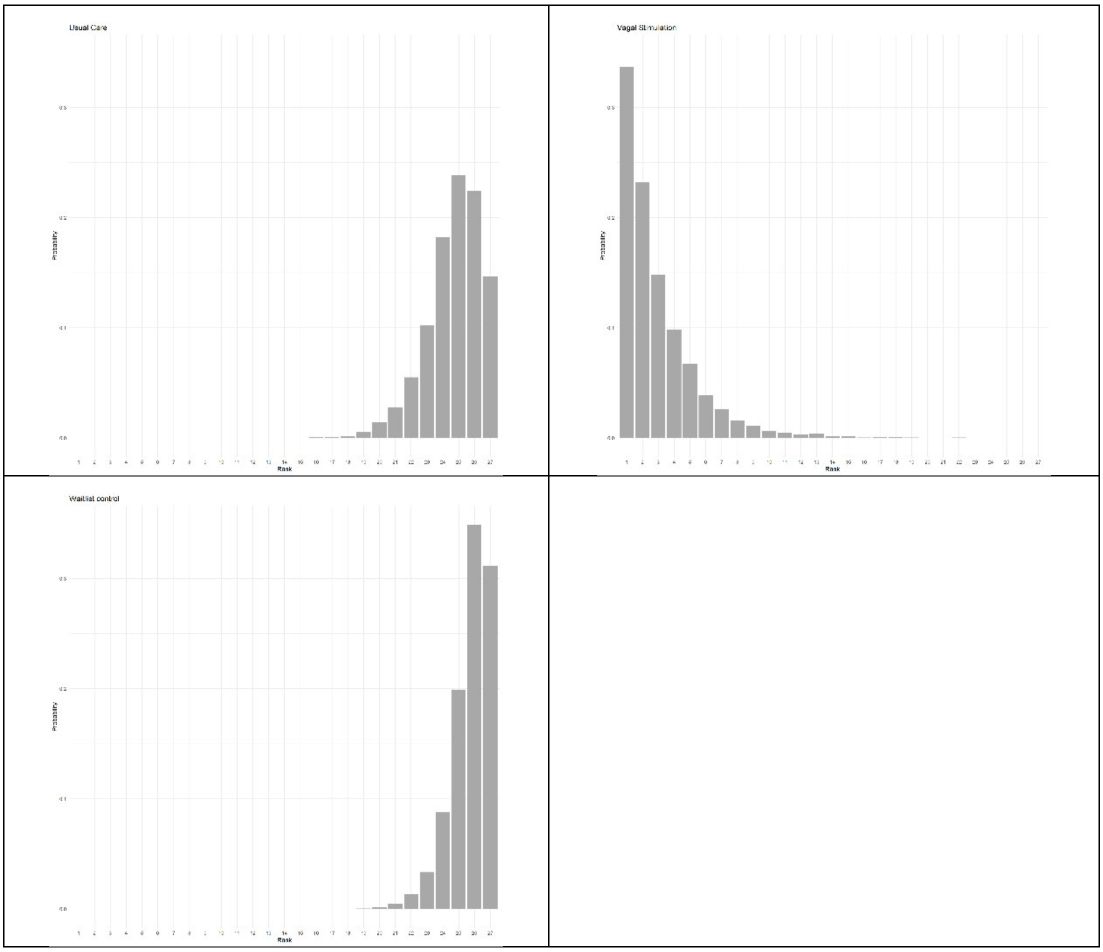
Usual Care
Vagal Stimulation
Waitlist control
Probability
Rank

9.2.2 ST

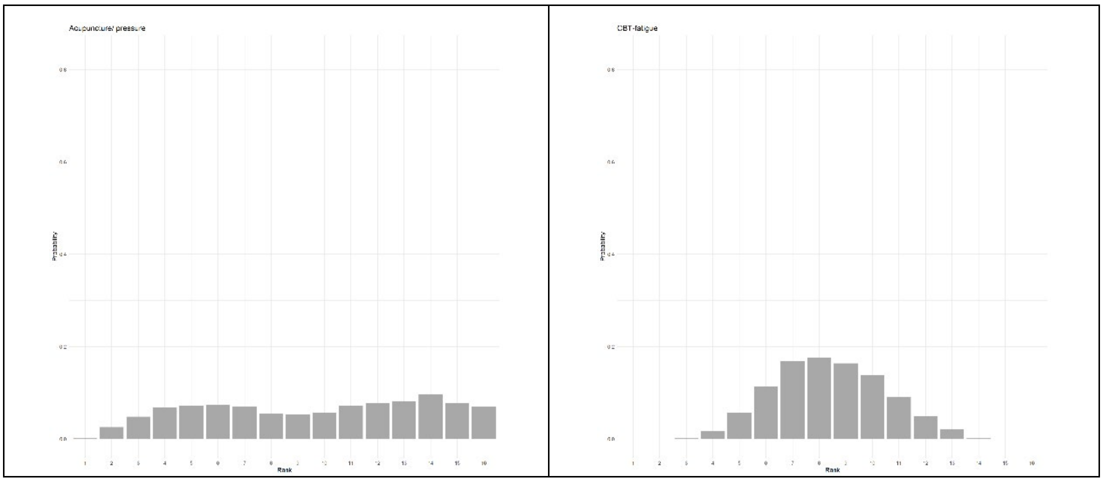
Acupuncture/ pressure
CBT-fatigue
Probability
Rank

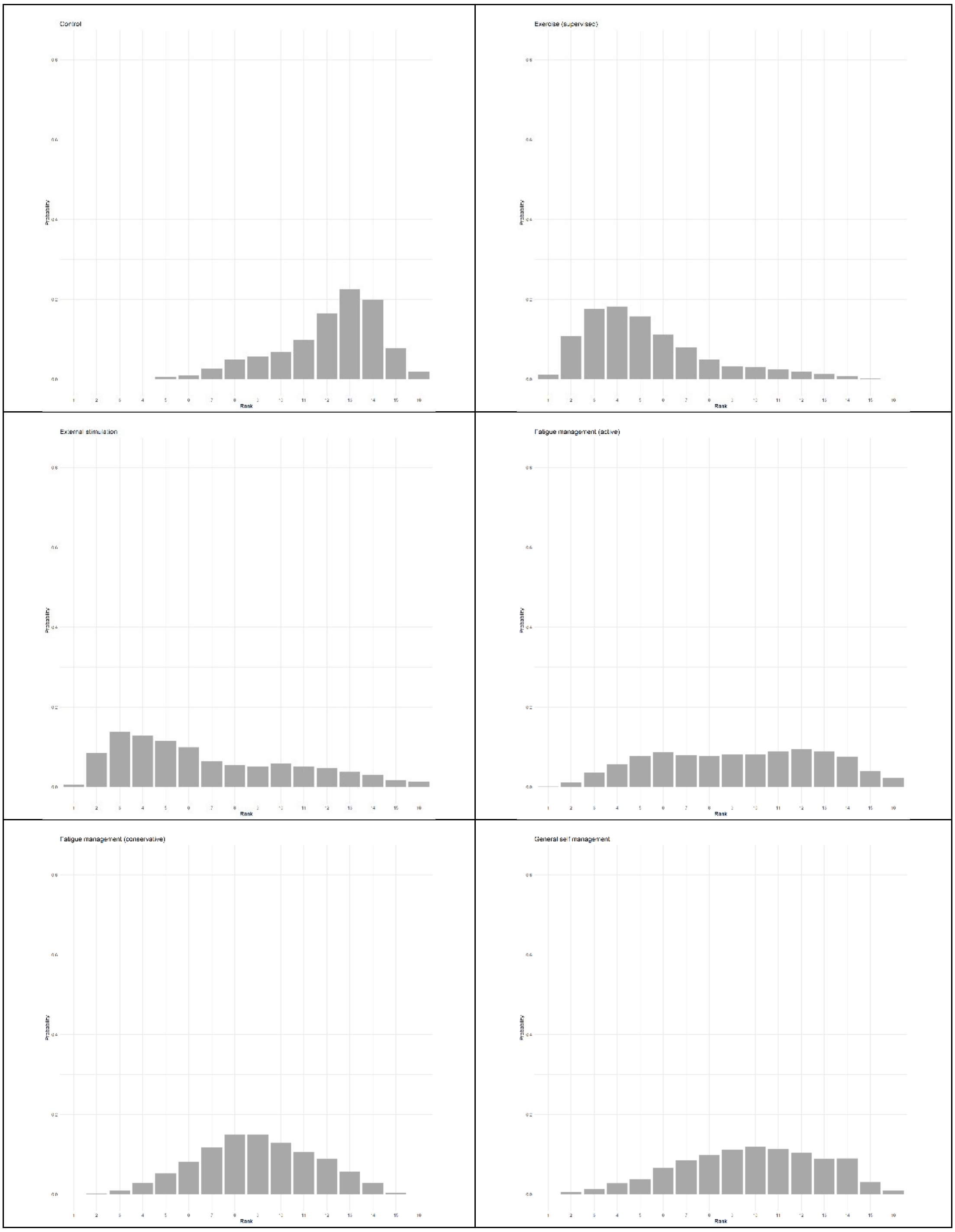
Control
Exercise (supervised)
External stimulation
Fatigue management (active)
Fatigue management (conservative)
General self management

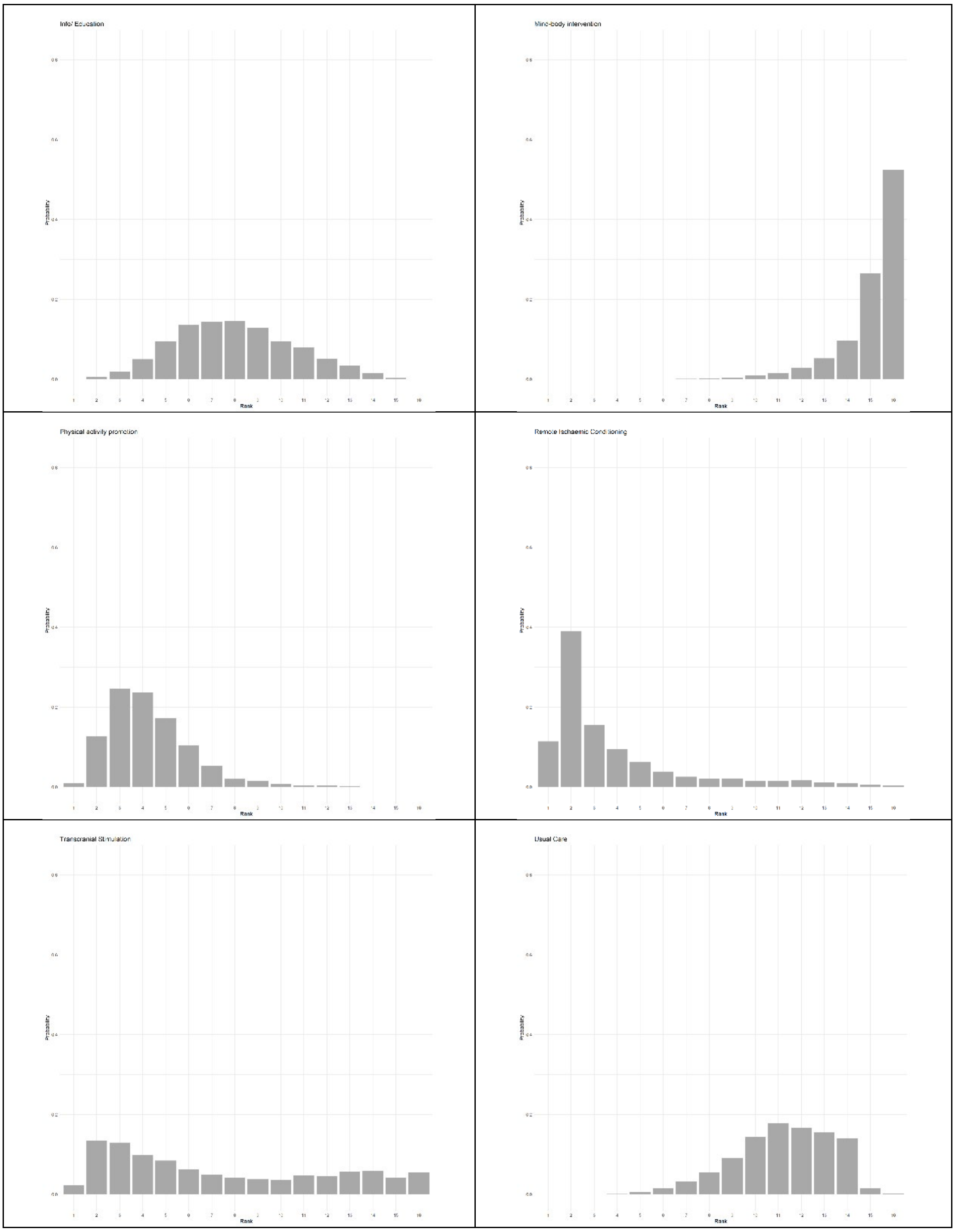
Info/ Education
Mind-body intervention
Physical activity promotion
Remote Ischaemic Conditioning
Transcranial Stimulation
Usual Care
Probability
Rank

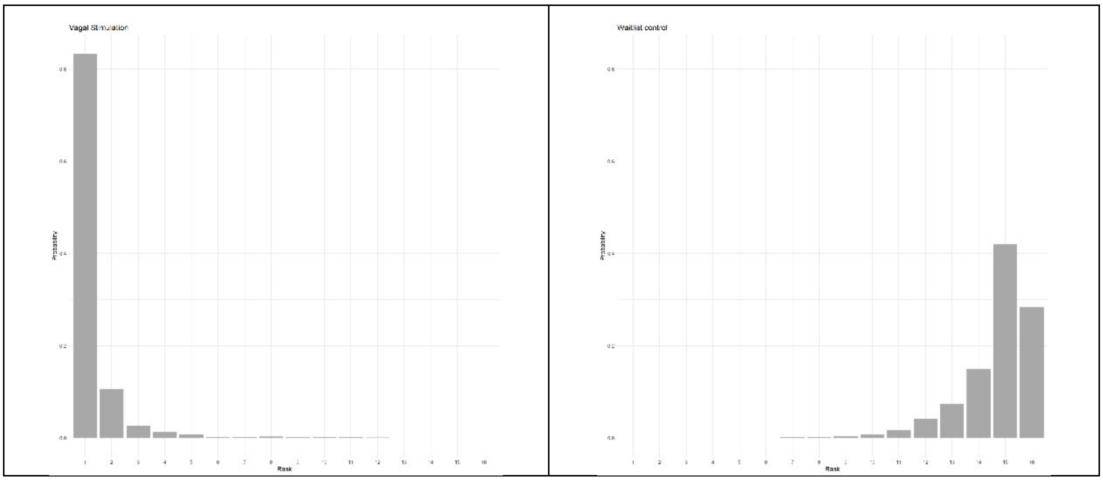
Vagal Stimulation
Waitlist control
Probability
Rank

9.2.3 LT

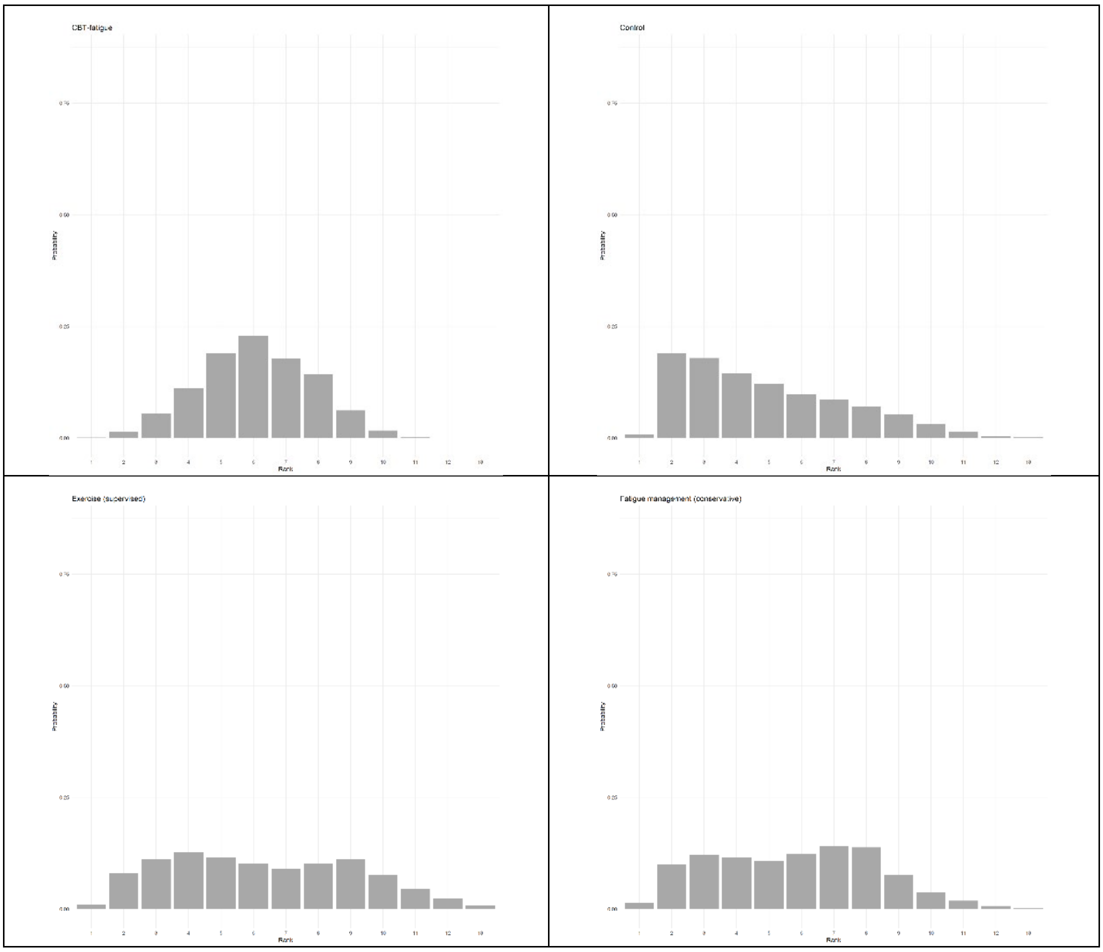
CBT-fatigue
Control
Exercise (supervised)
Fatigue management (conservative)
Probability
Rank

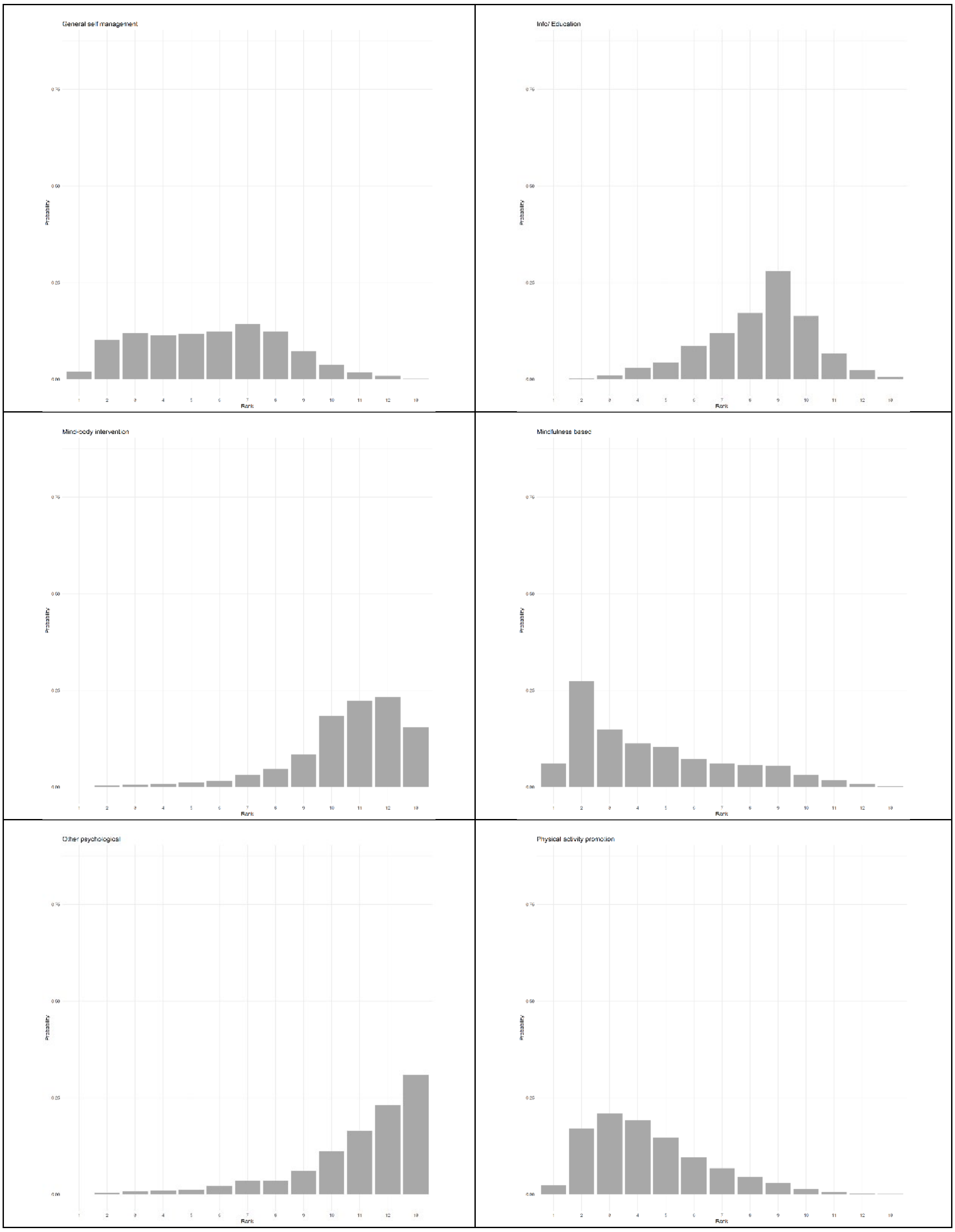
General self management
Info/ Education
Mind-body intervention
Mindfulness based
Other psychological
Physical activity promotion
Probability
Rank

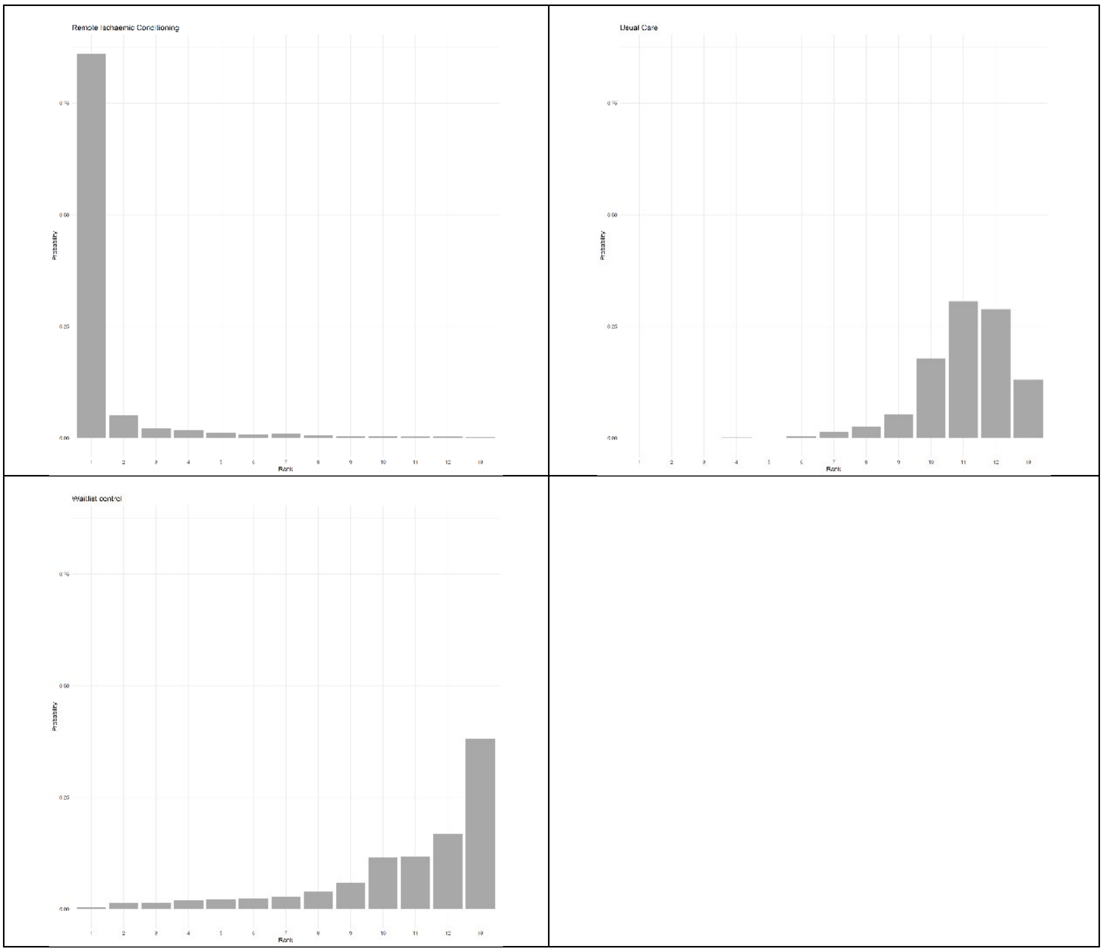

9.3 NMA scenario analysis: use of alternative data to inform the LT analysis

Data were available from 18 studies presenting a graded fatigue outcome at LT follow up. Five studies (Artom 2019[8], Ehde 2015[9], Gay 2023[10], Hammond 2008[11] and Hewlett 2019a[12]) presented alternative data for the LT follow-up at a time point closer to 3 months. These studies in the primary analysis had a follow up time of 10 months, 10 months, 12 months, 12 months and 46 weeks respectively. Within this scenario analysis, data collected at 4 months, 4 months, 6 months, 6 months and 20 weeks was instead used for each study in order to assess the potential impact of our decision to extract the longest available time points for the LT analysis. The network of evidence remains the same as that presented in Figure 3 of the main text.

The figure below shows the updated forest plot for the scenario analysis using alternative data for these five studies. There were no changes to which interventions were identified as statistically significant, though some minor differences were observed in the 95% credible intervals (CrIs). The between study heterogeneity was slightly increased in this analysis, compared with the LT primary analysis; 0.137 [95% CrI 0.009, 0.436] versus 0.096 [95% CrI 0.005, 0.356]. Both indicate moderate heterogeneity, but the slight increase is likely due to the inclusion of more variable follow-up times within this scenario.

Treatment	SMD	95% CrI	95% PrI	n	(N studies)
Behavioural					
Mindfulness based	-0.54	-1.056 to -0.035	-1.194 to 0.104	76	(1)
Physical activity promotion	-0.53	-0.928 to -0.125	-1.102 to 0.042	159	(2)
General self management	-0.44	-1.003 to 0.157	-1.125 to 0.280	231	(3)
Exercise (supervised)	-0.42	-0.985 to 0.135	-1.110 to 0.253	79	(2)
CBT-fatigue	-0.42	-0.684 to -0.196	-0.919 to 0.030	640	(9)
Fatigue management (conservative)	-0.40	-0.933 to 0.172	-1.053 to 0.304	175	(3)
Mind-body intervention	-0.03	-0.585 to 0.521	-0.716 to 0.641	60	(2)
Other psychological	0.04	-0.616 to 0.648	-0.727 to 0.761	45	(1)
Stimulation					
Remote Ischaemic Conditioning	-1.27	-2.335 to -0.233	-2.396 to -0.161	11	(1)
Other					
Control	-0.51	-1.045 to -0.014	-1.180 to 0.124	104	(3)
Info/ Education	-0.17	-0.603 to 0.299	-0.748 to 0.456	229	(4)
Waitlist control	0.03	-0.796 to 0.887	-0.882 to 0.980	34	(1)

Heterogeneity: between-study SD is 0.137 (95% CrI 0.009 to 0.436)

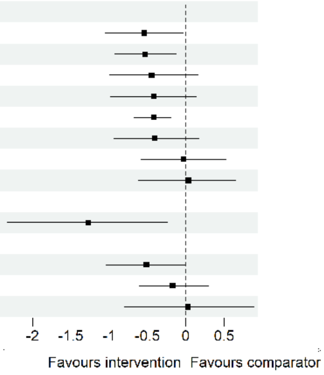

Figure 4 Estimated effects on fatigue outcomes of interventions, relative to usual care, at long term†, with 95% credible intervals (CrI) and 95% predictive intervals (PrI). The number of participants (n) and the number of studies (N studies) are given for context. Broad intervention categorisation is also presented to aid interpretation (Behavioural, Stimulation, Nutritional, and Other). The "control" node is displayed as this functioned to ensure connectivity of the network, but this is not an active intervention for consideration/recommendation. †Data for five studies changed to use earlier follow-up data within the long-term analysis time window.

9.4 NMA scenario analysis: exclusion of studies with <30 participants

The evidence base for the primary analysis consists of studies reporting results from RCTs with as few as 5 participants per arm. To assess the potential impact of the inclusion of small studies within the NMAs, we re-constructed the networks for EOT, ST and LT follow up, omitting any studies with a total number of participants lower than 30. This resulted in networks with 24, 13 and 12 connected interventions, informed by 62, 16 and 17 studies.

9.4.1 EOT

Generally, the NMA results when excluding small studies were similar to the primary analysis. Three interventions were no longer included in the network; remote ischaemic conditioning, transcranial stimulation, and plant based supplements. In the primary analysis, acupuncture was shown to exhibit statistically significant beneficial effects for fatigue outcomes, in this scenario analysis, the treatment effect of acupuncture/pressure was no longer shown to be statistically significant. Similarly, fish oil supplements in this scenario analysis were no longer found to be statistically significant. Physical activity promotion was informed by one less study and was no longer found to be statistically significant. The between study variance was comparable between this analysis and the primary analysis. Other than the changes listed above, there was generally a minor impact on treatment effect estimates and the associated 95% CrIs at EOT.

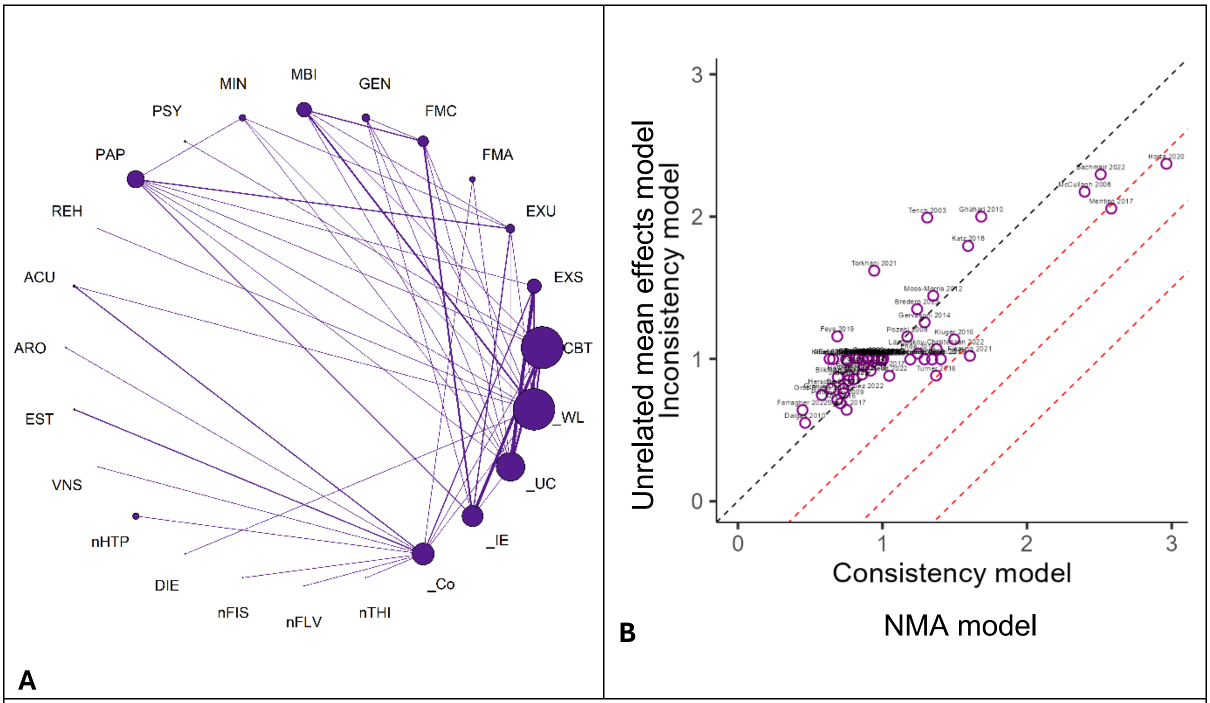

C

Treatment	SMD	95% CrI	95% PrI	n	(N studies)
Behavioural					
Fatigue management (active)	-0.67	-1.194 to -0.165	-1.428 to 0.069	138	(2)
CBT-fatigue	-0.60	-0.848 to -0.361	-1.202 to -0.009	974	(15)
Mindfulness based	-0.57	-0.992 to -0.151	-1.257 to 0.117	155	(3)
Exercise (supervised)	-0.46	-0.733 to -0.205	-1.078 to 0.138	334	(11)
Non-specific rehabilitation	-0.43	-1.318 to 0.471	-1.476 to 0.626	18	(1)
Other psychological	-0.42	-1.162 to 0.308	-1.344 to 0.489	45	(1)
Exercise (unsupervised)	-0.39	-0.751 to -0.032	-1.044 to 0.263	212	(5)
Physical activity promotion	-0.31	-0.624 to 0.007	-0.942 to 0.322	407	(6)
General self management	-0.23	-0.639 to 0.188	-0.911 to 0.458	189	(4)
Fatigue management (conservative)	-0.15	-0.498 to 0.211	-0.792 to 0.506	256	(7)
Mind-body intervention	-0.06	-0.401 to 0.279	-0.707 to 0.586	347	(7)
Stimulation					
Vagal Stimulation	-1.13	-2.119 to -0.144	-2.265 to -0.014	13	(1)
Aromatherapy	-1.09	-1.948 to -0.240	-2.114 to -0.085	27	(1)
External stimulation	-0.80	-1.494 to -0.112	-1.682 to 0.076	41	(2)
Acupuncture/ pressure	-0.57	-1.179 to 0.022	-1.394 to 0.228	65	(2)
Nutritional					
Thiamine	-0.91	-1.820 to -0.002	-1.973 to 0.145	20	(1)
Fish Oil	-0.84	-1.709 to 0.021	-1.864 to 0.177	25	(1)
Flavenoid (Cocoa)	-0.63	-1.534 to 0.269	-1.682 to 0.416	19	(1)
5-HTP	-0.35	-1.065 to 0.348	-1.251 to 0.534	152	(1)
Diet	-0.16	-1.014 to 0.707	-1.177 to 0.867	20	(1)
Other					
Control	-0.32	-0.712 to 0.064	-0.996 to 0.343	509	(14)
Info/ Education	-0.16	-0.455 to 0.121	-0.783 to 0.453	492	(12)
Waitlist control	0.11	-0.154 to 0.385	-0.496 to 0.727	964	(16)

Heterogeneity: between-study SD is 0.267 (95% CrI 0.178 to 0.378)

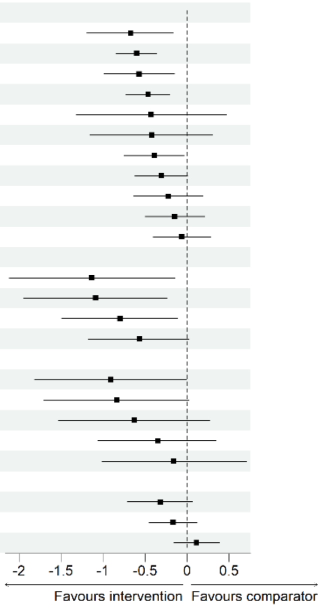

Figure 5 A) Network geometry, B) posterior residual deviances according to the unrelated mean effects model and NMA model, and C) estimated effects on fatigue outcomes of interventions, relative to usual care, with 95% credible intervals (CrI) and 95% predictive intervals (PrI); for the end of treatment analysis†, respectively. The number of participants (n) and the number of studies (N studies) are given for context. The "control" node is displayed as this functioned to ensure connectivity of the network, but this is not an active intervention for consideration/recommendation. †Data from small studies were excluded in this analysis.

9.4.2 ST

The SMDs and 95% credible intervals were mostly similar at ST follow-up when small studies were excluded. Three interventions were however no longer included in the network including: remote ischaemic conditioning, transcranial stimulation, and vagal stimulation interventions. The point estimate of the treatment effects for active fatigue management and acupuncture/pressure in the primary analysis were negative, with 95% CrI spanning zero effect, however in this secondary analysis, the point estimates were instead positive (still with 95% CrIs spanning zero). Between study variance was comparable between the two analyses.

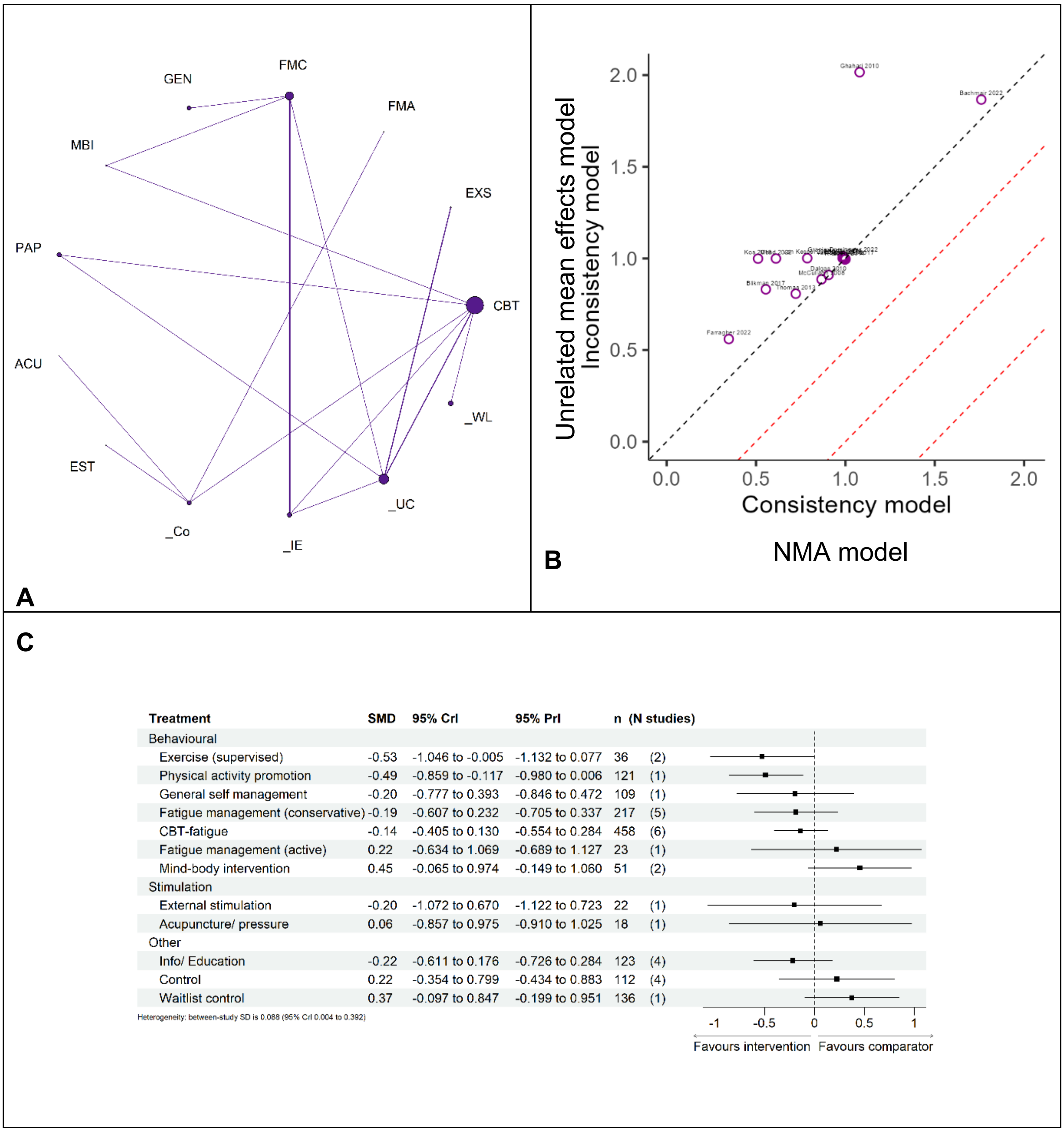

Treatment	SMD	95% CrI	95% PrI	n	(N studies)
Behavioural					
Exercise (supervised)	-0.53	-1.046 to -0.005	-1.132 to 0.077	36	(2)
Physical activity promotion	-0.49	-0.859 to -0.117	-0.980 to 0.006	121	(1)
General self management	-0.20	-0.777 to 0.393	-0.846 to 0.472	109	(1)
Fatigue management (conservative)	-0.19	-0.607 to 0.232	-0.705 to 0.337	217	(5)
CBT-fatigue	-0.14	-0.405 to 0.130	-0.554 to 0.284	458	(6)
Fatigue management (active)	0.22	-0.634 to 1.069	-0.689 to 1.127	23	(1)
Mind-body intervention	0.45	-0.065 to 0.974	-0.149 to 1.060	51	(2)
Stimulation					
External stimulation	-0.20	-1.072 to 0.670	-1.122 to 0.723	22	(1)
Acupuncture/ pressure	0.06	-0.857 to 0.975	-0.910 to 1.025	18	(1)
Other					
Info/ Education	-0.22	-0.611 to 0.176	-0.726 to 0.284	123	(4)
Control	0.22	-0.354 to 0.799	-0.434 to 0.883	112	(4)
Waitlist control	0.37	-0.097 to 0.847	-0.199 to 0.951	136	(1)

Heterogeneity: between-study SD is 0.088 (95% CrI 0.004 to 0.392)

Figure 6 A) Network geometry, B) posterior residual deviances according to the unrelated mean effects model versus the NMA model, and C) estimated effects on fatigue outcomes of interventions, relative to usual care, with 95% credible intervals (CrI) and 95% predictive intervals (PrI); for the short term analysis†, respectively. The number of participants (n) and the number of studies (N studies) are given for context. The "control" node is displayed as this functioned to ensure connectivity of

the network, but this is not an active intervention for consideration/recommendation. †Data from small studies were excluded in this analysis.

9.4.3 LT

In the LT follow-up analysis, only one study had fewer than 30 participants and was thus not included. Remote ischaemic conditioning was no longer included within the network but otherwise the point estimates and 95% CrI were similar to those reported in the primary analysis. As with the other time points, between study variance was comparable to that observed in the primary analyses.

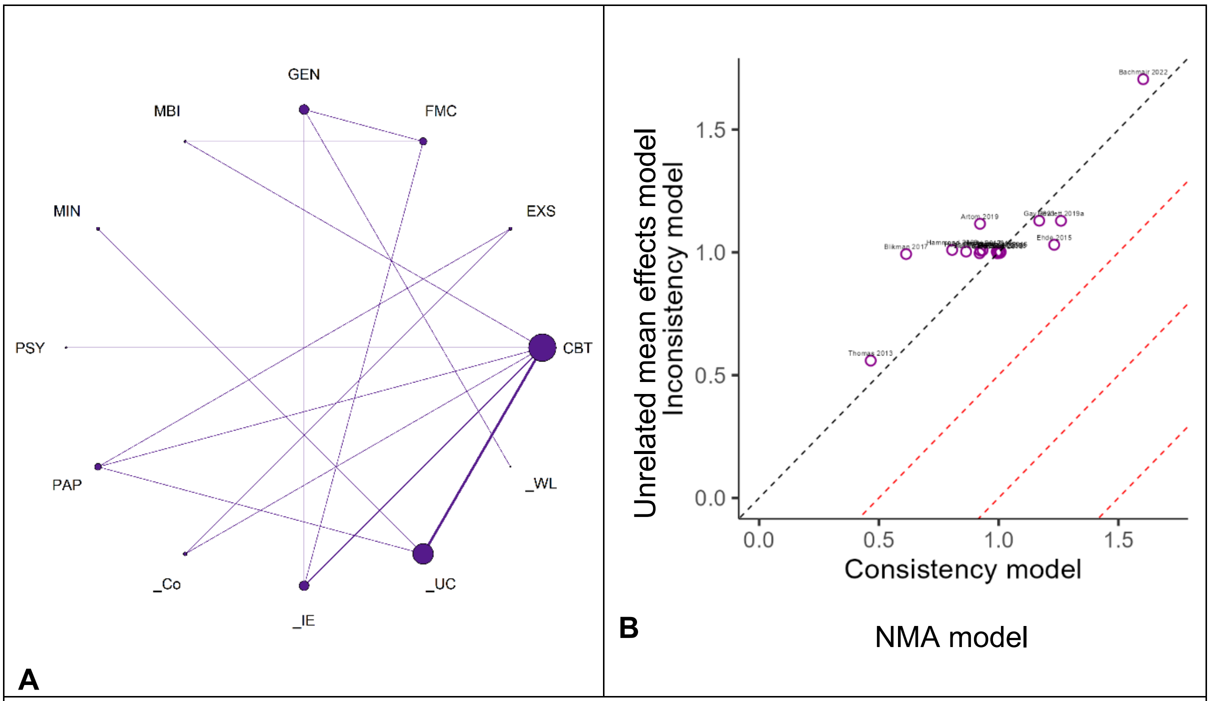

C

Treatment	SMD	95% CrI	95% PrI	n	(N studies)
Behavioural					
Mindfulness based	-0.54	-0.978 to -0.104	-1.076 to -0.009	76	(1)
Physical activity promotion	-0.52	-0.860 to -0.183	-0.977 to -0.060	159	(2)
General self management	-0.43	-0.918 to 0.096	-1.001 to 0.189	231	(3)
Fatigue management (conservative)	-0.42	-0.899 to 0.089	-0.973 to 0.184	175	(3)
Exercise (supervised)	-0.41	-0.911 to 0.087	-0.998 to 0.164	79	(2)
CBT-fatigue	-0.41	-0.621 to -0.214	-0.797 to -0.050	640	(9)
Mind-body intervention	-0.03	-0.529 to 0.473	-0.620 to 0.550	60	(2)
Other psychological	0.05	-0.526 to 0.600	-0.603 to 0.671	45	(1)
Other					
Control	-0.50	-0.971 to -0.048	-1.064 to 0.037	93	(2)
Info/ Education	-0.26	-0.643 to 0.150	-0.739 to 0.264	229	(4)
Waitlist control	0.04	-0.691 to 0.808	-0.744 to 0.871	34	(1)

Heterogeneity: between-study SD is 0.094 (95% CrI 0.005 to 0.356)

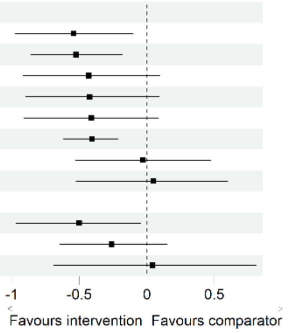

Figure 7 A) Network geometry, B) posterior residual deviances according to the unrelated mean effects model and the NMA model, and C) estimated effects on fatigue outcomes of interventions, relative to usual care, with 95% credible intervals (CrI) and 95% predictive intervals (PrI); for the long term analysis†, respectively. The number of participants (n) and the number of studies (N studies) are given for context. The "control" node is displayed as this functioned to ensure connectivity of the network, but this is not an active intervention for consideration/recommendation. †Data from small studies were excluded in this analysis.

9.5 NMA scenario analysis: relaxation of the transdiagnostic assumption

Condition group specific networks were constructed to help assess any potential differences in treatment effects across different condition groups. Due to the sparsity of evidence, networks could only be constructed using: EOT data for multiple sclerosis (MS), musculoskeletal conditions (MSK), inflammatory bowel disease (IBD), Kidney-related and Stroke-related conditions; ST for MS; and LT for MS and MSK. The results for each of these networks are presented below.

9.5.1 EOT

For the EOT condition-specific networks, Figure 8, there were 6 viable networks relating to the following condition groups: MS, MSK (two disconnected networks), IBD, Kidney, and Stroke. The largest EOT network was for MS with 19 interventions across 44 studies; the network originally included 46 studies, but statistically significant inconsistency was detected via node-splitting, which led to the removal of two studies, Fleming (2021)[2] and Turner (2016)[5] which provided direct evidence for the interventions flagged with statistically significant inconsistency. Two disconnected EOT networks were constructed for MSK: the first, “MSK #1”, included 10 interventions over 12 studies; the second, “MSK #2”, included 3 interventions over 3 studies. The EOT network for IBD included 8 interventions over 6 studies. Whilst the EOT networks for Kidney and Stroke each contained 4 interventions across 3 studies. Note that no inconsistency checking via node-splitting was not feasible for the following networks: MSK #2, Kidney, and Stroke, because the networks contained no closed loops of evidence.

The point estimates and 95% CrIs for the EOT condition-specific networks are shown in Figures 9-14; the treatment effect is relative to usual care unless otherwise stated. Several differences can be seen between the primary analysis where the transdiagnostic assumption is upheld and the condition group specific networks – these are detailed below.

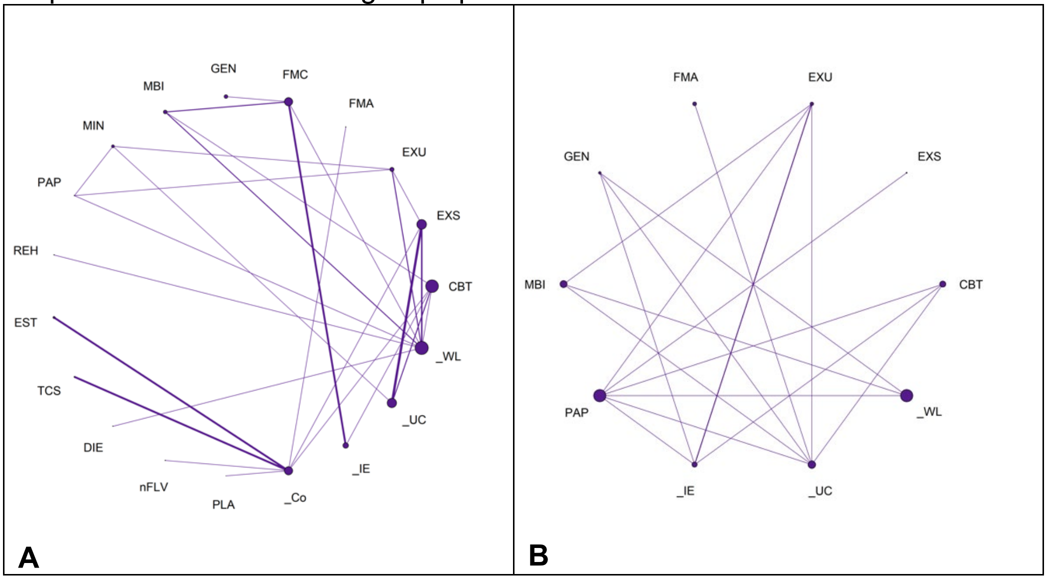

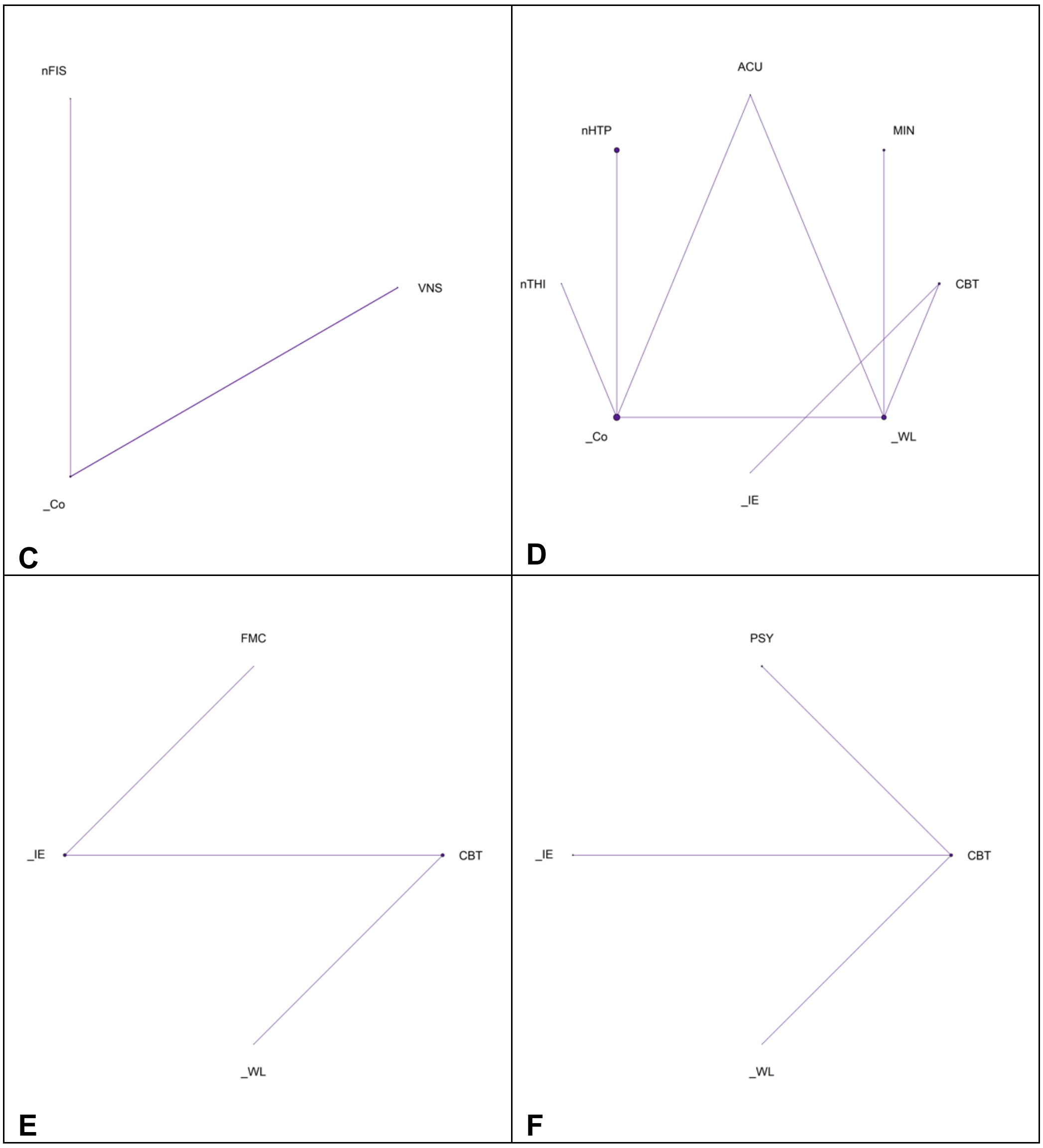

Figure 8 Network geometry for A) MS, B) MSK #1, C) MSK #2, D) IBD, E) Kidney, and F) Stroke condition-specific analyses, at end of treatment, respectively, indicating the number of participants who received each intervention (size of node) and the number of studies contributing to the direct evidence and comparisons between interventions (thickness of line).

In the EOT MS-specific network, all behavioural interventions (except physical activity promotion) were found to have beneficial, statistically significant effects on fatigue outcomes. Transcranial and external stimulation were also shown to have beneficial estimated effects. Of the nutritional interventions, only Flavenoid (cocoa) supplements was shown to have a potentially beneficial, statistically significant effect on fatigue outcomes, but as in the primary analysis, this was only evidenced by one study and should be interpreted with caution.
A number of treatments were found to have statistically significant effects which were not identified in the primary analysis, these included: non-specific rehabilitation, fatigue management (conservative), general self management, mind-body intervention and Flavenoid (Cocoa), suggesting these interventions may have improved effects on fatigue for individuals with MS. Conversely, physical activity promotion is not shown to have a statistically significant beneficial effect on fatigue outcomes. Furthermore, the treatment effect of waitlist control relative to usual care was non-beneficial for fatigue outcomes within the primary analysis but within the MS-specific network was shown to have a statistically significant, beneficial effect. Generally, treatment effects observed in the MS-specific

network were indicative of greater treatment effects on fatigue outcomes when compared to the primary analysis, however, the evidence base of the MS-specific network is approximately half of that analysed within the primary analysis (44 vs. 84 studies) and results should therefore be interpreted with appropriate caution.
The between study heterogeneity was found to be 0.12 [95% CrI 0.007, 0.312] which indicates moderate heterogeneity within the network.

Treatment	SMD	95% CrI	95% PrI	n	(N studies)
Behavioural					
Fatigue management (active)	-1.11	-1.892 to -0.322	-1.956 to -0.262	23	(1)
CBT-fatigue	-1.05	-1.352 to -0.738	-1.487 to -0.605	373	(6)
Non-specific rehabilitation	-0.95	-1.735 to -0.147	-1.792 to -0.084	18	(1)
Fatigue management (conservative)	-0.83	-1.279 to -0.358	-1.372 to -0.252	243	(8)
General self management	-0.79	-1.391 to -0.156	-1.458 to -0.080	109	(1)
Exercise (supervised)	-0.69	-0.940 to -0.441	-1.089 to -0.276	285	(10)
Mindfulness based	-0.65	-1.041 to -0.251	-1.151 to -0.129	88	(2)
Mind-body intervention	-0.53	-0.961 to -0.086	-1.061 to 0.015	109	(5)
Exercise (unsupervised)	-0.51	-0.920 to -0.089	-1.023 to 0.027	123	(4)
Physical activity promotion	-0.46	-1.127 to 0.203	-1.190 to 0.275	19	(2)
Stimulation					
Transcranial Stimulation	-1.53	-2.214 to -0.857	-2.281 to -0.791	34	(4)
External stimulation	-1.02	-1.596 to -0.440	-1.673 to -0.360	66	(4)
Nutritional					
Plant-based	-0.86	-1.827 to 0.124	-1.873 to 0.175	12	(1)
Flavenoid (Cocoa)	-0.84	-1.650 to -0.026	-1.701 to 0.036	19	(1)
Diet	-0.68	-1.434 to 0.094	-1.495 to 0.156	20	(1)
Other					
Info/ Education	-0.84	-1.277 to -0.367	-1.367 to -0.256	164	(5)
Control	-0.53	-0.961 to -0.090	-1.061 to 0.011	243	(13)
Waitlist control	-0.40	-0.716 to -0.077	-0.839 to 0.064	386	(12)

Heterogeneity: between-study SD is 0.12 (95% CrI 0.007 to 0.312)

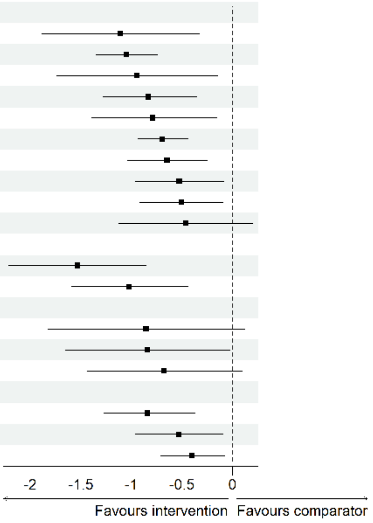

Figure 9 Estimated effects on fatigue outcomes of interventions within the MS-specific network, relative to usual care, at end of treatment, with 95% credible intervals (CrI) and 95% predictive intervals (PrI). The number of participants (n) and the number of studies (N studies) are given for context. Broad intervention categorisation is also presented to aid interpretation (Behavioural, Stimulation, Nutritional, and Other). The "control" node is displayed as this functioned to ensure connectivity of the network, but this is not an active intervention for consideration/recommendation.

In the EOT MSK #1 network, exercise (supervised) and fatigue management (active) had statistically significant, beneficial effects on fatigue outcomes, as in the primary analysis. However, although CBT-fatigue and physical activity promotion were found to be statistically significantly beneficial in the primary analysis, in the MSK #1 analysis, they were no longer found to be statistically significant. The evidence base of the MSK #1 network is however much smaller than the primary analysis network (12 vs 84 studies) and therefore the treatment effects presented should be interpreted with caution as no intervention featured across more than 4 MSK studies.
The between study heterogeneity was 0.138 [95% CrI 0.007, 0.498], which indicates a moderate study heterogeneity within the network.

Treatment	SMD	95% CrI	95% PrI	n	(N studies)
Behavioural					
Exercise (supervised)	-0.82	-1.546 to -0.072	-1.650 to 0.047	36	(1)
Fatigue management (active)	-0.54	-1.070 to -0.004	-1.216 to 0.155	115	(1)
Exercise (unsupervised)	-0.39	-0.836 to 0.038	-1.018 to 0.218	107	(3)
General self management	-0.22	-0.700 to 0.265	-0.871 to 0.424	80	(3)
Physical activity promotion	-0.21	-0.593 to 0.172	-0.793 to 0.376	365	(4)
CBT-fatigue	-0.18	-0.637 to 0.206	-0.825 to 0.391	182	(2)
Mind-body intervention	-0.07	-0.528 to 0.414	-0.699 to 0.594	210	(2)
Other					
Waitlist control	0.07	-0.360 to 0.544	-0.532 to 0.721	363	(3)
Info/ Education	0.11	-0.355 to 0.530	-0.546 to 0.694	155	(4)

Heterogeneity: between-study SD is 0.138 (95% CrI 0.007 to 0.498)

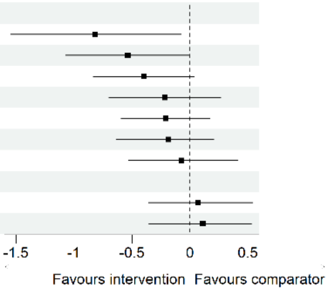

Figure 10 Estimated effects on fatigue outcomes of interventions within the MSK-specific network (#1), relative to usual care, at end of treatment, with 95% credible intervals (CrI) and 95% predictive intervals (PrI). The number of participants (n) and the number of studies (N studies) are given for context. Broad intervention categorisation is also presented to aid interpretation (Behavioural, Stimulation, Nutritional, and Other).

The EOT MSK #2 network treatment effects are presented relative to control and only included comparison of vagal stimulation and fish oil, Figure 11. As there were fewer than 5 studies within the 2nd network for MSK-related conditions, an informative prior on the between study heterogeneity was used. Vagal stimulation was found to be statistically significant with a positive effect on fatigue outcomes, however, this network only consisted of 3 studies, two of which influenced the vagal stimulation node, results should therefore be interpreted with caution. The between study heterogeneity was estimated to be 0.14 [95%CrI 0.027, 0.477] indicating moderate heterogeneity.

Treatment	SMD	95% CrI	95% PrI	n	(N studies)
Stimulation					
Vagal Stimulation	-1.04	-1.730 to -0.379	-1.853 to -0.260	25	(2)
Nutritional					
Fish Oil	-0.52	-1.214 to 0.180	-1.338 to 0.300	25	(1)

Heterogeneity: between-study SD is 0.14 (95% CrI 0.027 to 0.477)

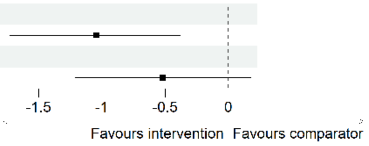

Figure 11 Estimated effects on fatigue outcomes of interventions within the MSK-specific network (#2), relative to control, at end of treatment, with 95% credible intervals (CrI) and 95% predictive intervals (PrI). The number of participants (n) and the number of studies (N studies) are given for context. Broad intervention categorisation is also presented to aid interpretation (Behavioural, Stimulation, Nutritional, and Other).

The EOT analysis for IBD conditions showed that none of the interventions were identified to have statistically significant effects relative to waitlist control. However, these interventions were informed by a maximum of 2 studies and thus have minimal evidence. In addition to the low number of studies, the between study heterogeneity standard deviation was found to be 1.385 [95% CrI 0.071, 2.694], which indicates extremely high heterogeneity amongst the 6 studies included within the network, and therefore these results should be interpreted accordingly.

Treatment	SMD	95% CrI	95% PrI	n	(N studies)
Behavioural					
Mindfulness based	-0.64	-4.107 to 2.846	-5.562 to 4.327	67	(1)
CBT-fatigue	-0.32	-3.814 to 3.212	-5.289 to 4.638	71	(2)
Stimulation					
Acupuncture/ pressure	-2.11	-5.656 to 1.479	-7.089 to 2.877	18	(1)
Nutritional					
Thiamine	-1.38	-6.313 to 3.598	-7.447 to 4.686	20	(1)
5-HTP	-0.81	-5.757 to 4.161	-6.818 to 5.218	152	(1)
Other					
Control	-0.78	-4.281 to 2.757	-5.722 to 4.195	191	(3)
Info/ Education	0.45	-4.512 to 5.402	-5.574 to 6.480	16	(1)

Heterogeneity: between-study SD is 1.385 (95% CrI 0.071 to 2.694)

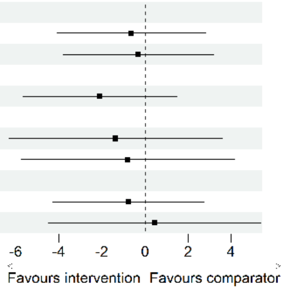

Figure 12 Estimated effects on fatigue outcomes of interventions within the IBD-specific network, relative to wait list, at end of treatment, with 95% credible intervals (CrI) and 95% predictive intervals (PrI). The number of participants (n) and the number of studies (N studies) are given for context. Broad intervention categorisation is also presented to aid interpretation (Behavioural, Stimulation, Nutritional, and Other). The "control" node is displayed as this functioned to ensure connectivity of the network, but this is not an active intervention for consideration/recommendation.

As there were fewer than 5 studies within the network for Kidney-related conditions, an informative prior on the between study heterogeneity was used. The analysis showed that neither CBT-fatigue nor fatigue management (conservative) were found to be statistically significantly beneficial for fatigue outcomes relative to wait list controls. This network was informed by only four studies and is likely underinformed. The between study heterogeneity was found to be moderate (0.143 [95%CrI 0.027, 0.483]).

Treatment	SMD	95% CrI	95% PrI	n	(N studies)
Behavioural					
CBT-fatigue	-0.49	-1.380 to 0.409	-1.481 to 0.502	95	(2)
Fatigue management (conservative)	-0.04	-1.371 to 1.278	-1.441 to 1.341	15	(1)
Other					
Info/ Education	-0.05	-1.088 to 0.999	-1.178 to 1.080	92	(2)

Heterogeneity: between-study SD is 0.143 (95% CrI 0.027 to 0.483)

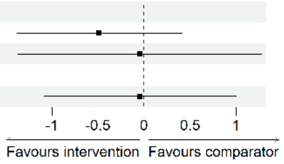

Figure 13 Estimated effects on fatigue outcomes of interventions within the Kidney-specific network, relative to wait list, at end of treatment, with 95% credible intervals (CrI) and 95% predictive intervals (PrI). The number of participants (n) and the number of studies (N studies) are given for context. Broad intervention categorisation is also presented to aid interpretation (Behavioural, Stimulation, Nutritional, and Other).

Finally, the analysis of Stroke-related conditions had again less than 5 studies informing the network and thus the informative prior was used. Three studies provided evidence for CBT-fatigue, for which a statistically significant beneficial effect was identified relative to wait list control. A statistically significant, beneficial effect was also identified for "other psychological" interventions. Due to the low numbers of studies, these results should be interpreted with caution. The between study heterogeneity was again found to be moderate, with the standard deviation equal to 0.143 [95% CrI 0.027, 0.484].

Treatment	SMD	95% CrI	95% PrI	n	(N studies)
Behavioural					
CBT-fatigue	-1.77	-2.996 to -0.568	-3.070 to -0.491	86	(3)
Other psychological	-1.59	-2.950 to -0.244	-3.020 to -0.174	45	(1)
Other					
Info/ Education	-1.68	-3.059 to -0.319	-3.120 to -0.261	36	(1)

Heterogeneity: between-study SD is 0.143 (95% CrI 0.027 to 0.484)

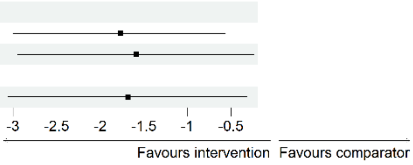

Figure 14 Estimated effects on fatigue outcomes of interventions within the Stroke-specific network, relative to wait list, at end of treatment, with 95% credible intervals

(CrI) and 95% predictive intervals (PrI). The number of participants (n) and the number of studies (N studies) are given for context. Broad intervention categorisation is also presented to aid interpretation (Behavioural, Stimulation, Nutritional, and Other).

Inconsistency was assessed for the EOT condition-specific networks with closed loops using the posterior mean residual deviances, followed by node-splitting, Figure 15. Following removal of Fleming (2021)[2] and Turner (2016)[5] from the EOT MS-specific network, no statistically significant inconsistency was detected in the EOT analyses.

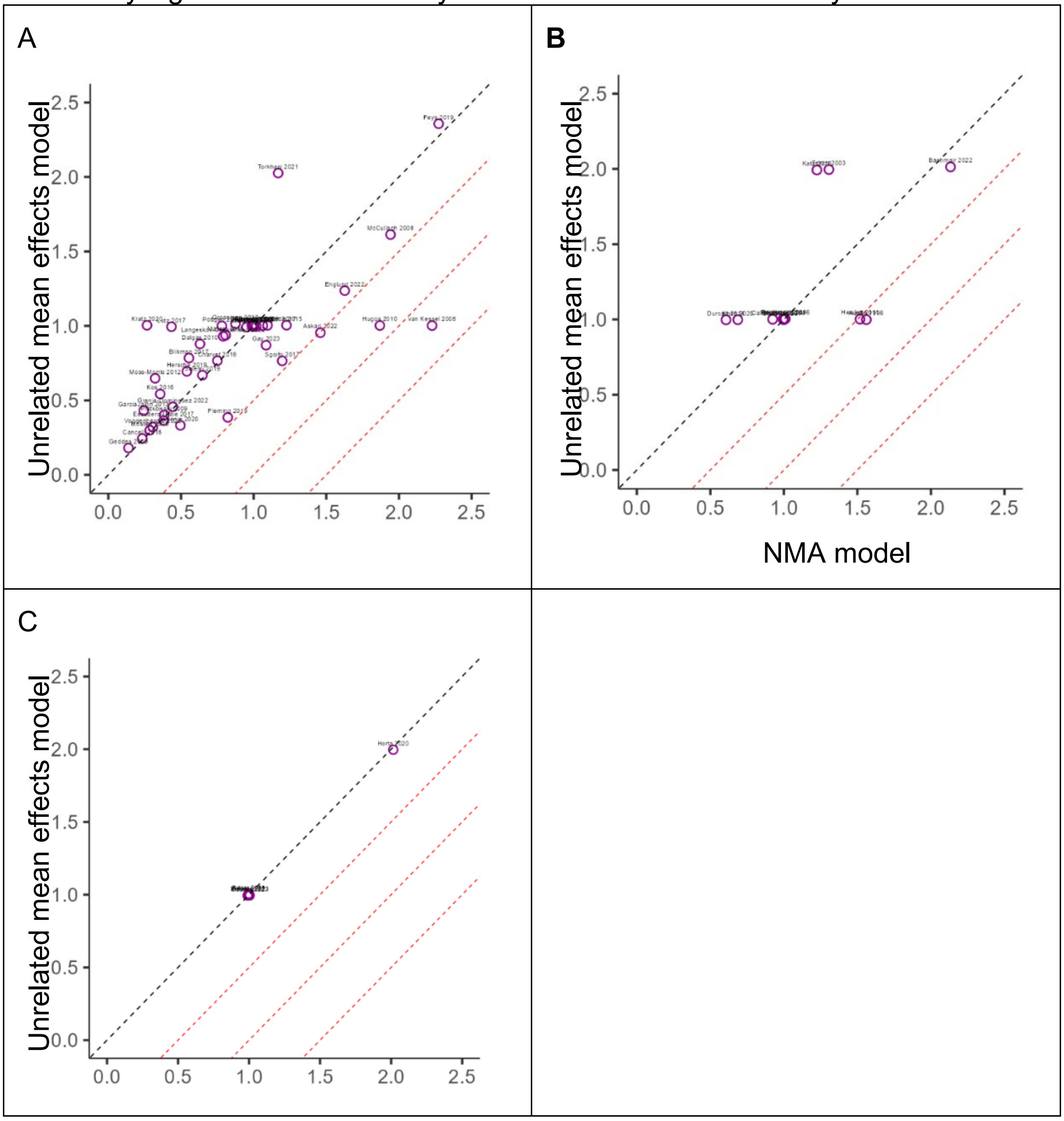

Figure 15 Mean posterior residual deviances according to the unrelated mean effects model versus the NMA model for A) MS, B) MSK #1, and C) IBD condition-specific analyses, at end of treatment, respectively. Black dashed line is given by $y = x$, red dashed lines represent contours separated by differences of 0.5 between the two models. Any studies below the first red dashed line indicative of potential inconsistency.

9.5.2 ST

For the ST condition-specific analysis, only one viable network could be constructed which summarised evidence for studies in MS, shown in Figure 16 A. This network included 12 interventions across 14 studies. The point estimates and 95% CrIs for the ST MS network are shown in Figure 16 B where the treatment effect is relative to usual care. Inconsistency

was assessed for the ST MS network via comparison of the posterior mean residual deviances from the unrelated mean effects model and NMA model, followed by node-splitting, Figure 16 C; no statistically significant inconsistency was detected.

In the ST MS-specific network, no treatment was identified to have a statistically significant effect on fatigue outcomes, however, none of the included treatments were found to be statistically significant in the ST primary analysis. Additionally, the evidence base of the MS-specific network is smaller than the evidence within the primary analysis (14 vs. 24 studies). In general, the treatment effects should be interpreted with caution as no intervention featured in more than 4 studies.

The between study heterogeneity standard deviation was 0.243 [95% CrI 0.01, 1.528] which indicates moderate heterogeneity within the network.

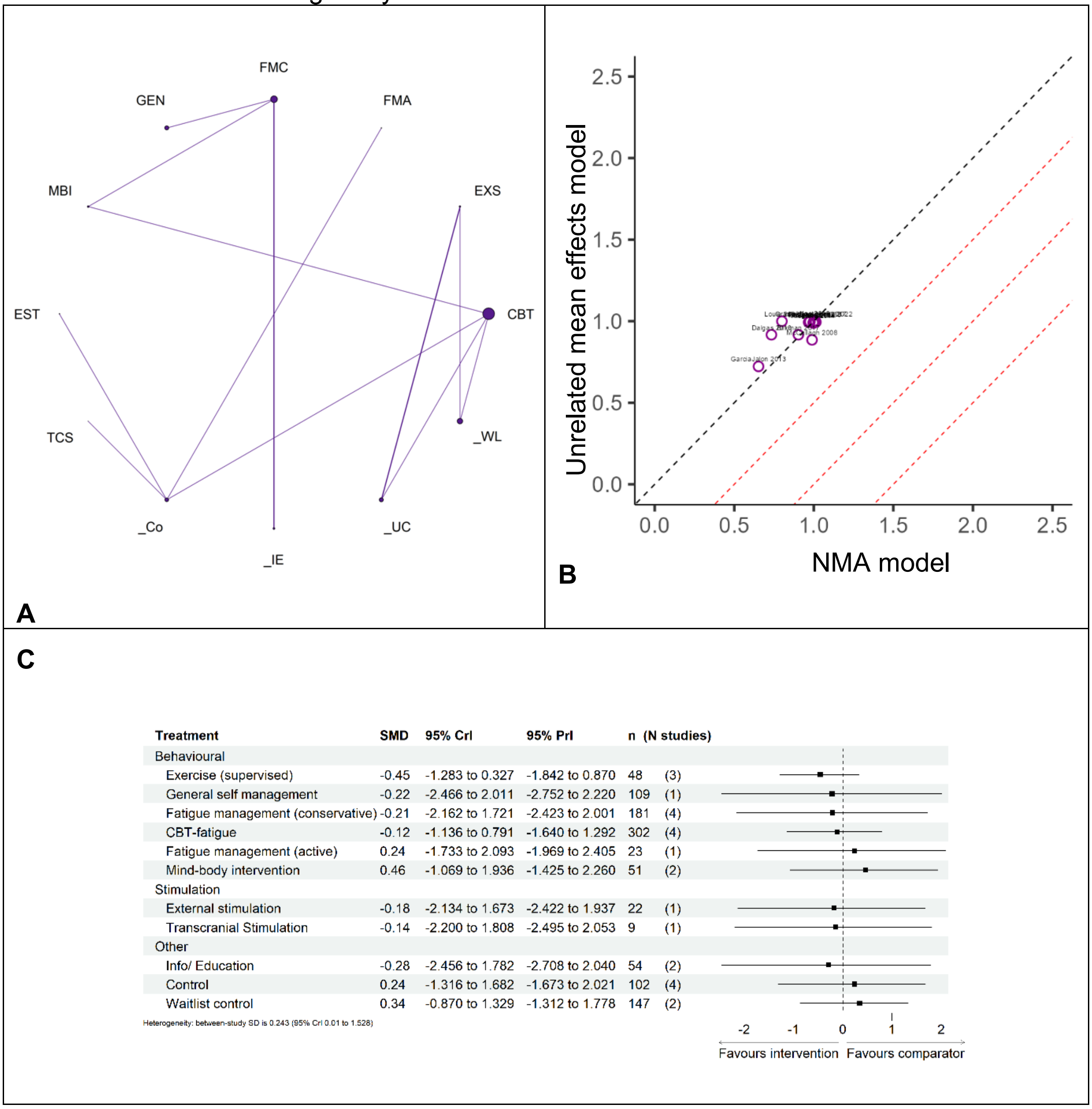

Figure 16 A) Network geometry, B) posterior residual deviances according to the unrelated mean effects model and the NMA model, and C) Estimated effects on fatigue outcomes of interventions, relative to usual care, with 95% credible intervals (CrI) and 95% predictive intervals (PrI); for the MS condition-specific analysis, at short term, respectively. The number of participants (n) and the number of studies (N studies) are given for context. The “control” node is displayed as this functioned to ensure

connectivity of the network, but this is not an active intervention for consideration/recommendation.

9.5.3 LT

For the LT condition-specific analysis, there were two viable networks relating to: MS, shown in Figure 17 A, and MSK, shown in Figure 18 A. These networks included: 9 interventions across 10 studies, and 4 interventions across 3 studies, respectively. The point estimates and 95% CrIs for the LT networks are shown in Figure 17 B and 18 B, respectively; the treatment effects are relative to usual care. Inconsistency was assessed for the LT networks by comparing the posterior mean residual deviances from the unrelated mean effects model and the NMA model, followed by node-splitting, Figure 17 C and 18 C; no statistically significant inconsistency was detected in either network.

In the LT MS-specific network, no treatment was shown to have a statistically significant effect on fatigue outcomes. In the LT primary analysis, mindfulness and CBT-fatigue were shown to have statistically significant, beneficial effects on fatigue outcomes, but this was not mirrored in the MS-specific analysis. As for the MS-specific EOT and ST networks, the evidence base is approximately half of the transdiagnostic case (10 vs. 18 studies). Though there is some consensus between treatment effects seen in the MS-specific network and the primary analysis, however the broadening of the 95% CrIs resulted in non-significance.

The between study heterogeneity standard deviation was 0.317 [95% CrI 0.015, 2.119], indicating moderate to high heterogeneity within the network.

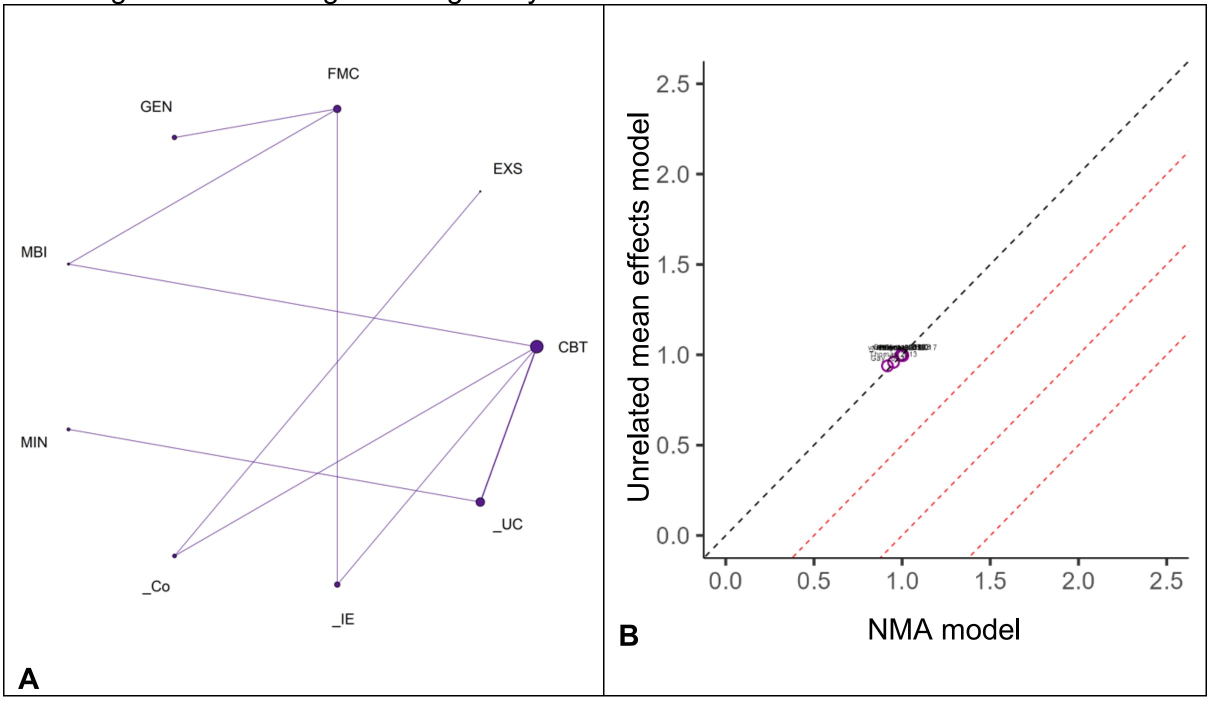

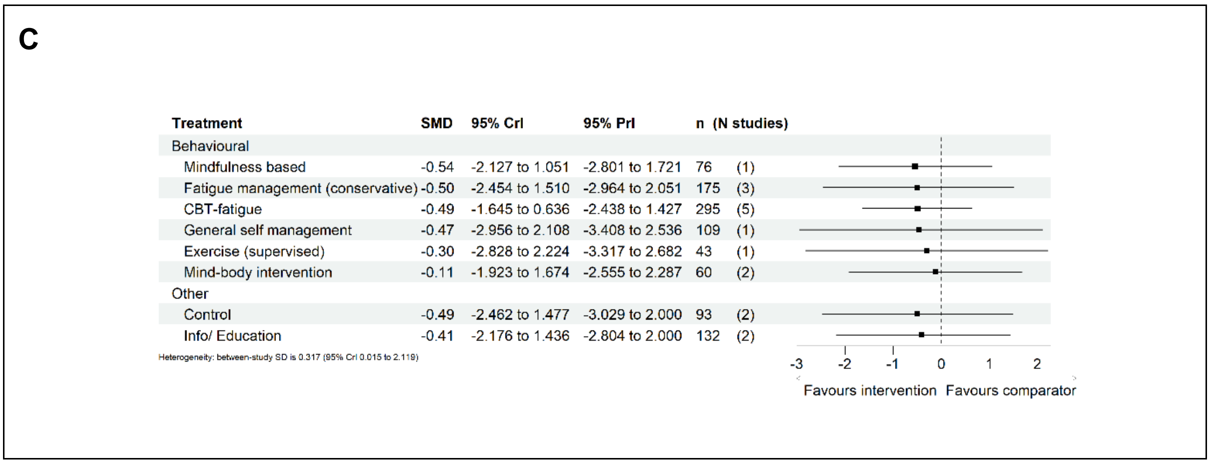

C

Treatment	SMD	95% CrI	95% PrI	n	(N studies)
Behavioural					
Mindfulness based	-0.54	-2.127 to 1.051	-2.801 to 1.721	76	(1)
Fatigue management (conservative)	-0.50	-2.454 to 1.510	-2.964 to 2.051	175	(3)
CBT-fatigue	-0.49	-1.645 to 0.636	-2.438 to 1.427	295	(5)
General self management	-0.47	-2.956 to 2.108	-3.408 to 2.536	109	(1)
Exercise (supervised)	-0.30	-2.828 to 2.224	-3.317 to 2.682	43	(1)
Mind-body intervention	-0.11	-1.923 to 1.674	-2.555 to 2.287	60	(2)
Other					
Control	-0.49	-2.462 to 1.477	-3.029 to 2.000	93	(2)
Info/ Education	-0.41	-2.176 to 1.436	-2.804 to 2.000	132	(2)

Heterogeneity: between-study SD is 0.317 (95% CrI 0.015 to 2.119)

Figure 17 A) Network geometry, B) posterior residual deviances according to the unrelated model effects model and the NMA model, and C) Estimated effects on fatigue outcomes of interventions, relative to usual care, with 95% credible intervals (CrI) and 95% predictive intervals (PrI); for the MS condition-specific analysis, at long term, respectively. The number of participants (n) and the number of studies (N studies) are given for context. The "control" node is displayed as this functioned to ensure connectivity of the network, but this is not an active intervention for consideration/recommendation.

In the LT MSK-specific network, two treatments were shown to have statistically significant treatment effects relative to usual care: physical activity promotion and CBT-fatigue. Exercise (supervised) was found not to be statistically significant – these results are consistent with the LT primary analysis.

The between study heterogeneity standard deviation was moderate, 0.121 [95% CrI 0.025, 0.435].

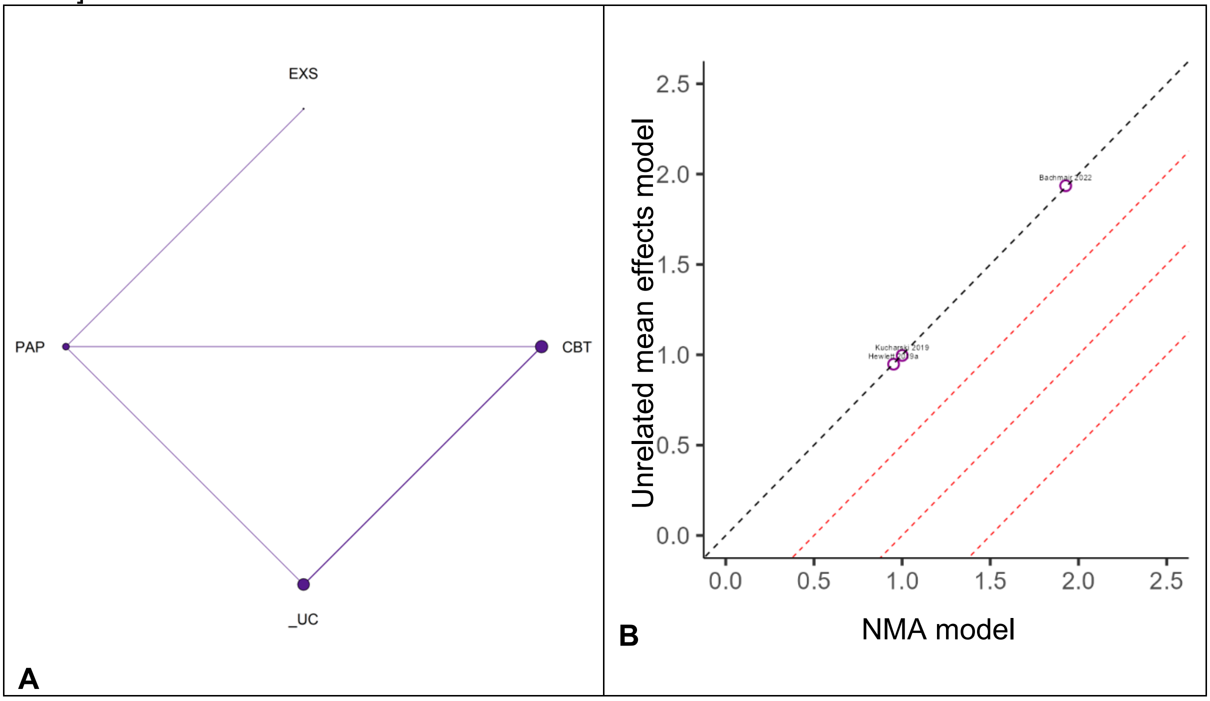

Treatment	SMD	95% CrI	95% PrI	n	(N studies)
Behavioural					
Exercise (supervised)	-0.54	-1.249 to 0.176	-1.348 to 0.268	36	(1)
Physical activity promotion	-0.53	-0.957 to -0.113	-1.103 to 0.039	159	(2)
CBT-fatigue	-0.35	-0.669 to -0.043	-0.857 to 0.141	292	(2)

Heterogeneity: between-study SD is 0.121 (95% CrI 0.025 to 0.435)

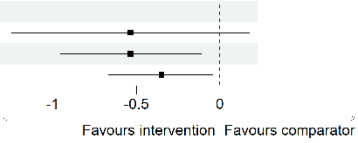

C

Figure 18 A) Network geometry, B) posterior residual deviances according to the unrelated mean effects model and the NMA model, and C) estimated effects on fatigue outcomes of interventions, relative to usual care, with 95% credible intervals (CrI) and 95% predictive intervals (PrI); for the MSK condition-specific analysis, at long term, respectively. The number of participants (n) and the number of studies (N studies) are given for context. The “control” node is displayed as this functioned to ensure connectivity of the network, but this is not an active intervention for consideration/recommendation.

9.6 NMA scenario analysis: exclusion of pilot and feasibility studies

The evidence base for the primary analysis consists of studies reporting results from RCTs as well as pilot and feasibility trials. To assess the potential impact of the inclusion of pilot and feasibility studies within the NMAs, we re-constructed the networks for EOT, ST and LT follow up, omitting any pilot or feasibility studies. This resulted in networks with 23, 13 and 12 connected interventions, informed by 65, 15 and 15 studies.

9.6.1 EOT

Generally, the NMA results when excluding pilot/feasibility studies were similar to the primary analysis. Four interventions were no longer included in the network; remote ischaemic conditioning, fish oil supplements, plant based supplements, and flavonoid (cocoa) derived supplements. In the primary analysis, acupuncture was shown to exhibit statistically significant beneficial effects for fatigue outcomes, this was however only directly informed by two studies. In this scenario analysis, one of these studies was excluded and the treatment effect of acupuncture was no longer shown to be statistically significant. Similarly, thiamine-based supplements in this scenario analysis were no longer found to be statistically significant. The between study variance was comparable between this analysis and the primary analysis. Other than the changes listed above, there was generally a minor impact on treatment effect estimates and the associated 95% CrIs at EOT.

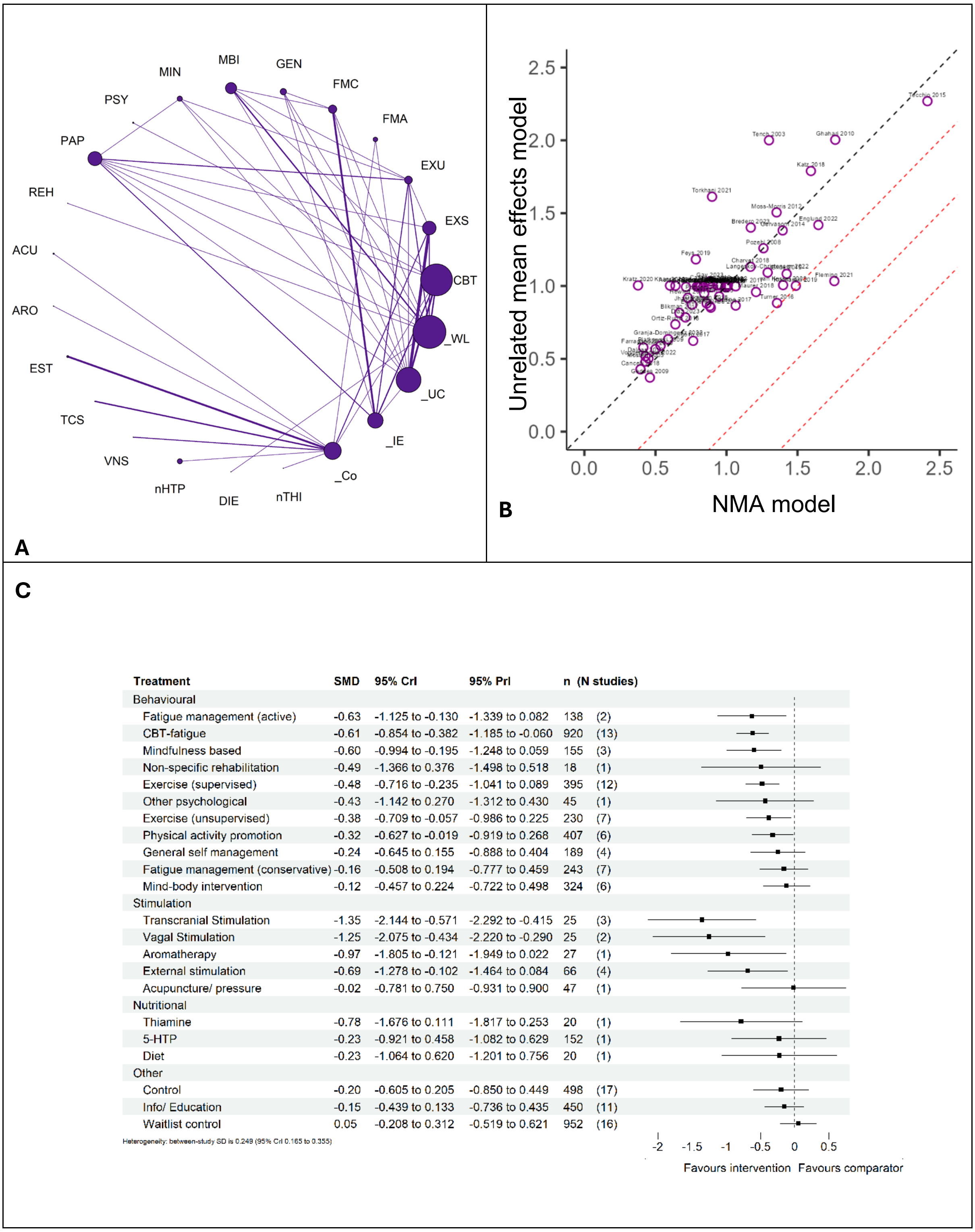

Treatment	SMD	95% CrI	95% PrI	n	(N studies)
Behavioural					
Fatigue management (active)	-0.63	-1.125 to -0.130	-1.339 to 0.082	138	(2)
CBT-fatigue	-0.61	-0.854 to -0.382	-1.185 to -0.060	920	(13)
Mindfulness based	-0.60	-0.994 to -0.195	-1.248 to 0.059	155	(3)
Non-specific rehabilitation	-0.49	-1.366 to 0.376	-1.498 to 0.518	18	(1)
Exercise (supervised)	-0.48	-0.716 to -0.235	-1.041 to 0.089	395	(12)
Other psychological	-0.43	-1.142 to 0.270	-1.312 to 0.430	45	(1)
Exercise (unsupervised)	-0.38	-0.709 to -0.057	-0.986 to 0.225	230	(7)
Physical activity promotion	-0.32	-0.627 to -0.019	-0.919 to 0.268	407	(6)
General self management	-0.24	-0.645 to 0.155	-0.888 to 0.404	189	(4)
Fatigue management (conservative)	-0.16	-0.508 to 0.194	-0.777 to 0.459	243	(7)
Mind-body intervention	-0.12	-0.457 to 0.224	-0.722 to 0.498	324	(6)
Stimulation					
Transcranial Stimulation	-1.35	-2.144 to -0.571	-2.292 to -0.415	25	(3)
Vagal Stimulation	-1.25	-2.075 to -0.434	-2.220 to -0.290	25	(2)
Aromatherapy	-0.97	-1.805 to -0.121	-1.949 to 0.022	27	(1)
External stimulation	-0.69	-1.278 to -0.102	-1.464 to 0.084	66	(4)
Acupuncture/ pressure	-0.02	-0.781 to 0.750	-0.931 to 0.900	47	(1)
Nutritional					
Thiamine	-0.78	-1.676 to 0.111	-1.817 to 0.253	20	(1)
5-HTP	-0.23	-0.921 to 0.458	-1.082 to 0.629	152	(1)
Diet	-0.23	-1.064 to 0.620	-1.201 to 0.756	20	(1)
Other					
Control	-0.20	-0.605 to 0.205	-0.850 to 0.449	498	(17)
Info/ Education	-0.15	-0.439 to 0.133	-0.736 to 0.435	450	(11)
Waitlist control	0.05	-0.208 to 0.312	-0.519 to 0.621	952	(16)

Figure 19 A) Network geometry, B) posterior residual deviances according to the unrelated mean effects model and NMA model, and C) estimated effects on fatigue outcomes of interventions, relative to usual care, with 95% credible intervals (CrI) and 95% predictive intervals; for the end of treatment analysis†, respectively. The number of participants (n) and the number of studies (N studies) are given for context. The "control" node is displayed as this functioned to ensure connectivity of the network, but this is not an active intervention for consideration/recommendation. †Data from pilot/feasibility studies were excluded in this analysis.

9.6.2 ST

The SMDs and 95% credible intervals were similar at ST follow-up when pilot and feasibility studies were excluded with the primary analysis, with no changes in statistical significance for the included interventions. Three interventions were however no longer included in the network including: remote ischaemic conditioning, transcranial stimulation, and acupuncture/pressure based interventions. Between study variance was comparable between the two analyses.

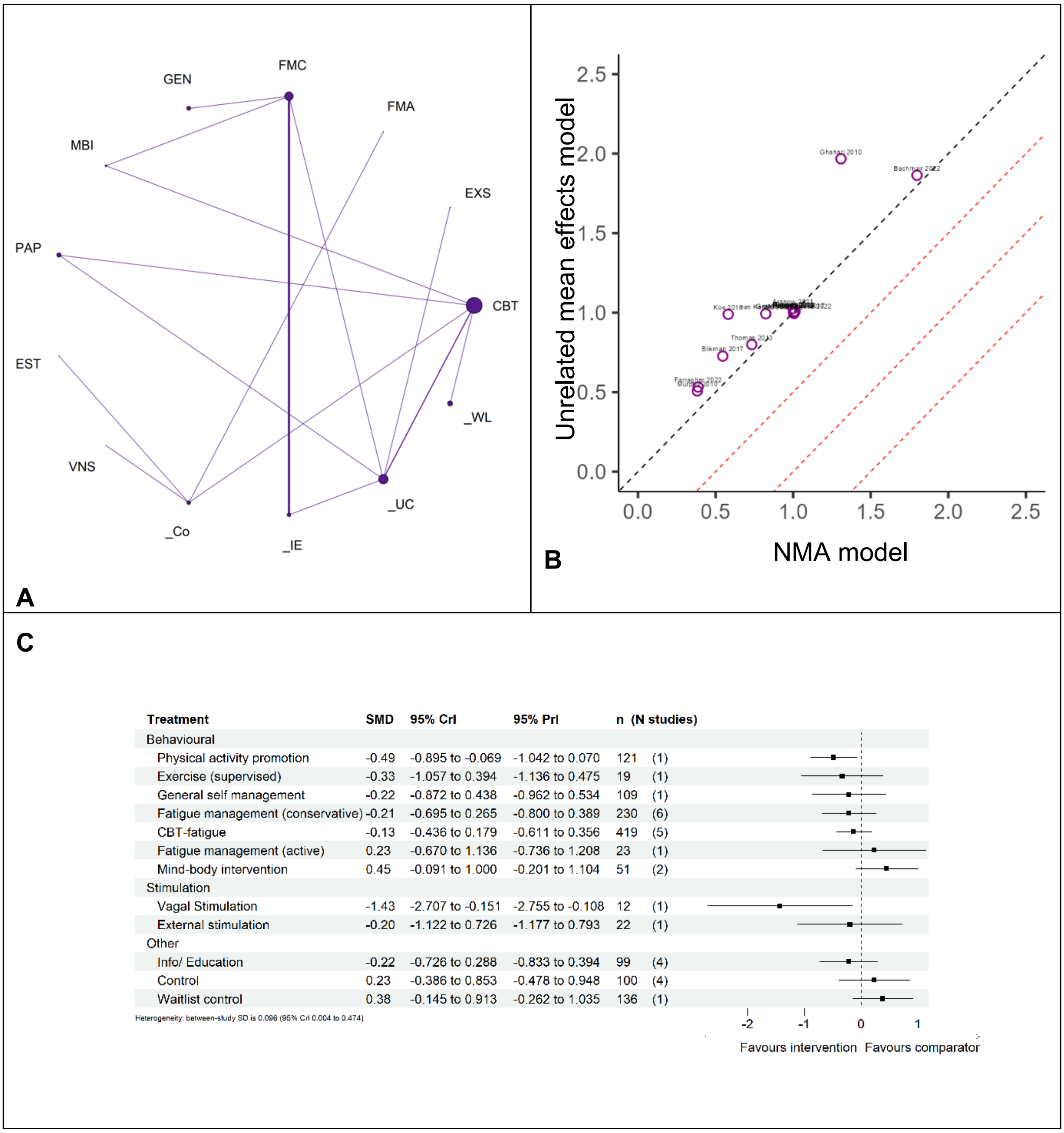

Figure 20 A) Network geometry, B) posterior residual deviances according to the unrelated mean effects model versus the NMA model, and C) estimated effects on fatigue outcomes of interventions, relative to usual care, with 95% credible intervals (CrI) and 95% predictive intervals (PrI); for the short term analysis†, respectively. The number of participants (n) and the number of studies (N studies) are given for context. The "control" node is displayed as this functioned to ensure connectivity of the network, but this is not an active intervention for consideration/recommendation. †Data from pilot/feasibility studies were excluded in this analysis.

9.6.3 LT

In the LT follow-up analysis, when pilot studies were not included, slightly broader 95% CrIs were evident for the majority of interventions compared to the primary analysis. Despite this, two interventions were found to exhibit statistically significant, beneficial effects for fatigue, which were not found to be statistically significant in the primary analysis, including: conservative fatigue management approaches, and general self management. Only one study directly evidencing conservative fatigue management was a pilot study in the primary analysis, and thus the changes in the scenario analysis results appear to be an indirect effect resulting from changes to other interventions within the network. Despite this, other intervention treatment effect point estimates, appear to be similar to the primary analysis. As in the EOT and ST follow-up analyses, remote ischaemic conditioning was no longer included within the network. As with the other time points, between study variance was comparable to that observed in the primary analyses.

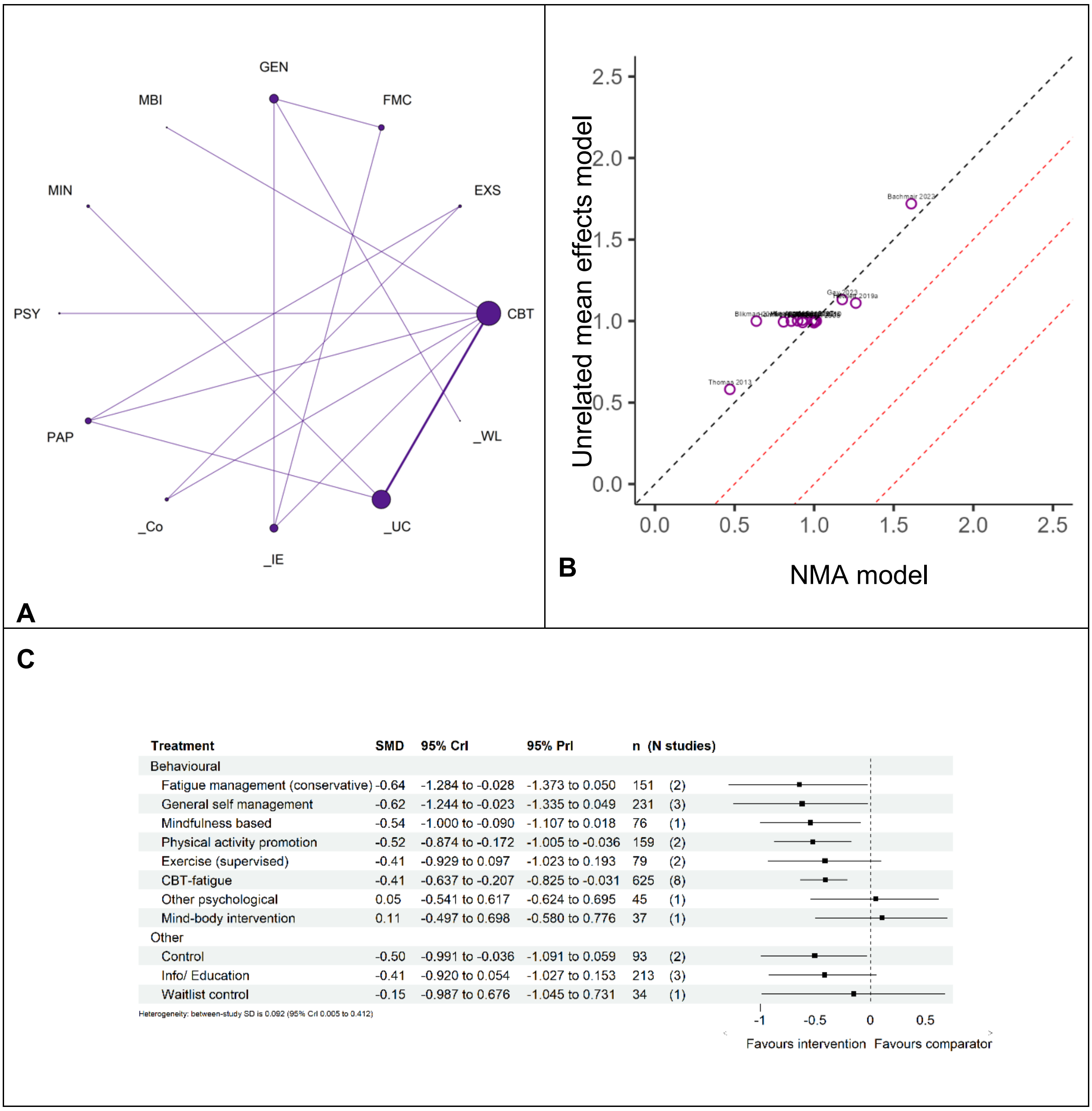

Figure 21 A) Network geometry, B) posterior residual deviances according to the unrelated mean effects model and the NMA model, and C) estimated effects on fatigue outcomes of interventions, relative to usual care, with 95% credible intervals (CrI) and 95% predictive intervals (PrI); for the long term analysis†, respectively. The number of

participants (n) and the number of studies (N studies) are given for context. The "control" node is displayed as this functioned to ensure connectivity of the network, but this is not an active intervention for consideration/recommendation. †Data from pilot/feasibility studies were excluded in this analysis.

9.7 References

1. Louie, J., Baquie, K., Offerman, J., Granger, C. L., Khan, F., & Bower, K. J. (2022). Maximising Abilities, Negotiating and Generating Exercise options (MANAGE) in people with multiple sclerosis: A feasibility randomised controlled trial. *Clinical Rehabilitation*, *36*(4), 498-510.
2. Fleming, K. M., Coote, S. B., & Herring, M. P. (2021). Home-based Pilates for symptoms of anxiety, depression and fatigue among persons with multiple sclerosis: An 8-week randomized controlled trial. *Multiple Sclerosis Journal*, *27*(14), 2267-2279.
3. Menting, J., Tack, C. J., van Bon, A. C., Jansen, H. J., van den Bergh, J. P., Mol, M. J., ... & Knoop, H. (2017). Web-based cognitive behavioural therapy blended with face-to-face sessions for chronic fatigue in type 1 diabetes: a multicentre randomised controlled trial. *The lancet Diabetes & endocrinology*, *5*(6), 448-456.
4. Langeskov-Christensen, M., Hvid, L. G., Jensen, H. B., Nielsen, H. H., Petersen, T., Stenager, E., & Dalgas, U. (2022). Efficacy of high-intensity aerobic exercise on common multiple sclerosis symptoms. *Acta Neurologica Scandinavica*, *145*(2), 229-238.
5. Turner, A. P., Hartoonian, N., Sloan, A. P., Benich, M., Kivlahan, D. R., Hughes, C., ... & Haselkorn, J. K. (2016). Improving fatigue and depression in individuals with multiple sclerosis using telephone-administered physical activity counseling. *Journal of Consulting and Clinical Psychology*, *84*(4), 297.
6. Horta, D., Lira, A., Sanchez-Lloansi, M., Villoria, A., Teggiachi, M., García-Rojo, D., ... & Calvet, X. (2020). A prospective pilot randomized study: electroacupuncture vs. sham procedure for the treatment of fatigue in patients with quiescent inflammatory bowel disease. *Inflammatory Bowel Diseases*, *26*(3), 484-492.
7. Clarke, A., Barker-Collo, S. L., & Feigin, V. L. (2012). Poststroke fatigue: does group education make a difference? A randomized pilot trial. *Topics in stroke rehabilitation*, *19*(1), 32-39.
8. Artom, M., Czuber-Dochan, W., Sturt, J., Proudfoot, H., Roberts, D., & Norton, C. (2019). Cognitive-behavioural therapy for the management of inflammatory bowel disease-fatigue: a feasibility randomised controlled trial. *Pilot and feasibility studies*, *5*(1), 1-20.
9. Ehde, D. M., Arewasikporn, A., Alschuler, K. N., Hughes, A. J., & Turner, A. P. (2018). Moderators of treatment outcomes after telehealth self-management and education in adults with multiple sclerosis: a secondary analysis of a randomized controlled trial. *Archives of physical medicine and rehabilitation*, *99*(7), 1265-1272.
10. Gay, M. C., Cassedanne, F., Barbot, F., Vaugier, I., Thomas, S., Manchon, E., ... & Heinzlef, O. (2024). Long-term effectiveness of a cognitive behavioural therapy (CBT) in the management of fatigue in patients with relapsing remitting multiple sclerosis (RRMS): a multicentre, randomised, open-label, controlled trial versus standard care. *Journal of Neurology, Neurosurgery & Psychiatry*, *95*(2), 158-166.

11. Hammond, A., Bryan, J., & Hardy, A. (2008). Effects of a modular behavioural arthritis education programme: a pragmatic parallel-group randomized controlled trial. *Rheumatology*, *47*(11), 1712-1718.
12. Hewlett, S., Almeida, C., Ambler, N., Blair, P. S., Choy, E. H., Dures, E., ... & RAFT Study Group. (2019). Reducing arthritis fatigue impact: two-year randomised controlled trial of cognitive behavioural approaches by rheumatology teams (RAFT). *Annals of the rheumatic diseases*, *78*(4), 465-472.
