# Supplementary material for: Effectiveness of non-pharmacological interventions for fatigue in long term conditions: systematic review and network meta-analysis
Source: BMJ Med. 2026 Mar 13;5(1):e001746. doi: 10.1136/bmjmed-2025-001746 (PMC12993367; doi:10.1136/bmjmed-2025-001746)
Supplement: online supplemental file 2 [file bmjmed-5-1-s002.pdf]

Effectiveness of non-pharmacological interventions for fatigue in long term conditions: systematic review and network meta-analysis

Supplementary Methods

Table of Contents

1 SUPPLEMENTARY METHODS 1: DETAILED SEARCH STRATEGY 3

2 SUPPLEMENTARY METHODS 2 RISK OF BIAS ASSESSMENT 25

2.1 DETAILED DESCRIPTION OF METHODS 25
2.2 ADAPTED RISK OF BIAS 2 ASSESSMENT CRITERIA 27

3 SUPPLEMENTARY METHODS 3 GRADE CLASSIFICATION 29

3.1 ADAPTED GRADE METHODS. 29

4 SUPPLEMENTARY METHODS 4 : INTERVENTION CLASSIFICATION CRITERIA 33

4.1 PHYSICAL ACTIVITY-ORIENTED INTERVENTIONS 33
4.1.1 EXERCISE SUPERVISED. 33
4.1.2 EXERCISE UNSUPERVISED. 33
4.1.3 PHYSICAL ACTIVITY PROMOTION 33
4.1.4 ACTIVE RECREATIONAL 33
4.2 SELF-MANAGEMENT INTERVENTIONS 33
4.2.1 CBT-FATIGUE 33
4.2.2 FATIGUE MANAGEMENT- ACTIVE 33
4.2.3 FATIGUE MANAGEMENT – CONSERVATIVE 33
4.2.4 GENERAL SELF-MANAGEMENT 34
4.2.5 NON-SPECIFIC REHABILITATION 34
4.3 MIND / MIND-BODY INTERVENTIONS 34
4.3.1 MIND-BODY 34
4.3.2 MINDFULNESS-BASED 34
4.3.3 PSYCHOSOCIAL ADAPTATION TO CONDITION 34
4.3.4 OTHER PSYCHOLOGICAL THERAPY 34
4.4 STIMULATION 34
4.4.1 NON-INVASIVE NEUROSTIMULATION 34
4.4.2 EXTERNAL STIMULATION 34
4.4.3 AROMATHERAPY 34
4.4.4 TOUCH-BASED 35
4.4.5 ACUPUNCTURE/PRESSURE 35
4.5 NUTRITIONAL INTERVENTIONS 35
4.5.1 PLANT BASED 35
4.5.2 NUTRITIONAL SUPPLEMENT 35

4.5.3 DIET 35
4.6 CONTROL DEFINITIONS 35
4.6.1 INFORMATION/EDUCATION 35
4.6.2 USUAL CARE 35
4.6.3 WAIT LIST 35
4.6.4 CONTROL 35

5 SUPPLEMENTARY METHODS 5: FOCUS GROUPS 36

5.1 PATIENT FOCUS GROUPS 36
5.1.1 PARTICIPANTS AND RECRUITMENT 36
5.1.2 FOCUS GROUPS 36
5.2 PARTICIPANT CHARACTERISTICS 36
5.3 FOCUS GROUP FINDINGS INFORMING THE CLINICAL EFFECTIVENESS ANALYSIS 36

6 SUPPLEMENTARY METHODS 6 - ADDITIONAL STATISTICAL ANALYSIS METHODS 38

6.1 MULTIPLE FATIGUE MEASURES 38
6.2 EVALUATION OF THE SMD 38
6.3 STATISTICAL MODEL FOR THE NMA 39
6.4 DEFINITION OF PRIORS 40
6.5 IMPLEMENTATION 40
6.6 REFERENCES 40

1 Supplementary Methods 1: Detailed search strategy

Search Strategies: RCT search
Ovid MEDLINE(R) ALL <1946 to September 27, 2023>

1 exp Chronic Disease/ 625076
2 ((chronic or long-term or long term) adj (condition* or disease* or illness*)).ti,ab. 129174
3 chronically ill.ti,ab. 6151
4 exp Rheumatic Diseases/ 261192
5 rheumati*.ti,ab. 63183
6 exp Diabetes Mellitus/ 511411
7 diabet*.ti,ab. 776423
8 exp Endocrine System Diseases/ 1133836
9 exp Thyroid Diseases/ 163788
10 exp Adrenal Gland Diseases/ or exp Adrenal Insufficiency/ 72739
11 exp Autoimmune Diseases/ 546702
12 ((endocrine or thyroid or adrenal or autoimmune or auto-immune or auto immune) adj1 (disorder* or disease* or condition*)).ti,ab. 122720
13 adrenal insufficiency.ti,ab. 7311
14 exp Heart Failure/ 148655
15 heart failure*.ti,ab. 209132
16 exp Coronary Disease/ 236955
17 coronary heart disease*.ti,ab. 55465
18 exp Renal Insufficiency, Chronic/ 135178
19 exp Kidney Failure, Chronic/ 101277
20 (chronic adj (renal or kidney) adj (insuffucien* or failure* or disease*)).ti,ab. 98172
21 exp Renal Dialysis/ 126720
22 dialysis.ti,ab. 122294
23 exp Transplants/ 31895
24 (transplant* adj3 (heart* or kidney* or liver* or lung*)).ti,ab. 180737
25 exp Multiple Sclerosis/ 70469
26 multiple sclerosis.ti,ab. 90255
27 exp Stroke/ 174658
28 stroke.ti,ab. 305418
29 exp Neurodegenerative Diseases/ 371816
30 ((neurodegenerative or neuro-degenerative or neuro degenerative) adj (disease* or disorder* or condition*)).ti,ab. 99788
31 exp Parkinson Disease/ 82813
32 (parkinson* adj disease).ti,ab. 116256
33 exp Arthritis, Rheumatoid/ 126357
34 rheumatoid arthritis.ti,ab. 120734
35 exp Osteoarthritis/ 78077
36 osteoarthritis.ti,ab. 84060
37 exp Lupus Erythematosus, Systemic/ 67452
38 lupus.ti,ab. 88490
39 exp Scleroderma, Systemic/ 23231
40 (systemic sclerosis or scleroderma).ti,ab. 28912
41 exp Inflammatory Bowel Diseases/ 97805
42 (inflammatory bowel disease* or IBD).ti,ab. 67194
43 exp Liver Cirrhosis, Biliary/ 8772
44 (primary biliary cirrhosis or PBS).ti,ab. 36509
45 exp Cholangitis, Sclerosing/ 4703
46 sclerosing cholangiti*.ti,ab. 7289
47 exp Lung Diseases/ 1236542
48 ((lung or pulmonary) adj (disease* or disorder* or condition*)).ti,ab. 142936
49 exp Pulmonary Disease, Chronic Obstructive/ 67441
50 ((chronic obstructive adj (pulmonary or lung or airway) adj (disease* or obstruction*)) or (COPD or COAD)).ti,ab. 83179
51 exp Asthma/ 143083
52 (asthma or asthmatic).ti,ab. 176832

53 exp Muscular Diseases/ 196410
54 (((muscle or muscular or myopathic) adj (disorder* or disease* or condition*)) or (myopathy or myopathies)).ti,ab. 36194
55 exp Muscular Dystrophies/ 30020
56 (muscular dystroph* or myodystroph*).ti,ab. 27210
57 or/1-56 5558763
58 "Fatigue Questionnaire".ti,ab. 382
59 "Fatigue Severity Scale".ti,ab. 1808
60 "Multidimensional Assessment of Fatigue".ti,ab. 139
61 "Short Form-36 Vitality".ti,ab. 34
62 ("Functional Assessment of Chronic Illness Therapy Fatigue" or FACIT F).ti,ab. 633
63 "Brief Fatigue Inventory".ti,ab. 472
64 "Numerical Rating Scale Fatigue".ti,ab. 7
65 ("Visual Analog Scale Fatigue" or VAS F).ti,ab. 105
66 "Checklist Individual Strength".ti,ab. 323
67 "Chalder Fatigue Scale".ti,ab. 201
68 "Multidimensional Fatigue Inventory Scale".ti,ab. 8
69 "Piper Fatigue Scale".ti,ab. 268
70 (PROMIS-29 or PROMIS 29 or PROMIS29).ti,ab. 235
71 Pittsburgh Fatigability Scale.ti,ab. 32
72 Fatigue Descriptive Scale.ti,ab. 10
73 Modified Fatigue Impact Scale.ti,ab. 517
74 ("40-item Fatigue Impact Scale" or "40 item Fatigue Impact Scale").ti,ab. 4
75 ("29-item Fatigue Assessment Instrument" or "29 item Fatigue Assessment Instrument").ti,ab. 1
76 ("Functional Assessment of Multiple Sclerosis" or FAMS).ti,ab. 172
77 or/58-76 4995
78 *Fatigue/ 16397
79 (fatigue adj7 (scale* or subscale* or sub-scale* or questionnaire* or assessment* or inventor* or measure* or tool*)).ti,ab. 17355
80 (scale or subscale or sub-scale or questionnaire or assessment or inventory or measure or measurement).ti,ab. 3456677
81 (fatigability or fatigable).ti,ab. 3406
82 78 or 81 19535
83 80 and 82 6761
84 79 or 83 19584
85 77 or 84 20135
86 57 and 85 8442
87 exp randomized controlled trial/ 602157
88 controlled clinical trial.pt. 95425
89 randomized.ab. 618304
90 placebo.ab. 241721
91 clinical trials as topic/ 201321
92 randomly.ab. 417343
93 trial.ti. 293560
94 or/87-93 1550631
95 exp animals/ not humans/ 5158236
96 94 not 95 1427530
97 86 and 96 1938

Embase <1974 to 2023 Week 38>

1 *chronic disease/ 32846
2 ((chronic or long-term or long term) adj (condition* or disease* or illness*)).ti,ab. 179677
3 chronically ill.ti,ab. 7536
4 *rheumatic disease/ 32096
5 rheumati*.ti,ab. 80760
6 *diabetes mellitus/ 242661
7 diabet*.ti,ab. 1174089
8 *endocrine disease/ 7079

9	*thyroid disease/	13779
10	*adrenal disease/	2041
11	*adrenal insufficiency/	4392
12	*autoimmune disease/	35243
13	((endocrine or thyroid or adrenal or autoimmune or auto-immune or auto immune) adj1 (disorder* or disease* or condition*)).ti,ab.	182768
14	adrenal insufficiency.ti,ab.	10682
15	*heart failure/	126614
16	heart failure*.ti,ab.	350732
17	*coronary artery disease/	96075
18	coronary heart disease*.ti,ab.	75791
19	*chronic kidney failure/	61974
20	(chronic adj (renal or kidney) adj (insuffucien* or failure* or disease*)).ti,ab.	155133
21	*hemodialysis/	63019
22	dialysis.ti,ab.	181634
23	*transplantation/	64169
24	(transplant* adj3 (heart* or kidney* or liver* or lung*)).ti,ab.	304389
25	*multiple sclerosis/	102769
26	multiple sclerosis.ti,ab.	141053
27	*cerebrovascular accident/	105016
28	stroke.ti,ab.	488091
29	*degenerative disease/	18573
30	((neurodegenerative or neuro-degenerative or neuro degenerative) adj (disease* or disorder* or condition*)).ti,ab.	131356
31	*Parkinson disease/	121773
32	(parkinson* adj disease).ti,ab.	168860
33	*rheumatoid arthritis/	127396
34	rheumatoid arthritis.ti,ab.	180981
35	*osteoarthritis/	52154
36	osteoarthritis.ti,ab.	120269
37	*systemic lupus erythematosus/	63341
38	lupus.ti,ab.	126358
39	*systemic sclerosis/	22876
40	(systemic sclerosis or scleroderma).ti,ab.	44582
41	*inflammatory bowel disease/	27489
42	(inflammatory bowel disease* or IBD).ti,ab.	119395
43	*biliary cirrhosis/	2200
44	(primary biliary cirrhosis or PBS).ti,ab.	59132
45	*sclerosing cholangitis/	1978
46	sclerosing cholangiti*.ti,ab.	12483
47	*lung disease/	34450
48	((lung or pulmonary) adj (disease* or disorder* or condition*)).ti,ab.	217538
49	*chronic obstructive lung disease/	82916
50	((chronic obstructive adj (pulmonary or lung or airway) adj (disease* or obstruction*)) or (COPD or COAD)).ti,ab.	143936
51	*asthma/	152110
52	(asthma or asthmatic).ti,ab.	258285
53	*muscle disease/	9985
54	(((muscle or muscular or myopathic) adj (disorder* or disease* or condition*)) or (myopathy or myopathies)).ti,ab.	51568
55	*muscular dystrophy/	9507
56	(muscular dystroph* or myodystroph*).ti,ab.	36327
57	or/1-56	4496914
58	"Fatigue Questionnaire".ti,ab.	625
59	"Fatigue Severity Scale".ti,ab.	3460
60	"Multidimensional Assessment of Fatigue".ti,ab.	269
61	"Short Form-36 Vitality".ti,ab.	39
62	("Functional Assessment of Chronic Illness Therapy Fatigue" or FACIT F).ti,ab.	1676
63	"Brief Fatigue Inventory".ti,ab.	881
64	"Numerical Rating Scale Fatigue".ti,ab.	11

65 ("Visual Analog Scale Fatigue" or VAS F).ti,ab. 163
66 "Checklist Individual Strength".ti,ab. 452
67 "Chalder Fatigue Scale".ti,ab. 310
68 "Multidimensional Fatigue Inventory Scale".ti,ab. 14
69 "Piper Fatigue Scale".ti,ab. 376
70 (PROMIS-29 or PROMIS 29 or PROMIS29).ti,ab. 617
71 Pittsburgh Fatigability Scale.ti,ab. 44
72 Fatigue Descriptive Scale.ti,ab. 17
73 Modified Fatigue Impact Scale.ti,ab. 1077
74 ("40-item Fatigue Impact Scale" or "40 item Fatigue Impact Scale").ti,ab. 4
75 ("29-item Fatigue Assessment Instrument" or "29 item Fatigue Assessment Instrument").ti,ab. 2
76 ("Functional Assessment of Multiple Sclerosis" or FAMS).ti,ab. 355
77 exp Fatigue Severity Scale/ or exp "Functional Assessment of Chronic Illness Therapy Fatigue Scale"/ or exp Multidimensional Fatigue Inventory/ or exp Chalder Fatigue Scale/ or exp Piper fatigue scale/ or exp "fatigue scale for motor and cognitive functions"/ or exp Fatigue Impact Scale/ 6541
78 or/58-77 12125
79 *fatigue/ 25859
80 (fatigue adj7 (scale* or subscale* or sub-scale* or questionnaire* or assessment* or inventor* or measure* or tool*)).ti,ab. 29052
81 (scale or subscale or sub-scale or questionnaire or assessment or inventory or measure or measurement).ti,ab. 4694159
82 (fatigability or fatigable).ti,ab. 5144
83 79 or 82 30552
84 81 and 83 12023
85 80 or 84 32495
86 78 or 85 35039
87 57 and 86 12690
88 exp randomized controlled trial/ 785235
89 controlled clinical trial/ 470992
90 random$.ti,ab. 1975440
91 randomization/ 98376
92 intermethod comparison/ 300743
93 placebo.ti,ab. 365413
94 (compare or compared or comparison).ti,ab. 7636197
95 ((evaluated or evaluate or evaluating or assessed or assess) and (compare or compared or comparing or comparison)).ab. 2778695
96 (open adj label).ti,ab. 108778
97 ((double or single or doubly or singly) adj (blind or blinded or blindly)).ti,ab. 273962
98 double blind procedure/ 210736
99 parallel group$1.ti,ab. 32147
100 (crossover or cross over).ti,ab. 124632
101 ((assign$ or match or matched or allocation) adj5 (alternate or group$1 or intervention$1 or patient$1 or subject$1 or participant$1)).ti,ab. 415356
102 (assigned or allocated).ti,ab. 490792
103 (controlled adj7 (study or design or trial)).ti,ab. 450700
104 (volunteer or volunteers).ti,ab. 282605
105 human experiment/ 642664
106 trial.ti. 401617
107 or/88-106 10036592
108 (random$ adj sampl$ adj7 ("cross section$" or questionnaire$1 or survey$ or database$1)).ti,ab. not (comparative study/ or controlled study/ or randomi?ed controlled.ti,ab. or randomly assigned.ti,ab.) 9609
109 cross-sectional study/ not (exp randomized controlled trial/ or controlled clinical trial/ or controlled study/ or randomi?ed controlled.ti,ab. or control group$1.ti,ab.) 361562search
110 (((case adj control$) and random$) not randomi?ed controlled).ti,ab. 21555
111 systematic review.ti,ab. not (trial or study).ti. 325581
112 (nonrandom$ not random$).ti,ab. 18945
113 "random field$".ti,ab. 2966

114 (random cluster adj3 sampl$).ti,ab. 1583
115 (review.ab. and review.pt.) not trial.ti. 1131497
116 "we searched".ab. and (review.ti. or review.pt.) 49364
117 "update review".ab. 136
118 (databases adj4 searched).ab. 62550
119 (rat or rats or mouse or mice or swine or porcine or murine or sheep or lambs or pigs or piglets or rabbit or rabbits or cat or cats or dog or dogs or cattle or bovine or monkey or monkeys or trout or marmoset$1).ti. and animal experiment/ 1220906
120 animal experiment/ not (human experiment/ or human/) 2564767
121 or/108-120 4382762
122 107 not 121 8767282
123 87 and 122 6538
124 limit 123 to "remove medline records" 4030

CINAHL via EBSCO
Monday, October 02, 2023 4:18:56 PM
S1 (MH "Chronic Disease+")
S2 TI ((chronic or long-term or long term) N1 (condition* or disease* or illness*)) OR AB ((chronic or long-term or long term) N1 (condition* or disease* or illness*))
S3 TI chronically ill OR AB chronically ill
S4 (MH "Rheumatic Diseases+")
S5 TI rheumati* OR AB rheumati*
S6 (MH "Diabetes Mellitus+")
S7 TI diabet* OR AB diabet*
S8 (MH "Endocrine Diseases+")
S9 (MH "Thyroid Diseases+")
S10 (MH "Adrenal Gland Diseases+")
S11 (MH "Adrenal Insufficiency+")
S12 (MH "Autoimmune Diseases+")
S13 TI ((endocrine or thyroid or adrenal or autoimmune or auto-immune or auto immune) N1 (disorder* or disease* or condition*)) OR AB ((endocrine or thyroid or adrenal or autoimmune or auto-immune or auto immune) N1 (disorder* or disease* or condition*))
S14 TI adrenal insufficiency OR AB adrenal insufficiency
S15 (MH "Heart Failure+")
S16 TI heart failure* OR AB heart failure*
S17 (MH "Coronary Disease+")
S18 TI coronary heart disease* OR AB coronary heart disease*
S19 (MH "Renal Insufficiency, Chronic+")
S20 (MH "Kidney Failure, Chronic+")
S21 TI (chronic adj (renal or kidney) N1 (insuffucien* or failure* or disease*)) OR AB (chronic adj (renal or kidney) N1 (insuffucien* or failure* or disease*))
S22 (MH "Dialysis Patients")
S23 TI dialysis OR AB dialysis
S24 TI (transplant* N3 (heart* or kidney* or liver* or lung*)) OR AB (transplant* N3 (heart* or kidney* or liver* or lung*))
S25 (MH "Multiple Sclerosis+")
S26 TI multiple sclerosis OR AB multiple sclerosis
S27 (MH "Stroke+")
S28 TI stroke OR AB stroke
S29 (MH "Neurodegenerative Diseases+")
S30 TI ((neurodegenerative or neuro-degenerative or neuro degenerative) N1 (disease* or disorder* or condition*)) OR AB ((neurodegenerative or neuro-degenerative or neuro degenerative) N1 (disease* or disorder* or condition*))
S31 (MH "Parkinson Disease")
S32 TI (parkinson* N1 disease) OR AB (parkinson* N1 disease)
S33 (MH "Arthritis, Rheumatoid+")
S34 TI rheumatoid arthritis OR AB rheumatoid arthritis
S35 (MH "Osteoarthritis+")
S36 TI osteoarthritis OR AB osteoarthritis
S37 (MH "Lupus Erythematosus, Systemic+")

S38 TI lupus OR AB lupus
S39 (MH "Scleroderma, Systemic+")
S40 TI (systemic sclerosis or scleroderma) OR AB (systemic sclerosis or scleroderma)
S41 (MH "Inflammatory Bowel Diseases+")
S42 TI (inflammatory bowel disease* or IBD) OR AB (inflammatory bowel disease* or IBD)
S43 (MH "Liver Cirrhosis+")
S44 TI (primary biliary cirrhosis or PBS) OR AB (primary biliary cirrhosis or PBS)
S45 (MH "Cholangitis, Sclerosing")
S46 TI sclerosing cholangiti* OR AB sclerosing cholangiti*
S47 (MH "Lung Diseases+")
S48 TI ((lung or pulmonary) N1 (disease* or disorder* or condition*)) OR AB ((lung or pulmonary) N1 (disease* or disorder* or condition*))
S49 (MH "Pulmonary Disease, Chronic Obstructive+")
S50 TI ((chronic obstructive N1 (pulmonary or lung or airway) N1 (disease* or obstruction*)) or (COPD or COAD)) OR AB ((chronic obstructive N1 (pulmonary or lung or airway) N1 (disease* or obstruction*)) or (COPD or COAD))
S51 (MH "Asthma+")
S52 TI (asthma or asthmatic) OR AB (asthma or asthmatic)
S53 (MH "Muscular Diseases+")
S54 TI (((muscle or muscular or myopathic) ADJ1 (disorder* or disease* or condition*)) or (myopathy or myopathies)) OR AB (((muscle or muscular or myopathic) ADJ1 (disorder* or disease* or condition*)) or (myopathy or myopathies))
S55 (MH "Muscular Dystrophy+")
S56 TI (muscular dystroph* or myodystroph*) OR AB (muscular dystroph* or myodystroph*)
S57 S1 OR S2 OR S3 OR S4 OR S5 OR S6 OR S7 OR S8 OR S9 OR S10 OR S11 OR S12 OR S13 OR S14 OR S15 OR S16 OR S17 OR S18 OR S19 OR S20 OR S21 OR S22 OR S23 OR S24 OR S25 OR S26 OR S27 OR S28 OR S29 OR S30 OR S31 OR S32 OR S33 OR S34 OR S35 OR S36 OR S37 OR S38 OR S39 OR S40 OR S41 OR S42 OR S43 OR S44 OR S45 OR S46 OR S47 OR S48 OR S49 OR S50 OR S51 OR S52 OR S53 OR S54 OR S55 OR S56
S58 TI "Fatigue Questionnaire" OR AB "Fatigue Questionnaire"
S59 TI "Fatigue Severity Scale" OR AB "Fatigue Severity Scale"
S60 TI "Multidimensional Assessment of Fatigue" OR AB "Multidimensional Assessment of Fatigue"
S61 TI "Short Form-36 Vitality" OR AB "Short Form-36 Vitality"
S62 TI ("Functional Assessment of Chronic Illness Therapy Fatigue" or FACIT F) OR AB ("Functional Assessment of Chronic Illness Therapy Fatigue" or FACIT F)
S63 TI "Brief Fatigue Inventory" OR AB "Brief Fatigue Inventory"
S64 TI "Numerical Rating Scale Fatigue" OR AB "Numerical Rating Scale Fatigue"
S65 TI ("Visual Analog Scale Fatigue" or VAS F) OR AB ("Visual Analog Scale Fatigue" or VAS F)
S66 TI "Checklist Individual Strength" OR AB "Checklist Individual Strength"
S67 TI "Chalder Fatigue Scale" OR AB "Chalder Fatigue Scale"
S68 TI "Multidimensional Fatigue Inventory Scale" OR AB "Multidimensional Fatigue Inventory Scale"
S69 TI "Piper Fatigue Scale" OR AB "Piper Fatigue Scale"
S70 TI (PROMIS-29 or PROMIS 29 or PROMIS29) OR AB (PROMIS-29 or PROMIS 29 or PROMIS29)
S71 TI Pittsburgh Fatigability Scale OR AB Pittsburgh Fatigability Scale
S72 TI Fatigue Descriptive Scale OR AB Fatigue Descriptive Scale
S73 TI Modified Fatigue Impact Scale OR AB Modified Fatigue Impact Scale
S74 TI ("40-item Fatigue Impact Scale" or "40 item Fatigue Impact Scale") OR AB ("40-item Fatigue Impact Scale" or "40 item Fatigue Impact Scale")
S75 TI ("29-item Fatigue Assessment Instrument" or "29 item Fatigue Assessment Instrument") OR AB ("29-item Fatigue Assessment Instrument" or "29 item Fatigue Assessment Instrument")
S76 TI ("Functional Assessment of Multiple Sclerosis" or FAMS) OR AB ("Functional Assessment of Multiple Sclerosis" or FAMS)
S77 S58 OR S59 OR S60 OR S61 OR S62 OR S63 OR S64 OR S65 OR S66 OR S67 OR S68 OR S69 OR S70 OR S71 OR S72 OR S73 OR S74 OR S75 OR S76
S78 (MM "Fatigue")
S79 TI (fatigue N7 (scale* or subscale* or sub-scale* or questionnaire* or assessment* or inventor* or measure* or tool*)) OR AB (fatigue N7 (scale* or subscale* or sub-scale* or questionnaire* or assessment* or inventor* or measure* or tool*))

S80 TI (scale or subscale or sub-scale or questionnaire or assessment or inventory or measure or measurement) OR AB (scale or subscale or sub-scale or questionnaire or assessment or inventory or measure or measurement)
S81 TI (fatigability or fatigable) OR AB (fatigability or fatigable)
S82 S78 OR S81
S83 S80 AND S82
S84 S79 OR S83
S85 S77 OR S84
S86 S57 AND S85
S87 MH "Clinical Trials+"
S88 PT Clinical trial S89 TX clinic* n1 trial*
S90 TX ((singl* n1 blind*) or (singl* n1 mask*)) or TX ((doubl* n1 blind*) or (doubl* n1 mask*)) or TX ((tripl* n1 blind*) or (tripl* n1 mask*)) or TX ((trebl* n1 blind*) or (trebl* n1 mask*))
S91 TX randomi* control* trial*
S92 MH "Random Assignment" S93 TX random* allocat*
S94 TX placebo*
S95 MH "Placebos"
S96 MH "Quantitative Studies"
S97 TX allocat* random*
S98 S87 OR S88 OR S89 OR S90 OR S91 OR S92 OR S93 OR S94 OR S95 OR S96 OR S97
S99 S86 AND S98 Results 1905

APA PsycInfo <1806 to September Week 4 2023>
1 exp Chronic Illness/ 34600
2 ((chronic or long-term or long term) adj (condition* or disease* or illness*)).ti,ab. 29719
3 chronically ill.ti,ab. 3184
4 exp Rheumatoid Arthritis/ 2100
5 rheumati*.ti,ab. 1112
6 exp Diabetes Mellitus/ 10460
7 diabet*.ti,ab. 36523
8 exp Thyroid Disorders/ 1539
9 exp Adrenal Gland Disorders/ 422
10 exp Immunologic Disorders/ 53042
11 ((endocrine or thyroid or adrenal or autoimmune or auto-immune or auto immune) adj1 (disorder* or disease* or condition*)).ti,ab. 3899
12 adrenal insufficiency.ti,ab. 148
13 exp Heart Disorders/ 16450
14 heart failure*.ti,ab. 4536
15 exp Cardiovascular Disorders/ 72080
16 coronary heart disease*.ti,ab. 4403
17 exp Kidney Diseases/ 2674
18 (chronic adj (renal or kidney) adj (insuffucien* or failure* or disease*)).ti,ab. 1543
19 exp Dialysis/ 2247
20 dialysis.ti,ab. 2381
21 exp Organ Transplantation/ 5421
22 (transplant* adj3 (heart* or kidney* or liver* or lung*)).ti,ab. 2086
23 exp Multiple Sclerosis/ 14366
24 multiple sclerosis.ti,ab. 17136
25 exp Cerebrovascular Accidents/ 24837
26 stroke.ti,ab. 37683
27 exp Neurodegenerative Diseases/ 95903
28 ((neurodegenerative or neuro-degenerative or neuro degenerative) adj (disease* or disorder* or condition*)).ti,ab. 19229
29 (parkinson* adj disease).ti,ab. 32763
30 exp Rheumatoid Arthritis/ 2100
31 rheumatoid arthritis.ti,ab. 2806
32 exp Arthritis/ 4810
33 osteoarthritis.ti,ab. 2288
34 exp Lupus/ 883
35 lupus.ti,ab. 1629

36 (systemic sclerosis or scleroderma).ti,ab. 219
37 exp Colon Disorders/ 5096
38 (inflammatory bowel disease* or IBD).ti,ab. 1201
39 exp Liver Disorders/ 5073
40 (primary biliary cirrhosis or PBS).ti,ab. 1564
41 sclerosing cholangiti*.ti,ab. 19
42 exp Lung Disorders/ 5469
43 ((lung or pulmonary) adj (disease* or disorder* or condition*)).ti,ab. 3973
44 exp Chronic Obstructive Pulmonary Disease/ 1788
45 ((chronic obstructive adj (pulmonary or lung or airway) adj (disease* or obstruction*)) or (COPD or COAD)).ti,ab. 3014
46 exp Asthma/ 5266
47 (asthma or asthmatic).ti,ab. 8470
48 exp Muscular Disorders/10761
49 (((muscle or muscular or myopathic) adj (disorder* or disease* or condition*)) or (myopathy or myopathies)).ti,ab. 1828
50 exp Muscular Dystrophy/ 1524
51 (muscular dystroph* or myodystroph*).ti,ab. 1629
52 or/1-51 380568
53 "Fatigue Questionnaire".ti,ab. 128
54 "Multidimensional Assessment of Fatigue".ti,ab. 35
55 "Short Form-36 Vitality".ti,ab. 7
56 ("Functional Assessment of Chronic Illness Therapy Fatigue" or FACIT F).ti,ab. 75
57 "Brief Fatigue Inventory".ti,ab. 105
58 "Numerical Rating Scale Fatigue".ti,ab. 4
59 ("Visual Analog Scale Fatigue" or VAS F).ti,ab. 21
60 "Checklist Individual Strength".ti,ab. 110
61 "Chalder Fatigue Scale".ti,ab. 73
62 "Multidimensional Fatigue Inventory Scale".ti,ab. 0
63 "Piper Fatigue Scale".ti,ab. 74
64 (PROMIS-29 or PROMIS 29 or PROMIS29).ti,ab. 54
65 Pittsburgh Fatigability Scale.ti,ab. 8
66 Fatigue Descriptive Scale.ti,ab. 1
67 Modified Fatigue Impact Scale.ti,ab. 139
68 ("40-item Fatigue Impact Scale" or "40 item Fatigue Impact Scale").ti,ab. 2
69 ("29-item Fatigue Assessment Instrument" or "29 item Fatigue Assessment Instrument").ti,ab. 1
70 ("Functional Assessment of Multiple Sclerosis" or FAMS).ti,ab. 62
71 or/52-70 380976
72 exp Fatigue/ 11615
73 (fatigue adj7 (scale* or subscale* or sub-scale* or questionnaire* or assessment* or inventor* or measure* or tool*)).ti,ab. 5324
74 (scale or subscale or sub-scale or questionnaire or assessment or inventory or measure or measurement).ti,ab. 1060391
75 (fatigability or fatigable).ti,ab. 536
76 72 or 75 11998
77 74 and 76 4608
78 73 or 77 7340
79 52 and 78 2359
80 (double-blind or random: assigned or control).tw. 554265
81 79 and 80 497

Web of Science Core Collection (Science and Social Sciences Citation Indexes – SCI-EXPANDED, SSCI)
TS=(((chronic) NEAR/1 (condition* or disease* or illness*))) — 3,672,386
TS=(((long-term) NEAR/1 (condition* or disease* or illness*))) — 16,292
TS=(chronically ill) — 6,072
TS=(rheumati*) — 54,425
TS=(diabet*) — 879,022
TS=(((endocrine) NEAR/1 (disorder* or disease* or condition*))) — 8,914

TS=(((thyroid) NEAR/1 (disorder* or disease* or condition*))) — 20,043
TS=(((adrenal) NEAR/1 (disorder* or disease* or condition*))) — 1,557
TS=(((autoimmune) NEAR/1 (disorder* or disease* or condition*))) — 106,203
TS=(((auto-immune) NEAR/1 (disorder* or disease* or condition*))) — 1,931
TS=(((auto immune) NEAR/1 (disorder* or disease* or condition*))) — 3,507
TS=adrenal insufficiency — 8,383
TS=heart failure* — 326,475
TS=coronary heart disease* — 192,572
TS=(chronic NEAR/1 (renal or kidney) NEAR/1 (insuffucien* or failure* or disease*)) — 121,026
TS=dialysis — 131,347
TS=(transplant* NEAR/3 (heart* or kidney* or liver* or lung*)) — 257,475
TS=multiple sclerosis — 146,702
TS=stroke — 409,574
TS=((neurodegenerative) NEAR/1 (disease* or disorder* or condition*)) — 106,263
TS=((neuro-degenerative) NEAR/1 (disease* or disorder* or condition*)) — 786
TS=((neuro degenerative) NEAR/1 (disease* or disorder* or condition*)) — 996
TS=(parkinson* NEAR/1 disease) — 176,729
TS=rheumatoid arthritis — 192,358
TS=osteoarthritis — 111,937
TS=lupus — 123,376
TS=(systemic sclerosis or scleroderma) — 45,897
TS=(inflammatory bowel disease* or IBD) — 113,244
TS=(primary biliary cirrhosis or PBS) — 55,103
TS=sclerosing cholangiti* — 11,277
TS=((lung or pulmonary) NEAR/1 (disease* or disorder* or condition*)) — 167,964
TS=((chronic obstructive NEAR/1 (pulmonary or lung or airway) NEAR/1 (disease* or obstruction*)) or (COPD or COAD)) — 104,320
TS=(asthma or asthmatic) — 225,022
TS=(((muscle or muscular or myopathic) NEAR/1 (disorder* or disease* or condition*)) or (myopathy or myopathies)) — 49,529
TS=(muscular dystroph* or myodystroph*) — 38,613
#1 OR #2 OR #3 OR #4 OR #5 OR #6 OR #7 OR #8 OR #9 OR #10 OR #11 OR #12 OR #13 OR #14 OR #15 OR #16 OR #17 OR #18 OR #19 OR #20 OR #21 OR #22 OR #23 OR #24 OR #25 OR #26 OR #27 OR #28 OR #29 OR #30 OR #31 OR #32 OR #33 OR #34 OR #35 – 4,071,294
TS=("Fatigue Questionnaire") — 349
TS=("Fatigue Severity Scale") — 1,806
TS="Multidimensional Assessment of Fatigue" — 126
TS="Short Form-36 Vitality" — 34
TS=("Functional Assessment of Chronic Illness Therapy Fatigue" or FACIT F) — 598
TS="Brief Fatigue Inventory" — 438
TS=("Visual Analog Scale Fatigue" or VAS F) — 1,168
TS="Checklist Individual Strength" — 318
TS="Chalder Fatigue Scale" — 189
TS="Multidimensional Fatigue Inventory Scale" — 6
TS="Piper Fatigue Scale" — 234
TS=(PROMIS-29 or PROMIS 29 or PROMIS29) — 464
TS=Pittsburgh Fatigability Scale — 35
TS=Fatigue Descriptive Scale — 785
TS=Modified Fatigue Impact Scale — 852
TS=("40-item Fatigue Impact Scale" or "40 item Fatigue Impact Scale") — 4
TS=("29-item Fatigue Assessment Instrument" or "29 item Fatigue Assessment Instrument") — 1
TS=("Functional Assessment of Multiple Sclerosis" or FAMS) — 266
TS=(fatigue NEAR/7 (scale* or subscale* or sub-scale* or questionnaire* or assessment* or inventor* or measure* or tool*)) — 26,432
TS=(scale or subscale or sub-scale or questionnaire or assessment or inventory or measure or measurement) — 10,552,202
TS=(fatigue or fatigability or fatigable) — 260,526
#56 AND #57 — 7,113
#55 OR #58 — 93,806

#32 OR #33 OR #34 OR #35 OR #36 OR #37 OR #38 OR #39 OR #40 OR #41 OR #42 OR #43 OR #44 OR #45 OR #46 OR #47 OR #48 OR #49 OR #50 OR #51 OR #52 OR #53 OR #54 -7,673
TI=(randomi?ed controlled trial) — 153,872
#36 AND #60 AND #61 - 756

Cochrane
Date Run: 03/10/2023 15:24:43

ID	Search	Hits
#1	MeSH descriptor: [Chronic Disease] explode all trees	38848
#2	((chronic or long-term or long term) NEXT (condition* or disease* or illness*)):ti OR ((chronic or long-term or long term) NEXT (condition* or disease* or illness*)):ab	14796
#3	chronically ill:ti OR chronically ill:ab	553
#4	MeSH descriptor: [Rheumatic Diseases] explode all trees	21037
#5	rheumati*:ti OR rheumati*:ab	4521
#6	MeSH descriptor: [Diabetes Mellitus] explode all trees	46685
#7	diabet*:ti OR diabet*:ab	109423
#8	MeSH descriptor: [Endocrine System Diseases] explode all trees	61968
#9	MeSH descriptor: [Thyroid Diseases] explode all trees	2994
#10	MeSH descriptor: [Adrenal Gland Diseases] explode all trees	755
#11	MeSH descriptor: [Adrenal Insufficiency] explode all trees	327
#12	MeSH descriptor: [Autoimmune Diseases] explode all trees	25146
#13	((endocrine or thyroid or adrenal or autoimmune or auto-immune or auto immune) NEAR/1 (disorder* or disease* or condition*)):ti OR ((endocrine or thyroid or adrenal or autoimmune or auto-immune or auto immune) NEAR/1 (disorder* or disease* or condition*)):ab	6114
#14	adrenal insufficiency:ti OR adrenal insufficiency:ab	517
#15	MeSH descriptor: [Heart Failure] explode all trees	14623
#16	heart failure*:ti OR heart failure*:ab	38262
#17	MeSH descriptor: [Coronary Disease] explode all trees	18489
#18	coronary heart disease*:ti OR coronary heart disease*:ab	21575
#19	MeSH descriptor: [Renal Insufficiency, Chronic] explode all trees	8653
#20	MeSH descriptor: [Kidney Failure, Chronic] explode all trees	5550
#21	(chronic NEXT (renal or kidney) NEXT (insuffucien* or failure* or disease*)):ti OR (chronic NEXT (renal or kidney) NEXT (insuffucien* or failure* or disease*)):ab	12353
#22	MeSH descriptor: [Renal Dialysis] explode all trees	6578
#23	dialysis:ti OR dialysis:ab	13972
#24	MeSH descriptor: [Transplantation] explode all trees	16684
#25	(transplant* NEAR/3 (heart* or kidney* or liver* or lung*)):ti OR (transplant* NEAR/3 (heart* or kidney* or liver* or lung*)):ab	13405
#26	MeSH descriptor: [Multiple Sclerosis] explode all trees	5959
#27	multiple sclerosis:ti OR multiple sclerosis:ab	11695
#28	MeSH descriptor: [Stroke] explode all trees	15152
#29	stroke:ti OR stroke:ab	63149
#30	MeSH descriptor: [Neurodegenerative Diseases] explode all trees	14828
#31	((neurodegenerative or neuro-degenerative or neuro degenerative) NEXT (disease* or disorder* or condition*)):ti OR ((neurodegenerative or neuro-degenerative or neuro degenerative) NEXT (disease* or disorder* or condition*)):ab	2697
#32	MeSH descriptor: [Parkinson Disease] explode all trees	6233
#33	(parkinson* NEXT disease):ti OR (parkinson* NEXT disease):ab	11158
#34	MeSH descriptor: [Arthritis, Rheumatoid] explode all trees	7374
#35	rheumatoid arthritis:ti OR rheumatoid arthritis:ab	17496
#36	MeSH descriptor: [Osteoarthritis] explode all trees	10596
#37	osteoarthritis:ti OR osteoarthritis:ab	19163
#38	MeSH descriptor: [Lupus Erythematosus, Systemic] explode all trees	1448
#39	lupus:ti OR lupus:ab	3797
#40	MeSH descriptor: [Scleroderma, Systemic] explode all trees	731
#41	(systemic sclerosis or scleroderma):ti OR (systemic sclerosis or scleroderma):ab	2068
#42	MeSH descriptor: [Inflammatory Bowel Diseases] explode all trees	4872

#43 (inflammatory bowel disease* or IBD):ti OR (inflammatory bowel disease* or IBD):ab 4800
#44 MeSH descriptor: [Liver Cirrhosis, Biliary] explode all trees 368
#45 (primary biliary cirrhosis or PBS):ti OR (primary biliary cirrhosis or PBS):ab 1704
#46 MeSH descriptor: [Cholangitis, Sclerosing] explode all trees 135
#47 sclerosing cholangiti*:ti OR sclerosing cholangiti*:ab 365
#48 MeSH descriptor: [Lung Diseases] explode all trees 58875
#49 ((lung or pulmonary) NEXT (disease* or disorder* or condition*)):ti OR ((lung or pulmonary) NEXT (disease* or disorder* or condition*)):ab 20401
#50 MeSH descriptor: [Pulmonary Disease, Chronic Obstructive] explode all trees 7303
#51 ((chronic obstructive NEXT (pulmonary or lung or airway) NEXT (disease* or obstruction*)) or (COPD or COAD)):ti OR ((chronic obstructive NEXT (pulmonary or lung or airway) NEXT (disease* or obstruction*)) or (COPD or COAD)):ab 22726
#52 MeSH descriptor: [Asthma] explode all trees 15046
#53 (asthma or asthmatic):ti OR (asthma or asthmatic):ab 34442
#54 MeSH descriptor: [Muscular Diseases] explode all trees 11960
#55 (((muscle or muscular or myopathic) NEXT (disorder* or disease* or condition*)) or (myopathy or myopathies)):ti OR (((muscle or muscular or myopathic) NEXT (disorder* or disease* or condition*)) or (myopathy or myopathies)):ab 1361
#56 MeSH descriptor: [Muscular Dystrophies] explode all trees 595
#57 (muscular dystroph* or myodystroph*):ti OR (muscular dystroph* or myodystroph*):ab 1101
#58 {OR #1-#57} 494013
#59 "Fatigue Questionnaire":ti OR "Fatigue Questionnaire":ab 240
#60 "Fatigue Severity Scale":ti OR "Fatigue Severity Scale":ab 1152
#61 "Multidimensional Assessment of Fatigue":ti OR "Multidimensional Assessment of Fatigue":ab 56
#62 "Short Form-36 Vitality":ti OR "Short Form-36 Vitality":ab 8
#63 ("Functional Assessment of Chronic Illness Therapy Fatigue" or FACIT F):ti OR ("Functional Assessment of Chronic Illness Therapy Fatigue" or FACIT F):ab 954
#64 "Brief Fatigue Inventory":ti OR "Brief Fatigue Inventory":ab 412
#65 "Numerical Rating Scale Fatigue":ti OR "Numerical Rating Scale Fatigue":ab 4
#66 ("Visual Analog Scale Fatigue" or VAS F):ti OR ("Visual Analog Scale Fatigue" or VAS F):ab 2248
#67 "Checklist Individual Strength":ti OR "Checklist Individual Strength":ab 167
#68 "Chalder Fatigue Scale":ti OR "Chalder Fatigue Scale":ab 190
#69 "Multidimensional Fatigue Inventory Scale":ti OR "Multidimensional Fatigue Inventory Scale":ab 3
#70 "Piper Fatigue Scale":ti OR "Piper Fatigue Scale":ab 205
#71 (PROMIS-29 or PROMIS 29 or PROMIS29):ti OR (PROMIS-29 or PROMIS 29 or PROMIS29):ab 258
#72 Pittsburgh Fatigability Scale:ti OR Pittsburgh Fatigability Scale:ab 10
#73 Fatigue Descriptive Scale:ti OR Fatigue Descriptive Scale:ab 493
#74 Modified Fatigue Impact Scale:ti OR Modified Fatigue Impact Scale:ab 700
#75 ("40-item Fatigue Impact Scale" or "40 item Fatigue Impact Scale"):ti OR ("40-item Fatigue Impact Scale" or "40 item Fatigue Impact Scale"):ab 1
#76 ("29-item Fatigue Assessment Instrument" or "29 item Fatigue Assessment Instrument"):ti OR ("29-item Fatigue Assessment Instrument" or "29 item Fatigue Assessment Instrument"):ab 1
#77 ("Functional Assessment of Multiple Sclerosis" or FAMS):ti OR ("Functional Assessment of Multiple Sclerosis" or FAMS):ab 50
#78 {OR #59-#77} 6577
#79 MeSH descriptor: [Fatigue] this term only 8377
#80 (fatigue NEXT/7 (scale* or subscale* or sub-scale* or questionnaire* or assessment* or inventor* or measure* or tool*)):ti OR (fatigue NEXT/7 (scale* or subscale* or sub-scale* or questionnaire* or assessment* or inventor* or measure* or tool*)):ab 7123
#81 (scale or subscale or sub-scale or questionnaire or assessment or inventory or measure or measurement):ti OR (scale or subscale or sub-scale or questionnaire or assessment or inventory or measure or measurement):ab 512278
#82 (fatigability or fatigable):ti OR (fatigability or fatigable):ab 393
#83 #79 OR #82 8708

#84 #81 AND #83 3579
#85 #80 OR #84 9134
#86 #58 AND #85 3981

Search Strategies: Systematic Reviews search
Ovid MEDLINE(R) Epub Ahead of Print and In-Process, In-Data-Review & Other Non-Indexed Citations and Daily <November 27, 2024>
1 exp Chronic Disease/ 654158
2 ((chronic or long-term or long term) adj (condition* or disease* or illness*)).ti,ab. 141815
3 chronically ill.ti,ab. 6323
4 exp Rheumatic Diseases/ 272346
5 rheumati*.ti,ab. 66215
6 exp Diabetes Mellitus/ 538090
7 diabet*.ti,ab. 834172
8 exp Endocrine System Diseases/ 1181269
9 exp Thyroid Diseases/ 168992
10 exp Adrenal Gland Diseases/ or exp Adrenal Insufficiency/ 74467
11 exp Autoimmune Diseases/ 570667
12 ((endocrine or thyroid or adrenal or autoimmune or auto-immune or auto immune) adj1 (disorder* or disease* or condition*)).ti,ab. 132811
13 adrenal insufficiency.ti,ab. 7868
14 exp Heart Failure/ 157212
15 heart failure*.ti,ab. 226915
16 exp Coronary Disease/ 242628
17 coronary heart disease*.ti,ab. 57626
18 exp Renal Insufficiency, Chronic/ 141810
19 exp Kidney Failure, Chronic/ 103657
20 (chronic adj (renal or kidney) adj (insuffucien* or failure* or disease*)).ti,ab. 107957
21 exp Renal Dialysis/ 130524
22 dialysis.ti,ab. 127861
23 exp Transplants/ 33321
24 (transplant* adj3 (heart* or kidney* or liver* or lung*)).ti,ab. 191883
25 exp Multiple Sclerosis/ 73877
26 multiple sclerosis.ti,ab. 96133
27 exp Stroke/ 186713
28 stroke.ti,ab. 330970
29 exp Neurodegenerative Diseases/ 394228
30 ((neurodegenerative or neuro-degenerative or neuro degenerative) adj (disease* or disorder* or condition*)).ti,ab. 111431
31 exp Parkinson Disease/ 88202
32 (parkinson* adj disease).ti,ab. 126026
33 exp Arthritis, Rheumatoid/ 130626
34 rheumatoid arthritis.ti,ab. 126907
35 exp Osteoarthritis/ 82888
36 osteoarthritis.ti,ab. 92680
37 exp Lupus Erythematosus, Systemic/ 69932
38 lupus.ti,ab. 93222
39 exp Scleroderma, Systemic/ 24020
40 (systemic sclerosis or scleroderma).ti,ab. 30500
41 exp Inflammatory Bowel Diseases/ 103655
42 (inflammatory bowel disease* or IBD).ti,ab. 73818
43 exp Liver Cirrhosis, Biliary/ 9010
44 (primary biliary cirrhosis or PBS).ti,ab. 38807
45 exp Cholangitis, Sclerosing/ 4920
46 sclerosing cholangiti*.ti,ab. 7766
47 exp Lung Diseases/ 1312727
48 ((lung or pulmonary) adj (disease* or disorder* or condition*)).ti,ab. 154066
49 exp Pulmonary Disease, Chronic Obstructive/ 70946
50 ((chronic obstructive adj (pulmonary or lung or airway) adj (disease* or obstruction*)) or (COPD or COAD)).ti,ab. 89722

51 exp Asthma/ 147301
52 (asthma or asthmatic).ti,ab. 184955
53 exp Muscular Diseases/ 203180
54 (((muscle or muscular or myopathic) adj (disorder* or disease* or condition*)) or (myopathy or myopathies)).ti,ab. 38290
55 exp Muscular Dystrophies/ 30943
56 (muscular dystroph* or myodystroph*).ti,ab. 28443
57 or/1-56 5874536
58 "Fatigue Questionnaire".ti,ab. 421
59 "Fatigue Severity Scale".ti,ab. 2067
60 "Multidimensional Assessment of Fatigue".ti,ab. 144
61 "Short Form-36 Vitality".ti,ab. 34
62 ("Functional Assessment of Chronic Illness Therapy Fatigue" or FACIT F).ti,ab. 752
63 "Brief Fatigue Inventory".ti,ab. 517
64 "Numerical Rating Scale Fatigue".ti,ab. 7
65 ("Visual Analog Scale Fatigue" or VAS F).ti,ab. 120
66 "Checklist Individual Strength".ti,ab. 352
67 "Chalder Fatigue Scale".ti,ab. 240
68 "Multidimensional Fatigue Inventory Scale".ti,ab. 10
69 "Piper Fatigue Scale".ti,ab. 289
70 (PROMIS-29 or PROMIS 29 or PROMIS29).ti,ab. 335
71 Pittsburgh Fatigability Scale.ti,ab. 43
72 Fatigue Descriptive Scale.ti,ab. 11
73 Modified Fatigue Impact Scale.ti,ab. 606
74 ("40-item Fatigue Impact Scale" or "40 item Fatigue Impact Scale").ti,ab. 4
75 ("29-item Fatigue Assessment Instrument" or "29 item Fatigue Assessment Instrument").ti,ab. 1
76 ("Functional Assessment of Multiple Sclerosis" or FAMS).ti,ab. 193
77 or/58-76 5751
78 *Fatigue/ 17037
79 (fatigue adj7 (scale* or subscale* or sub-scale* or questionnaire* or assessment* or inventor* or measure* or tool*)).ti,ab. 19310
80 (scale or subscale or sub-scale or questionnaire or assessment or inventory or measure or measurement).ti,ab. 3753921
81 (fatigability or fatigable).ti,ab. 3630
82 78 or 81 20385
83 80 and 82 7203
84 79 or 83 21678
85 77 or 84 22367
86 57 and 85 9455
87 (MEDLINE or systematic review).tw. or meta analysis.pt. 500343
88 86 and 87 325

Embase <1974 to 2024 Week 47>

1 *chronic disease/ 34954
2 ((chronic or long-term or long term) adj (condition* or disease* or illness*)).ti,ab. 196339
3 chronically ill.ti,ab. 7801
4 *rheumatic disease/ 33333
5 rheumati*.ti,ab. 86414
6 *diabetes mellitus/ 255008
7 diabet*.ti,ab. 1266395
8 *endocrine disease/ 7508
9 *thyroid disease/ 14564
10 *adrenal disease/ 2112
11 *adrenal insufficiency/ 4871
12 *autoimmune disease/ 37088
13 ((endocrine or thyroid or adrenal or autoimmune or auto-immune or auto immune) adj1 (disorder* or disease* or condition*)).ti,ab. 199767
14 adrenal insufficiency.ti,ab. 12291

15 *heart failure/ 136001
16 heart failure*.ti,ab. 381715
17 *coronary artery disease/ 100373
18 coronary heart disease*.ti,ab. 79147
19 *chronic kidney failure/ 69844
20 (chronic adj (renal or kidney) adj (insuffucien* or failure* or disease*)).ti,ab. 172600
21 *hemodialysis/ 67261
22 dialysis.ti,ab. 193744
23 *transplantation/ 64725
24 (transplant* adj3 (heart* or kidney* or liver* or lung*)).ti,ab. 329022
25 *multiple sclerosis/ 108475
26 multiple sclerosis.ti,ab. 149971
27 *cerebrovascular accident/ 115826
28 stroke.ti,ab. 529463
29 *degenerative disease/ 20667
30 ((neurodegenerative or neuro-degenerative or neuro degenerative) adj (disease* or disorder* or condition*)).ti,ab. 145110
31 *Parkinson disease/ 129695
32 (parkinson* adj disease).ti,ab. 181036
33 *rheumatoid arthritis/ 133978
34 rheumatoid arthritis.ti,ab. 191238
35 *osteoarthritis/ 55895
36 osteoarthritis.ti,ab. 131490
37 *systemic lupus erythematosus/ 67671
38 lupus.ti,ab. 135191
39 *systemic sclerosis/ 24539
40 (systemic sclerosis or scleroderma).ti,ab. 47556
41 *inflammatory bowel disease/ 33488
42 (inflammatory bowel disease* or IBD).ti,ab. 131258
43 *biliary cirrhosis/ 2407
44 (primary biliary cirrhosis or PBS).ti,ab. 62982
45 *sclerosing cholangitis/ 2054
46 sclerosing cholangiti*.ti,ab. 13366
47 *lung disease/ 35911
48 ((lung or pulmonary) adj (disease* or disorder* or condition*)).ti,ab. 237743
49 *chronic obstructive lung disease/ 88575
50 ((chronic obstructive adj (pulmonary or lung or airway) adj (disease* or obstruction*)) or (COPD or COAD)).ti,ab. 155701
51 *asthma/ 157738
52 (asthma or asthmatic).ti,ab. 271814
53 *muscle disease/ 10264
54 (((muscle or muscular or myopathic) adj (disorder* or disease* or condition*)) or (myopathy or myopathies)).ti,ab. 55279
55 *muscular dystrophy/ 9781
56 (muscular dystroph* or myodystroph*).ti,ab. 38669
57 or/1-56 4825307
58 "Fatigue Questionnaire".ti,ab. 685
59 "Fatigue Severity Scale".ti,ab. 3809
60 "Multidimensional Assessment of Fatigue".ti,ab. 278
61 "Short Form-36 Vitality".ti,ab. 41
62 ("Functional Assessment of Chronic Illness Therapy Fatigue" or FACIT F).ti,ab. 1940
63 "Brief Fatigue Inventory".ti,ab. 951
64 "Numerical Rating Scale Fatigue".ti,ab. 11
65 ("Visual Analog Scale Fatigue" or VAS F).ti,ab. 181
66 "Checklist Individual Strength".ti,ab. 500
67 "Chalder Fatigue Scale".ti,ab. 365
68 "Multidimensional Fatigue Inventory Scale".ti,ab. 18
69 "Piper Fatigue Scale".ti,ab. 401
70 (PROMIS-29 or PROMIS 29 or PROMIS29).ti,ab. 804
71 Pittsburgh Fatigability Scale.ti,ab. 56

72 Fatigue Descriptive Scale.ti,ab. 18
73 Modified Fatigue Impact Scale.ti,ab. 1198
74 ("40-item Fatigue Impact Scale" or "40 item Fatigue Impact Scale").ti,ab. 4
75 ("29-item Fatigue Assessment Instrument" or "29 item Fatigue Assessment Instrument").ti,ab. 2
76 ("Functional Assessment of Multiple Sclerosis" or FAMS).ti,ab. 374
77 exp Fatigue Severity Scale/ or exp "Functional Assessment of Chronic Illness Therapy Fatigue Scale"/ or exp Multidimensional Fatigue Inventory/ or exp Chalder Fatigue Scale/ or exp Piper fatigue scale/ or exp "fatigue scale for motor and cognitive functions"/ or exp Fatigue Impact Scale/ 7913
78 or/58-77 13999
79 *fatigue/ 27870
80 (fatigue adj7 (scale* or subscale* or sub-scale* or questionnaire* or assessment* or inventor* or measure* or tool*)).ti,ab. 31993
81 (scale or subscale or sub-scale or questionnaire or assessment or inventory or measure or measurement).ti,ab. 5085498
82 (fatigability or fatigable).ti,ab. 5536
83 79 or 82 32922
84 81 and 83 13199
85 80 or 84 35785
86 78 or 85 38984
87 57 and 86 14155
88 exp review/ 3355072
89 (literature adj3 review$).ti,ab. 494495
90 exp meta analysis/ 338595
91 exp "Systematic Review"/ 496268
92 88 or 89 or 90 or 91 3753315
93 (medline or medlars or embase or pubmed or cinahl or amed or psychlit or psyclit or psychinfo or psycinfo or scisearch or cochrane).ti,ab. 523890
94 RETRACTED ARTICLE/ 14987
95 93 or 94 538405
96 92 and 95 424367
97 (systematic$ adj2 (review$ or overview)).ti,ab. 458236
98 (meta?anal$ or meta anal$ or meta-anal$ or metaanal$ or metanal$).ti,ab. 411264
99 96 or 97 or 98 771746
100 87 and 99 519
101 limit 100 to "remove medline records" 247

CINAHL via EBSCO
S1 (MH "Chronic Disease+")
S2 TI ((chronic or long-term or long term) N1 (condition* or disease* or illness*)) OR AB ((chronic or long-term or long term) N1 (condition* or disease* or illness*))
S3 TI chronically ill OR AB chronically ill
S4 (MH "Rheumatic Diseases+")
S5 TI rheumati* OR AB rheumati*
S6 (MH "Diabetes Mellitus+")
S7 TI diabet* OR AB diabet*
S8 (MH "Endocrine Diseases+")
S9 (MH "Thyroid Diseases+")
S10 (MH "Adrenal Gland Diseases+")
S11 (MH "Adrenal Insufficiency+")
S12 (MH "Autoimmune Diseases+")
S13 TI ((endocrine or thyroid or adrenal or autoimmune or auto-immune or auto immune) N1 (disorder* or disease* or condition*)) OR AB ((endocrine or thyroid or adrenal or autoimmune or auto-immune or auto immune) N1 (disorder* or disease* or condition*))
S14 TI adrenal insufficiency OR AB adrenal insufficiency
S15 (MH "Heart Failure+")
S16 TI heart failure* OR AB heart failure*
S17 (MH "Coronary Disease+")
S18 TI coronary heart disease* OR AB coronary heart disease*

S19 (MH "Renal Insufficiency, Chronic+")
S20 (MH "Kidney Failure, Chronic+")
S21 TI (chronic adj (renal or kidney) N1 (insuffucien* or failure* or disease*)) OR AB (chronic adj (renal or kidney) N1 (insuffucien* or failure* or disease*))
S22 (MH "Dialysis Patients")
S23 TI dialysis OR AB dialysis
S24 TI (transplant* N3 (heart* or kidney* or liver* or lung*)) OR AB (transplant* N3 (heart* or kidney* or liver* or lung*))
S25 (MH "Multiple Sclerosis+")
S26 TI multiple sclerosis OR AB multiple sclerosis
S27 (MH "Stroke+")
S28 TI stroke OR AB stroke
S29 (MH "Neurodegenerative Diseases+")
S30 TI ((neurodegenerative or neuro-degenerative or neuro degenerative) N1 (disease* or disorder* or condition*)) OR AB ((neurodegenerative or neuro-degenerative or neuro degenerative) N1 (disease* or disorder* or condition*))
S31 (MH "Parkinson Disease")
S32 TI (parkinson* N1 disease) OR AB (parkinson* N1 disease)
S33 (MH "Arthritis, Rheumatoid+")
S34 TI rheumatoid arthritis OR AB rheumatoid arthritis
S35 (MH "Osteoarthritis+")
S36 TI osteoarthritis OR AB osteoarthritis
S37 (MH "Lupus Erythematosus, Systemic+")
S38 TI lupus OR AB lupus
S39 (MH "Scleroderma, Systemic+")
S40 TI (systemic sclerosis or scleroderma) OR AB (systemic sclerosis or scleroderma)
S41 (MH "Inflammatory Bowel Diseases+")
S42 TI (inflammatory bowel disease* or IBD) OR AB (inflammatory bowel disease* or IBD)
S43 (MH "Liver Cirrhosis+")
S44 TI (primary biliary cirrhosis or PBS) OR AB (primary biliary cirrhosis or PBS)
S45 (MH "Cholangitis, Sclerosing")
S46 TI sclerosing cholangiti* OR AB sclerosing cholangiti*
S47 (MH "Lung Diseases+")
S48 TI ((lung or pulmonary) N1 (disease* or disorder* or condition*)) OR AB ((lung or pulmonary) N1 (disease* or disorder* or condition*))
S49 (MH "Pulmonary Disease, Chronic Obstructive+")
S50 TI ((chronic obstructive N1 (pulmonary or lung or airway) N1 (disease* or obstruction*)) or (COPD or COAD)) OR AB ((chronic obstructive N1 (pulmonary or lung or airway) N1 (disease* or obstruction*)) or (COPD or COAD))
S51 (MH "Asthma+")
S52 TI (asthma or asthmatic) OR AB (asthma or asthmatic)
S53 (MH "Muscular Diseases+")
S54 TI (((muscle or muscular or myopathic) ADJ1 (disorder* or disease* or condition*)) or (myopathy or myopathies)) OR AB (((muscle or muscular or myopathic) ADJ1 (disorder* or disease* or condition*)) or (myopathy or myopathies))
S55 (MH "Muscular Dystrophy+")
S56 TI (muscular dystroph* or myodystroph*) OR AB (muscular dystroph* or myodystroph*)
S57 S1 OR S2 OR S3 OR S4 OR S5 OR S6 OR S7 OR S8 OR S9 OR S10 OR S11 OR S12 OR S13 OR S14 OR S15 OR S16 OR S17 OR S18 OR S19 OR S20 OR S21 OR S22 OR S23 OR S24 OR S25 OR S26 OR S27 OR S28 OR S29 OR S30 OR S31 OR S32 OR S33 OR S34 OR S35 OR S36 OR S37 OR S38 OR S39 OR S40 OR S41 OR S42 OR S43 OR S44 OR S45 OR S46 OR S47 OR S48 OR S49 OR S50 OR S51 OR S52 OR S53 OR S54 OR S55 OR S56
S58 TI "Fatigue Questionnaire" OR AB "Fatigue Questionnaire"
S59 TI "Fatigue Severity Scale" OR AB "Fatigue Severity Scale"
S60 TI "Multidimensional Assessment of Fatigue" OR AB "Multidimensional Assessment of Fatigue"
S61 TI "Short Form-36 Vitality" OR AB "Short Form-36 Vitality"
S62 TI ("Functional Assessment of Chronic Illness Therapy Fatigue" or FACIT F) OR AB ("Functional Assessment of Chronic Illness Therapy Fatigue" or FACIT F)
S63 TI "Brief Fatigue Inventory" OR AB "Brief Fatigue Inventory"
S64 TI "Numerical Rating Scale Fatigue" OR AB "Numerical Rating Scale Fatigue"

S65 TI ("Visual Analog Scale Fatigue" or VAS F) OR AB ("Visual Analog Scale Fatigue" or VAS F)
S66 TI "Checklist Individual Strength" OR AB "Checklist Individual Strength"
S67 TI "Chalder Fatigue Scale" OR AB "Chalder Fatigue Scale"
S68 TI "Multidimensional Fatigue Inventory Scale" OR AB "Multidimensional Fatigue Inventory Scale"
S69 TI "Piper Fatigue Scale" OR AB "Piper Fatigue Scale"
S70 TI (PROMIS-29 or PROMIS 29 or PROMIS29) OR AB (PROMIS-29 or PROMIS 29 or PROMIS29)
S71 TI Pittsburgh Fatigability Scale OR AB Pittsburgh Fatigability Scale
S72 TI Fatigue Descriptive Scale OR AB Fatigue Descriptive Scale
S73 TI Modified Fatigue Impact Scale OR AB Modified Fatigue Impact Scale
S74 TI ("40-item Fatigue Impact Scale" or "40 item Fatigue Impact Scale") OR AB ("40-item Fatigue Impact Scale" or "40 item Fatigue Impact Scale")
S75 TI ("29-item Fatigue Assessment Instrument" or "29 item Fatigue Assessment Instrument") OR AB ("29-item Fatigue Assessment Instrument" or "29 item Fatigue Assessment Instrument")
S76 TI ("Functional Assessment of Multiple Sclerosis" or FAMS) OR AB ("Functional Assessment of Multiple Sclerosis" or FAMS)
S77 S58 OR S59 OR S60 OR S61 OR S62 OR S63 OR S64 OR S65 OR S66 OR S67 OR S68 OR S69 OR S70 OR S71 OR S72 OR S73 OR S74 OR S75 OR S76
S78 (MM "Fatigue")
S79 TI (fatigue N7 (scale* or subscale* or sub-scale* or questionnaire* or assessment* or inventor* or measure* or tool*)) OR AB (fatigue N7 (scale* or subscale* or sub-scale* or questionnaire* or assessment* or inventor* or measure* or tool*))
S80 TI (scale or subscale or sub-scale or questionnaire or assessment or inventory or measure or measurement) OR AB (scale or subscale or sub-scale or questionnaire or assessment or inventory or measure or measurement)
S81 TI (fatigability or fatigable) OR AB (fatigability or fatigable)
S82 S78 OR S81
S83 S79 AND S80
S84 (S82 OR S83)
S85 (TI (systematic* n3 review*)) or (AB (systematic* n3 review*)) or (TI (systematic* n3 bibliographic*)) or (AB (systematic* n3 bibliographic*)) or (TI (systematic* n3 literature)) or (AB (systematic* n3 literature)) or (TI (comprehensive* n3 literature)) or (AB (comprehensive* n3 literature)) or (TI (comprehensive* n3 bibliographic*)) or (AB (comprehensive* n3 bibliographic*)) or (TI (integrative n3 review)) or (AB (integrative n3 review)) or (JN “Cochrane Database of Systematic Reviews”) or (TI (information n2 synthesis)) or (TI (data n2 synthesis)) or (AB (information n2 synthesis)) or (AB (data n2 synthesis)) or (TI (data n2 extract*)) or (AB (data n2 extract*)) or (TI (medline or pubmed or psyclit or cinahl or (psycinfo not “psycinfo database”) or “web of science” or scopus or embase)) or (AB (medline or pubmed or psyclit or cinahl or (psycinfo not “psycinfo database”) or “web of science” or scopus or embase)) or (MH “Systematic Review”) or (MH “Meta Analysis”) or (TI (meta-analy* or metaanaly*)) or (AB (meta-analy* or metaanaly*)) 319,272
S86 (S84 AND S85) 266

APA PsycInfo <1806 to November 2024 Week 4>

1 exp Chronic Illness/ 38747
2 ((chronic or long-term or long term) adj (condition* or disease* or illness*)).ti,ab. 31815
3 chronically ill.ti,ab. 3266
4 exp Rheumatoid Arthritis/ 2191
5 rheumati*.ti,ab. 1171
6 exp Diabetes Mellitus/ 10907
7 diabet*.ti,ab. 38686
8 exp Thyroid Disorders/ 1601
9 exp Adrenal Gland Disorders/ 434
10 exp Immunologic Disorders/ 62844
11 ((endocrine or thyroid or adrenal or autoimmune or auto-immune or auto immune) adj1 (disorder* or disease* or condition*)).ti,ab. 4101
12 adrenal insufficiency.ti,ab. 152
13 exp Heart Disorders/ 17285
14 heart failure*.ti,ab. 4886
15 exp Cardiovascular Disorders/ 75870

16 coronary heart disease*.ti,ab. 4513
17 exp Kidney Diseases/ 2855
18 (chronic adj (renal or kidney) adj (insuffucien* or failure* or disease*)).ti,ab. 1678
19 exp Dialysis/ 2398
20 dialysis.ti,ab. 2467
21 exp Organ Transplantation/ 5681
22 (transplant* adj3 (heart* or kidney* or liver* or lung*)).ti,ab. 2172
23 exp Multiple Sclerosis/ 14944
24 multiple sclerosis.ti,ab. 17760
25 exp Cerebrovascular Accidents/ 26271
26 stroke.ti,ab. 39801
27 exp Neurodegenerative Diseases/ 102451
28 ((neurodegenerative or neuro-degenerative or neuro degenerative) adj (disease* or disorder* or condition*)).ti,ab. 20837
29 (parkinson* adj disease).ti,ab. 34494
30 exp Rheumatoid Arthritis/ 2191
31 rheumatoid arthritis.ti,ab. 2904
32 exp Arthritis/ 5077
33 osteoarthritis.ti,ab. 2449
34 exp Lupus/ 922
35 lupus.ti,ab. 1691
36 (systemic sclerosis or scleroderma).ti,ab. 232
37 exp Colon Disorders/ 6783
38 (inflammatory bowel disease* or IBD).ti,ab. 1324
39 exp Liver Disorders/ 5368
40 (primary biliary cirrhosis or PBS).ti,ab. 1666
41 sclerosing cholangiti*.ti,ab. 21
42 exp Lung Disorders/ 6793
43 ((lung or pulmonary) adj (disease* or disorder* or condition*)).ti,ab. 4200
44 exp Chronic Obstructive Pulmonary Disease/ 1930
45 ((chronic obstructive adj (pulmonary or lung or airway) adj (disease* or obstruction*)) or (COPD or COAD)).ti,ab. 3223
46 exp Asthma/ 5507
47 (asthma or asthmatic).ti,ab. 8811
48 exp Muscular Disorders/11266
49 (((muscle or muscular or myopathic) adj (disorder* or disease* or condition*)) or (myopathy or myopathies)).ti,ab. 1877
50 exp Muscular Dystrophy/ 1575
51 (muscular dystroph* or myodystroph*).ti,ab. 1679
52 or/1-51 403622
53 "Fatigue Questionnaire".ti,ab. 135
54 "Multidimensional Assessment of Fatigue".ti,ab. 36
55 "Short Form-36 Vitality".ti,ab. 8
56 ("Functional Assessment of Chronic Illness Therapy Fatigue" or FACIT F).ti,ab. 85
57 "Brief Fatigue Inventory".ti,ab. 112
58 "Numerical Rating Scale Fatigue".ti,ab. 4
59 ("Visual Analog Scale Fatigue" or VAS F).ti,ab. 26
60 "Checklist Individual Strength".ti,ab. 121
61 "Chalder Fatigue Scale".ti,ab. 84
62 "Multidimensional Fatigue Inventory Scale".ti,ab. 0
63 "Piper Fatigue Scale".ti,ab. 76
64 (PROMIS-29 or PROMIS 29 or PROMIS29).ti,ab. 83
65 Pittsburgh Fatigability Scale.ti,ab. 14
66 Fatigue Descriptive Scale.ti,ab. 1
67 Modified Fatigue Impact Scale.ti,ab. 151
68 ("40-item Fatigue Impact Scale" or "40 item Fatigue Impact Scale").ti,ab. 2
69 ("29-item Fatigue Assessment Instrument" or "29 item Fatigue Assessment Instrument").ti,ab. 1
70 ("Functional Assessment of Multiple Sclerosis" or FAMS).ti,ab. 62
71 or/52-70 404079

72	exp Fatigue/	12644
73	(fatigue adj7 (scale* or subscale* or sub-scale* or questionnaire* or assessment* or inventor* or measure* or tool*)).ti,ab.	5752
74	(scale or subscale or sub-scale or questionnaire or assessment or inventory or measure or measurement).ti,ab.	1120968
75	(fatigability or fatigable).ti,ab.	558
76	72 or 75	13033
77	74 and 76	5113
78	73 or 77	8002
79	52 and 78	2577
80	(meta-analysis or search:).tw.	169257
81	79 and 80	93

Web of Science Core Collection (Science and Social Sciences Citation Indexes – SCI-EXPANDED, SSCI)
TS=(((chronic) NEAR/1 (condition* or disease* or illness*)))	Results: 359541
(TS=(((long-term) NEAR/1 (condition* or disease* or illness*))))	Results: 18258
TS=(chronically ill)	Results: 6642
TS=(rheumati*)	Results: 60176
TS=(diabet*)	Results: 950365
TS=(((endocrine) NEAR/1 (disorder* or disease* or condition*)))	Results: 10304
TS=(((thyroid) NEAR/1 (disorder* or disease* or condition*)))	Results: 22399
TS=(((adrenal) NEAR/1 (disorder* or disease* or condition*)))	Results: 1817
TS=(((autoimmune) NEAR/1 (disorder* or disease* or condition*)))	Results: 116974
TS=(((auto-immune) NEAR/1 (disorder* or disease* or condition*)))	Results: 2123
TS=(((auto immune) NEAR/1 (disorder* or disease* or condition*)))	Results: 3843
TS=adrenal insufficiency	Results: 9764
TS=heart failure*	Results: 358758
TS=coronary heart disease*	Results: 204650
TS=(chronic NEAR/1 (renal or kidney) NEAR/1 (insuffucien* or failure* or disease*))	Results: 133763
TS=dialysis	Results: 149587
TS=(transplant* NEAR/3 (heart* or kidney* or liver* or lung*))	Results: 275387
TS=multiple sclerosis	Results: 156702
TS=stroke	Results: 450117
TS=((neurodegenerative) NEAR/1 (disease* or disorder* or condition*))	Results: 117914
TS=((neuro-degenerative) NEAR/1 (disease* or disorder* or condition*))	Results: 826
TS=((neuro degenerative) NEAR/1 (disease* or disorder* or condition*))	Results: 1058
TS=(parkinson* NEAR/1 disease)	Results: 190306
TS=rheumatoid arthritis	Results: 205223
TS=osteoarthritis	Results: 123264
TS=lupus	Results: 132734
TS=(systemic sclerosis or scleroderma)	Results: 49435
TS=(inflammatory bowel disease* or IBD)	Results: 123692
TS=(primary biliary cirrhosis or PBS)	Results: 60151
TS=sclerosing cholangiti*	Results: 12099
TS=((lung or pulmonary) NEAR/1 (disease* or disorder* or condition*))	Results: 185254
TS=((chronic obstructive NEAR/1 (pulmonary or lung or airway) NEAR/1 (disease* or obstruction*)) or (COPD or COAD))	Results: 112767
TS=(asthma or asthmatic)	Results: 239662
TS=(((muscle or muscular or myopathic) NEAR/1 (disorder* or disease* or condition*)) or (myopathy or myopathies))	Results: 54161
TS=(muscular dystroph* or myodystroph*)	Results: 41449
#1 OR #2 OR #3 OR #4 OR #5 OR #6 OR #7 OR #8 OR #9 OR #10 OR #11 OR #12 OR #13 OR #14 OR #15 OR #16 OR #17 OR #18 OR #19 OR #20 OR #21 OR #22 OR #23 OR #24 OR #25 OR #26 OR #27 OR #28 OR #29 OR #30 OR #31 OR #32 OR #33 OR #34 OR #35	Results: 3991303
TS=(fatigue or fatigability or fatigable)	Results: 288108
TS=(fatigue NEAR/7 (scale* or subscale* or sub-scale* or questionnaire* or assessment* or inventor* or measure* or tool*))	Results: 29495

TS=(scale or subscale or sub-scale or questionnaire or assessment or inventory or measure or measurement) Results: 11727743
#37 AND #39 Results: 103757
#38 OR #40 Results: 104634
#36 AND #41 Results: 17495
TI=(systematic NEAR/3 (review OR overview)) OR TI=(methodologic NEAR/3 (review OR overview)) OR TI=(quantitative NEAR/3 (review OR overview OR synthesis)) OR TI=(research NEAR/3 (integrative OR overview)) OR TI=(integrative NEAR/3 (review OR overview)) OR TI=(collaborative NEAR/3 (review OR overview)) Results: 316800
#42 AND #43 Results: 627

Cochrane Database of Systematic Reviews
Issue 11 of 12, November 2024

ID	Search
#1	MeSH descriptor: [Chronic Disease] explode all trees
#2	((chronic or long-term or long term) NEXT (condition* or disease* or illness*)):ti OR ((chronic or long-term or long term) NEXT (condition* or disease* or illness*)):ab
#3	chronically ill:ti OR chronically ill:ab
#4	MeSH descriptor: [Rheumatic Diseases] explode all trees
#5	rheumati*:ti OR rheumati*:ab
#6	MeSH descriptor: [Diabetes Mellitus] explode all trees
#7	diabet*:ti OR diabet*:ab
#8	MeSH descriptor: [Endocrine System Diseases] explode all trees
#9	MeSH descriptor: [Thyroid Diseases] explode all trees
#10	MeSH descriptor: [Adrenal Gland Diseases] explode all trees
#11	MeSH descriptor: [Adrenal Insufficiency] explode all trees
#12	MeSH descriptor: [Autoimmune Diseases] explode all trees
#13	((endocrine or thyroid or adrenal or autoimmune or auto-immune or auto immune) NEAR/1 (disorder* or disease* or condition*)):ti OR ((endocrine or thyroid or adrenal or autoimmune or auto-immune or auto immune) NEAR/1 (disorder* or disease* or condition*)):ab
#14	adrenal insufficiency:ti OR adrenal insufficiency:ab
#15	MeSH descriptor: [Heart Failure] explode all trees
#16	heart failure*:ti OR heart failure*:ab
#17	MeSH descriptor: [Coronary Disease] explode all trees
#18	coronary heart disease*:ti OR coronary heart disease*:ab
#19	MeSH descriptor: [Renal Insufficiency, Chronic] explode all trees
#20	MeSH descriptor: [Kidney Failure, Chronic] explode all trees
#21	(chronic NEXT (renal or kidney) NEXT (insuffucien* or failure* or disease*)):ti OR (chronic NEXT (renal or kidney) NEXT (insuffucien* or failure* or disease*)):ab
#22	MeSH descriptor: [Renal Dialysis] explode all trees
#23	dialysis:ti OR dialysis:ab
#24	MeSH descriptor: [Transplantation] explode all trees
#25	(transplant* NEAR/3 (heart* or kidney* or liver* or lung*)):ti OR (transplant* NEAR/3 (heart* or kidney* or liver* or lung*)):ab
#26	MeSH descriptor: [Multiple Sclerosis] explode all trees
#27	multiple sclerosis:ti OR multiple sclerosis:ab
#28	MeSH descriptor: [Stroke] explode all trees
#29	stroke:ti OR stroke:ab
#30	MeSH descriptor: [Neurodegenerative Diseases] explode all trees
#31	((neurodegenerative or neuro-degenerative or neuro degenerative) NEXT (disease* or disorder* or condition*)):ti OR ((neurodegenerative or neuro-degenerative or neuro degenerative) NEXT (disease* or disorder* or condition*)):ab
#32	MeSH descriptor: [Parkinson Disease] explode all trees
#33	(parkinson* NEXT disease):ti OR (parkinson* NEXT disease):ab
#34	MeSH descriptor: [Arthritis, Rheumatoid] explode all trees
#35	rheumatoid arthritis:ti OR rheumatoid arthritis:ab
#36	MeSH descriptor: [Osteoarthritis] explode all trees
#37	osteoarthritis:ti OR osteoarthritis:ab
#38	MeSH descriptor: [Lupus Erythematosus, Systemic] explode all trees
#39	lupus:ti OR lupus:ab

#40 MeSH descriptor: [Scleroderma, Systemic] explode all trees
#41 (systemic sclerosis or scleroderma):ti OR (systemic sclerosis or scleroderma):ab
#42 MeSH descriptor: [Inflammatory Bowel Diseases] explode all trees
#43 (inflammatory bowel disease* or IBD):ti OR (inflammatory bowel disease* or IBD):ab
#44 MeSH descriptor: [Liver Cirrhosis, Biliary] explode all trees
#45 (primary biliary cirrhosis or PBS):ti OR (primary biliary cirrhosis or PBS):ab
#46 MeSH descriptor: [Cholangitis, Sclerosing] explode all trees
#47 sclerosing cholangiti*:ti OR sclerosing cholangiti*:ab
#48 MeSH descriptor: [Lung Diseases] explode all trees
#49 ((lung or pulmonary) NEXT (disease* or disorder* or condition*)):ti OR ((lung or pulmonary) NEXT (disease* or disorder* or condition*)):ab
#50 MeSH descriptor: [Pulmonary Disease, Chronic Obstructive] explode all trees
#51 ((chronic obstructive NEXT (pulmonary or lung or airway) NEXT (disease* or obstruction*)) or (COPD or COAD)):ti OR ((chronic obstructive NEXT (pulmonary or lung or airway) NEXT (disease* or obstruction*)) or (COPD or COAD)):ab
#52 MeSH descriptor: [Asthma] explode all trees
#53 (asthma or asthmatic):ti OR (asthma or asthmatic):ab
#54 MeSH descriptor: [Muscular Diseases] explode all trees
#55 (((muscle or muscular or myopathic) NEXT (disorder* or disease* or condition*)) or (myopathy or myopathies)):ti OR (((muscle or muscular or myopathic) NEXT (disorder* or disease* or condition*)) or (myopathy or myopathies)):ab
#56 MeSH descriptor: [Muscular Dystrophies] explode all trees
#57 (muscular dystroph* or myodystroph*):ti OR (muscular dystroph* or myodystroph*):ab
#58 {OR #1-#57}
#59 "Fatigue Questionnaire":ti OR "Fatigue Questionnaire":ab
#60 "Fatigue Severity Scale":ti OR "Fatigue Severity Scale":ab
#61 "Multidimensional Assessment of Fatigue":ti OR "Multidimensional Assessment of Fatigue":ab
#62 "Short Form-36 Vitality":ti OR "Short Form-36 Vitality":ab
#63 ("Functional Assessment of Chronic Illness Therapy Fatigue" or FACIT F):ti OR ("Functional Assessment of Chronic Illness Therapy Fatigue" or FACIT F):ab
#64 "Brief Fatigue Inventory":ti OR "Brief Fatigue Inventory":ab
#65 "Numerical Rating Scale Fatigue":ti OR "Numerical Rating Scale Fatigue":ab
#66 ("Visual Analog Scale Fatigue" or VAS F):ti OR ("Visual Analog Scale Fatigue" or VAS F):ab
#67 "Checklist Individual Strength":ti OR "Checklist Individual Strength":ab
#68 "Chalder Fatigue Scale":ti OR "Chalder Fatigue Scale":ab
#69 "Multidimensional Fatigue Inventory Scale":ti OR "Multidimensional Fatigue Inventory Scale":ab
#70 "Piper Fatigue Scale":ti OR "Piper Fatigue Scale":ab
#71 (PROMIS-29 or PROMIS 29 or PROMIS29):ti OR (PROMIS-29 or PROMIS 29 or PROMIS29):ab
#72 Pittsburgh Fatigability Scale:ti OR Pittsburgh Fatigability Scale:ab
#73 Fatigue Descriptive Scale:ti OR Fatigue Descriptive Scale:ab
#74 Modified Fatigue Impact Scale:ti OR Modified Fatigue Impact Scale:ab
#75 ("40-item Fatigue Impact Scale" or "40 item Fatigue Impact Scale"):ti OR ("40-item Fatigue Impact Scale" or "40 item Fatigue Impact Scale"):ab
#76 ("29-item Fatigue Assessment Instrument" or "29 item Fatigue Assessment Instrument"):ti OR ("29-item Fatigue Assessment Instrument" or "29 item Fatigue Assessment Instrument"):ab
#77 ("Functional Assessment of Multiple Sclerosis" or FAMS):ti OR ("Functional Assessment of Multiple Sclerosis" or FAMS):ab
#78 {OR #59-#77}
#79 MeSH descriptor: [Fatigue] this term only
#80 (fatigue NEXT/7 (scale* or subscale* or sub-scale* or questionnaire* or assessment* or inventor* or measure* or tool*)):ti OR (fatigue NEXT/7 (scale* or subscale* or sub-scale* or questionnaire* or assessment* or inventor* or measure* or tool*)):ab
#81 (scale or subscale or sub-scale or questionnaire or assessment or inventory or measure or measurement):ti OR (scale or subscale or sub-scale or questionnaire or assessment or inventory or measure or measurement):ab
#82 (fatigability or fatigable):ti OR (fatigability or fatigable):ab
#83 #79 OR #82
#84 #81 AND #83

#85	#80 OR #84
#86	#58 AND #85

2 Supplementary Methods 2 risk of bias assessment

2.1 Detailed description of methods

Risk of bias assessment of all studies included in the network meta-analysis (NMA) of the present review was undertaken by two experienced reviewers (MMSJ and JL). Any disagreements were resolved through discussion.

Version 2 of the Cochrane risk-of-bias tool (RoB2) for randomised trials (RCTs) [(Sterne et al. 2019] is the recommended tool to assess the risk of bias in RCTs included in Cochrane Reviews. We selected RoB2 as this provides a domain-based approach to identifying biases in RCTs. The tool is structured into five domains through which bias might be introduced into an RCT's results:

Domain1: bias arising from the randomisation process,

Domain 2: bias due to deviations from intended interventions,

Domain 3: bias due to missing outcome data,

Domain 4: bias in measurement of the outcome; and

Domain 5: bias in selection of the reported result.

The judgment for each domain (high risk, low risk, some concerns) is then used to inform an overall risk of bias judgement for each RCT (high risk, low risk, some concerns).

These domains focus on different aspects of trial design, conduct, and reporting. Within each domain, a series of questions ('signalling questions') aim to elicit information about features of the RCT that are relevant to risk of bias. On the RoB2 tool, each signalling question has up to six possible responses - yes, partial yes, no, partial no, not applicable, no information.

There are 22 signalling questions in total in RoB2, which means that the completion rate for each RCT included in a review can be lengthy. We estimate ≤4 RCT reports per day. For pragmatic reasons relating the volume of studies included in the NMA in the present review, we adapted the RoB2 tool to facilitate quicker completion, whilst still capturing the issues with methodological conduct, reporting and other potential biases we observed in some of the RCTs (independent, small-scale, unregistered, minimally reported) that were included in the present NMA.

Two reviewers with RoB2 experience adapted the RoB2 tool so that some signalling questions that would be redundant for the RCTs included in the present NMA were omitted. The remaining signalling questions (n=15) responses were still yes, no, or unclear. We then adapted the RoB2 algorithms to be able to still judge each domain as low risk, high risk, or some concerns. We also retained the overall RoB2 tool risk of bias judgement algorithm for each RCT as follows:

All domains judged as 'low risk' = overall low risk,

Any domain judged as 'high risk' = overall high risk,

All domains judged as 'some concerns' = overall some concerns

Some domains 'low risk' and some domains 'unclear risk' = some concerns

Through the adaptation process we were also able to combine some domains resulting in four assessment domains, with a total of 15 signalling questions, as follows:

Domain1: bias arising from the randomisation process – three signalling questions,

Domain 2: bias due to blinding– three signalling questions,

Domain 3: bias due to missing outcome data– five signalling questions; and

Domain 4: Selection of the reported result and analysis of the outcome– four signalling questions

Agreement of the domains and signalling questions to be included in the adapted RoB2 tool was reached through discussion. We developed the adapted RoB2 tool in Excel and the two reviewers independently piloted this on 10 of the RCTs included in the NMA. Any amendments needed to the tool were discussed and agreed through discussion. As a result of this process, a key adjustment was made to the criteria used for risk of bias judgements for 'attrition'. In consultation with the subject experts on the team we amended our prior threshold of >10% attrition to equal high risk of bias to >20%, due to this level of attrition being within the bounds of normal expectations for behavioural interventions. A copy of the adapted RoB2 tool is presented below.

Reference:

Sterne JAC, Savović J, Page MJ, Elbers RG, Blencowe NS, Boutron I, Cates CJ, Cheng H-Y, Corbett MS, Eldridge SM, Hernán MA, Hopewell S, Hróbjartsson A, Junqueira DR, Jüni P, Kirkham JJ, Lasserson T, Li T, McAleenan A, Reeves BC, Shepperd S, Shrier I, Stewart LA, Tilling K, White IR, Whiting PF, Higgins JPT. RoB 2: a revised tool for assessing risk of bias in randomised trials. BMJ 2019; 366: l4898.

2.2 Adapted Risk of Bias 2 assessment criteria

Domain	Signalling questions	Responses	Domain judgement
Domain 1: Risk of bias arising from the randomization process	Is randomisation unbiased	Yes – e.g., computer generated<br>No – e.g., alternate allocation<br>Unclear – e.g., 1:1 ratio or method not reported	All 'yes' = low risk<br>All 'no' = high risk<br>1 or 2 'unclear' = unclear risk<br>1 or 2 'no' = high risk
	Is allocation concealed	Yes – e.g., sequential, opaque sealed envelopes, interactive voice<br>No – e.g., staff aware of assignment<br>Unclear – method not reported	
	Are groups balanced at baseline	Yes – balanced in the publication<br>No – balanced in the publication<br>Unclear -not reported	
Domain 2: Risk of bias due to blinding	Are participants blind	Yes – reported as blind<br>No – reported as unblinded or impossible that intervention could be blind from participants<br>Unclear -not reported (where intervention could be blinded)	All 'yes' = low risk<br>All 'no' = high risk<br>All 'unclear' = unclear risk<br>Any 'no' = high risk
	Are care-givers blind	Yes – reported as blind<br>No – reported as unblinded or impossible that intervention could be blind from caregivers<br>Unclear -not reported (where intervention could be blinded)	
	Are outcome assessors blind	Yes – reported as blind<br>No – reported as unblinded or impossible that group allocation could be blind from outcome assessors<br>Unclear -not reported (where group allocation could be blinded)	
Domain 3: Missing outcome data	Is sample size based on power	Yes – sample sized based on power calculation<br>No – reported as underpowered<br>Unclear -not reported	All 'yes' = low risk<br>All 'no' = high risk<br>All 'unclear' = unclear risk<br>2, 3, 4, 'yes', 5 'no' = low risk<br>2, 3, 4, or 5 'no' = high risk
	Is number recruited and completed reported	Yes – both n allocation and n completed reported<br>No – only reports n allocated or only reports n completed<br>Unclear -unclear if n is n allocated or n completed	
	Is attrition <20%	Yes – attrition <20% in all study groups<br>No – attrition ≥20% in any study group<br>Unclear -attrition not reported or unclear	

Domain	Signalling questions	Responses	Domain judgement
	Are withdrawals balanced across groups	Yes – attrition balanced across groups<br>No – attrition imbalanced across groups<br>Unclear -not reported or unclear	
	Is ITT used to include withdrawals	Yes – reported as analysed as ITT and ns in flowcharts and tables support this<br>No – reported as completer analysis only<br>Unclear - reported as analysed as ITT but ns in flowcharts and tables do not support this or unclear if ITT and ns not reported	
Domain 4: Selection of the reported result and analysis of the outcome	Is the study on a trials register (reported in the paper)?	Yes – trial register and number reported<br>No – reports that it is not on a trials register or unable to find on one	All 'yes' = low risk<br>All 'no' = high risk<br>All 'unclear' = unclear risk<br>3 or 4 'no' = high risk
	Is there a pre-defined protocol that can be obtained (i.e., as a supplement not just a trials register record)?	Yes – full study protocol available<br>No – reports there is a study protocol, but no details of how to obtain it<br>Unclear – not reported if there is a protocol or not	
	Is the outcome and its analysis pre-specified in the protocol	Yes – in the protocol<br>No – not in the protocol<br>Unclear – unable to assess (no protocol)	
	Is the outcome analysed as per the protocol	Yes – matches the protocol<br>No – difference between what is in the publication and the protocol<br>Unclear – unable to assess (no protocol)	
Overall Risk of Bas Judgment			
			All domains 'low' = low risk<br>Any domain 'high' = high risk<br>All domains 'unclear' = some concerns<br>Some domains 'low' and some domains 'unclear' = some concerns

3 Supplementary Methods 3 GRADE Classification

3.1 Adapted GRADE methods.

We assessed the certainty of the effect estimates of non-pharmacological interventions for fatigue compared to usual care using an adaption of GRADE and CINeMA methodology. Methods are described in detail below, and were designed to be appropriate for interpreting results from the body of evidence identified in this review, with judgements based on the data generated by the analyses of our network meta-analysis. The CINeMA framework is largely based on the GRADE framework, with modifications to facilitate assessment of network meta-analyses. As part of the introduction of CINeMA, a web application has been developed, which enables the conduct of network meta-analyses via the netmeta package in R within the application. As our analyses were conducted using a Bayesian approach and the R2WinBUGS package, we were unable to utilise the CINeMA web application for assessment of the network. Therefore, we used a modified GRADE assessment, incorporating elements of the CINeMA framework for assessment of heterogeneity and inconsistency.

All evidence was derived from randomised controlled trials (RCTs), which were considered to be high quality as a starting point. As per GRADE methodology, the quality of evidence was to be upgraded for large effect size (up one or two levels depending on the magnitude of the effect size) and dose response (up one level). Quality was downgraded for high risk of bias, imprecision, inconsistency and heterogeneity. Number of participants given in the summary tables refers to total N in the relevant intervention arm, not the total in the studies. This is due to inclusion of indirect evidence which may be categorised in another intervention arm (not usual care). The evidence for each outcome was assessed using this framework by JL (RoB, publication bias) and JF (inconsistency, heterogeneity) and validated by the other, or independently in duplicate by JL and JF (imprecision). Disagreements were resolved through discussion. Any uncertainties were discussed with CB. Effect sizes were graded using Cohen's categories; not substantial (SMD<0.2), small (0.2<=SMD<0.5), medium (0.5<=SMD<0.8), large (0.8<=SMD) (J. Cohen, *Statistical power analysis for the behavioral sciences*. Academic press, 2013). Final ratings were high, moderate, low or very low.

GRADE judgements were based on the following domains of the GRADE book (Neumann et al. 2024):

Limitations in the design or execution of randomized trials (RoB): Individual studies were assessed using Cochrane RoB v2.0 tool. Overall ratings were then clustered by intervention group, as analysed in the NMA. For a variety of reasons, outlined in our detailed RoB section, the evidence included in most intervention groups was rated as high risk of bias and was downgraded by 1. The reason for an overall high RoB rating in many studies was for 'blinding', which may not be possible in behavioural interventions. Lack of blinding is more problematic with outcomes that

have a subjective component, and as the fatigue measures in the included studies were self-reported and were administered in situations where participants or investigators could influence the probability of the outcomes, studies of these interventions were judged to be at high risk of bias as per the RoB v 2.0 handbook. The few studies with overall ‘some concerns’ were judged to also be at risk of bias and were also downgraded by 1.

Inconsistency: For the purpose of applying the GRADE rating, inconsistency was interpreted as any meaningful differences between the direct evidence (provided by the study data used within the NMA) and indirect evidence (resulting from indirect comparisons within the network). Comparisons of interventions relative to usual care were assessed as these are the primary results presented within forest plots. Other comparisons within the network may exhibit potential inconsistencies but not have been within this assessment. Agreement of indirect and direct evidence was assessed via node-splitting, if any of the estimated treatment effects from direct evidence were statistically significantly different from indirect estimates or no direct evidence was available, the comparison was downgraded by 1. If the difference between the two estimates (direct and indirect) differed by an amount greater than 0.34, chosen as a clinically meaningful SMD, the comparison was downgraded by one. At all three timepoints, the majority of interventions could not be assessed for inconsistency of evidence relative to usual care, due to other comparators being used and connecting within the network.

Imprecision: Ratings were based around thresholds for the minimal important difference for fatigue. In consultation with subject experts, we used a threshold of an SMD of 0.34 as clinically meaningful, as described in the methods section. Imprecision was judged on whether the credible intervals of estimated treatment effects spanned both the lower and upper bounds of the clinically meaningful SMD, i.e. -0.34 and +0.34. If a credible interval spanned both -0.34 and +0.34, we rated down by 2 levels (major concerns), if the 95% credible interval spanned SMD=0 and one clinically meaningful threshold, we rated down by 1 (serious imprecision) and if the 95% credible interval was entirely included within the shaded region we did not downgrade.

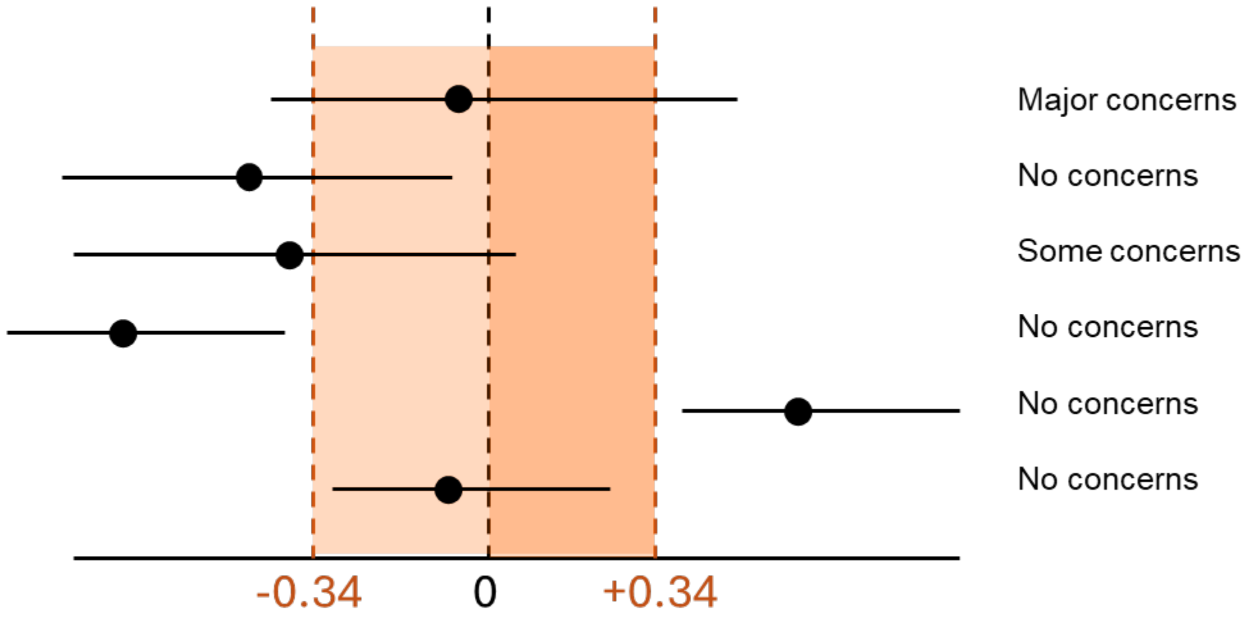

Values which do not clinically meaningfully favour the intervention or usual care. Darker region indicates interval between null effect and clinically important effect in opposite direction to observed effect.

Heterogeneity: The 95% prediction intervals were compared to the clinically meaningful thresholds. The estimated intervals capture the uncertainty in the modelled treatment effect but also the heterogeneity between studies. The prediction intervals were graded as for the 95% credible intervals for the imprecision domain, as shown in the figure above.

Publication bias: Funnel plots were not created due to too few studies comparing the same two interventions. Any remaining assessments regarding publication bias are by necessity based on subjective judgements around the likelihood that evidence has been missed, for example: non-inclusion of conference abstracts or grey literature; non-publication of negative studies without an external funder; non-publication of negative studies in novel/emerging interventions. We decided therefore not to include publication bias as a formal domain in our overall assessment. Instead we offer the following observations: The body of evidence for non-pharmacological interventions comes from studies that are generally small in nature. These were not always externally funded or published on trials registries and it is therefore possible that other, similar studies with negative results may not have been published. This is also possible for some emerging interventions, for example the stimulation interventions, where there is a risk of publication bias due to studies with negative results potentially remaining unpublished. It is also important to note that just under half of the intervention groups consist of only one study - in fact all of those included in the nutritional group are single study interventions. Because of the small, exploratory nature of some of these single study interventions, we chose not to upgrade for large effect size where a large effect was observed.

References

Schünemann H, Brożek J, Guyatt G, Oxman A, editors. GRADE handbook for grading quality of evidence and strength of recommendations. Updated October 2013. The GRADE Working Group, 2013. Available from guidelinedevelopment.org/handbook.

Nikolakopoulou, A., Higgins, J. P., Papakonstantinou, T., Chaimani, A., Del Giovane, C., Egger, M., & Salanti, G. (2020). CINeMA: an approach for assessing confidence in the results of a network meta-analysis. *PLoS medicine*, *17*(4), e1003082.

4 Supplementary Methods 4 : Intervention classification criteria

4.1 Physical activity-oriented interventions

4.1.1 Exercise Supervised.

Multiple sessions involving physical activity in a supervised / observed environment. Can include familiarisation with exercise, addressing barriers to exercise in addition to PA aimed at increasing exercise capacity / strength or fitness. May include recommendations to continue between sessions. May be delivered in groups or 1:1. May be in a gym, community resource, or outdoors. May include advice to repeat at home. Excludes specific forms of activity around training e.g. balance.

4.1.2 Exercise Unsupervised.

Unsupervised exercise at home aimed at increasing exercise capacity / strength or fitness. May involve initial explanation of physical activity and addressing barriers to exercise. Includes ongoing contact / review to adapt exercise through the programme. May include initial or occasional observed sessions. May take place at home or at other personally relevant location.

4.1.3 Physical Activity Promotion

One or more sessions aimed at increasing physical activity by addressing barriers to exercise, goal setting, and encouraging greater physical activity. May involve motivational techniques, cognitive / behavioural features, reporting and feedback, or the use of activity sensors. Less focused on structured exercise regimen than Exercise-Home,

4.1.4 Active Recreational

Engage in physical activity generally used as recreation – includes hippotherapy, dance and Interventions involving specific therapeutic environment. Combine body based activity with indirect positive mental well-being. *(This was placed in the classification but no studies were included)*

4.2 Self-management interventions

4.2.1 CBT-Fatigue

Multiple sessions, focused on reducing and adapting to fatigue. May include other symptoms or aspects of the condition. Content includes (1) discussion of helpful / unhelpful thoughts and beliefs (2) behavioural activation - this may include increasing physical activity, management of time / resources, and body/emotion regulating activities (3) tasks / homework between sessions. May be 1:1 or in groups, in person or online. May include second generation features such as Acceptance and Commitment. Can include mindfulness as long as clearly meets CBT definition

4.2.2 Fatigue Management- Active

One or more sessions focused on adapting to fatigue and increasing overall activity / engagement. Has only limited emphasis on energy conservation. Includes encouragement to increase activity – either social (behavioural activation) or physical (explicitly increasing physical activity). May include isolated CBT component such as thought challenges but does not meet sufficient criteria for CBT

4.2.3 Fatigue Management – Conservative

One or more sessions focused on adapting to fatigue. Primary focus is on energy conservation and prudent allocation. Does not explicitly encourage increase in overall physical or social activity or set out to challenge thoughts. Includes activity pacing and other energy conservation concepts

4.2.4 General Self-Management

Multiple sessions focused on self-management of specific medical condition / disability. May include condition-related fatigue but that is not the primary focus (see Fatigue Self-Management). Includes condition monitoring / specific self-care.

4.2.5 Non-Specific Rehabilitation

Multiple sessions focused on rehab from medical condition / disability. Has specific focus on either function or condition.

4.3 Mind / Mind-body interventions

4.3.1 Mind-Body

Multiple sessions at least partly supervised which use approaches to maximise body-mind connection. Can involve traditional methods (yoga, tai-chi) or "scientific" methods e.g. neurofeedback. Emphasises control of the body (contrast with mindfulness which emphasises control of the mind). Also includes Pilates and Exercise-Breathing where slow movement and controlled breathing are combined. Includes interventions focused on mental relaxation/control e.g. relaxation, imagery etc.

4.3.2 Mindfulness-Based

Multiple contacts focusing on learning and applying mindfulness-based techniques (meditation / breathwork). May include general guidance on living within energy resources, sleep, mental health and social interaction. Main focus is on applying mindfulness to daily life (rather than explicitly on addressing fatigue – which would be categorised as CBT with mindfulness)

4.3.3 Psychosocial adaptation to condition

Psychosocial intervention focused on adapting to emotional consequences of medical condition. Less explicit structure and content than CBT, more focus on emotional consequences and less on other behavioural factors than Living Well / rehabilitation. *(This was placed in the classification but no studies were included)*

4.3.4 Other Psychological Therapy

Multiple sessions, focused adapting to medical condition without specific focus on fatigue. Includes condition focused cognitive therapy and problem solving

4.4 Stimulation

4.4.1 Non-invasive neurostimulation

Use of one or more sessions of external stimulation of the nervous system either transcranially or via cranial or peripheral nerves (Includes transcutaneous vagal nerve stimulation)

4.4.2 External stimulation

Application of detectable or undetectable external stimulation of the body (includes vibration, heat, light, electromagnetic force

4.4.3 Aromatherapy

Intervention defined as aromatherapy

4.4.4 Touch-based

Therapies that involve the direct (or indirect) use of human touch / interaction. Includes massage, reiki etc. Typically delivered in CAMH settings. May include passive movement. *(This was placed in the classification but no studies were included)*

4.4.5 Acupuncture/pressure

Interventions using traditional chinese anatomical framework to deliver stimulation - acupuncture, acupressure etc.

4.5 Nutritional Interventions

4.5.1 Plant based

Non-pharmacological supplement described by source rather than ingredient (e.g. paeony extract, ginseng)

4.5.2 Nutritional Supplement

Non-pharmacological supplement described by ingredient rather than source (specifically: thiamine, fish oil, plant-based, 5-HTP, flavonoid (cocoa))

4.5.3 Diet

Specific dietary intervention (e.g. low-GI, anti-inflammatory)

4.6 Control Definitions

4.6.1 Information/Education

Provision of written / digital information with no more than one session of personal contact;

Provision and discussion / tailoring of written / digital information with more than one session of personal contact

4.6.2 Usual Care

Usual care or equivalent term either explicit or clearly implied.

4.6.3 Wait List

Use of wait list control. Note can include both cross-over design (where arms cross over and all followed to final FU and parallel with no follow up of 2nd arm active intervention.

4.6.4 Control

Includes attentional control (presumed inert activity to adjust for time / attention), unfocused discussion meetings and activities (e.g. writing). Also used as default term if not sufficiently clear; sham (use of inert external procedure); placebo (use of inert ingested substance)

5 Supplementary Methods 5: Focus Groups

5.1 Patient focus groups

We recruited 5 focus groups in order to reflect diversity of participants, clinical conditions, and location. Each group met on three occasions during the study, in early and mid 2024 and in early 2025. We conducted the focus groups using participatory approaches that we had previously found effective in PPI work with diverse patient groups, including using concise information summaries to inform interactive discussion and activities such as preference sorting. Ethics approval was obtained from the NHS Health Research Authority (23/SC/0292). The focus groups were co-led by an academic researcher (KF) and patient-researchers (DC & SM).

5.1.1 Participants and recruitment

Inclusion and exclusion criteria matched those of the systematic review. We used multiple approaches to ensure a diverse sample involving contacting patients through specialist clinics and recruitment through patient and other community organisations with a focus of ethnic minority heritage. We recruited through the patient organisations and community groups, particularly in communities of minority ethnic heritage. Invitations were sent as posters / flyers as organisations permitted with the opportunity to respond via email or by a dedicated phone number. Individuals who expressed an interest were then contacted and received further information prior to enrolment. Participants provided written consent before the first focus group and this was verbally confirmed at the start of each focus group.

5.1.2 Focus Groups

Focus groups were held online (3) and in community settings (2). The first round of focus groups gave participants the opportunity to describe their experiences of fatigue and to compare and contrast experiences across conditions. The second round focused on potential interventions and involved both description of experiences and discussion about a set of vignettes of potential fatigue interventions. The third round focused on communicating results of the evidence synthesis. The content of the groups was audio-recorded and transcribed before analysis which used thematic analysis. Developing findings were discussed within the study team.

5.2 Participant characteristics

The focus groups were recruited through patient organisations and community groups, specifically targeted non-white ethnic heritage. From 44 respondents, we recruited 25 (18 women 7 men) who were able to attend focus group which were held in person (2 groups) and online (3 groups) each on three separate occasions. While some individuals missed one of the series of three groups, none actively withdrew. Although we intended that people stay in the same group allowed people to move between groups and were struck that people were keen to continue their engagement.

Ages of participants ranged from under 30 to over 70, with the most represented age group being 50-59 years. We recruited from diverse ethnic heritages, with 10 identifying as South Asian, 8 as White, 5 as African-Caribbean and 2 others. Long term medical conditions reported included kidney or liver disease (6 participants), arthritis (5) diabetes (5) diabetes, heart conditions and neurological disorders (3 each).

5.3 Focus group findings informing the clinical effectiveness analysis

We drew on three themes in framing our analysing and reporting of clinical effectiveness.

1. Fatigue is an invisible problem. Few people had talked constructively about their fatigue with peers or with clinicians. There was little common language or models for explanation about fatigue and few people were aware that interventions for fatigue had been developed.

2. The experience of fatigue crosses diagnostic boundaries. Each individual's experience of fatigue was personal to them. Where similarities occurred with others, these were as much across conditions as within. Nonetheless, certain features of conditions affected the experience of fatigue or constrained the approaches that might be taken to reduce it.

3. There is no one-size-fits-all solution. Differences in the experience of fatigue extended to differences in what people had found helpful for them (or saw as appropriate to try). There were some instances of scepticism, particularly where an intervention conflicted with prior experiences or beliefs, however in general people were open to considering new information and to trying interventions if these were made available. Participants often ranked availability or accessibility as more important than the particular name or content of an intervention.

6 Supplementary Methods 6 - additional statistical analysis methods

6.1 Multiple fatigue measures

Some studies reported multiple measures of fatigue. The scale “FSS” was prioritised, following input from the clinical experts on the team, followed by “MFIS”. Therefore, if a study reported multiple fatigue outcomes using different scales, and if one of the outcomes was reported using the “FSS” scale, this data was selected and used for the network meta-analysis (NMA). However, if they did not use the “FSS” scale but used the “MFIS” scale, the data reported using the MFIS scale was selected for the NMA. This ruling resolved most cases, however in cases where there were multiple scales not including “FSS” or “MFIS”, clinical experts were consulted to obtain the most appropriate scale for inclusion within the NMA.

6.2 Evaluation of the SMD

As mentioned above, fatigue outcomes were measured using different scoring methods across studies. To facilitate the analysis of studies using different scoring methods within a single NMA, standardised mean differences (SMDs) of the change from baseline of fatigue, were calculated for each study. The use of SMDs is based on the assumption that all scoring scales are quantifying the same treatment effect and can be transformed onto a common scale by dividing the mean difference in change from baseline between the intervention and comparator within each study by the standard deviation of the difference.[1]

Raw data extracted from study results in the form of means, standard deviations, standard errors, inter quartile ranges and confidence intervals were used to evaluate the SMD and subsequently the standard error (SE) of the SMD for each study using Hedge’s correction.[1] In some studies, change from baseline was reported as opposed to pre and post intervention data, where available this data was extracted and used within the network.

Using intervention 1 as the reference treatment (for most analyses in this work, this corresponds to “usual care”), the SMD for the interventions in arm t, at follow-up f is given by

$$SMD_{t,f} = c \cdot \frac{\mu_{f,t} - \mu_{f,1}}{S},$$

$$S = \sqrt{\frac{(n_1 - 1)SD_1^2 + (n_t - 1)SD_t^2}{n_1 + n_t - 2}},$$

$$c = 1 - \frac{3}{4(n_1 + n_t) - 9}$$

where $\mu_{f,t}$ and $\mu_{f,1}$ is the change in fatigue score before and after treatment in arm t and arm 1 at follow-up f, respectively; S is the within group standard deviation pooled across arms, SD_t and SD_1 are the standard deviations in arm t and arm 1, respectively; n_t and n_1 are the number of participants at baseline in arm t and arm 1, respectively; and c is Hedges’ correction factor. The standard error (SE) of the SMD is given by

$$SE(SMD_{t,f}) = \sqrt{c^2\left(\frac{n_1 + n_t}{n_1 n_t} + \frac{SMD_{t,f}^2}{2(n_1 + n_t)}\right)}.$$

For studies where only the 95% confidence interval was presented alongside the mean instead of the standard deviation, the standard deviation was evaluated using

$$SD_t = \sqrt{n_t}\,\frac{CI_{t_{upper}} - CI_{t_{lower}}}{3.92},$$

where n_t is the number of participants in study arm t, $CI_{t_{upper}}$ and $CI_{t_{lower}}$ are the upper and lower 95% confidence intervals. For cases where only the inter-quartile range was recorded, the standard deviation was evaluated as

$$SD_t = \frac{IQR_{t_{upper}} - IQR_{t_{lower}}}{1.349},$$

where $IQR_{t_{upper}}$ and $IQR_{t_{lower}}$ are the upper and lower quartiles.[2] In most cases, fatigue scores were presented at baseline and then post-treatment, the mean change from baseline was therefore evaluated and the standard deviation of the mean change from baseline calculated using

$$SD_{change} = \sqrt{SD_{f,1}^2 + SD_{f,t}^2 - \left(2 \times corr \times SD_{f,1} \times SD_{f,t}\right)}$$

where the correlation coefficient ($corr$) was assumed to be equal to 0.5 as a conservative estimate.[3-5]

For studies where multiple arms presented data for the same intervention, according to the intervention classification conducted by the clinical experts, the data were combined. The mean change from baseline was evaluated as a weighted average, according to the number of participants in the arms being combined. The standard deviation was then evaluated as

$$SD = \sqrt{\frac{\left(N_{f,1} - 1\right)SD_{f,1}^2 + \left(N_{f,t} - 1\right)SD_{f,t}^2 + \frac{n_1 n_t}{n_1 + n_t}\left(M_{f,1}^2 + M_{f,t}^2 - 2M_{f,1}M_{f,t}\right)}{n_1 + n_t - 1}},$$

where $M_{f,t}$ is the weighted average of the mean change from baseline in arm t at follow-up f.[5]

In the case where no available data were available to evaluate the mean and standard deviation of the change from baseline of the fatigue score for each arm, the study was not included within the NMA. No studies which only presented data graphically were included within the NMA due to the high number of studies.

6.3 Statistical model for the NMA

A random-effects NMA model was used to account for between study heterogeneity.[6] Let y_{ik} denote the standardised mean difference (SMD) of arm k of trial i, where $k = 1, \dots, na$ and $i = 1, \dots, ns$, with variance V_{ik}. Here, na and ns correspond to the number of arms and the number of studies respectively. We assume that the treatment effects are normally distributed according to

$$y_{ik} \sim \mathcal{N}(\theta_{ik}, V_{ik}),$$

where $\boldsymbol{\theta}$ are the parameters of interest. The individual θ_{ik} are modelled using the identity link function as they are continuous on the entire real line

$$\theta_{ik} = \delta_{i,1k},$$

where $\delta_{i,1k}$ is the individual study treatment effect of intervention k relative to intervention 1 in study i. To allow for heterogeneity of treatment effects across studies, a random-effects model was assumed. The random-effects model was structured such that all individual study treatment effects arise from a common normal distribution centred about a mean population treatment effect, with some variance τ^2

$$\delta_{i,1k} \sim \mathcal{N}\left(d_{t_{i1},t_{ik}}, \tau^2\right),$$

where $d_{t_{i1},t_{ik}}$ is the mean effect of the intervention in arm k of study i (t_{ik}) compared to the intervention in arm 1 of study i (t_{i1}).

In the case of studies with more than two arms, adjustment to the likelihood (function) was necessary to account for the correlation between multiple comparisons to arm 1 and was included via the assumption that the covariance between two comparisons relative to treatment 1 can be approximated as $^1/_{n_{i,1}}$, where $n_{i,1}$ is the number of participants in arm 1 of study i at baseline.[7]

6.4 Definition of priors

Parameters were estimated using a Bayesian framework, as such, weakly-informative priors were chosen for the between-study variance of treatment effects

$$\tau \sim \mathcal{U}(0,5) \cdot \frac{\sqrt{3}}{\pi}.$$

The $\sqrt{3}/_{\pi}$ factor is included to account for the transformation of τ between the odds-ratio scale and the SMD scale.[8] An informative prior, a truncated log-normal prior on τ^2 was used in cases where there were less than 5 studies within the connected network.[8]

$$\tau^2 \sim \text{lognormal}(-2.56, 0.33) \cdot I(0,1).$$

The prior on the mean treatment effects were defined as

$$d_{t_{i1},t_{ik}} \sim \mathcal{N}(0, 100^2)$$

6.5 Implementation

All analyses were conducted using the freely available software package WinBUGS[9] and R, via the R2Winbugs[10] interface package. Model code was modified from NICE technical support document 2.[11] Convergence to the target posterior distributions was assessed using the Gelman-Rubin statistic, as modified by Brooks and Gelman, for three chains with different initial values.[12] The autocorrelation of samples from the burn-in period was also assessed for any significant autocorrelation which requires sample thinning. A burn-in period of 50,000 samples was implemented, with a further 1,000,000 samples after the burn-in period. The samples after the burn-in were subject to a thinning by a factor of 10.

Results are presented using the posterior median treatment effects and 95% credible intervals (CrI).

The validity of the inconsistency assumption was assessed by comparing the posterior mean residual deviance from the unrelated mean effects model and the NMA model; and node-splitting analysis. The posterior means of the deviance contributions for the unrelated mean effects model and the NMA model were plotted, cases where the posterior means lie across the $y = x$ line, demonstrated that the inconsistency assumption held. Cases where the posterior deviance contributions deviated from this line (indicating an improvement greater than 0.5 points in the unrelated mean effects model) were investigated using node-splitting via the gemtc package in R.[13]

6.6 References

1. Higgins JPT, Altman DG, Sterne JAC (editors). Chapter 8: Assessing risk of bias in included studies. In: Higgins JPT, Churchill R, Chandler J, Cumpston MS (editors), Cochrane Handbook for Systematic Reviews of Interventions version 5.2.0 (updated June 2017), Cochrane, 2017. Available from www.training.cochrane.org/handbook.

2. Wan, X., Wang, W., Liu, J., & Tong, T. (2014). Estimating the sample mean and standard deviation from the sample size, median, range and/or interquartile range. *BMC medical research methodology*, *14*, 1-13.
3. Follmann, D., Elliott, P., Suh, I. L., & Cutler, J. (1992). Variance imputation for overviews of clinical trials with continuous response. *Journal of clinical epidemiology*, *45*(7), 769-773.
4. Pearson, M. J., & Smart, N. A. (2018). Reported methods for handling missing change standard deviations in meta-analyses of exercise therapy interventions in patients with heart failure: A systematic review. *PLoS One*, *13*(10), e0205952.
5. Higgins JPT, Thomas J, Chandler J, Cumpston M, Li T, Page MJ, Welch VA (editors). *Cochrane Handbook for Systematic Reviews of Interventions* version 6.5 (updated August 2024). Cochrane, 2024. Available from www.training.cochrane.org/handbook.
6. Dias S, Sutton AJ, Ades AE, Welton NJ. Evidence Synthesis for Decision Making 2: A Generalized Linear Modeling Framework for Pairwise and Network Meta-analysis of Randomized Controlled Trials. Med Decis Making. 2013;33(5):607-17.
7. Efthimiou, O., Mavridis, D., Riley, R. D., Cipriani, A., & Salanti, G. (2015). Joint synthesis of multiple correlated outcomes in networks of interventions. *Biostatistics*, *16*(1), 84-97.
8. Ren, S., Oakley, J. E., & Stevens, J. W. (2018). Incorporating genuine prior information about between-study heterogeneity in random effects pairwise and network meta-analyses. *Medical Decision Making*, *38*(4), 531-542.
9. Lunn, D. J., Thomas, A., Best, N., & Spiegelhalter, D. (2000). WinBUGS-a Bayesian modelling framework: concepts, structure, and extensibility. *Statistics and computing*, *10*, 325-337.
10. Sturtz, S., Ligges, U., & Gelman, A. (2005). R2WinBUGS: a package for running WinBUGS from R. *Journal of Statistical software*, *12*, 1-16.
11. Dias, S., Welton, N.J., Sutton, A.J. & Ades, A.E. NICE DSU Technical Support Document 2: A Generalised Linear Modelling Framework for Pairwise and Network Meta-Analysis of Randomised Controlled Trials. 2011; last updated September 2016; available from http://www.nicedsu.org.uk
12. Brooks SP, Gelman A. General Methods for Monitoring Convergence of Iterative Simulations. Journal of Computational and Graphical Statistics. 1998;7(4):434-55.
13. van Valkenhoef, G., Dias, S., Ades, A. E., & Welton, N. J. (2016). Automated generation of node-splitting models for assessment of inconsistency in network meta-analysis. Research synthesis methods, 7(1), 80-93.
