# Supplementary material for: Effectiveness of non-pharmacological interventions for fatigue in long term conditions: systematic review and network meta-analysis
Source: BMJ Med. 2026 Mar 13;5(1):e001746. doi: 10.1136/bmjmed-2025-001746 (PMC12993367; doi:10.1136/bmjmed-2025-001746)
Supplement: Supplementary file 3 [file bmjmed-2025-001746.reviewer_comments.pdf]

**PEER REVIEW HISTORY**

BMJ Medicine publishes all reviews undertaken for accepted manuscripts. Reviewers are asked to complete a checklist review form and are provided with free text boxes to elaborate on their assessment. These free text comments are reproduced below.

**ARTICLE DETAILS**

| | |
|---|---|
| **TITLE (PROVISIONAL)** | Effectiveness of non-pharmacological interventions for fatigue in long term conditions: systematic review and network meta-analysis |
| **AUTHORS** | Burton, Christopher; Leaviss, Joanna; Forsyth, Jessica; Booth, Andrew; Coyle, David; Daly, George; Davis, Sarah; Dawes, Helen; Deary, Vincent; Dwivedi, Kritica; Fryer, Kate; McCormick, Samantha; Martyn-St James, Marissa; Newton, Julia; Ren, Shijie; Rooney, Gillian; Sutton, Anthea; Mon-Yee, Mon |

**VERSION 1 - REVIEW**

| | |
|---|---|
| **REVIEWER NAME** | Mair, Jacqueline Louise |
| **REVIEWER AFFILIATION** | Singapore-ETH Centre |
| **REVIEWER CONFLICT OF INTEREST** | None |
| **AI DISCLOSURE** | No |
| **DATE REVIEW RETURNED** | 17-Jul-2025 |

| | |
|---|---|
| **GENERAL COMMENTS** | Thank you for the opportunity the review this paper and congratulations on conducting such an immense and important piece of work. The findings will certainly have impact for patients, clinicians, and beyond. Overall, the review has been executed to a very high standard and the inclusion of PPI groups is commendable. The article is very well written in terms of style and grammar. With some revisions I believe the article will be of interest to the readership of BMJ Medicine. I have a few suggestions that I think could improve the paper.<br><br>Major points:<br><br>I recommend using the PRISMA-NMA extension reporting checklist and including this completed checklist as part of the supplementary files. While many elements of the review are well described, there are some aspects specific to NMA that are missing. See Hutton B, Salanti G, Caldwell DM, Chaimani A, Schmid CH, Cameron C, Ioannidis JP, Straus S, Thorlund K, Jansen JP, Mulrow C, Catalá-López F, Gøtzsche PC, Dickersin K, Boutron I, Altman DG, Moher D. The PRISMA Extension Statement for Reporting of Systematic Reviews Incorporating Network Meta-analyses of Health Care Interventions: Checklist and Explanations. Ann Intern Med. 2015;162(11):777-784. doi: 10.7326/M14-2385<br><br>This is an important evidence synthesis of intervention effectiveness for fatigue and has clear impact for the clinical community and beyond. However, given the importance of the message to be conveyed I feel that the interventions are overly simplified and could lead to misunderstanding. These are largely behavioural or holistic |

| | |
|---|---|
| | interventions with multiple components and are likely highly complex in nature. Yet they have been grouped together under relatively broad categories. I don’t mean this as a criticism and I fully understand there is a need to group intervention for analysis purposes but there are many factors that can influence the effectiveness of an intervention within a group, such as the mode of delivery, intervention dose, degree of personalisation, adherence rates, incentives etc. I appreciate there is some discussion of this limitation, but I am a little concerned about the overall message. I think one of the most important ones is: “We found no single clear best intervention and from our parallel focus group work and qualitative evidence synthesis recognise that offering patients a choice of interventions is better”. I think you want to avoid a message that only certain things work and other approaches do not, and kindly ask you to consider this overall message that you want to convey and whether it might be necessary to draw atten attention aspects of your work.<br><br><br>Minor points:<br><br>The supplementary file is still in draft form – there are some unresolved comments to address. Please check these files for completeness.<br><br>Page 6 L45: Suggest to include ‘United Kingdom’ for international readers.<br><br>Page 6 L53: At “participants consented to participation and groups were recorded and transcribed for analysis” - suggest to substitute ‘groups’ for ‘discussions’<br><br>Page 8 L43: Suggest not to start a sentence with a numeral.<br><br>Page 8 L48: A Cohen’s Kappa of 0.58 suggests only moderate agreement with title/abstract screening decisions– I note that discrepancies are explained for full text screening, but I think it would be worth explaining where the discrepancies lay between reviewers at this stage too.<br><br>Page 9 L53: “We used a threshold of an SMD of 0.34 as clinically meaningful (see below for rationale)” - this is a little cumbersome to read, please revise this sentence and check if SMD has been written in full previously.<br><br>Page 10 L14: “First a simple description of the interventions in each arm was recorded by during data extraction” - please check this – it seems like a word is missing.<br><br>Page 10 L12: The paragraph describing the intervention classification process is a little ambiguous (e.g. reference to categories and criteria without context) and needs to be read in conjunction with the supplementary file to gain a clear understanding of the method used. I wonder if you can explain the process with a little more detail for the benefit of the main text? You may consider describing interventions using an ontology, given these are non-pharmacological and multi-component interventions which can be complex and overlapping (https://www.bciontology.org/). |

| | |
|---|---|
| | Throughout the results, could you ensure consistency of reporting decimal places.<br><br>Page 14 L25: For active fatigue management, could you include the SMD and CrI for non-significant findings too – e.g. “but this was not sustained to short term (2 studies)”.<br><br>Page 14 L26: By “Conservative self-management" I think you mean Fatigue self-management (energy conservation) as you categorise in the supplementary file - I think it is important to be very clear with the description of the intervention and keep terminology (or classification of interventions) consistent to ensure clear reporting of specific interventions and their effects.<br><br>Page 14 L32: For mindfulness interventions, were there no short term follow up data available? Please could you confirm.<br><br>Page 14 L48 – regarding non-invasive stimulation and nutrition interventions – could you also report the SMD and CrI here?<br><br>P15 L23- minor grammar correction needed here “Re-analysing the longer term data, instead using the shortest follow up point after 3 months had minimal impact on the results of the NMA”<br><br>P16 L20 - “interventions that increase physical activity” - please revise to interventions that promote physical activity.<br><br>P16 L23 - “self-management OF fatigue”?<br><br>P18 Conclusion: “Interventions which support individuals to increase physical activity or that are based on cognitive behavioural are effective in reducing fatigue in people with long-term medical conditions”- missing the word ‘therapy’<br><br>References are listed twice. |

| | |
|---|---|
| **REVIEWER NAME** | Abonie, Ulric |
| **REVIEWER AFFILIATION** | |
| **REVIEWER CONFLICT OF INTEREST** | None |
| **AI DISCLOSURE** | No |
| **DATE REVIEW RETURNED** | 25-Aug-2025 |

| | |
|---|---|
| **GENERAL COMMENTS** | The manuscript has strong potential, but it requires improvements in multiple sections, particularly in clarity, transparency of reporting, and adherence to systematic review best practices. The PRISMA guidelines were followed to some extent, but some aspects need to be addressed.<br>Abstract<br>Include more specific details about the search period (e.g., “from database inception to September, 2024”).<br>The conclusion should mention the heterogeneity observed, as this affects the interpretation of the meta-analysis results.<br>Introduction<br>Expand on the gap in the literature. While previous condition specific reviews are mentioned, more detail on the need for the same intervention across different conditions is needed. |

| | |
|---|---|
| | Clarify the specific research question or hypotheses. This will help guide the reader and align with PRISMA's recommendation for a clear statement of objectives.<br>Methods<br>Clearly state the information sources and dates of the last search. Mention the specific date ranges for each database search.<br>Provide more detail on the network meta-analysis approach, particularly regarding how you handled heterogeneity and the rationale for using the random-effects model.<br>Results<br>Describe the characteristics of the included studies in more detail. For example, include information on the sample size range and the settings of the interventions.<br>Address the potential impact of heterogeneity.<br>Discussion<br>Provide a more detailed discussion of the potential sources of heterogeneity observed in the network meta-analysis. Discuss factors such as differences in intervention types, duration, and participant characteristics.<br>Conclusion<br>Emphasize the need for better-designed RCTs that address the limitations identified in this review, particularly the issue of small number of most condition specific studies and heterogeneity.<br>Figures and Tables<br>Strengths: The figures and tables are informative and well-organized.<br>Improvements: Ensure all acronyms are defined in the table legends (MSK, IBS, Endo). |

| | |
|---|---|
| **REVIEWER NAME** | Dolgoy, Naomi |
| **REVIEWER AFFILIATION** | |
| **REVIEWER CONFLICT OF INTEREST** | None |
| **AI DISCLOSURE** | No |
| **DATE REVIEW RETURNED** | 09-Sep-2025 |

| | |
|---|---|
| **GENERAL COMMENTS** | This was a really interesting and well described review that contributes to the literature on fatigue. Thank you for the opportunity to read and offer suggestions.<br><br>I had four considerations:<br>- (1) Abstract conclusion (page 6, line 4) cognitive behavioural – Is the next word missing – Therapies, techniques, approaches?<br>- (2) General comment: would suggest to screen the paper for punctuation (noticed some missing or erroneous periods etc. at the end of the abstract, in the introduction, on page 15 lines 17 and 24).<br>- (3) Page 17, lines 5-6 "within the short and longer term networks (0.079, 0.004 to 0.308) and (0.096, 0.005 to 0.356)" – would suggest to revise to "within the short (0.079, 0.004 to 0.308) and longer term (0.096, 0.005 to 0.356) networks" for clarity.<br>-(4) Suggestion for the Discussion – the results on Page 17 lines 10-14: "This suggests that there are generally smaller differences between the study design, interventions and populations in the short and longer term networks compared to the end of treatment network." may be a place for further expansion. For the network meta-analysis and clinically relevant outcomes, these findings on the consistency and comparability across the studies was very interesting to read about. Could these findings point to challenges or |

| | |
|---|---|
| | opportunities in designing research or interventions across populations? Could these be more explicitly connected in the Discussion? |

| | |
|---|---|
| **REVIEWER NAME** | Riley, Richard |
| **REVIEWER AFFILIATION** | University of Birmingham, Institute of Applied Health Research |
| **REVIEWER CONFLICT OF INTEREST** | None |
| **AI DISCLOSURE** | No |
| **DATE REVIEW RETURNED** | 18-Sep-2025 |

| | |
|---|---|
| **GENERAL COMMENTS** | Thank you for the opportunity to review this article for BMJ Medicine. The authors have clearly worked hard to undertake this large review and network meta-analysis. I have reviewed this from a statistical perspective and have some comments for improvement.<br><br>1) Rather than focusing on statistical significance to make firm statements about benefit or not, I encourage the authors to consider the strength of evidence and that areas for further research are needed. For example, results such as '(-0.42, -0.9 to 0.09) are firmly labelled as no statistically significant benefit, when there is actually further research is warranted as the majority of the CI is in favour of a benefit.<br>2) The abstract should discuss whether there is heterogeneity or inconsistency in the network meta-analyses.<br>3) Please be consistent in using two decimal places for the effect estimates, and not sometimes 1<br>4) The authors use PRISMA but should have used PRISMA-NMA for the reporting guideline.<br>5) 'Study heterogeneity was assessed and interpreted using established categories' – this is too vague and we need to know exactly how it was assessed, quantified and what these 'categories' are.<br>6) Apart from to reduce time, it is unclear why the authors modified ROB2 from 22 to 15 items, and the impact this may have had on the ROB classifications could be considerable.<br>7) 'non-informative parameter priors' were used, but I do not think any prior for the between-study variance can be called 'non-informative' in a meta-analysis setting. Please tell the reader what these priors were and why they were chosen, and in particular justify that used for the between-study variance parameter.<br>8) It is unclear whether the model allowed one between-study variance (assumed the same for all comparisons) or otherwise. (I appreciate some of these queries are addressed in the lovely supp material section, but it needs to be addressed in the main paper for the reader too, as it is important to the robustness of the findings)<br>9) I wonder if the transitivity of the network was considered before analysis, for example by comparing the distribution of potential effect modifiers across studies with different sets of comparisons.<br>10) I wonder, would the results be improved by also presented rankograms that detail the probabilities of each intervention being first, second, third, …, last etc?<br>11) What if a study analysed the fatigue outcome as a dichotomised measure? How was this then converted to the continuous (standard mean difference) scale? E.g. if only the number of people above and below a fatigue cutpoint were reported for each group, but not the means and SDs of fatigue score.<br>12) 'Figures 4 and 5 show the predicted SMDs' – the word predicted |

| | |
|---|---|
| | is a bit strange, I think estimated (or strictly the median?) is better. Otherwise, it sounds like a prediction interval is being referred to, which I do not think is actually being done? (though given the heterogeneity, it may also be helpful to present prediction intervals not just confidence intervals)<br>13) There is no examination of small study effects (potential publication bias)<br>14) As many studies are not at low risk of bias, I think sensitivity analyses considering the impact of ROB on the findings would be welcome<br><br>I hope these comments are constructive and allow the authors to improve their article at revision. |

**VERSION 1 – AUTHOR RESPONSE**

| **Reviewer 1:** | |
|---|---|
| I recommend using the PRISMA-NMA extension reporting checklist and including this completed checklist as part of the supplementary files | Many thanks to the reviewer for suggesting the use of the PRISMA-NMA checklist, we have replaced our original PRISMA checklist in the supplementary files. Some small clarifications on Items S1, S2 and S4 in the checklist were also included in the manuscript, see pages 11,12,13,14. |
| The interventions are overly simplified and could lead to misunderstanding. These are largely behavioural or holistic interventions with multiple components and are likely highly complex in nature. Yet they have been grouped together under relatively broad categories. "We found no single clear best intervention and from our parallel focus group work and qualitative evidence synthesis recognise that offering patients a choice of interventions is better". I think you want to avoid a message that only certain things work and other approaches do not, and kindly ask you to consider this overall message. | We have added more detail about the classification in the methods (see also comment by another reviewer regarding this), and hope this gives the reviewer greater confidence in the validity of the categories – page 10.<br><br>We note the need for caution and have added in a paragraph at the end of the discussion to reflect this – page 21. |
| The supplementary file is still in draft form – there are some unresolved comments to address | We have now resolved the previous unresolved comments. Anything that remains relates to the tracked changes for the current revisions. |
| Page 6 L45: Suggest to include 'United Kingdom' for international readers. | Added as suggested |
| Page 6 L53: At "participants consented to participation and groups were recorded and transcribed for analysis" - suggest to substitute 'groups' for 'discussions' | Amended as suggested |

| | |
|---|---|
| Page 8 L43: Suggest not to start a sentence with a numeral. | Amended as suggested |
| Page 8 L48: A Cohen's Kappa of 0.58 suggests only moderate agreement with title/abstract screening decisions– I note that discrepancies are explained for full text screening, but I think it would be worth explaining where the discrepancies lay between reviewers at this stage too. | Generally the moderate kappa was a result of one reviewer being more 'dove-ish' compared to one reviewer being more 'hawk-ish'. Double sifting meant that every conflict was discussed with reference to the clinical team where ambiguity remained. The following text has been added to page 9.<br><br>"Whilst this kappa suggests moderate agreement, double-sifting meant that each disagreement was discussed to reach resolution. Generally, conflicts were the result of one reviewer being consistently over-inclusive. Common ambiguities were referred to the clinical experts, for example whether a nutritional intervention was classed as a 'supplement' or the dose was too high and should be excluded as a pharmacological intervention." |
| Page 9 L53: "We used a threshold of an SMD of 0.34 as clinically meaningful (see below for rationale)" - this is a little cumbersome to read, please revise this sentence and check if SMD has been written in full previously. | We have amended the sentence as recommended to improve the clarity of the sentence and added the definition of "SMD" at the first point it is mentioned in the manuscript, thank you for highlighting. |
| Page 10 L14: "First a simple description of the interventions in each arm was recorded by during data extraction" - please check this – it seems like a word is missing. | Thank you for spotting this error, we have corrected this to include the missing reviewers' names. |
| Page 10 L12: The paragraph describing the intervention classification process is a little ambiguous (e.g. reference to categories and criteria without context) and needs to be read in conjunction with the supplementary file to gain a clear understanding of the method used. I wonder if you can explain the process with a little more detail for the benefit of the main text? You may consider describing interventions using an ontology, given these are non-pharmacological and multi-component interventions which can be complex and overlapping (https://www.bciontology.org/). | We have added an introductory rationale for this approach and some further detail within the methods. Hopefully that means the reader understands what we did and why before checking details in the supplementary material – page 10. |
| Throughout the results, could you ensure consistency of reporting decimal places. | We have ensure consistency to 2dp within the text, and 3dp in the Forest plots. |

| | |
|---|---|
| Page 14 L25: For active fatigue management, could you include the SMD and CrI for non-significant findings too – e.g. “but this was not sustained to short term (2 studies)”. | We have now added the SMD and CrI for the non-significant findings:<br><br>“but this was not sustained to short term (-0.11, -0.79 to 0.54, 2 studies)” |
| Page 14 L26: By “Conservative self-management" I think you mean Fatigue self-management (energy conservation) as you categorise in the supplementary file - I think it is important to be very clear with the description of the intervention and keep terminology (or classification of interventions) consistent to ensure clear reporting of specific interventions and their effects. | Thank you for spotting this – we have now ensured that the intervention names are consistent in the main text and the supplementary file. |
| Page 14 L32: For mindfulness interventions, were there no short term follow up data available? Please could you confirm. | We confirm that there was no available short term follow up data for the mindfulness intervention. To ensure this is clear to readers, we have added the following sentence to page 17: “*Note that no studies evaluating mindfulness at short term were identified for inclusion within the short term analysis.”* |
| Page 14 L48 – regarding non-invasive stimulation and nutrition interventions – could you also report the SMD and CrI here? | We have now added this detail in |
| P15 L23- minor grammar correction needed here “Re-analysing the longer term data, instead using the shortest follow up point after 3 months had minimal impact on the results of the NMA | We have reworded this paragraph to correct the grammar. |
| P16 L20 - “interventions that increase physical activity” - please revise to interventions that promote physical activity. | Thank you – we have revised this as requested |
| P16 L23 - “self-management OF fatigue”? | Yes – we have corrected |
| P18 Conclusion: “Interventions which support individuals to increase physical activity or that are based on cognitive behavioural are effective in reducing fatigue in people with long-term medical conditions” - missing the word ‘therapy’ | We have corrected this |
| **Reviewer 2:** | |

| | |
|---|---|
| Abstract - Include more specific details about the search period (e.g., "from database inception to September, 2024"). | The search dates have been added to the abstract. |
| The conclusion should mention the heterogeneity observed, as this affects the interpretation of the meta-analysis results. | We have added in a fuller presentation and discussion of the observed heterogeneity between studies throughout the results and discussion sections. |
| Introduction - Expand on the gap in the literature. While previous condition specific reviews are mentioned, more detail on the need for the same intervention across different conditions is needed | This is similar to a comment from the editor. We have expanded the opening of the introduction to explain why it is reasonable to consider fatigue in a transdiagnostic way (analogous to chronic pain). We also state that we found no reviews of the effectiveness of other interventions across multiple conditions – page 5. |
| Introduction - Clarify the specific research question or hypotheses. This will help guide the reader and align with PRISMA's recommendation for a clear statement of objectives. | We have reworded the introduction and have included the statement on page 6:<br><br>"We conducted a systematic review and network meta-analysis to investigate the research question: What is the clinical effectiveness of non-pharmacological interventions for fatigue in long term conditions?" |
| Methods - Clearly state the information sources and dates of the last search. Mention the specific date ranges for each database search. | All search dates and coverage of databases have now been included in Table 1. Please note that CENTRAL does not have a start date due to the composite nature of the database. See: Cochrane Controlled Register of Trials (CENTRAL) \| Cochrane Library |
| Methods - Provide more detail on the network meta-analysis approach, particularly regarding how you handled heterogeneity and the rationale for using the random-effects model. | Thank you to the reviewer for providing this comment, in line with this suggestion and reviewer 4's comments, we have expanded the details on the NMA approach – page 11,12. |
| Results - Describe the characteristics of the included studies in more detail. For example, include information on the sample size range and the settings of the interventions | We have added in a new section to the results with a fuller discussion of the sources of heterogeneity identified in the included studies, and referred to the supplementary materials for a full account of each study's characteristics by PICO – page 13,14. |
| Results - Address the potential impact of heterogeneity | We have now addressed this as described above. |
| Discussion - Provide a more detailed discussion of the potential sources of heterogeneity observed in the network meta -analysis. Discuss factors such as | We have now provided a fuller discussion of the observed heterogeneity both in the results section (see comment above) and in the discussion – page 20,21. |

| | |
|---|---|
| differences in intervention types, duration, and participant characteristics. | |
| Conclusion - Emphasize the need for better-designed RCTs that address the limitations identified in this review, particularly the issue of small number of most condition specific studies and heterogeneity. | We have added these points into the conclusions – page 22. |
| Figures and Tables<br>Improvements: Ensure all acronyms are defined in the table legends (MSK, IBS, Endo). | We have now defined these |
| **Reviewer 3:** | |
| Abstract conclusion (page 6, line 4) cognitive behavioural – Is the next word missing – Therapies, techniques, approaches? | Yes – thank you, changed to therapies |
| General comment: would suggest to screen the paper for punctuation (noticed some missing or erroneous periods etc. at the end of the abstract, in the introduction, on page 15 lines 17 and 24) | Thank you – we have checked and corrected where appropriate |
| Page 17, lines 5-6 "within the short and longer term networks (0.079, 0.004 to 0.308) and (0.096, 0.005 to 0.356)" – would suggest to revise to "within the short (0.079, 0.004 to 0.308) and longer term (0.096, 0.005 to 0.356) networks" for clarity. | We have revised as suggested. |
| Suggestion for the Discussion – the results on Page 17 lines 10-14: "This suggests that there are generally smaller differences between the study design, interventions and populations in the short and longer term networks compared to the end of treatment network." may be a place for further expansion. For the network meta-analysis and clinically relevant outcomes, these findings on the consistency and comparability across the studies was very interesting to read about. Could these findings point to challenges or opportunities in designing research or interventions across populations? Could these be more explicitly connected in the Discussion? | We realise the statement about heterogeneity and follow up durations was rather unclear.<br><br>We have edited the discussion to state "The reduction in observed heterogeneity between studies with longer follow up underlines the importance of adequate study duration to generate robust conclusions." We think this also makes a key point about future studies – page 20.<br><br>The final statement of the recommendations for research is that "We recommend that further research focuses on delivery of interventions in a transdiagnostic format." – page 21. |
| **Reviewer 4:** | |

| | |
|---|---|
| Rather than focusing on statistical significance to make firm statements about benefit or not, I encourage the authors to consider the strength of evidence and that areas for further research are needed. For example, results such as '(-0.42, -0.9 to 0.09) are firmly labelled as no statistically significant benefit, when there is actually further research is warranted as the majority of the CI is in favour of a benefit. | We have thought hard about this challenge of interpreting the data. The reviewer picks up on the most challenging example of this (energy conservation-based management). The reason we had adopted a more critical stance towards this was that the effect sizes observed at end of treatment and short term were considerably weaker (in contrast to the physical activity and CBT-based interventions). Had values for the energy conservation interventions been similar across time points (and closer to the clinically important difference) we would have been more inclined to consider it approximately equivalent to the others. We have, however, been a little more cautious in our phrasing about this. |
| The abstract should discuss whether there is heterogeneity or inconsistency in the network meta-analyses. | Two clarifying statements relating to the degree of heterogeneity and inconsistency within the networks have been added to the abstract. |
| Please be consistent in using two decimal places for the effect estimates, and not sometimes 1 | We have ensured consistency to 2dp within the text (3dp in the Forest plots) |
| The authors use PRISMA but should have used PRISMA-NMA for the reporting guideline. | We have replaced our original PRISMA checklist in the supplementary files. Some small clarifications on Items S1, S2 and S4 in the checklist were also included in the manuscript, see pages 11,12,13,14. |
| 'Study heterogeneity was assessed and interpreted using established categories' – this is too vague and we need to know exactly how it was assessed, quantified and what these 'categories' are. | Thank you for highlighting this lack of detail, we have added a sentence to clarify the source of the categories as well as their limits – page 12. |
| Apart from to reduce time, it is unclear why the authors modified ROB2 from 22 to 15 items, and the impact this may have had on the ROB classifications could be considerable. | Whilst we adapted the RoB2 tool for pragmatic reasons, this was also due in part to the nature of the body of evidence that we identified. RoB2 uses stringent criteria and works well identifying detailed risks within the specific domains. There are inherent risks of bias for these types of interventions, such as blinding not being possible, and for these, some of the signalling questions were judged to be more than was required in order for the domain to be judged as high risk. With this in mind, we abridged the RoB2 tool to remove some of the signalling questions that we judged would not distinguish the risks between the studies in any meaningful way, whilst still capturing the key issues with methodological conduct, reporting and other potential biases we observed in the majority of RCTs that were included in the present NMA. Whilst the tool allows for some reviewer judgement in |

| | |
|---|---|
| | assessing the body of evidence a whole – i.e. within review assessment where all studies may have the same inherent risks in a particular domain, the robust nature of the RoB2 tool also means that any 'red' high risk domain means the study scores high risk of bias overall. The nature of the studies included in this review meant that all but one study was judged to be at high risk of bias. We considered sensitivity analyses to account for risk of bias either overall or by specific domains, but, as described below in response to another comment, we were unable to construct meaningful networks for any of the timepoints. We acknowledge that this is a limitation to our findings and have added more text into the discussion to make this clearer – page 20. |
| 'non-informative parameter priors' were used, but I do not think any prior for the between-study variance can be called 'non-informative' in a meta-analysis setting. Please tell the reader what these priors were and why they were chosen, and in particular justify that used for the between-study variance parameter. | Thank you for the comment, we have updated the description of the priors to "weakly-informative" – page 12, and provided additional details on the definitions of the priors which were previously only included in the supplemental methods. |
| It is unclear whether the model allowed one between-study variance (assumed the same for all comparisons) or otherwise. (I appreciate some of these queries are addressed in the lovely supp material section, but it needs to be addressed in the main paper for the reader too, as it is important to the robustness of the findings) | We have clarified this in the manuscript: the model used a single, shared between-study variance (i.e. a common heterogeneity parameter across all comparisons), which we selected due to the large number of interventions and sparsity in parts of the network – page 11,12. |
| I wonder if the transitivity of the network was considered before analysis, for example by comparing the distribution of potential effect modifiers across studies with different sets of comparisons. | We recognise that a strong network transitivity assumption has been made in this study, chiefly with regard to the transdiagnostic assumption. The condition of interest across the included studies was considered as a potential effect modifier, along with other covariates such as age, sex and other comorbidities. Following the review protocol, the potential treatment effect modifiers were compared across the included studies. We identified potential sources of heterogeneity before the statistical analyses - for example differences in delivery of interventions, differences in demographic characteristics of participants etc. Naturally, the condition of interest did vary across studies. |

| | |
|---|---|
| | The review protocol stated that, in the case of moderate to large heterogeneity, a meta-regression based on conditions and sociodemographic data would be performed where compatible data allowed. However, it was quickly recognised that several conditions were not evidenced in areas of the network and that extrapolating potential effect modifiers into these areas whilst lacking any evidence would be inappropriate.<br><br>Accordingly, condition-specific treatment effects were explored through condition-specific networks within a set of scenario analyses and presented in the supplementary results; these allow for a comparison of the transdiagnostic evidence against the (limited) condition-specific evidence. Inevitably, the only way to verify or invalidate the transdiagnostic assumption would be to collect data in a transdiagnostic setting which is one of the key recommendations from this review.<br><br>Finally, with regard to other potential effect modifiers, e.g. age, sex, etc, meta-regression was not feasible due to incompatible reporting of subgroups/covariates (including lack thereof) |
| I wonder, would the results be improved by also presented rankograms that detail the probabilities of each intervention being first, second, third, …, last etc? | Thank you for the suggestion, we have included rankograms for the primary analyses at end of treatment, short term and long term follow up in the supplementary materials. |
| What if a study analysed the fatigue outcome as a dichotomised measure? How was this then converted to the continuous (standard mean difference) scale? E.g. if only the number of people above and below a fatigue cutpoint were reported for each group, but not the means and SDs of fatigue score. | We can confirm that none of the studies included in the NMA analysed fatigue as a dichotomised measure. |
| 'Figures 4 and 5 show the predicted SMDs' – the word predicted is a bit strange, I think estimated (or strictly the median?) is better. Otherwise, it sounds like a prediction interval is being referred to, which I do not think is actually being done? (though given the heterogeneity, it may also be helpful to | All references to "predicted" treatment effects or SMDs have been amended to "estimated" treatment effects or SMDs. Thank you also for the helpful comment to include the predictive intervals in the forest plots for additional context given the heterogeneity within the network, we have incorporated these in all forest plots. We have also |

| | |
|---|---|
| present prediction intervals not just confidence intervals) | added a note to the methods explaining how predictive intervals can be interpreted. |
| There is no examination of small study effects (potential publication bias) | Thank you for highlighting this gap in our assessment of the reported analyses. As many of the interventions used alternative “reference” or “comparator” treatments there was not a single obvious choice for a reference treatment for the use within comparison-adjusted funnel plots. We therefore opted instead to conduct further sensitivity analyses by excluding all studies with less than 30 participants. We have referenced these analyses in the main text – page 12, and report the full results in the supplementary results file. |
| As many studies are not at low risk of bias, I think sensitivity analyses considering the impact of ROB on the findings would be welcome | We attempted a sensitivity analysis excluding studies at high risk of bias; however, this left too few studies to form a meaningful, connected network at any time point, making a valid NMA infeasible. We have added clarification to the Strengths and Limitations section in the Discussion (page 20,21) to reflect this and to emphasise cautious interpretation of the findings. |
